# Prognostic models in COVID-19 infection that predict severity: a systematic review

**DOI:** 10.1007/s10654-023-00973-x

**Published:** 2023-02-25

**Authors:** Chepkoech Buttia, Erand Llanaj, Hamidreza Raeisi-Dehkordi, Lum Kastrati, Mojgan Amiri, Renald Meçani, Petek Eylul Taneri, Sergio Alejandro Gómez Ochoa, Peter Francis Raguindin, Faina Wehrli, Farnaz Khatami, Octavio Pano Espínola, Lyda Z. Rojas, Aurélie Pahud de Mortanges, Eric Francis Macharia-Nimietz, Fadi Alijla, Beatrice Minder, Alexander B. Leichtle, Nora Lüthi, Simone Ehrhard, Yok-Ai Que, Laurenz Kopp Fernandes, Wolf Hautz, Taulant Muka

**Affiliations:** 1 Institute of Social and Preventive Medicine, University of Bern, Bern, Switzerland; 2 Department of Molecular Epidemiology, German Institute of Human Nutrition Potsdam-Rehbrücke, Nuthetal, Germany; 3 ELKH-DE Public Health Research Group of the Hungarian Academy of Sciences, Department of Public Health and Epidemiology, Faculty of Medicine, University of Debrecen, Debrecen, Hungary; 4 Department of Epidemiology, Erasmus MC University Medical Center, Rotterdam, The Netherlands; 5 Department of Pediatrics, “Mother Teresa” University Hospital Center, Tirana, University of Medicine, Tirana, Albania; 6 HRB-Trials Methodology Research Network College of Medicine, Nursing and Health Sciences University of Galway, Galway, Ireland; 7 Graduate School for Health Sciences, University of Bern, Bern, Switzerland; 8 Department of Community Medicine, Tehran University of Medical Sciences, Tehran, Iran; 9 Department of Preventive Medicine and Public Health, University of Navarre, Pamplona, Spain; 10 Navarra Institute for Health Research, IdiSNA, Pamplona, Spain; 11 Research Group and Development of Nursing Knowledge (GIDCEN-FCV), Research Center, Cardiovascular Foundation of Colombia, Floridablanca, Santander, Colombia; 12 Faculty of Medicine, University of Bern, Bern, Switzerland; 13 Thoracic Surgery Department, University Hospital Basel, University of Basel, Basel, Switzerland; 14 Public Health and Primary Care Library, University Library of Bern, University of Bern, Bern, Switzerland; 15 University Institute of Clinical Chemistry, Inselspital, Bern University Hospital, and Center for Artificial Intelligence in Medicine (CAIM), University of Bern, Bern, Switzerland; 16 Emergency Department, Inselspital, Bern University Hospital, University of Bern, Freiburgstrasse 16C, 3010 Bern, Switzerland; 17 Department of Intensive Care Medicine, Inselspital, Bern University Hospital, University of Bern, Bern, Switzerland; 18 Deutsches Herzzentrum Berlin (DHZB), Berlin, Germany; 19 Charité Universitätsmedizin Berlin, Berlin, Germany; 20 Swiss Paraplegic Research, Nottwil, Switzerland; 21 Epistudia, Bern, Switzerland; 22 Department of Diabetes, Endocrinology, Nutritional Medicine and Metabolism, Inselspital, Bern University Hospital, University of Bern, Bern, Switzerland; 23 Division of Endocrinology and Diabetology, Department of Internal Medicine, Medical University of Graz, Graz, Austria; 24 Julius Center for Health Sciences and Primary Care, University Medical Center Utrecht, Utrecht University, Utrecht, The Netherlands; 25 German Center for Diabetes Research (DZD), München-Neuherberg, Germany; 26 Faculty of Health Sciences, University of Lucerne, Lucerne, Switzerland

**Keywords:** COVID-19, Prediction models, Mortality, ICU, Systematic review, Biomarkers

## Abstract

Current evidence on COVID-19 prognostic models is inconsistent and clinical applicability remains controversial. We performed a systematic review to summarize and critically appraise the available studies that have developed, assessed and/or validated prognostic models of COVID-19 predicting health outcomes. We searched six bibliographic databases to identify published articles that investigated univariable and multivariable prognostic models predicting adverse outcomes in adult COVID-19 patients, including intensive care unit (ICU) admission, intubation, high-flow nasal therapy (HFNT), extracorporeal membrane oxygenation (ECMO) and mortality. We identified and assessed 314 eligible articles from more than 40 countries, with 152 of these studies presenting mortality, 66 progression to severe or critical illness, 35 mortality and ICU admission combined, 17 ICU admission only, while the remaining 44 studies reported prediction models for mechanical ventilation (MV) or a combination of multiple outcomes. The sample size of included studies varied from 11 to 7,704,171 participants, with a mean age ranging from 18 to 93 years. There were 353 prognostic models investigated, with area under the curve (AUC) ranging from 0.44 to 0.99. A great proportion of studies (61.5%, 193 out of 314) performed internal or external validation or replication. In 312 (99.4%) studies, prognostic models were reported to be at high risk of bias due to uncertainties and challenges surrounding methodological rigor, sampling, handling of missing data, failure to deal with overfitting and heterogeneous definitions of COVID-19 and severity outcomes. While several clinical prognostic models for COVID-19 have been described in the literature, they are limited in generalizability and/or applicability due to deficiencies in addressing fundamental statistical and methodological concerns. Future large, multi-centric and well-designed prognostic prospective studies are needed to clarify remaining uncertainties.

**Supplementary Information:**

The online version contains supplementary material available at 10.1007/s10654-023-00973-x.

## Introduction

As of December 2022, over 650 million cases of Corona Virus Disease 2019 (COVID-19), caused by the severe acute respiratory syndrome coronavirus 2 (SARS-CoV-2), have been confirmed and over 6.6 million deaths globally were reported to the World Health Organization (WHO). To date, despite of vaccination efforts and other public health measures, viral transmission and therefore evolution, is a persisting challenge, with the numbers of confirmed cases still on the rise [1]. The clinical picture of COVID-19 infection is heterogeneous and ranges from asymptomatic or pre-asymptomatic phase, mild or moderate respiratory symptoms to severe viral pneumonia and acute respiratory distress syndrome, septic shock and/or multiple organ dysfunction requiring admission to intensive care unit (ICU), which might eventually lead to need for mechanical ventilation (MV), intubation, extracorporeal membrane oxygenation (ECMO) and death [2–4]. Early identification of COVID-19 patients at risk of critical illness is crucial for early identification of patients requiring urgent medical attention or who would benefit the most from treatment. In addition, early prediction of the disease course not only enables cost-effective allocation of health care resources, but potentially decreases fatality rates as well [5, 6]. The supply and demand of emergency department (ED) and ICU beds has created an imbalance, due to the large number of individuals affected by COVID-19, hence straining the available health care resources [7].

Demographics, comorbidities, physical examinations, laboratory parameters and imaging predictors have been tested in several studies and used to develop prognostic models for COVID-19. These models have been used to evaluate disease prognosis and to perform triage in case of scarce resources in some clinical settings [7]. Laboratory indicators including, but not limited to, lymphocyte and platelet count, creatinine, interleukin 6 (IL-6), procalcitonin (PCT), d-dimer, ferritin, lactate dehydrogenase (LDH), C-reactive protein (CRP), aspartate aminotransferase (AST), alanine aminotransferase (ALT), high-sensitivity troponin T (hs-TnT), albumin and creatine kinase (CK), have been identified as common predictors of poor outcomes in COVID-19 [8]. However, most of the reported models are at high risk of bias due to deficiencies in the methods used, definitions of COVID-19 (e.g., cases defined based on clinical features rather than on the result of laboratory diagnostic test for SARS-CoV-2) and the use of heterogeneous outcomes. Thus, there is an urgent need to comprehensively and critically assess the available literature and identify the best performing and methodologically rigorous prognostic models for COVID-19 progression.

With that in mind, we performed this systematic review to summarize and critically appraise the available studies that have developed, assessed and/or validated clinical prognostic models for COVID-19 to predict progression to severe or critical disease, ICU admission, need for MV, intubation, HFNT and mortality.

## Methods

### Data sources and search strategy

We conducted our systematic review following a recently published guide on performing systematic reviews and meta-analyses [9] and report based on the Preferred Reporting Items for Systematic Review and Meta-analyses (PRISMA) recommendations [10]. The protocol was registered with the international prospective register of systematic reviews (PROSPERO) with ID: CRD42021257478. We searched 6 electronic databases: Embase Ovid, Medline Ovid, Cochrane Central, Web of Science Core Collection, WHO COVID-19 Global literature on coronavirus disease and Google Scholar from inception to June 20th, 2022. To identify relevant records, we combined (a) COVID-19 related terms with (b) prognostic model-related terms, such as “risk prediction models”, “biomarkers”, and (c) outcome type: mortality, ICU admission, intubation, HFNT and ECMO. We performed our search with the assistance of an experienced medical librarian. We used EndNote to manage references. Details on the search strategy are provided in the *Supplemental material: Appendix A*.

### Study selection

We used the following criteria in selecting studies for inclusion: (a) conducted in adult humans diagnosed with SARS-CoV-2 infection and/or COVID-19; (b) analyzed data from a prospective or retrospective cohort; (c) used a prognostic model to predict one or more adverse outcomes of COVID-19; (d) reported on the model’s predictive performance; and (e) published in a peer-reviewed journal. There was no restriction on publication year or language. We excluded case-reports, case–control and cross-sectional studies, dissertation abstracts, letters to the editor, conference proceedings, systematic reviews or meta-analyses, books, book chapters and animal studies. Two reviewers independently evaluated abstracts and full texts of the studies. Discrepancies between reviewers were resolved through a consensus or in consultation with a third independent reviewer.

### Data extraction

Data from included studies were extracted independently in duplicate, based on various domains including the number of participants, predictors, outcomes, data analysis details and performance of the prediction model (including, but not limited to, AUC/c-index, sensitivity, specificity, positive and negative predictive values, likelihood ratio, accuracy, etc.). The extracted data were cross-checked by two reviewers and the complete data extraction form can be found in the *Supplemental material, Tables 3 and 4*.

### Quality assessment

We used the PROBAST checklist to evaluate potential sources of bias and issues of individual studies that may affect the applicability of results in relation to the intended use of models. PROBAST includes 20 signaling questions across 4 domains (i.e., participants, predictors, outcome, and analysis). Two reviewers conducted this assessment independently. Any disagreements were handled by discussion and consensus. Following the PROBAST standards, if a prediction model evaluation was judged at low risk on all domains relating to bias and applicability, then the overall judgment was assessed as “low risk of bias (RoB)” or “low concern regarding applicability”. If an evaluation was judged as high RoB for at least 1 domain, it was considered as “high RoB” or “high concern regarding applicability”. If the prediction model evaluation was unclear in 1 or more domains and was rated as low in the remaining domains, it was judged as having “unclear RoB” or “unclear concern regarding applicability” [11]. Key information was organized by relevant domains in the *Supplemental material, Table 5.*

### Data synthesis

For each study, we reported measures quantifying the performance of the prediction model and 95% confidence intervals (CI), when provided. Heterogeneity permitting, we sought to pool the results using either a fixed or random effects meta-analysis model to provide the pooled estimates and the corresponding 95% CIs.

## Results

### Literature search

**Fig. 1:**
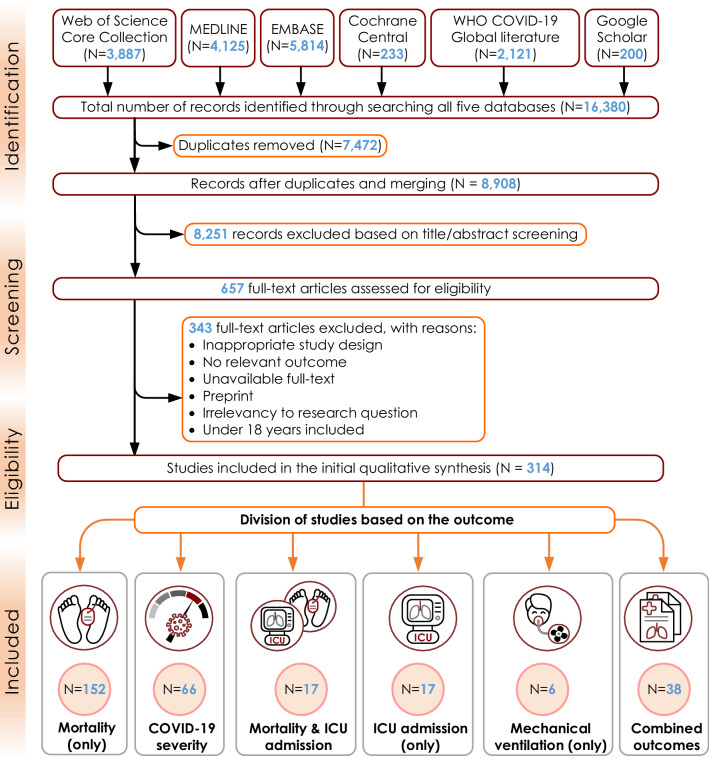
Flowchart of inclusions and exclusions adapted from PRISMA

After excluding duplicates, 8,908 unique references were identified. Based on the initial screening of titles and abstracts, the full texts of 657 articles were retrieved and further evaluated. After full-text assessment, 343 studies were excluded due to inappropriate study design, no relevant outcome, unavailable full-text, pre-prints, irrelevance to research question, model performance not reported, or studies including human participants under 18 years. After examining full-texts, we identified 314 eligible articles, of which 152 (48.4%) presented mortality as the outcome [4, 5, 12–160], 66 (21.0%) concentrated on severity and/or critical illness [2, 161–225], 35 (11.1%) presented ICU admission and mortality combined [7, 226–259], 17 (5.4%) assessed ICU admission only [225, 260–275], 6 (1.9%) looked at mechanical ventilation only [276–281] and 38 (12.1%) assessed multiple combined outcomes [195, 282–318]. We present the results in the PRISMA flow chart Fig. 1. A meta-analysis was not feasible because of the large heterogeneity of study designs, measurement techniques, methods of analysis and handling of continuous variables, inclusion criteria, as well as reporting of different outcome measures.

### Characteristics of the included studies

Of the 314 studies meeting eligibility criteria, 111 (35.4%) were conducted in Asia, 113 (36.0%) in Europe, 52 (16.6%) in North and Central America, 16 (5.1%) in South America, 7 (2.2%) in Africa, 1 (0.3%) in Australia and 14 (4.5%) were multi-national studies. The sample size ranged from 11 to 7,704,111 participants. Two hundred and seventy-five studies (87.6%) were based on retrospective study design, 36 (11.5%) on prospective study design and 3 (0.9%) on ambispective study design. The mean age ranged from 18 to 93 years with percentage of male participants ranging between 31.8–100%. Fifty-seven studies explored only model development, 26 performed only model validation, 167 developed and validated models internally and/or externally and 64 studies either assessed, analyzed, identified or compared prediction biomarkers.

The research population in the studies covered adult population (> 18 years old) presenting in the emergency room with clinical suspicion of COVID-19 or patients who were admitted to the hospital with laboratory confirmed SARS-CoV-2 infection by polymerase chain reaction (PCR) testing of nasopharyngeal samples. In 6 studies, the diagnosis was based on COVID-19 symptoms only. Of the 314 studies, 271 focused on hospitalized patients at baseline, 37 on hospitalized patients in the ICU, 5 focused on non-hospitalized patients and one study included both hospitalized patients in the ICU and non-hospitalized.

Out of 314 studies, 55 (17.5%) of the total studies adhered to transparent reporting of a multivariable prediction model for individual prognosis or diagnosis (TRIPOD) model evaluation guidelines. Across studies, multivariate logistic regression analysis was the most frequently used method of model development, followed by logistic regression, univariate logistic regression and machine learning analysis. We present the general characteristics of the eligible articles in the *Supplementary material, Table 1.*

### Models predicting mortality

The most common outcome was mortality which was evaluated in 152 (48.4%) studies [4, 5, 12–160]. The population sample size ranged from 11 to 6,952,440 participants. The mean age ranged from 18 to 93 years, and the percentage of male participants from 31.8% to 100%. Among studies that specified and reported prediction time for mortality there was variation in reporting. In only 4 studies, prediction time was defined in a fixed time frame, i.e., within the time interval 8 to 30 days. The range of prediction time in the latter studies was between 24 h to 90 days. However, in some studies, prediction time was defined as time-to-event (i.e., death). The difference between fixed time frame and time-to-event outcomes, lies mainly in the fact that time-to-event reporting takes account of whether an event takes place and the time at which the event occurs, such that both the event and the timing of the event are important. In addition, it shows the differences duration of survival. Fixed timeframe reporting is commonly used when outcome measure data are collected for each participant and does not correspond to the overall duration of the study, but to a specified timeframe within which the outcome is most expected. By ‘prediction time’ we mean the timeframe which the model prespecified to assess its performance. Predictors encountered most frequently in the 152 studies that developed or validated mortality prediction models were increased age, sex, decreased oxygen saturation, elevated levels of CRP, blood urea nitrogen, body temperature, number of comorbidities, unconsciousness, white blood cells count, lymphocyte count, D-dimer level, platelets and pulse rate. Model performances assessed with AUC/ROC or c‐index were reported in 133 studies and ranged between 0.49 to 0.99. Additionally, sensitivity and specificity were reported in 73 studies ranging from 15.4 to 100% and 10.9 to 98.7%, respectively. Thirteen (8.6%) of the 152 included studies focused solely on model validation, whereas 83 (54.6%) combined development with internal and/or external validation. The best reported predictive performance belonged to a model developed in Boston, USA, and externally validated in Wuhan, China, with 375 participants. The development cohort, internal validation, and external validation’s respective AUC scores were 0.987, 0.999, and 0.992, respectively. The model was based on information acquired on admission, including age, lymphocyte count, d-dimer, CRP and creatinine (ALDCC) [123]. We present the detailed characteristics of the eligible articles based on mortality as outcome in the *Supplementary material Table 2.*

### Models predicting severity or critical illness

Severity or critical illness in COVID-19 was reported in 66 (21.0%) [2, 161–225]. The definition of severity or critical outcome in COVID-19 varied across the studies. Some studies used the standard definition, which grades COVID-19 severity using the following criteria: (1) shortness of breath: respiratory rate > 30 breaths/min in the resting state; (2) pulse oxygen saturation < 93% or (3) arterial blood oxygen pressure (PaO_2_)/oxygen concentration (FiO_2_) < 300 mmHg. Criteria for critical patients includes: (1) respiratory failure requiring MV; (2) shock; (3) other multi-organ failure requiring ICU monitoring treatment. While other studies defined severity or critical illness as the occurrence of any of the following events: ICU admission, need for invasive mechanical ventilation (IMV) or death. We present the detailed definitions of COVID-19 severity or critical illness in the *Supplemental material Table 6.* Sample size ranged from 55 to 7,704,111 participants. The mean age ranged from 38.2 to 87.0 years with percentage male ranging from 41.4% to 77%. Prediction time defined as timeframe which the model pre-specified to assess its performance, i.e., from admission to the worsening severity or critical illness was different across studies, and the longest time of follow-up was 30 days. The most frequently encountered predictors were older age, sex, body temperature, number of comorbidities (cardiovascular disease (CVD), hypertension, diabetes), decreased oxygen saturation, elevated levels of CRP, blood urea nitrogen (BUN), body temperature, systolic blood pressure (SBP), neutrophil-to-lymphocyte ratio (NLR), white blood cells count (WBC), lymphocyte count and pulse rate. Model performances were assessed with AUC ranging from 0.57 to 0.99 in 60 studies, and sensitivity and specificity ranging from 7.1 to 100% and 19.5% − 100%, respectively. Thirty-nine (59.1%) of the 66 studies that were considered, combined development with internal and/or external validation, whereas 3 (4.5%) of the studies only focused on model validation. A combined machine learning model (Support Vector Machine (SVM), Gradient Boosted Decision Tree (GBDT), and Neural Network(NN)) based on procalcitonin, [T + B + NK cell] count, IL-6, CRP, IL-2-receptor, T-helper lymphocyte/T-suppressor lymphocyte as predictors of critical illness had the best reported predictive performance, with an AUC of 0.99. This model was developed in China with 450 participants (NN) [174]. We present detailed characteristics in the *supplemental material Table 4.*

### Models predicting mortality and ICU admission

Thirty-five (11.1%) out of 314 studies reported mortality and ICU admission as outcome [7, 226–259]. Sample size in these studies ranged from 53 to 5,831 participants. The mean age differed from 43 to 82.2 years with percentage male ranging from 46.8% to 70.3%. Prediction time or the time from admission to ICU admission and death ranged from 24 h to 30 days. Common predictors were age, gender, pulse rate, albumin, WBC count, procalcitonin, LDH, CRP, ferritin, BUN, comorbidities and oxygen saturation among others. The AUC ranged from 0.63 to 0.98 in 32 studies, and sensitivity and specificity in 19 studies ranging from 10.5–98.7% from 41–100%, respectively. Eleven (31.4%) studies combined development with internal and/or external validation, whereas 4 (11.4%) of the studies only focused on model validation. Among these studies, the biomarker with the best predictive performance was CRP, showing an AUC of 0.975 [234]*.* We present detailed characteristics of the studies in the *Supplemental material Table 4.*

### Models predicting ICU admission only

In total, there were only 17 (5.4%) studies which reported ICU admission as their outcome [225, 260–275]. Sample size in these studies varied from 67 to 4663 participants with a mean age ranging from 40 to 71.4 years and percentage male between 39.5% and 75.8%. Predictors reported were patients’ age, sex, presence of hypertension and diabetes, fever, short-ness of breath, serum glucose, AST, respiratory rate, NLR ratio, LDH, systolic blood pressure, CRP and fibrinogen. Model performances were assessed with AUC ranging from 0.44 to 0.97 in 15 studies, and sensitivity and specificity in 11 studies ranging from 30.2% to 92.4% and 45.5% to 99.7%, respectively.

Ten out of 17 studies (58.8%) provided combined development with internal and/or external validation, with the best reported predictive performance belonging to a model developed in Greece with 67 participants and an AUC of 0.97 based on patients’ gender, presence of hypertension and diabetes, fever, shortness of breath, serum glucose, AST, LDH, CRP and fibrinogen [261].We present detailed characteristics of the studies in the *supplemental material Table 4.*

### Models predicting combined outcomes

Six (1.9%) of 314 studies reported models predicted MV only [195, 282–318], and 38 (12.1%) reported on a combination of outcomes (intubation, HFNT, ECMO, ICU admission and mortality) [195, 282–318]. Prediction time defined as timeframe which the model pre-specified to assess its performance, i.e., from admission to event outcomes, was different across studies, and the longest time of follow-up here was 30 days. AUC assessed in all 44 studies ranged from 0.53 to 0.94, and sensitivity and specificity ranging from 21.5% to 98.6% and 13.7% to 89.2%, respectively. A combination of model development with internal and/or external validation was provided by 24 (54.5%) out of 44 studies with an AUC of 0.94 for the two models with the best predictive performance [294, 316]*.* Additional information on prediction time, model performance and predictors are presented in * Supplementary material Table 4*.

### Risk assessment

In the participants domain, 31/314 (9.9%) studies were rated as being at low RoB, 15/314 (4.8%) as unclear and 268/314 (85.4%) were at high RoB. High RoB in the participants domain mainly resulted from retrospective study design, in which data were generated from existing sources such as existing cohort studies or routine care registries.

In the predictors domain, 40/314 (13.1%) studies were rated as being at low RoB, 118/314 (37.6%) as unclear and 156/119 (49.7%) were at high RoB. The 40 studies with low RoB were rated as such given that the predictor assessment was conducted without prior knowledge of the outcome. However, most of the studies did not report information on predictor blinding, hence high or unclear RoB.

In the outcome domain, 53/314 (16.9%) studies were rated as being at low RoB, 157/314 (50.0%) as unclear and 104/314 (33.1%) as being at high RoB. High or unclear RoB was mainly due to the outcome being determined with prior knowledge of predictor information, leading to biased predictive performance. Another reason was lack of information on the time interval between predictor assessment and outcome determination in most of the studies.

In the analysis domain, 4/314 (1.3%) studies were rated as being at low RoB, 30/314 (9.6%) as unclear and 280/314 (89.2%%) as high RoB. Some of the reasons leading to studies being rated at high RoB were: (i) unreasonable number of participants with the outcome; (ii) inappropriate method of handling missing data; (iii) predictors selected based on univariable analysis prior to multivariable modelling and (iv) calibration and discrimination were not evaluated and no internal validation preformed.

Inappropriate method of handling missing data is related to the sampling, as a statistically valid analysis for a prognostic model which has appropriate mechanisms and assumptions for the missing data should be conducted to consider it at low risk of bias. The ‘appropriateness’ of handling missing values depends on the type of missing data, as well as the assumptions based on the reasons for the missing data. For instance, if the data that are missing are missing completely at random, the analysis is likely to be unbiased. Power may be lost in the design, but the estimated parameters are not biased by the absence of the data. However, if data are not missing at random, this might be problematic and there are specific methods that should be used to handle them. For instance, one way to obtain an unbiased estimate of the parameters in such a case is to model the missing data. The model may then be incorporated into a more complex one for estimating the missing values. Another way is to conduct a sensitivity analysis, or apply techniques like listwise or case deletion, pairwise deletion, mean substitution, regression imputation, last observation carried forward, maximum likelihood, expectation–maximization, multiple imputation, etc. Knight et al. (2022) [304] used multiple imputation to deal with missing data and examined heterogeneity in detail by NHS region, ethnicity and month of admission, and could serve as low RoB example.

Overall, the RoB assessment was rated to be at high or unclear RoB in 312 studies. This could be explained by shortcomings such as poor methodological quality, small sample size, poor handling of missing data, failure to deal with overfitting, definitions of COVID-19 based on clinical features rather than on the result of laboratory diagnostic test for SARSCoV-2 and its severity with studies using heterogeneous outcomes. Only two studies [82, 304], were considered to have a high level of methodological rigor with overall low RoB in all the domains. Knight et al., developed and validated the 4C Mortality score, which was rated as of good quality. This model includes 8 variables namely age, sex, number of comorbidities, respiratory rate, peripheral oxygen saturation, level of consciousness, urea level and CRP. It showed high discrimination for mortality with AUC of 0.77, (95% CI 0.76—0.77) in the validation cohort. The same 4C prognostic model was further validated in a large prospective cohort in UK and it showed AUCs of 0.78 (95% CI 0.77—0.78) and 0.76 (95% CI 0.75—0.77) for 4Cmortality and 4C Deterioration scores respectively.

According to the validation study, both the 4C Mortality and the 4C Deterioration scores can be used to stratify patients who have been admitted to the hospital with confirmed COVID-19. Both scores can also be used to make treatment decisions [82, 304].

### Data availability

Only 14 (4.5%) of the 314 studies reviewed indicated the availability of data by either providing a link or where the data could be found. In a hundred and six (33.8%) studies, the authors stated that data are available upon request. The majority of the studies did not mention of the availability of the data.

## Discussion

**Fig. 2:**
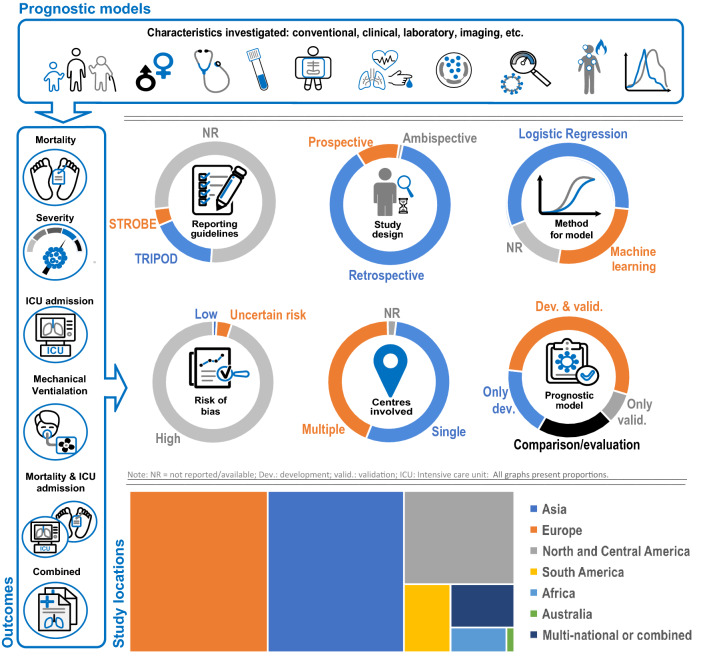
Visual summary of the findings on evaluated prognostic models

Fig. 2 To our knowledge, this is the most comprehensive systematic review of developed and/or validated clinical prognostic models for COVID-19. Our results indicate that only one predictive model was considered to be at low RoB, while the rest of the models were developed from studies suffering from many methodological issues including limitations in model development, presentation and being incompletely or inadequately reported. Thus, the polarized focus on limitations and methodological challenges that emerged from this report serves as a reminder that current prognostic models on COVID-19 severity, have limited applicability and require caution before implementation in routine care in the clinical setting. In addition, models were concentrated in specific regions, as they were developed and validated predominantly in China and Europe. Hence, there is a need to develop models tailored to other countries before generalization and application.

Contrary to the review done by Wynants et.al [319] which primarily focused on models for diagnosis and prognosis of COVID-19, our work exclusively focused on studies that either developed or validated models predicting COVID-19 severity and mortality. We included 314 studies that investigated either univariable or multivariable prognostic models that predict COVID-19 adverse outcomes, whereas Wynants et al. analyzed only multivariable related models and scoring systems. Thus, the studies included by Wynants et al. were limited in understanding single biomarkers that might have clinical utility in predicting COVID-19 severity. Additionally, in our research, all reviewed articles were peer-reviewed and published articles, omitting pre-prints for which there is also no guarantee that the information provided is supported by the data due to lack of formal peer review.

Eighty seven percent of the studies in this review are based on retrospective databases leading to lack of consistency in predictor and outcome measurement and deficiencies related to missing data. Of the included studies, only 36 studies were of prospective design. This indicates a high potential of model development and validation studies being appraised as being at high RoB, because the existing data sources, registries or existing cohorts were used. Data in the registries are usually collected for many purposes other than development of the prognostic models [320]. Thus, future studies using standardized and repeated measurements and prospective design can provide better answer to which extend prediction models can accurately predict COVID-19 severity.

In our review, only 55 (17.5%) studies adhered to the TRIPOD reporting guidelines. Similar to ours, previous reviews have shown that the quality of reporting in most of the articles describing model development or validation is relatively poor, and in the absence of detailed and transparent reporting of key study details, it is difficult for researchers to objectively judge the strengths and weaknesses of a prediction model study [321]. TRIPOD provides good reporting of the studies developing or validating prognostic models, thereby enabling the strength and weaknesses of a study to be revealed, hence facilitating its interpretation, and making it usable [11, 319].

Univariable and multivariable logistic regression analysis was the most used method of the prognostic model development in our review. Logistic regression is a widely used statistical method that allows for multivariable analysis and modelling of a binary dependent variable [322]. Other than logistic regression methods, machine learning methods such as decision tree (DT), gradient boosting decision trees (GB), support vector machine (SVM) and neural network (NN) were also applied to develop prediction models. Machine and artificial learning are becoming more common techniques due to increasing availability of large datasets. However, models developed based on machine learning and artificial intelligence must be carefully developed to reduce the risk of overfitting when data are sparse [11].

The overall sample size of the eligible studies in our review was ranging from 11 to 7,704,111 participants. However, by considering the commonly used *rules of thumb* to determine the sample size for prognostic models, it is evident that the sample sizes of some of the included studies in the review were relatively low. The number of EPV is the number of events divided by the number of predictor variables considered in developing the prediction model [323]. The rule of thumb recommends observation of at least 10 events per variable (EPV) and is based on estimates of stability of coefficient estimates for individual variables [324]. Future studies need to fulfil this criterion before seeking to validate these models externally and eventually predicting disease severity in a clinical scenario.

Our findings indicate that most studies do not provide information on availability of data, and among those studies providing information, only a minority provides information where the data can be found and accessed. Data availability and sharing is one of the cornerstones of quality science that allows reanalysis of data, reproducibility and merging of different dataset to address small sample size and other methodological issues. Thus, more work should be done in the future to address this issue, and facilitate access and exchange of data.

While we made a comprehensive search strategy, we do not exclude possibility of missing other relevant articles that reported regression results in abstract but the prognostic ability results only in full text. However, these studies very likely did not have as primary goal the generation or validation of a prognostic model, which was the primary interest of this review.

A future consideration for prognostic modelling studies should be the determination of the variant of the virus circulating, as certain variants, may be more severe than others, and may influence severity, adverse outcomes and death. For instance, in 2021, the Delta variant was identified and appeared to be more transmissible than the ancestral strain and also more severe. A study from the UK (*n* = 43,000 cases) showed that patients infected with Delta had twice the risk of hospitalization compared to those infected with Alpha, despite overall being younger [325].

### Implications and recommendations

Future studies or researchers are recommended to use prospective longitudinal cohort design for prognostic model development and validation where the methods are prespecified and consistent. By adhering to consistent methods, participants data are systematically and validly recorded [11]. Another important aspect of prediction models is the internal and/or external validation process, which was done in 61.5% of studies included in this review. Future studies should therefore consider validating or comparing the existing models in different settings. To avoid models with biased selection of variables and inaccurate predictions, future studies are recommended to use large sample sizes or to report their justification for the choice of sample size [324]. Quality can be further improved by properly addressing the non-linear prognostic factors and missing values in studies. Authors are advised to follow the TRIPOD guidelines at the same time as they develop their predictive models in order to reduce their risk of bias [11, 321]. Considering that most included studies were found to be at high risk of bias, it is essential that future studies provide new and robust insight into the topic. Several prognostic models have been developed, but none is clearly superior nor accurately predicts deterioration or mortality to a great degree [326, 327]. The speed of symptom progression is not an accurate predictor of worse outcomes [328] and pre-intubation sequential organ assessment score has been shown to perform poorly as a predictor of death in patients with COVID-19 [329].

Additionally, researchers should consider well-designed prospective studies and biomarkers that could be easily assessed, readily available and are inexpensive to measure, to improve the clinical accessibility and applicability of the resulting models.

Finally, there is a need to build capacity and infrastructure to carry out research on prognostic tools that are potentially of benefit when applied in a clinical setting. Assessment of impact on decision-making should be critical when awarding grants for the development of predictive tools for quality improvement.

### Example of good methods and reporting

Despite the general poor assessment on the quality of the articles, there were 2 articles that were appraised as low risk of bias, both by Knight et al. These articles used appropriate method and adhered to adequate reporting guidelines [82, 304].

The author included: (a) appropriate study design for prognostic models; (b) sufficient sample size to allow model development (event per variable (EPV) values of over 40); (c) reporting of the model method and coefficients of the final model. The 4C Mortality Score was found to have excellent discrimination and calibration in the validation cohort. It showed good applicability within the validation cohort and consistency across all performance measures. The score can accurately characterize the patients of high risk of death in hospital since it uses commonly available clinical observations, blood parameters and demographics at the time of hospital admission.

## Conclusion

In this review, models predicting COVID-19 severity used comparable predictors, with different prediction performance. However, due to concerns in resolving statistical and methodological difficulties, the evidence is of relatively poor methodological rigor with limited generalizability and applicability. Future large, multi-center and well-designed prospective studies are needed for the development of predictive models for COVID-19 with clinical utility that can be applied to diverse populations. This requires a homogeneous definition of COVID-19 and outcomes and appropriate model selection methods to lead user-friendly models that can be externally validated.

## Supplementary Information

Below is the link to the electronic supplementary material.

Supplementary file1 (DOCX 23 KB)

Supplementary file2 (DOCX 52 KB)

Supplementary file3 (DOCX 123 KB)

Supplementary file4 (DOCX 228 KB)

Supplementary file5 (DOCX 246 KB)

Supplementary file6 (DOCX 35 KB)

## Data Availability

Data sharing is not applicable to this article as no datasets were generated or analyzed during the current study.
