# Supplementary material for: Prognostic models in COVID-19 infection that predict severity: a systematic review
Source: Eur J Epidemiol. 2023 Feb 25;38(4):355–72. doi: 10.1007/s10654-023-00973-x (PMC9958330; doi:10.1007/s10654-023-00973-x)
Supplement: Supplementary file 1 — Supplementary file1 (DOCX 23 KB) [file 10654_2023_973_MOESM1_ESM.docx]

**Supplemental material: Appendix A** Detailed search strategies used in this review

**Overview databases & results**

Date last searched June 20^th^, 2022

**Medline ALL (Ovid)**Ovid MEDLINE(R) ALL <1946 to June 17, 2022>

1 (exp Coronavirus/ or exp Coronavirus Infections/ or (coronavir* or corona virus* or HCoV* or ncov* or cov2 or cov-2 or 2019nCoV or "novel CoV" or covid*2 or covid-19 or covid19 or sars-cov* or sarscov* or sars-coronavirus* or Severe Acute Respiratory Syndrome Coronavirus*).mp.) and ((20191* or 202*).dp. or 20190101:20301231.(ep).) 270003

2 ((Prognosis/ or exp Risk Assessment/) and (exp Models, Theoretical/ or exp Machine Learning/ or algorithms/ or Medical Informatics/)) or Early Warning Score/ or Forecasting/ or (((predict* or prognos* or learning) and (model* or scor* or biomarker* or marker* or variable* or outcome*)) or (early adj2 (warning* or identification*)) or forecast* or ((machin* or deep or transfer) adj3 learning*) or algorithm* or software* or "clinical informatic*").ti,ab,kf. 2082872

3 "sensitivity and specificity"/ or "predictive value of tests"/ or roc curve/ or area under curve/ or odds ratio/ or proportional hazards models/ or (accurac* or validat* or ((risk or predict* or cox or hazard) adj5 (model* or abilit* or analys* or stratif*)) or sensitivity or specificity or (predic* adj1 value*) or ppv or npv or (diagnos* adj1 value*) or likelihood ratio* or c-statistic* or c-index or multivaria* or AUC or AUROC or ((area-under or ROC) adj3 curve) or ((odd* or risk) adj3 ratio*)).ab,ti. 3571891

4 exp Intensive Care Units/ or Emergency Service, Hospital/ or Critical Care/ or Intubation/ or Intubation, Intratracheal/ or Extracorporeal Membrane Oxygenation/ or exp Respiration, Artificial/ or exp Mortality/ or mortality.fs. or exp survival analysis/ or (ICU or ICUs or ((intensive or critical) adj1 care) or intubat* or ventilat* or hospital* or (critical* adj (ill or illness*)) or "high-flow nasal" or "highflow nasal" or "nasal high flow" or HFNC or HFNCT or "Extracorporeal Membrane Oxygenat*" or "Extra-corporeal Membrane Oxygenat*" or ECMO or ECLS or mortalit* or death* or died or fatalit* or surviv*).ti,ab,kf. 4508654

5 exp cohort studies/ or follow-up studies/ or exp longitudinal studies/ or prospective studies/ or retrospective studies/ or observational study/ or exp clinical trial/ or multicenter studies as topic/ or validation study/ or (cohort* or case-cohort* or (nested case* adj3 control*) or prospectiv* or retrospectiv* or longitudinal or follow-up or followup or followed-up or ((observ* or populat* or intervent* or clinical or validation) adj2 (study or studies or trial*)) or (randomi?ed adj7 trial*) or (controlled adj3 trial*)).ab,ti. 5117794

6 1 and 2 and 3 and 4 and 5 4236

7 6 not (exp animals/ not humans/) not (letter or news or comment or editorial or congress or abstracts).pt. not ((exp infant/ or exp child/ or adolescent/) not exp adult/) not (child* or infan* or pediatric* or paediatric*).ti. 4125

**Embase (Ovid)**

Embase <1974 to 2022 June 17>

1 (coronavirus disease 2019/ or experimental coronavirus disease 2019/ or exp SARS-related coronavirus/ or severe acute respiratory syndrome/ or anti-SARS-CoV-2 agent/ or exp SARS-CoV-2 vaccine/ or exp covid-19 testing/ or (coronavir* or corona virus* or HCoV* or nCoV* or 2019 CoV or 2019nCoV* or COVID or covid19 or sars-cov* or sarscov* or sars-coronavirus* or sarscoronavir* or Severe Acute Respiratory Syndrome Coronavirus*).mp.) and 20191101:20301231.(dc). 302262

2 ((exp prognosis/ or prediction/ or exp risk assessment/) and (theoretical model/ or exp model/ or exp machine learning/ or software/ or exp algorithm/ or exp medical informatics/)) or disease severity assessment/ or prognostic assessment/ or early warning score/ or computer prediction/ or forecasting/ or scoring system/ or prognostic model/ or predictive model/ or (((predict* or prognos* or learning) and (model* or scor* or biomarker* or marker* or variable* or outcome*)) or (early adj2 (warning* or identification*)) or forecast* or ((machin* or deep or transfer) adj3 learning*) or algorithm* or software* or "clinical informatic*").ti,ab,kw. 3112102

3 "sensitivity and specificity"/ or "predictive value"/ or predictor variable/ or prediction/ or accuracy/ or diagnostic accuracy/ or diagnostic test accuracy study/ or exp diagnostic value/ or receiver operating characteristic/ or exp area under the curve/ or odds ratio/ or proportional hazards model/ or (accurac* or validat* or ((risk or predict* or cox or hazard) adj5 (model* or abilit* or analys* or stratif*)) or sensitivity or specificity or (predic* adj1 value*) or ppv or npv or (diagnos* adj1 value*) or likelihood ratio* or c-statistic* or c-index or multivaria* or AUC or AUROC or ((area-under or ROC) adj3 curve) or ((odd* or risk) adj3 ratio*)).ab,ti. 5124458

4 exp intensive care unit/ or exp emergency health service/ or emergency ward/ or intensive care/ or intubation/ or exp respiratory tract intubation/ or exp extracorporeal oxygenation/ or exp artificial ventilation/ or critically ill patient/ or exp mortality/ or "cause of death"/ or mortality risk/ or exp survival/ or survival analysis/ or (ICU or ICUs or ((intensive or critical) adj1 care) or intubat* or ventilat* or hospital* or (critical* adj (ill or illness*)) or "high-flow nasal" or "highflow nasal" or "nasal high flow" or HFNC or HFNCT or "Extracorporeal Membrane Oxygenat*" or "Extra-corporeal Membrane Oxygenat*" or ECMO or ECLS or mortalit* or death* or died or fatalit* or surviv*).ti,ab,kw. 6591342

5 "cohort analysis"/ or follow-up/ or exp longitudinal study/ or prospective study/ or retrospective study/ or observational study/ or intervention study/ or clinical study/ or controlled study/ or exp clinical trial/ or major clinical study/ or multicenter study topic/ or validation study/ or (cohort* or case-cohort* or (nested case* adj3 control*) or prospectiv* or retrospectiv* or longitudinal or follow-up or followup or followed-up or ((observ* or populat* or intervent* or clinical or validation) adj2 (study or studies or trial*)) or (randomi?ed adj7 trial*) or (controlled adj3 trial*)).ab,ti. 14768205

6 1 and 2 and 3 and 4 and 5 8136

7 6 not ((exp animal/ or nonhuman/) not exp human/) not (letter or note or editorial or conference).pt. not (exp juvenile/ not exp adult/) not (child* or infan* or pediatric* or paediatric*).ti. 5814

**Cochrane Central Register of Controlled Trials**

#1 (coronavir* OR corona NEXT virus* OR HCoV* or nCov* or cov2 or cov-2 or 2019nCoV or "novel CoV" OR covid OR covid19 OR Severe-Acute-Respiratory-Syndrome-Coronavirus* OR sars-cov* OR SARSCoV* OR sarscoronavir*):ti,ab,kw 11292

#2 (((predict* or prognos* or learning) and (model* or scor* or biomarker* or marker* or variable* or outcome*)) or (early NEAR/2 (warning* or identification*)) or forecast* or ((machin* or deep or transfer) NEAR/3 learning*) or algorithm* or software* or clinical NEXT informatic*):ti,ab,kw 155197

#3 (accurac* or validat* or ((risk or predict* or cox or hazard) NEAR/5 (model* or abilit* or analys* or stratif*)) or sensitivity or specificity or (predic* NEAR/1 value*) or ppv or npv or (diagnos* NEAR/1 value*) or likelihood-ratio* or c-statistic* or c-index or multivaria* or AUC or AUROC or ((area-under or ROC) NEAR/3 curve) or ((odd* or risk) NEAR/3 ratio*)):ti,ab,kw 241672

#4 (ICU or ICUs or ((intensive or critical) NEAR/1 care) or intubat* or ventilat* or hospital* or (critical* NEXT (ill or illness*)) or "high-flow nasal" or "highflow nasal" or "nasal high flow" or HFNC or HFNCT or Extracorporeal NEXT Membrane NEXT Oxygenat* or Extra-corporeal NEXT Membrane NEXT Oxygenat* or ECMO or ECLS or mortalit* or death* or died or fatalit* or surviv*):ti,ab,kw 429056

#5 #1 AND #2 AND #3 AND #4 244 Trials

**Web of Science Core Collection**

#1 TS=(coronavir* OR corona virus* OR HCoV* or nCov* or cov2 or cov-2 or 2019nCoV or "novel CoV" OR covid OR covid19 OR Severe-Acute-Respiratory-Syndrome-Coronavirus* OR sars-cov* OR SARSCoV* OR SARScoronavirus*)

#2 TS=(((predict* or prognos* or learning) and (model* or scor* or biomarker* or marker* or variable* or outcome*)) or (early NEAR/2 (warning* or identification*)) or forecast* or ((machin* or deep or transfer) NEAR/3 learning*) or algorithm* or software* or "clinical informatic*")

#3 TS=(accurac* or validat* or ((risk or predict* or cox or hazard) NEAR/5 (model* or abilit* or analys* or stratif*)) or sensitivity or specifity or (predic* NEAR/1 value*) or ppv or npv or (diagnos* NEAR/1 value*) or likelihood-ratio* or c-statistic* or c-index or multivaria* or AUC or AUROC or ((area-under or ROC) NEAR/3 curve) or ((odd* or risk) NEAR/3 ratio*))

#4 TS=(ICU or ICUs or ((intensive or critical) NEAR/1 care) or intubat* or ventilat* or hospital* or (critical* NEAR/1 (ill or illness*)) or "high-flow nasal" or "highflow nasal" or "nasal high flow" or HFNC or HFNCT or "Extracorporeal Membrane Oxygenat*" or "Extra-corporeal Membrane Oxygenat*" or ECMO or ECLS or mortalit* or death* or died or fatalit* or surviv*)

#5 TS=(cohort* or case-cohort* or (nested-case* NEAR/2 control*) or prospectiv* or retrospectiv* or longitudinal or follow-up or followup or followed-up or ((observ* or populat* or intervent* or clinical or validation) NEAR/6 (study or studies or trial*)) or (randomi?ed NEAR/6 trial*) or (controlled NEAR/2 trial*))

#1 AND #2 AND #3 AND #4 AND #5

Refined by: PUBLICATION YEARS: (2022 OR 2021 OR 2020 OR 2019 ) = 3887 Results

**WHO COVID-19 Database: Global literature on coronavirus disease**The WHO Database of publications on coronavirus disease (COVID-19).
<https://www.who.int/emergencies/diseases/novel-coronavirus-2019/global-research-on-novel-coronavirus-2019-ncov>

(tw:(predict* OR prognos* OR learning)) AND (tw:(model* OR scor* OR biomarker* OR marker* OR variable* OR metrics OR outcome* OR forecast* OR algorithm* OR software OR informatic* OR machine))
AND
(tw:(icu OR icus OR "intensive care" OR "critical care" OR intubat* OR ventilat* OR hospital* OR "critical ill" OR "critical illness*" OR "high-flow nasal" OR "highflow nasal" OR "nasal high flow" OR hfnc OR hfnct OR "Extracorporeal Membrane Oxygenat*" OR "Extra-corporeal Membrane Oxygenat*" OR ecmo OR ecls OR mortalit* OR death* OR died OR fatalit* OR surviv*)) AND (tw:( accurac* OR validat* OR sensitivity OR specifity OR "predictive value*" OR ppv OR npv OR or likelihood-ratio* OR c-statistic* OR c-index OR multivaria* OR "area under the curve" OR auc OR auroc OR "risk ratio" OR "odds ratio"))
AND
db:("EuropePMC" OR "Scopus" OR "ICTRP" OR "ProQuest Central" OR "Academic Search Complete" OR "COVIDWHO" OR "ScienceDirect" OR "PubMed" OR "LILACS" OR "PMC" OR "GREY-COVIDWHO" OR "Indonesian Research" OR "CAB Abstracts" OR "APA PsycInfo" OR "CINAHL" OR "PUBMED" OR "Africa Wide Information" OR "Centers for Disease Control and Prevention")

AND document type:("article" OR "clinical trial register" OR "non-conventional")
(preprints have been removed)

Results: 2121

**Google scholar** (first 200 results out of 21'100 in total)
results according to relevance ranking, 2019-2030

covid-19|coronavirus "predictive|prediction|prognostic|prognosis|learning|risk model|score|tool|markers|biomarker|machine|software" ICU|intubation|ventilation|"high-flow nasal"|oxygenation|HFNC|ECMO|mortality|death|survival validation|validate
