# Supplementary material for: Prognostic models in COVID-19 infection that predict severity: a systematic review
Source: Eur J Epidemiol. 2023 Feb 25;38(4):355–72. doi: 10.1007/s10654-023-00973-x (PMC9958330; doi:10.1007/s10654-023-00973-x)
Supplement: Supplementary file 2 — Supplementary file2 (DOCX 52 KB) [file 10654_2023_973_MOESM2_ESM.docx]

**Table 1: General characteristics of the eligible studies**

| **Outcomes** | **Mortality (n = 152)** | **Severity or critical illness (n = 66)** | **Mortality and ICU admission (n = 35)** | **ICU admission (only) (n = 17)** | **Mechanical Ventilation (n = 6)** | **Combined Outcomes (n = 38)** |
| --- | --- | --- | --- | --- | --- | --- |
| **1. Publication year** |  |  |  |  |  |  |
| 2020 | 27 | 12 | 4 | 4 | 0 | 2 |
| 2021 | 70 | 36 | 21 | 10 | 4 | 20 |
| 2022 | 55 | 18 | 10 | 3 | 2 | 16 |
| **2. Study location** |  |  |  |  |  |  |
| Asia | 53 | 36 | 7 | 8 | 2 | 5 |
| Europe | 58 | 17 | 17 | 5 | 1 | 15 |
| North and Central America | 25 | 7 | 7 | 2 | 1 | 10 |
| South America | 6 | 1 | 1 | 1 | 2 | 5 |
| Africa | 2 | 3 | 0 | 1 | 0 | 1 |
| Australia | 0 | 0 | 1 | 0 | 0 | 0 |
| Multi-national or combined | 8 | 2 | 2 | 0 | 0 | 2 |
| **3. Sample size** | 11 - 6,952,440 | 55 -7,704,171 | 53 - 5,831 | 67 - 4,663 | 100 -2,040 | 44 - 76,588 |
| **4. No. of centers** |  |  |  |  |  |  |
| Single center | 75 | 34 | 24 | 14 | 4 | 19 |
| Multi-center | 71 | 31 | 11 | 3 | 2 | 19 |
| Not Reported | 6 | 1 | 0 | 0 | 0 | 0 |
| **5. Data source** |  |  |  |  |  |  |
| Retrospective cohort study | 135 | 61 | 29 | 15 | 6 | 29 |
| Prospective cohort study | 16 | 4 | 6 | 2 | 0 | 8 |
| Ambispective study | 1 | 1 | 0 | 0 | 0 | 1 |
| **6. Patient’s characteristics** |  |  |  |  |  |  |
| Male (%) | 31.8 - 100.0 | 41.4 - 77.0 | 46.8 - 70.3 | 39.5 - 75.78 | 50.8 - 71.0 | 34.1 - 77.9 |
| Mean age (years) | 18.0 -93.0 | 38.2 - 87.0 | 43.0 - 82.8 | 40.0 -71.4 | 52.0 - 66.0 | 37.0 -72.5 |
| **7. Reporting guidelines** |  |  |  |  |  |  |
| TRIPOD | 31 | 9 | 5 | 3 | 0 | 7 |
| STROBE | 4 | 1 | 4 | 2 | 1 | 2 |
| Not available | 117 | 56 | 26 | 12 | 5 | 29 |
| **8. Model development** |  |  |  |  |  |  |
| Logistic Regression Analysis | 95 | 38 | 16 | 12 | 4 | 16 |
| Machine learning | 38 | 18 | 6 | 3 | 1 | 16 |
| Not available | 19 | 10 | 13 | 2 | 1 | 6 |
| **9. Predictive performance (range (95%CI))** |  |  |  |  |  |  |
| AUC | 0.49 - 0.99 | 0.57 - 0.99 | 0.63- 0.98 | 0.44 - 0.97 | 0.73 - 0.92 | 0.53 - 0.94 |
| SENS (%) | 15.4 -100 | 7.1 – 100 | 10.5 - 98.7 | 30.2 - 92.4 | 81 - 96.4 | 21.5 - 98.6 |
| SPEC (%) | 10.9 - 98.7 | 19.5 -100 | 41 - 100 | 45.5 -99.7 | 70.3 - 89 | 13.7 - 89.2 |
| **10. Model development and validation** |  |  |  |  |  |  |
| Development | 32 | 9 | 4 | 2 | 1 | 9 |
| Development and internal and/or external validation | 83 | 39 | 11 | 10 | 2 | 22 |
| Validation (only) | 13 | 3 | 4 | 1 | 2 | 3 |
| Comparison/assessment/investigation/evaluation of biomarkers | 24 | 15 | 16 | 4 | 1 | 4 |
| **Abbreviations:** AUC; Area Under the Curve, SENS; Sensitivity, SPEC; Specificity, CI; Confidence Interval, TRIPOD; Transparent reporting of a multivariable prediction model for individual prognosis or diagnosis, STROBE; Strengthening the Reporting of Observational Studies in Epidemiology, EQUATOR: Enhancing the QUAlity and Transparency Of health Research | | | | | | |

**Table 2: Characteristics of eligible studies with mortality as outcome**

| **Reference #** | **First Author (year)** | **Model** | **Predictors in the final model** | **Model predictive performance** | | | |
| --- | --- | --- | --- | --- | --- | --- | --- |
|  |  |  | | **Accuracy (95%CI)** | **AUC/ROC (95%CI)** | **Sensitivity (95%CI)** | **Specificity (95%CI)** |
| [12] | Acar et al. (2021) | Nomogram | Nomogram based on age and 7 additional parameters (comorbidity, dyspnea, SpO2(%), HCT, CRP, AST, Ferritin) | NR | 0.92 | NR | NR |
| [4] | Aciksari et al. (2021) | M-ATRIA RS | M-ATRIA-RS, troponin I, LDH and history of malignancy | NR | 0.74 (0.70-0.79) | NR | NR |
| [14] | Al Abbasi et al. (2020) | Elevated troponin-I levels in the first 24 hours of admission | Age, atrial fibrillation, coronary artery disease, hypertension, diabetes mellitus, peripheral vascular disease, severe valvular disease, asthma, serum creatinine, lactic acid, total bilirubin, serum glucose, blood urea nitrogen, serum sodium, and absolute lymphocyte count | 79% (74 – 84) | NR | 66% (52 – 78) | 83 % (77% – 87%) |
| [17] | Alfaro-Martınez et al. (2021) | Alba-Score | Diabetes mellitus, Onco-hematologic disease, Confusion, Age, Heart rate, SaFiO_2_, LDH | NR | Generating cohort (numerical scores: 0.862 categorical scores: 0.85)  Validation cohort  numerical scores: 0.85 categorical scores: 0.83 | NR | NR |
| [21] | Allahverdiyev et al. (2020) | NLR (neutrophil-to-lymphocyte ratio) | Age, NLR, LDH, glomerular filtration rate, ALT, AST on admission | NR | 0.84 (0.79 – 0.89) | 92% | 53% |
| [22] | Altschul et al. (2020) | Novel COVID-19 severity score | Age (> 60, > 70 and > 80 years), female sex, oxygen saturation, MAP, PT/INR, creatinine, BUN, interleukin-6, CRP and PCT | NR | Derivation cohort: 0.82 (0.81–0.85) Validation cohort: 0.80 (0.79–0.82) | NR | NR |
| [23] | Andreano et al. (2021) | Multivariate logistic regression model | Age, sex, 32 comorbidities and 5 interaction terms | NR | Derivation: 0.79 Internal validation: 0.784 | NR | NR |
| [26] | Asghar et al. (2020) | Biochemical markers | LDH, PCT, D-dimer, CRP and ferritin | NR | PCT: 0.77 D-dimer: 0.83 LDH: 0.72 CRP: 0.70 Ferritin: 0.64 | PCT: 85% D-dimer: 79.5% LDH: 59.8% | NR |
| [35] | Bertsimas, 2020 | COVID-19 Mortality Risk (CMR) tool | Age, spO_2_, CRP, blood urea nitrogen and blood creatinine | 86.8% (82.3 - 91.2) | 0.92 (0.88-0.95) | NR | 87.4(83.0-91.8) |
| [37] | Besutti et al. (2021) | Prognostic model based on clinical features | Age, sex, HDL cholesterol, dementia, heart failure, vascular disease, days from symptom Onset, neutrophils, LDH, SpO_2_ (CT extension: two multivariate prognostic models, one with and one without rating of chest CT) | NR | Model without CT extension: 0.94 (0.92–0.96), validation: 0.93 (0.90–0.95) Model with CT extension: 0.95 (0.93–0.96)  Validation: 0.94 (0.91–0.95) | NR | NR |
| [41] | Cai et al. (2021) | Nomogram | Age, CRP, and D-dimer | NR | Primary: 0.81 (0.71-0.90) Validation: 0.90 (0.80-1.0) | Primary: 72.9% Validation: 86.4% | Primary: 74.8% Validation: 89.5% |
| [42] | Cheng P et al. (2021) | APACHE II score  MuLBSTA score | APACHE II: Temperature, Heart rate, Breathing rate, BP, Oxygen partial pressure, PH, K+, Na+, Creatinine, HCT, WBC, consciousness, age and chronic health evaluation  MuLBSTA: multilobular infiltration, lymphocytes, bacterial infection, smoking status, hypertension and age | NR | Death  APACHE II: 0.90 MuLBSTA: 0.9 | NR | NR |
| [51] | Ebell et al. (2021) | COVID-NoLab  COVID-SimpleLab | Covid-NoLab model: Age, respiratory rate and oxygen saturation.   Covid-SimpleLab model: Age, respiratory rate, oxygen saturation, WBC, CRP, serum creatinine, and comorbid asthma | NR | COVID-NoLab: AUROCC: 0.77 (derivation group) 0.80 (validation group)   COVID-SimpleLab:0.83 (derivation group)  0.83 (validation group) | NR | NR |
| [54] | Fan et al. (2021) | NRL Model  NL model | Neutrophil percentage and lactate dehydrogenase with and without oxygen saturation (SaO_2_) using the training data‐ set | NR | Training set 0.93 (NRL model)  0.90 (NL model)  Test set  0.91 (NRL model) 0.87 (NL model) | NR | NR |
| [59] | Gue et al. (2020) | COVID mortality score | Age, sex and mSIC score (modified sepsis-induced coagulopathy) | NR | 0.79 (0.74 – 0.84) - development cohort  0.77 - validation cohort | 67.6% | 78.4% |
| [62] | Hajifathalian et al. (2020) | COVID-AID risk tool | Age, MAP, presence of severe hypoxia, presence of kidney dysfunction | NR | Internal validation  7-day: 0.86 (0.74 – 0.98)  14-day: 0.83 (0.69 – 0.97)   External validation 7-day: 0.85 (0.78 – 0.92) 14-day: 0.82 (0.76 – 0.89) | NR | NR |
| [70] | Hu Hai et al. (2020) | REMS | MAP, pulse rate, respiratory rate, SaO_2,_ GCS and age | NR | 0.84 (0.76 - 0.90) | 89.5% | 69.8% |
| [68] | Hu C et al. (2021) | Logistic regression model | Age, hs-CRP, lymphocyte count and d-dimer | NR | Derivation: 0.89 External Validation: 0.88 | 89.2% 83.9% | 98.7% 79.4% |
| [69] | Hu Haifeng et al. (2020) | COVID-19 risk model | Age, comorbidities, lymphocyte count, neutrophil count, NLR, albumin and CRP | NR | 0.92 (0.832-1.00) | 90.5% | 84.2% |
| [75] | Jiang et al. (2021) | ABCS-mortality score | ABCS; age, biomarkers, COPD, sex and 7 biomarkers (aspartate aminotransferase, hs-CRP, high-sensitivity troponin I, WBC count, lymphocyte count, D-dimer and PCT) | NR | Derivation: 0.89 (0.87 –0.91) Validation: 0.84 (0.78–0.90) | NR | NR |
| [80] | King et al. (2020) | VACO index | Demographics (age, sex, ethnicity), pre-existing medical conditions and the Charlson Comorbidity Index (CCI) derived from ICD-10 | NR | Development: 0.79 (0.77–0.81) Early validation: 0.81 (0.78–0.83)  Late validation: 0.84 (0.78–0.86) | NR | NR |
| [82] | Knight et al. 2020) | 4C Mortality Score | Age, sex, comorbidities, respiratory rate, peripheral oxygen saturation, level of consciousness, urea level, and CRP | NR | 0.79 (0.78 - 0.79)  Validation: 0.77 (0.76 - 0.77) | NR | NR |
| [84] | Laguna-Goya et al. (2021) | IL-6-based mortality risk model | IL-6 level, LDH level, NLR, SpO_2_/FiO_2_ ratio and age | NR | 0.94 (0.89 - 1.00) | 88% | 89% |
| [89] | Li J et al. (2020) | PLANS model | Platelet count, lymphocyte count, age, neutrophil count and sex | NR | NR | NR | NR |
| [90] | Li et al. (2021) | Prognostic Nomogram | Age, disease severity at admission, dyspnea, heart disease, LDH, total bilirubin, blood glucose, urea | NR | NR | NR | NR |
| [93] | Liu Q et al. (2020) | Prediction model | D-dimer, Lymphocytes, BUN, PRE | NR | 0.99 (0.98 – 1.00) | 100% | 97.2% |
| [92] | Liu H et al. (2021) | PAWNN score | Platelet count, age, WBC count, neutrophil count, and NLR | 91.1% | Training cohort ranges: 0.92 (0.91–0.93) - 0.93 (0.92–0.94) Internal validation: 0.97 External validation: 0.80 | 93.8% (90.5– 98.1) | 90.9% (85.1 –92.8) |
| [94] | Liu S et al. (2020) | SOFA qSOFA | SOFA score: PaO_2_/FiO_2_, Creatinine, bilirubin, MAP, platelets, GCS;  qSOFA: respiratory frequency, systolic BP, vigilance | 84.2% 75.8% | SOFA: 0.91 (0.85–0.96) qSOFA: 0.74 (0.66–0.82) | 90% 70% | 83.2% 80.4% |
| [95] | López-Escobar et al. (2021) | RIM Score | Age, sex, SaO_2_, CRP, NPR, NLR, VNPR and VNLR | NR | Internal validation Model with NPR: 0.86 (0.82–0.90) Model with NLR: 0.853 (0.81–0.89) Model with VPNR: 0.90 (0.86–0.93) Model with VNLR: 0.86 (0.83–0.90) | NR | NR |
| [97] | Ma X et al. (2020) | Death prediction model | Age, demographics, symptoms and laboratory tests | NR | Age: 0.91 (0.83–0.98) Combined model: 0.98 (0.96–1.00) | NR | NR |
| [98] | Ma et al. (2020) | Nomogram based on 7 variables | Age, CHD, lymphocyte %, platelet, CRP, LDH, D-dimer | NR | 0.95 (0.92–0.97) | NR | NR |
| [99] | Magro et al. (2021) | Simple clinical prediction app | Age, sex, duration of symptoms before hospital admission shorter than 10 days, type 2 diabetes, CHD, chronic liver disease, LDH | NR | Internal validation: 0.82 (0.72–0.92) External validation: 0.82 (0.72–0.92) | NR | NR |
| [117] | Pan et al. (2020) | Nomogram | CRP, PaO_2_/FiO_2_, cTnI | NR | Primary: 0.99 (0.97–1.00) Validation: 0.96 (0.87–1.00) | NR | NR |
| [119] | Pigoga et al. (2021) | AFEM COVID-19 Mortality Scale (AFEM-CMS) | Male sex at birth, age, comorbidities, number of comorbidities, GCS, systolic BP, respiratory rate and heart rate | NR | NR | NR | NR |
| [122] | Quanjel et al. (2020) | NR | LDH, hs-CRP and lymphocytes % | 27.0% | NR | NR | NR |
| [131] | Satici et al. (2020) | CURB-65 PSI | CURB-65: confusion, urea, respiratory rate, BP, and age  PSI: age, long-term care facility residency, comorbidity, symptoms at diagnosis, laboratory measurements | NR | CURB-65: 0.79 (0.72 – 0.86) PSI: 0.85 (0.78 – 0.90) | CURB-65: 73% (59–83) PSI: 80% (67 - 90) | CURB-65: 85% (82-88) PSI: 89% (86 - 91) |
| [132] | Selcuk et al. (2021) | D-dimer-focused model | Age, D-dimer, WBC count and creatinine | NR | 0.90 (0.84 - 0.97) | 83.2% | 84.7% |
| [134] | Shang et al. (2020) | COVID-19 scoring system (CSS) | Age, CHD, LYM%, PCT and D-dimer | NR | Training cohort: 0.92 (0.87 - 0.97) Validation cohort: 0.94 (0.90 - 0.97) | NR | NR |
| [137] | Soto-Mota et al. (2020) | LOW-HARM Score | Lymphopenia, SaO_2_, WBC, hypertension, age, renal injury, and myocardial injury | NR | 0.96 (0.94 – 0.98) | NR | 97.5% |
| [138] | Stachel et al. (2021) | Machine learning algorithms (Gradient boosting decision trees (GB) - highest performance) | Oximetry, respirations, BUN, lymphocyte %, calcium, troponin and neutrophil % | 82% | Gradient boosting decision trees (GB):  0.83 (0.80 - 0.86) | 53% | 91% |
| [140] | Tanboga et al. (2021) | Multivariable logistic regression model | Age, LDH, CRP, NLR, creatinine, D‐dimer, albumin, hemoglobin, platelet count, presence of HF, diabetes and pneumonia on CT | NR | Derivation: 0.94 (0.94 – 0.94) Internally validated: 0.94 | NR | NR |
| [141] | Tezza et al. (2021) | Random forest model | Age, vital signs (oxygen saturation and the quick SOFA) and lab parameters (creatinine, AST, lymphocytes, platelets, and hemoglobin) | NR | 0.84 (0.78 -0.9) | 78.7% (68 - 86) | 77.4% (72 - 82) |
| [148] | Wang X et al. (2020) | NLR model | CK, albumin, AST, serum creatinine and NLR | NR | NLR (cut-off=3.338): 0.96 (0.91-1.00) | 100% | 84% |
| [147] | Wang et al. (2020) | MEWS | Systolic BP, pulse rate, respiratory rate, temperature and level of consciousness | NR | MEWS (cut off= 4.5): 0.91 (0.86 – 0.94) | 67.6% | 94.5 |
| [5] | Weng et al. (2020) | Nomogram (ANDC) | ANDC: Age, NLR, D-dimer, and CRP | NR | Derivation:0.92 (0.83 – 0.97)  External Validation: 0.97 | NR | NR |
| [151] | Wongvibulsin et al. (2021) | SCARP | Age, clinical severity, sex, demographics, admission source, time-varying vital signs, and comorbid condition | NR | Week 1: 0.89 (0.88 – 0.90) Week 2: 0.89 (0.87– 0.91) | NR | NR |
| [153] | Yang et al. (2021) | Nomogram | Age, breathing rate, lymphocyte count and interleukin-6 | NR | Validation 1: 0.81 (0.76 – 0.96) Validation 2: 0.862 (0.70 – 0.92) | 77.3% 92.9% | 73.5% 64.5% |
| [156] | Yuan Y et al. (2020) | Risk score based on 3 biomarkers | LDH, hs-CRP and lymphocyte % | NR | 0.96 | NR | NR |
| [157] | Zayed et al. (2021) | CT-SS Score and CO-RADS score | CT-scans | NR | CT-SS: 0.89 (0.84 - 0.95) CO-RAD: 0.97 (0.95 - 0.99) | NR | NR |
| [158] | Zeng et al. (2021) | Nomogram | WBC, CRP, Lymphocyte, LDH | NR | Derivation:0.94(0.91 – 0.98) Validation: 0.98 -  Combined cohorts: 0.95 | NR | NR |
| [159] | Zhang S et al. (2020) | Nomogram | Age, LDH, AST, PT, Sodium, Fibrinogen, D-dimer, NLR, direct bilirubin | NR | NR | NR | NR |
| [160] | Zou X et al. (2020) | APACHE II score |  | NR | 0.97 (0.94 – 0.99) | 96.1% | 86.3% |
| [13] | Ahirwar et al. (2022) | Biomarker-based model | hs-CRP, ferritin, IL-6 and D-dimer | NR | Plasma D-dimer = 0.99 (0.97 -1.00) | NR | NR |
| [16] | Aletreby et al. (2022) | 4C ISARIC Mortality score | NR | NR | 0.81 (0.79 – 0.83) | 70.5% | 74% |
| [45] | Churpek et al. (2021) | eXtreme Gradient Boosting (XG-Boost)  STOP-COVID Mortality Index (SCMI) | Age, number of ICU beds, serum creatinine, LDH, arterial pH and P/F ratio | NR | XG-Boost - external validation: 0.81 SCIM- external validation: 0.78 | NR | NR |
| [125] | Raschke et al. (2022) | C-TIME (COVID-19 Time of Intubation Mortality Evaluation) | Age, sex, COPD, MAP, GCS score, PaO_2_/FiO_2_, creatinine, bilirubin, days receiving non-invasive respiratory support, days receiving corticosteroids and treatment with oral factor Xa inhibitors | NR | 0.75 (0.72 – 0.79) | NR | NR |
| [126] | Reina, Reina et al. (2022) | Machine learning model | Chronic airway obstruction, acute respiratory failure, age, sex | 84.2% | 0.871 | 83.3% | 84.3% |
| [127] | Riley et al. (2022) | 4C Mortality Score | Age, sex, comorbidities, respiratory rate, peripheral oxygen saturation, GCS, BUN, and CRP | NR | 0.85 (0.79 - 0.89) | NR | NR |
| [133] | Shanbehzadeh et al. (2022) #409 | ANN-based CDSS | Vomiting, oxygen therapy, loss of taste, loss of smell, rhinorrhea, WBC, platelet count, absolute neutrophil count, ESR, pleural fluid, ICU admission, length of hospitalization, and age | NR | Back-Propagation -Artificial Neural Networks = 0.89 | NR | NR |
| [135] | Singh et al. (2022) | Biomarker-based model | BUN, Albumin, BAR, CRP, and D-dimer | NR | Albumin:  0.73 (0.63 – 0.82) | 73% | 64% |
| [139] | Surme et al. (2022) | SAD-60 score | SpO2, albumin, D-dimer and age | NR | 0.78 | NR | NR |
| [144] | Van de Leur et al. (2022) | (a) Logistic regression model (b) LASSO model (c) Deep Neural Network (DNN) model | (a) Logistic regression baseline model: age and sex; (b) LASSO model: age, sex and human annotated ECG features; (c) Pre-trained DNN model: age, sex and the raw ECG waveforms. | NR | Model (a) = 0.73 (0.65 – 0.79) Model (b) = 0.76 (0.68 – 0.82) Model (c) = 0.77 (0.70 – 0.83) | NR | NR |
| [146] | Vieira et al. (2022) | Risk score | Age, need for intubation, diabetes, CVD and a laboratory or cardiological characteristic | NR | Age = 0.72 CRP = 0.84 D-dimer = 0.73 | Age: 61.9% CRP: 73.7% D-dimer :67.5% | Age: 63.3% CRP: 67.5% D-dimer: 68.4% |
| [149] | Webb et al. (2022) | Simple scoring model | Age, comorbidities severe immunocompromised status, BMI, ethnicity and sex | NR | Derivation cohort:0.91 (0.83 – 0.94) Validation cohort :0.8 (0.69 – 0.90) | NR | NR |
| [150] | Wirth et al. (2022) | 4C Deterioration Model and 4C Mortality Score | Deterioration Model: age, sex, nosocomial infection, GCS score, SpO2 at admission, breathing room air or oxygen therapy, respiratory rate, CRP, lymphocyte count, and presence of radiographic chest infiltrates.   Mortality Score: age, sex, respiratory rate, SpO2, GCS, CRP, and number of comorbidities | NR | 4C Deterioration Model = 0.78 (0.73 – 0.82)  4C Mortality Score = 0.85 (0.79 – 0.89) | NR | NR |
| [154] | Yilmaz et al. (2021) | CURB-65 Score  GESTALT | CURB -65 (confusion, BUN, respiratory rate, BP and age)  GESTALT ( | NR | CURB-65 Score: 0.67  Gestalt:0.63 | CURB-65 Score = 50%  Gestalt = 78.9% | CURB-65 Score = 84.1%  Gestalt = 44.4% |
| [87] | Leoni et al. (2021) | Multivariable prediction model | Age, obesity, procalcitonin, SOFA score and PaO_2_/FiO_2_ | NR | NR | NR | NR |
| [102] | Marincu et al. (2021) | Logistic regression model | Comorbid conditions, the number of comorbid conditions, sex, and age | NR | 0.773(0.71 – 0.84) | NR | NR |
| [113] | Ottenhoff et al. (2021) | Extreme gradient boosting (XGB-10) and Logistic Regression (LR) | Age, number of home medications, urea nitrogen, LDH, albumin, oxygen saturation (%), oxygen saturation is measured on room air, oxygen saturation is measured on oxygen therapy, blood gas pH and history of chronic cardiac disease. | NR | XGB-10: 0.82 (0.79 - 0.85)  LR: 0.81 (0.77 - 0.85) | NR | NR |
| [129] | Rozenbaum et al. (2021) | Light Gradient Boosting Machine (Light-GBM), a machine learning algorithm | 10 most important variables only |  | Validation cohort (mortality) 7-day: 0.86 14-day: 0.88  30-day: 0.85 | NR | NR |
| [130] | Ruscica et al. (2021) | Age and biochemical parameters-based model | Age, NT-proBNP, IL-6 and LDH | NR | 0.88 (0.71 - 0.95) | 89% | 71% |
| [143] | Valente Silva et al. (2021) | M-CHA2DS2-VASc Score | Modified CHA2DS2-VASc (congestive heart failure, hypertension, age ≥75, diabetes mellitus, prior stroke or transient ischemic attack, vascular disease, age 65–74 years, female) scheme | NR | 0.71 (0.64 - 0.77) | NR | NR |
| [50] | Cui et al. (2022) | Random forest (RF) algorithms models | Younger group: LDH, NEU, FIB, PT, CA, WBC, PCT, IL-6, LY, D-dimer and IL-10  Elderly group: HCO3, LDH, AST, hs-CRP, IL-10, NEU, HsCTnT, TCHO, IL-2R, IL-6 and TNF-a | NR | ≤70 years old:) is 0.87 (0.83 – 0.91) >70 years old: 0.84 (0.76 – 0.92). | NR | NR |
| [52] | Ergenç et al. (2022) | Biomarker | Procalcitonin/albumin ratio | NR | 0.95 | 87.3% | 91.3% |
| [53] | Falandry et al. (2022) | 8 variables (IADL8) score | Age increase of 10 years and IADL8 | NR | Derivation: 0.78 (0.72 - 0.85)  Cross-validation: 0.80 (0.66 – 0.88) | NR | NR |
| [101] | Marcolino et al. (2021) | Risk score | Age, blood urea nitrogen, number of comorbidities, CRP, SpO_2_ /FiO_2_ ratio, platelet count, and heart rate | NR | GAM:  Derivation cohort: 0.88 (0.88 - 0.89);  Validation cohort: 0.87 (0.86 - 0.88).  LASSO:  Derivation cohort - 0.84 (0.84 - 0.85);  Validation cohort - 0.86 (0.85 - 0.86).  ABC 2 -SPH:  Derivation cohort: 0.84 (0.84 - 0.84);  Validation cohort: 0.86 (0.85 - 0.86);  External validation: 0.89 (0.87 - 0.92). | CD | CD |
| [19] | Alkaabi et al. (2021) | Risk score | Age, LDH, Creatinine, Glasgow comma scale, neutrophils (%), SpO_2_ and respiratory rate | NR | 0.88 (0.86 - 0.92) | 81% | 79% |
| [100] | Mahdavi et al. (2021) | Machine learning model | Age, SpO_2_, CVD, PTT, BUN and LDH | 80% (79.7%-80.3%) | Joint model: 0.85 (0.847 - 0.854) | 73% | 88% |
| [108] | Murri et al. (2021) | Machine learning model | Age, platelet count, SpO_2_, BUN, hemoglobin, CRP, neutrophil count and sodium | NR | Training set: 0.87;  Testing set: 0.82 | Training set: 84%; Testing set: 81.3% | Training set: 76.6%; Testing set: 65% |
| [65] | Heber et al. (2021) | Predictive model | Age, fever on admission, LDH, platelet count, CRP, LYM and creatinine | NR | Development: 0.88 (0.83 - 093), Validation: 0.92 | NR | NR |
| [79] | Kilercik et al. (2021) | Hematocytometry index | MNR, NLR, PLT, RDW | 84.8% | 0.91(0.89-0.93) | 61.6% | 93.8% |
| [15] | Al Mutair et al. (2021) | Predictive model | Age, sex, cough, septic shock, ARDS, AKI, NEUT, D-dimer, BUN, Blood culture | NR | NR | NR | NR |
| [152] | Yang et al. (2021) | Machine learning model | LDH, NLR and CRP | 98% | Decision tree model: 0.96, LDH: 0.94, NLR: 0.95, CRP: 0.90 | NR | NR |
| [145] | Vicka et al. (2021) | Four prognostic scores were compared (4cmortality score, SAPPS II, APACHE II and SOFA score | NR | NR | SOFA: 0.68 (0.61 - 0.75);  SAPS II: 0.75 (0.69 - 0.81);  APACHE II: 0.77 (0.71 - 0.83);  4C mortality score: 0.75 (0.69 - 0.81). | NR | NR |
| [72] | Jain et al. (2021) | Predictive model | Age, Charlson comorbidity index, CRP, CT severity and D-dimer on admission | NR | Age: 0.49 (0.31- 0.68); CMI:0.58 (0.42 - 0.75); CRP (admission): 0.75 (0.61 - 0.89); D-dimer (admission): 0.86 (0.74 - 0.99); CT severity (admission): 0.73 (0.57 - 0.88) | Age: 80%, CMI:80%, CRP (admission): 80%, D-dimer (admission): 80%, CT severity (admission): 80% | Age: 73%, CMI:66.4%, CRP (admission): 44.2%, D-dimer (admission): 10.9%, CT severity (admission): 42.7% |
| [124] | Rahman et al. (2021) - Dev. And Val. Of an early scoring system | Prognostic model, nomogram and machine learning | Age, RB-DW, WBC, lymphocyte count, monocytes, platelet count, neutrophils count | Development: 88%;  Validation: 7 features: 91% | Development set: 0.95;  Internal validation: 0.88  External validation: 0.96 | Development: 87%; Validation: 7 features - 91% | Development: 90; Validation: 7 features – 91% |
| [121] | Ponce et al. (2021) | Machine learning model | Age, MV, Vasopressors, WBC, Severe infection at admission, COVID infection at hospital, AST, Renal replacement, moderate infection at admission, AKI etiology sepsis, hypertension, creatinine, AKI etiology COVID, sex, time from COVID to AKI, normal diuresis, nephrotoxic medication and dehydration | NR | Derivation cohort (Elastic net): 0.88 (0.83 - 0.97);  Validation cohort: 0.82 (0.75 - 0.88). | NR | NR |
| [61] | Haji Aghajani et al. (2021) | Nomogram | Age, sex, need for ICU/MV, pulse rate, lymphocytes, RBC, Troponin 1, LDH | Development: 81.9%;  Validation: 73.2% | Development: 0.82 (0.80 - 0.86), Validation: 0.80 (0.73 - 0.86) | Development: 72.3%; Validation: 64.4% | Development: 86.1%; Validation: 85.1% |
| [56] | Garrafa et al. (2021) | Brescia early-warning model | Age, LDH, D-dimer, NLR, CRP, lymphocyte %, ferritin std and monocyte % and Brescia chest X-ray score | NR | Development: 0.97 (0.97– 0.98);  Validation: 0.83 (0.80 – 0.87) | Development: 93% (91– 97); Validation: 82% (72 – 92) | Development: 0.92% (88 – 94); Validation: 75% (63 – 83) |
| [64] | He et al. (2021) | Nomograms | Nomogram 1: age, dyspnea, anorexia, WBC, NLR, PLT, AST, Albumin, CRP;  Nomogram 2: age, dyspnea, NLR, CRP | Development  Nomogram 1: 85.3% (83.8-89.1);  Nomogram 2: 85.9% (84.5-87.4)  Validation  Nomogram 1: 88.7% (87.1–90.3);  Nomogram 2: 89.6% (88.0 – 91.1) | Development  Nomogram 1: 0.92 (0.88 - 0.96);  Nomogram 2: 0.90(0.85 - 0.94).   Validation  Nomogram 1: 0.92 (0.86–0.98);  Nomogram 2: 0.89 (0.83–0.96) | Development: n1: 86.4% (77.3 - 93.9), n2: 80.3% (70.7-89.9).  Validation: Nomogram 1: 86.7% (74.5 – 98.8); nomogram 2: 76.7% (61.5 – 91.8) | Development: n1:85.2% (83.6% - 88.9). N2: 86.1% (84.6-87.6).   Validation: Nomogram 1: 88.7% (87.1 – 90.3); nomogram 2: 89.8% (88.3 – 91.4) |
| [55] | Moghaddam-Tabrizi et al. (2021) | Prognostic model | Age, pulmonary symptoms, need for MV, brain symptoms, nasal airway, job, gastrointestinal symptoms, brain disease history, heart disease history, heart symptoms, CKD history, psychological symptoms, dyspnea symptom, history of drug use and hypertension | Training sample: 89.8% (85.6 - 93.1);  Validation: 94.7% (89.4 - 97.9) | Training sample: 82.9;  Validation: 79.3 | Training: 91.9% (87.6 - 95.1); Validation: 94.5% (88.5 - 97.9) | Training: 89.5% (77.3 - 96.5); Validation: 95.7% (73.9 - 99.8) |
| [111] | Nishikimi et al. (2021) | Intubated COVID‑19 predictive (ICOP) score | Age, past medical history for CKD, BUN, ferritin, oxygen index, pH, MAP and dose of needed vasopressor | NR | Derivation cohort: 0.75(0.73 - 0.78);  Validation: 0.71 (0.67 - 0.75) | NR | NR |
| [136] | Sosa et al. (2021) | Lung Ultrasound Score (LUS) | LUS | NR | LUS at admission: 0.64;  LUS at day 5 after admission: 0.80 | LUS at admission: 63%; LUS at 5^th^ day of admission: 75% | LUS at admission: 59%; LUS at 5th day of admission: 78% |
| [31] | Banoei et al. (2021) | Machine learning model | 18 clinical and comorbidities predictors and 3 | Training set: 90%;  Validation: 87% | Training set: 0.95;  validation 0.91 | Training set: 80% and validation: 75% | Training set: 92% and validation: 90% |
| [128] | Riva et al. (2021) | Prognostic model | Monocyte Distribution Width | NR | 0.76 (0.66 – 0.87) | 75% | 70% |
| [123] | Rahman et al. (2021) – (mortality prediction using machine learning) | Nomogram | Age, lymphocyte count, D-dimer, CRP and creatinine at hospital admission | Development: 91% | Development: 0.99;  Internal validation: 0.999  External validation: 0.99 | Development: 91% | Development: 78% |
| [142] | Timpau et al. (2021) | Prognostic model | D-Dimer and CRP | NR | CRP: 0.71 (0.62-0.79) D-Dimer: 0.74 (0.66-0.82) | CRP (cut-off=0.74): 65.7%; D-Dimer (cut-off=48.5): 61.8% | CRP (cut-off=0.74): 70.8%; D-Dimer (cut-off=48.5): 62.5% |
| [330] | Martín-Rodríguez et al. (2021) | Risk prediction model | Age, location (rural or urban), institutionalization, desaturation, rhonchus, tachypnea, and altered level of consciousness | NR | Derivation cohort: 0.72 (0.68-0.75);  Validation cohort: 0.74 (0.70-0.79);  Re-validation cohort: 0.76 (0.72-0.80) | Metrics were presented for three risk levels (i.e., low (L), intermediate (I) and high (H)) in three models, i.e., Global model, SARS-COV-2 negative and SARS-COV-2 positive. Global - L: 98% (94-100), I: 67% (57-76) and H: 12% (7-17); SARS-CoV-2 negative – L: 97% (94-99), I: 58% (47-68), H: 8% (3-13) and SARS-CoV-2 positive - L: 96% (93-98), I: 70% (59-80) and H: 13% (7-18). | Global - L: 27.9% (22-32), I: 64% (54-73) and H: 97% (95-98); SARS-CoV-2 negative – L: 25% (20-30), I: 63% (53-73), H: 98% (96-99) and SARS-CoV-2 positive - L: 32% (27-36), I: 61% (51-71) and H: 96% (94-98). |
| [76] | Kar et al. (2021) | Machine learning model | Age, Sex, ARDS, diabetes, CKD, CAD, respiratory rate, SpO_2_, Lymphocyte % in DLC, INR, LDH and Ferritin | Development: 97.1%;  Validation: 93.0% | Development: 0.88;  Validation: 0.78 | Development: 78.1% and validation: 60.5% | Development: 98.7% and validation: 96% |
| [155] | Yu et al. (2021) | Prognostic model with cardiac-specific biomarkers | Hs-TnI I, CK-MB and MYO | NR | Early stage  HS-Tnl (7.9 pg/ml): 0.84 (0.78-0.89);  CK-MB (1.2 ng/ml): 0.78 (0.72-0.85);  MYO (80.8 ng/ml): 0.89 (0.84-0.94);  Late stage  HS-Tnl (15.7 pg/ml): 0.94 (0.90-0.98);  CK-MB (1.5 ng/ml): 0.92 (0.87-0.96);  MYO (98.0 ng/ml):0.96 (0.92-1.00). | Early stage: HS-Tnl (7.9 pg/ml) – 77% (65-87); CK-MB (1.2 ng/ml) – 71% (59-82) and MYO (80.8 ng/ml) – 85% (74-93); Late stage: HS-Tnl (15.7 pg/ml) – 89% (79-96); CK-MB (1.5 ng/ml) - 85% (74-93) and MYO (98.0 ng/ml) – 91% (81-97) | Early stage: HS-Tnl (7.9 pg/ml) – 77% (75-80); CK-MB (1.2 ng/ml) – 76% (74-79) and MYO (80.8 ng/ml) – 86% (84-88); Late stage: HS-Tnl (15.7 pg/ml) – 94% (93-96); CK-MB (1.5 ng/ml) – 91% (89-93) and MYO (98.0 ng/ml) -96% (95%-97%) |
| [57] | Zelikovna-Golukhova et al. (2022) | Transthoracic Echocardiography-Based Prediction Model | Right ventricle/left ventricle area, sPAP and right ventricle free wall longitudinal strain | NR | 0.92 (0.86 – 0.99) | 93.8% | 81.9% |
| [18] | Alhamar et al. (2022) | Biomarker-based model | Sex, non-Kuwaiti nationality, asthma, glycemia categories | NR | Training cohort: 0.90;  Internal validation: 0.826 (+- 0.91);  External validation with cohort from Italy: 0.687 (+- 0.06) | Training cohort: 75%; Additional Kuwait cohort: 66.9%; Italian cohort: 66.7%. | Training cohort: 86.3%; Additional Kuwait cohort: 76.7%; Italian cohort :70.7%. |
| [74] | Jamshidi et al. (2022) | Machine learning-based mortality prediction model | Age, sex, medical history, symptoms (around 20 parameters in total) | NR | Training cohort: 0.80;  Validation cohort: 0.79 | Training set: 76.1%; Test set: 74.9 | NR |
| [24] | Araiza et al. (2021) | Ichikado CT score | Ichikado CT score | NR | Ichikado CT score (cut-off= 172 points): 0.873 | 84.2% | 79.7% |
| [20] | Alkhasawneh et al. (2021) | Single predictor | MLR | 715% | NR | 66.8 % | NR |
| [29] | Bae et al. (2021) | Radiomics based model | 32 radiomics features | NR | 0.83 (0.79-0.87) | 79% (72-86) | 74% (65-83) |
| [58] | Gordon et al. (2022) | 4C Mortality Score | Age, sex, respiratory rate, SpO_2_, number of comorbidities, level of consciousness, BUN and CRP | NR | 4C validation: 0.76 (0.76 - 0.77);  RECOVER: 0.79 (0.77 - 0.80) | NR | NR |
| [60] | Gutierrez-Camacho et al. (2022) | Charlson Comorbidity Index as mortality predictor | Charlson Comorbidity Index | NR | 0.75 (0.69 - 0.80) | 63.6% (54.8 – 71.8) | 87.7% (82.6 –91.7) |
| [63] | Hassan et al. 2022 | 4C mortality score; CURB-65 score | 4C: age, sex, respiratory rate, Oxygen saturation on room air, GCS, urea, CRP, the number of comorbidities;  CURB-65: mental confusion, urea, respiratory rate, BP and age | NR | NR | NR | NR |
| [66] | Hippisley-Cox et al. (2021) | Cause-specific Cox proportional hazard models | Age, sex, ethnicity, deprivation, BMI, comorbidities, and SARS- CoV-2 infection rate. | NR | NR | NR | NR |
| [67] | Hohl et al. (2022) | The CCEDRRN COVID-19 Mortality Score (Canadian COVID-19 Emergency Department Rapid Response Network) | Age, sex, type of residence, arrival mode, chest pain, severe liver disease, respiratory rate and level of respiratory support need | NR | 0.92 (0.90 - 0.93) | NR | NR |
| [71] | Huang Chun‑Yen et al. (2022) | Hematologic and biochemical parameters | Age, sex, and comorbidities. | NR | CRP:0.678; PCT:0.74; LDH: 0.68; Ferritin: 0.68; D-dimer0.75; NLR: 0.57 | CRP:89.5%; PCT: 47.4%; LDH:52.6%; Ferritin: 47.4%; D-dimer: 73.7%; NLR:52.6%. | CRP: 46.2%; PCT:90.4%; LDH: 81.3%; Ferritin: 82.8%; D-dimer:75.1%; NLR: 66% |
| [73] | Jalalvand et al. (2022) | Logistic regression model | GGO pattern, age, lymphocyte count, Creatinine, CRP, LDH, and systolic BP | GGO: 94% (83-99);  Age: 66% (54-77);  Creatinine 18% (9-30); Lymph. count: 82% (70-90) | GGO: 0.96 (0.91, 1.00); Age: 0.79 (0.65, 0.93); Creatinine 0.76 (0.62, 0.89); Lymph. count: 0.79 (0.62, 0.96) | GGO: 100% (74 - 100); age: 83% (52 - 98); Creatinine: 100% (72 - 100%); Lymph. count: 78% (40 - 97) | GGO:92% (78 - 98); age: 63% (49 - 75); Creatinine 44% (30 - 59); Lymph. count: 82% (69%, 92%) |
| [77] | Khari et al. (2022) | qSOFA, CURB-65, SIRS | NR | NR | SIRS: 0.62 (0.55 - 0.69);  CURB-65: 0.66 (0.59 - 0.73);  qSOFA: 0.61 (0.54 - 0.67) | SIRS: 85.2% (75.7-91.6); CURB-65: 96.6% (89.7-99.1);  qSOFA: 78.4% (68.1-86.2) | SIRS: 34.3% (26.5-43);  CURB-65: 6.6% (3.2- 12.5);  qSOFA: 38.7% (30.6- 47.4) |
| [78] | Kibar Akilli et al. (2022) | PSI/PORT, A-DROP, NEWS-2, MEWS, CURB-65, Expanded CURB-65, qSOFA, qCSI, 4C mortality | NR | NR | PSI/POST: 0.97(0.96 - 0.98); A-DROP: 0.93(0.91 - 0.95) | PSI/POST: 91.7%; A-DROP: 84.2% | PSI/POST: 91.9%; A-DROP: 86.1% |
| [81] | Klen et al. (2022) | Machine learning-based online model CODOP | Age, neutrophils, CRP, creatinine, LDH, serum sodium, serum potassium, glucose and D-dimer, platelets, eosinophils and monocytes | NR | Range: 0·90 – 0·96 | Range: 78 –100% | Range: 89–97% |
| [83] | Kucuk, Berkay et al. (2022) | NUTRIC (nutrition risk in the critically ill) score, modified NUTRIC | NUTRIC: age, APACHE II score, SOFA score, the number of comorbidities, length of hospital stay before admission to ICU, and IL-6;  mNUTRIC: NUTRIC without IL-6 | NR | NUTRIC: 0.79 (0.74 - 0.85); mNUTRIC: 0.79 (0.74 - 0.83) | NR | NR |
| [85] | Laino et al. (2022) | Machine learning model | Age, P/F ratio, hs-TI, BNP, IL-6, procalcitonin, RDW, urea, creatinine, albumin | NR | 0.85 (0.82 – 0.87) | NR | NR |
| [88] | Li, G et al. (2021) | Biomarker | Age, sex; hypertension, diabetes, CHD; WBC, cTnI, hs-CRP and Cr. ROC: lowUA ≤ 166 μmol/L | NR | NR | 76.9% (68.5–85.1) | 74.9% (70.3–78.9) |
| [91] | Li, Y et al. (2022) | Two-step risk score | Age, comorbidity score≤1, respiratory rate<30/min, SpO2≥93%, hear rate ≥100/min, BUN, NLR, Platelets, CRP, NEU | NR | NR | NR | NR |
| [96] | Lyons et al. (2022) | QCOVID | NR | NR | NR | NR | NR |
| [103] | Martın-Rodrıguez et al. (2022) | Quick COVID-19 Severity Index (qCSI) and the National Early Warning Score (NEWS) | NR | NR | qCSI  0.77 (1-day mortality)  0.75 (90-day mortality),    NEWS:  0.82 for 1-day mortality  0.78 for 90-day mortality | NR | NR |
| [104] | Morello et al. (2022) | 4C Mortality dichotomic rule | NR | NR | 0.93 (0.84,1.00) | NR | NR |
| [105] | Moulaei et al. (2022) | Machine learning algorithms | Dyspnea, ICU admission, oxygen therapy, Age, fever, cough, Loss of taste, loss of smell, hypertension, contusion, muscular pain, chill, runny nose, Blood urea nitrogen, diabetes, sore throat, absolute lymphocyte count nausea/vomiting, other underlying disease: cardiac disease, chest pain and pressure, absolute neutrophil count, headache, sex, gastrointestinal symptoms, white cell count, C‐reactive protein, hypersensitive troponin, pneumonia, glucose, erythrocyte sedimentation rate, creatinine alkaline phosphatase, length of hospitalization, aspartate aminotransferase, smoking, alanine aminotransferase, platelet count | 95.0% | 0.99 | 90.7% | 95.1% |
| [106] | Mousavi et al. (2021) | Machine learning | RBC, platelets, hemoglobin, hematocrit, and platelet distribution width, all laboratory tests, age, sex, and the need for special care, such as oxygen therapy and ventilation | NR | NR | NR | NR |
| [107] | Munoz et al. (2022) | PESI | Age, sex, cancer, heart failure, chronic obstructive pulmonary disease, heart rate >110 bpm, BP, systolic < 100 mm Hg, respiratory rate > 30 rpm, Temperature < 36 centigrade degrees, altered mental status and arterial oxygen saturation (SaO2) < 90% | NR | 0.71 (0.63 - 0.79) | NR | NR |
| [109] | Najafi et al. (2021) | Age and kidney functions parameters model | Age, CKD, AKI, GFR | NR | NR | GFR<44.21 ml/min: 72.8% | GFR<44.21 ml/min: 65.8% |
| [110] | Naser et al. (2021) | Kidney function model | AKI | NR | NR | NR | NR |
| [112] | Ocho et al. (2022) | 4C mortality score | Age and sex | 84% (76-92) | NR | 82% | 74% |
| [114] | Özdemir et al. (2021) | Biomarkers | Albumin, CRP, D-Dimer, CRP/albumin ratio | NR | CRP/albumin :0.81 | 71.1% | 71.4% |
| [115] | Özdemir et al. (2022) | 3 scoring systems | Modified Early Warning Score (MEWS), Rapid Acute Physiology Score (RAPS) and Rapid Emergency Medicine Score (REMS) | MEWS:20.5%; RAPS:9.9%; REMS:35.1% | MEWS:0.51 (0.42 - 0.60); RAPS:0.50 (0.41 - 0.59); REMS:0.67 (0.58 - 0.76) | MEWS: 53.8%; RAPS: 15.4%; REMS: 84.6% | MEWS:25.7%; RAPS: 1596.5%; REMS: 50.5% |
| [116] | Ozger et al. (2021) | Model based on biomarkers measured serially at different sampling times | GCSF, IL-6, IL-7, IL-10, IL-15, IL-27 IP-10, MCP-1, D-dimer, Ferritin, CRP | NR | IL-6 = 0.69 (0.51– 0.83);  IL-10=0.64(0.46 – 0.79) | NR | NR |
| [118] | Pasculli et al. (2022) | Chest computed tomography score | Chest computed tomography findings; cycle threshold (Ct) values in RT-PCR of SARS-CoV-2; secondary infection occurrence | NR | CT= 0.68; Ct values= 0.75 | CT: 61.9% (55.3- 68.1); Ct values: 75.8% (69.3- 81.2) | CT: 68.8% (51.4- 82.1); Ct values: 69.4% (53.1- 82.0) |
| [120] | Plecko et al. (2022) | Rapid Evaluation of Coronavirus Illness Severity (RECOILS) score | Age, platelets, PaO_2_/FiO_2_ ratio, pH, BUN, temperature, PaCO_2_, GCS score measured within +/−24 h of ICU admission | NR | 0.78 (0.76 – 0.81) | NR | NR |
| [25] | Asaduzzaman et al. (2022) | Hemogram | Neutrophil‐to‐lymphocyte ratio (NLR), derived NLR (d‐NLR), and neutrophil‐to‐platelet ratio (NPR) | NR | NLR: 0.66 (0.58 – 0.73); d‐NLR: 0.65 (0.58 – 0.73); NPR: 0.68 (0.61 – 0.75); PLR: 0.55 (0.46 – 0.63); SII:0.60 (0.52 – 0.67) | NR | NR |
| [27] | Aygun et al. (2022) | Emergency department triage early warning score (TREWS) and modified early warning score (MEWS) | Emergency department Triage Early Warning Score (TREWS: which includes the patient age physiological parameters such as respiratory rate, oxygen saturation, need for oxygen support, body temperature, systolic blood pressure, heart rate, and consciousness.) and modified early warning score (MEWS includes; systolic BP, heart rate, respiratory rate, temperature) | NR | MEWS (28-day mortality): 0.83 (0.78– 0.89) | MEWS (cut-off = 0.5): 96.5% | 46.8% |
| [28] | Ayvat et al. (2022) | CT scan | APACHE, CT score, CRP, and age | NR | NR | NR | NR |
| [30] | Baikpour et al. (2022) | chest X-ray (CXR) scoring system | Age, sepsis, S/F ratio, need for MV and the CXR severity score | NR | 0.93 (0.89- 0.96) | NR | NR |
| [32] | Bartoszko et al. (2022) | Biomarkers and physiological variables | Age, temperature, LDH level, ventilation tidal volume, and vasopressor use | NR | 0.90 (0.80 - 0.90) | NR | NR |
| [33] | Beigmohammadi et al. (2022) | APACHE II and SOFA scores | APACHE II and SOFA scores | NR | SOFA Score: 0.89 | NR | NR |
| [34] | Bengelloun et al. (2022) | CONtrolling NUTritional status (CONUT) index | CONUT index, | NR | 0.71 (0.68 – 075) | NR | NR |
| [36] | Besutti et al. (2022) | CT scan, CXR, CRP or SpO2 | Age, sex, measures of disease severity and time from symptom onset | NR | CT: 0.92 (0.89 – 0.95);  CXR: 0.90 (0.86 – 0.94);  CRP: 0.88 (0.83 – 0.93); sO_2_: 0.88 (0.84 – 0.92) | NR | NR |
| [38] | Bezerra et al. (2022) | urinary biomarkers NGAL, KIM-1, MCP-1 and nephrin | Proteinuria*KIM-1*NGAL | NR | 0.81 (0.69– 0.93) | Proteinuria*KIM-1*NGAL (cut-off= 379 ng/mg-Cr:71% | Proteinuria*KIM-1*NGAL (cut-off= 379 ng/mg-Cr:6% |
| [39] | Bodolea et al. (2022) | Biological nutritional risk assessment instruments | Prognostic Nutritional Index (PNI), the Controlling Nutritional Status Score (CONUT), the Nutrition Risk in Critically Ill (NUTRIC), and the modified NUTRIC—mNUTRIC), along with CT-derived fat tissue and muscle mass measurements | NR | PNI had the best discriminative capabilities for mortality (cut-off = 28.05):0.77 | NR | NR |
| [40] | Bradley et al. (2022) | CURB-65  PSI | CURB-65 (Confusion, Urea, Respiratory Rate, BP, Age and the Pneumonia Severity Index (PSI) | NR | PSI: 0.82 (0.78 - 0.86);  CURB-65: 0.79 (0.75 - 0.84) | 83% | 66% |
| [43] | Chikhalkar et al. (2022) | NEWS-2 Score | NEWS-2 Score | 96% | 0.99 | NEWS2 score ≥ 5:97.2% | NEWS2 score ≥ 5: 96.6% |
| [44] | Chou et al. (2022) | NR | Age, BMI, CKD, CHF, Hepatitis, Transplant, NLR test × NLR, LDH test ×, LDH, NEWS score | NR | Derivation cohort: 0.94 (0.92 – 0.96) | NR | NR |
| [46] | Cidade et al. (2022) | Biomarker | D-Dimer levels | NR | 0.57 (0.42 - 0.71) | NR | NR |
| [48] | Citu Cosmin et al. (2022) -1686-evaluation | Multiple scores: 4C Mortality Score, NEWS and CURB-65 Scores | NEWS Score | NR | 0.86 (0.68 - 0.92) | NEWS >3: 100% | NEWS >3: 51% |
| [47] | Citu Cosmin et al. (2022) | Coagulation parameters: D Dimer, INR, PT, aPTT | D-Dimer | NR | 0.75 | D-Dimer (cut off 1.03): 76% | D-Dimer (cut off 1.03): 61% |
| [49] | Comoglu et al. (2022) | Charlson comorbidity index (CCI) | CCI, categorized into groups: CCI score 0, CCI score 1–2, CCI score 3–4, CCI score 5–6, and CCI score ≥7 | NR | 0.80 (0.75 – 0.86) | CCI score > 4: 87.2% | CCI score > 4: 61.4% |
| [86] | Lee DS et al. (2021) | Machine learning (Random Forest) | 304 variables including all demographic, interRAI functional, LTC-related, and community characteristics, along with comorbidities and laboratory tests | NR | 0.70 (0.67-0.74) | NR | NR |
| **Abbreviations:** Values are percentages or mean age ± SD as appropriate. SD: Standard Deviation, NR: value not reported or could not be found, PCR: polymerase chain reaction, Covid-19: Corona Virus Disease 2019, HFNT: High-Flow Nasal Therapy, ECMO: Extracorporeal Membrane Oxygenation, ICU: Intensive Care Unit, IMV: Invasive Mechanical Ventilation, MV: Mechanical Ventilation, Dev.: development, RT-PCR: Reverse Transcriptase - Polymerase chain reaction, AUC: Area Under the Curve, ROC: Receiver Operating Characteristic, SENS: Sensitivity, SPEC: Specificity, PPV: Positive Predicative Value, NPV: Negative Predicative Value, LR: Likelihood Ratio, C-statistics: Concordance Statistics, EDRF: Early deterioration of respiratory function, IRS: intensive respiratory support, CI: Confidence Interval, MEWS; Modified Early Warning Score, ANDC; Age, NLR, d-dimer, and CRP, PSI; Pneumonia Severity Index, Sp02; saturation of peripheral oxygen, HCT; Hematocrit, CRP; C-reactive protein, AST Aspartate aminotransferase, M-ATRIA-RS; Modified Anticoagulation and Risk Factors in Atrial Fibrillation Risk Score, LDH; lactate dehydrogenase, PCT; Procalcitonin, 4C Mortality Score (where 4C stands for Coronavirus Clinical Characterization Consortium), PLANS; Platelet count, lymphocyte count, age, neutrophil count, and sex, VNPR; Velocity of NPR (VNPR) and Velocity of NLR (VNLR), NPR; neutrophil-to-platelet-ratio, NLR; neutrophil-to-lymphocyte ratio, DD;D-dimer, ALB; Albumin, Lymr; lymphocyte ratio, Neur; neutrophil ratio, BUN; blood urea nitrogen, PRE; a combined predictive factor with lymr, BUN, DD, SOFA; Sequential Organ Failure Assessment , qSOFA; Quick Sequential Organ Failure Assessment, SCARP; Severe COVID-19 Adaptive Risk Predictor, COPD; chronic obstructive pulmonary disease, APACHE II; Acute Physiology and Chronic Health Evaluation II, LUS; Lung Ultra Sound, ICC: intraclass correlation coefficient, NEWS2; National Early Warning Score 2, BMI; Body Mass Index, ESR; erythrocyte sedimentation rate, CDCS; Charlson/Deyo comorbidity score, ALT; alanine aminotransferase, aPTT; activated partial thromboplastin time, CK; creatine kinase, WBC; white blood cell, cTn1; troponin I, IL-6; interleukin-6, DM; Diabetes Mellitus, BP; Blood Pressure. | | | | | | | |
