# Supplementary material for: Prognostic models in COVID-19 infection that predict severity: a systematic review
Source: Eur J Epidemiol. 2023 Feb 25;38(4):355–72. doi: 10.1007/s10654-023-00973-x (PMC9958330; doi:10.1007/s10654-023-00973-x)
Supplement: Supplementary file 3 — Supplementary file3 (DOCX 123 KB) [file 10654_2023_973_MOESM3_ESM.docx]

**Table 3: Baseline characteristics of the eligible studies**

| **No.** | **Author, (year)** | **Location** | **Sample size (N)** | **Age (years), mean ± SD** | **Male participants (%)** | **COVID-19 diagnosis** | | | **Baseline participants** | | |
| --- | --- | --- | --- | --- | --- | --- | --- | --- | --- | --- | --- |
|  |  |  |  |  |  | **PCR test** | **PCR + symptoms** | **Symptoms** | **Hospitalized** | **Hospitalized + in ICU** | **Non- hospitalized** |
|  | **Outcome: Mortality (only)** | | | | | | | | | | |
|  | Acar et al. (2021) | Turkey | 709 (NS:75; S:634) | 69 ± 14 | NS: 59 S:53 | **✔** |  |  | **✔** |  |  |
|  | Aciksari et al. (2021) | Turkey | 1001 (Group 1: 448; G 2: 268; G 3: 285) | G 1: 48 ± 2.8; G2: 60 ± 2; G 3: 76 ± 2.4 | G 1: 51.6; G 2: 58.2; G3: 64.4 | **NR** |  |  | **✔** |  |  |
|  | Al Abbasi et al. (2020) | United States | 257 (elevated troponin: 71); normal troponin: 186) | 63 ± 17 | Elevated troponin: 49; normal troponin:46 | **✔** |  |  | **✔** |  |  |
|  | Alfaro-Martınez et al. (2021) | Spain | 1470 | 68.1 ± 15.2 | 59.1 |  | **✔** |  | **✔** |  |  |
|  | Allahverdiyev et al. (2020) | Turkey | 455 | 58 ± 13.3 | 47.6 | **✔** |  |  | **✔** |  |  |
|  | Altschul et al. (2020) | United States | 4711 (DC: 2355; VC: 2356) | DC: 65.3 ± 15.9; VC: 61.4 ± 17.2 | DC: 46.7; VC: 59.8 | **✔** |  |  | **✔** |  |  |
|  | Andreano et al. (2021) | Italy | 18286 | 71 | 47.6 | **✔** |  |  | **✔** |  | **✔** |
|  | Asghar et al. (2020) | Pakistan | 364 | 52.6 ± 15.8 | 56 |  |  | **✔** |  | **✔** |  |
|  | Bertsimas et al. (2020) | 33 European countries & United States | 3062 | 68 ± 3.1 | 60.6 | **✔** |  |  | **✔** |  |  |
|  | Besutti et al. (2021) | Italy | 866 | 60.5 ± 3.4 | 60.8 | **✔** |  |  | **✔** |  |  |
|  | Cai et al. (2021) | China | DC: 85; VC: 41 | DC: 60 ± 3.4; VC: 60 ± 3.2 | DC: 64.7; VC: 51.2 | **✔** |  |  | **✔** |  |  |
|  | Cheng P et al. (2021) | China | 53 | 60 ± 16 | 67.9 | **✔** |  |  | **✔** |  |  |
|  | Ebell et al. (2021) | United States | 1442 | 61 ± 25 | 51.6 | **✔** |  |  | **✔** |  |  |
|  | Fan et al. (2021) | China | DC: 96; VC: 43 | DC: 63.5; VC: 63.4 | DC: 51; VC: 69.8 | **✔** |  |  | **✔** |  |  |
|  | Gue et al. (2020) | United Kingdom | 316 | S: 67 ± 4.8; NS: 81 ± 2.6 | S: 54.7; NS: 72.2 | **✔** |  |  | **✔** |  |  |
|  | Hajifathalian et al. (2020) | United States | 664 | 64 ± 17 | 63 | **✔** |  |  | **✔** |  |  |
|  | Hu Hai et al. (2020) | China | 105 | 60.8 ± 16.3 | 50.9 | **✔** |  |  | **✔** |  |  |
|  | Hu C et al. (2021) | China | 183 | S: 60.5 ± 13.2; NS: 68.4 ± 9.9 | S: 49.6; NS: 73.5 | **✔** |  |  | **✔** |  |  |
|  | Hu, H et al. (2020) | China | 40 | 52.7 ± 5.7 | 60 | **✔** |  |  | **✔** |  |  |
|  | Jiang et al. (2021) | China | DC: 1717; VC:188 | 63 ± 2.6 | DC: 50.9; VC: 52.1 | **✔** |  |  | **✔** |  |  |
|  | King et al. (2020) | United States | 13323 | 62 ± 2.9 | 91 | **✔** |  |  | **✔** |  |  |
|  | Knight et al. (2020) | United Kingdom | 35463 | 72 ± 2.9 | 58.3 | **✔** |  |  | **✔** |  |  |
|  | Laguna-Goya et al. (2021) | Spain | 501 | 52 ± 2.6 | 63.3 | **✔** |  |  | **✔** |  |  |
|  | Li J et al. (2020) | China | 2039 (DC:1008; VC: 1031) | DC: 54.7 ± 3.2; VC: 62 ± 2.7 | DC: 56.4; VC: 52.2 | **✔** |  |  | **✔** |  |  |
|  | Li Li et al. (2021) | China | 4086 | 60.2 ± 2.6 | 50 | **✔** |  |  | **✔** |  |  |
|  | Liu Q et al. (2020) | China | 336 | 62.5 ± 3.1 | 50.3 | **✔** |  |  | **✔** |  |  |
|  | Liu H et al. (2021) | China, Italy | 12759 | 58 ± 2.8 | 48.3 | **✔** |  |  | **✔** |  |  |
|  | Liu S et al. (2020) | China | 127 | 61.25 ± 15.5 | 70.1 |  |  | **✔** | **✔** |  |  |
|  | López-Escobar et al. (2021) | Spain | 1955 | 68.75 ± 3.3 | 60.1 |  | **✔** |  | **✔** |  |  |
|  | Max et al. (2020) | China | 523 | 43.5 ± 3.6 | 55.3 |  | **✔** |  | **✔** |  |  |
|  | Ma et al. (2020) | China | 262 | 66.55 ± 2.5 | 54.6 | **✔** |  |  | **✔** |  |  |
|  | Magro et al. (2021) | Italy | 2191 | 66 ± 3.04 | 69.4 | **✔** |  |  | **✔** |  |  |
|  | Pan et al. (2020) | China | 120 | 62.16 | 58.3 | **✔** |  |  | **✔** |  |  |
|  | Pigoga et al. (2021) | Sudan | 467 | 65 ± 19 | 70.2 |  | **✔** |  | **✔** |  |  |
|  | Quanjel et al. (2020) | Netherlands | 305 | 62.7 | 62 | **✔** |  |  |  |  | **✔** |
|  | Satici et al. (2020) | Turkey | 681 | 56.9 ± 15.7 | 51 | **✔** |  |  | **✔** |  |  |
|  | Selcuk et al. (2021) | Turkey | 231 | 60 | 55.8 | **✔** |  |  | **✔** |  |  |
|  | Shang et al. (2020) | China | 452 (DC: 113; VC: 339) | DC: 65 ± 2.8 | DC: 64.6 | **✔** |  |  | **✔** |  |  |
|  | Soto-Mota et al. (2020) | Mexico | 400 | NR | S: 66.5; NS: 73.5 | **✔** |  |  | **✔** |  |  |
|  | Stachel et al. (2021) | United States | 3395 | NR | NR | **✔** |  |  | **✔** |  |  |
|  | Tanboga et al. (2021) | Turkey | 60.98 | 49 ± 3.1 | 5300% | **✔** |  |  | **✔** |  |  |
|  | Tezza et al. (2021) | Italy | 341 | 73 ± 18,6 | 57.0 | **✔** |  |  | **✔** |  |  |
|  | Wang X et al. (2020) | China | 131 | 63.75 ± 2.8 | 42.7 | **✔** |  |  | **✔** |  |  |
|  | Wang et al. (2020) | China | 235 | 70.6 ± 8.0 | 55.7 |  |  |  | **✔** |  |  |
|  | Wenig et al. (2020) | China | 301 | 58 ± 4.9 | 43.5 | **✔** |  |  | **✔** |  |  |
|  | Wongvibulsin et al. (2021) | United States | 3494 | 60.5 ± 3.9 | 51.0 | **✔** |  | **✔** | **✔** |  |  |
|  | Yang et al. (2021) | China | 2541 (DC: 124; VC1:71; VC2: 76) | NR | DC: 55.7; VC1:59.2; VC2: 54 | **✔** |  |  | **✔** |  |  |
|  | Yuan Y et al. (2020) | China | 1479 | 60.6 ± 3.6 | 50.9 | **✔** |  |  | **✔** |  |  |
|  | Zayed et al. (2021) | Egypt | 142 | 41.2 ± 9.9 | 81.7 | **✔** |  |  | **✔** |  |  |
|  | Zeng et al. (2021) | China | 351 | 53 ± 4.8 | 53.8 | **✔** |  |  | **✔** |  |  |
|  | Zhang S et al. (2020) | China | 828 | 61 ± 2.8 | 53.9 | **✔** |  |  | **✔** |  |  |
|  | Zou X et al. (2020) | China | 154 | 60.7 ± 13 | 43.5 | **✔** |  |  | **✔** |  |  |
|  | Ahirwar et al. (2022) | India | 400 (S: 382; NS: 18) | S: 42 ± 17; NS: 65.7 ± 13 | 62.3 |  | **✔** |  | **✔** |  |  |
|  | Aletreby et al. (2022) | Saudi Arabia | 1493 (S:926; NS:567) | S: 51.1±13.6; NS: 56.8±14.4 | S: 76.6; NS: 76 | **✔** |  |  |  | **✔** |  |
|  | Churpek et al. (2021) | United States | 5075 (NS: 1846; S: 3229) | NS: 67±2.4; S: 58.7±2.6 | NS: 66; S:61 | **✔** |  |  |  | **✔** |  |
|  | Raschke et al. (2022) | United States | 2440 (DC: 1221; VC 1219) | DC: 65.7 ± 2.6; VC: 65.7 ± 2.9 | 61.6 |  | **✔** |  |  | **✔** |  |
|  | Reina et al. (2022) | Spain | 1201 | 49.53 ± 24.9 | 44.9 | **✔** |  |  | **✔** |  |  |
|  | Riley et al. (2022) | United States | 426 | 64.4 | 56.6 |  | **✔** |  | **✔** |  |  |
|  | Shanbehzadeh et al. (2022) | Iran | 1710 | 61.62 ± 17.6 | 61.6 |  |  |  | **✔** |  |  |
|  | Singh et al. (2022) | India | 131 | 54 ± 14 | 74.8 | **✔** |  |  | **✔** |  |  |
|  | Surme et al. (2022) | Turkey | 1013 | 60.5 ± 14.4 | 57.4 | **✔** |  |  | **✔** |  |  |
|  | Van de Leur et al. (2022) | Netherlands | 882 | 67.0 ± 14 | 65 | **NR** | **NR** | **NR** | **✔** |  |  |
|  | Vieira et al. (2022) | Brazil | 11 | 67 ± 17 | 58.5 |  | **✔** |  | **✔** |  |  |
|  | Webb et al. (2022) | Multi-national | 22.816 | 40.4 ± 16.5 | 49.9 | **✔** |  |  | **✔** |  |  |
|  | Wirth et al. (2022) | Switzerland | 546 | 68.2 ± 3.8 | 63.2 | **✔** |  |  | **✔** |  |  |
|  | Yilmaz et al. (2021) | Turkey | 101 | 75.9 ± 9.3 | 54.5 | **✔** |  |  | **✔** |  |  |
|  | Leoni et al. (2021) | Italy | 242 | 63.7 ± 2.7 | 81 | **✔** |  |  |  | **✔** |  |
|  | Marincu et al. (2021) | Romania | DC: 510 (NS:310; S: 200); VC: 541 | 67 | NS: 61.9; S: 45.5 | **✔** |  |  | **✔** |  |  |
|  | Ottenhoff et al. (2021) | Netherlands | 2273 | 68.5 ± 2.9 | 62.3 | **✔** |  |  | **✔** |  |  |
|  | Rozenbaum et al. (2021) | United States | 764 | 64.3 ± 3.7 | 52.1 | **✔** |  |  | **✔** |  |  |
|  | Ruscica et al. (2021) | Italy | 97 | 61 ± 1 | 70 | **✔** |  |  | **✔** |  |  |
|  | Valente Silva et al. (2021) | Portugal | 300 | 71 ± 3.8 | 59 | **✔** |  |  | **✔** |  |  |
|  | Cui et al. (2022) | China | 437 | 61 ± 12.7 | 54.9 | **✔** |  |  | **✔** |  |  |
|  | Ergenç et al. (2022) | Turkey | 105 (mild:51, critical: 54) | Mild:54 ±19.8; critical = 69.2 ±11.1 | Mild: 39; critical: 61 | **✔** |  |  | **✔** |  |  |
|  | Falandry et al. (2022) | France | 231 | 73.1 ± 7.4 | 75.3 | **✔** |  |  |  | **✔** |  |
|  | Marcolino et al. (2021) | Brazil & Spain | 5888 | 61 ± 18.3 | 53.8 | **✔** |  |  | **✔** |  |  |
|  | Alkaabi et al. (2021) | UAE | 1542 | NS: 56.7 ± 13.3; S: 47.8 ± 12.1 | 85.7 | **✔** |  |  |  | **✔** |  |
|  | Mahdavi et al. (2021) | Iran | 492 | 62 ± 18.5 | 66.1 |  | **✔** |  | **✔** |  |  |
|  | Murri et al (2021) | Italy | 2384 | DC (S: 64 ± 15.4; NS:84± 10.1)  VC (S: 65 ± 18.5; NS: 80 ± 11.3) | DC: 61.4; VC: 54.5 | **✔** |  |  | **✔** |  |  |
|  | Heber et al (2021) | Austria | 919 | S: 58 ± 20.8; NS: 82.3 ± 9 | S: 60; NS: 64 | **✔** |  |  | **✔** |  |  |
|  | Kilercik et al. (2021) | Turkey | 97 | S: 51.7 ± 14.5; NS: 71.7 ± 14.7 | S: 62.2; NS: 80 |  | **✔** |  | **✔** |  |  |
|  | Al Mutair et al. (2021) | Saudi Arabia | 73 | S: 49 ± 11.1; NS: 58.3 ± 6.9 | S: 95.7; NS: 86 | **✔** |  |  |  | **✔** |  |
|  | Yang et al. (2021) | China | 2169 | S: 60 ± 14.1; NS: 73.7 ±11.3 | S: 47; NS:65 | **✔** |  |  |  |  |  |
|  | Vicka et al. (2021) | Lithuania | 249 | S: 57.7 ±13.6; NS: 66.4 ±11.1 | S: 64.4, NS:61.2 | **✔** |  |  |  | **✔** |  |
|  | Jain et al. (2021) | India | 735 | S: 59 ±14.9; NS: 66.2 ±12.7 | S: 72; NS:75.71 | **✔** |  |  |  |  |  |
|  | Rahman et al. (2021) | China & Bangladesh | Chinese arm; 375; Bangladeshi arm: 103 | Chinese arm (S: 50.2 ±15; NS: 68.8 ±11.8; Bangladeshi arm (S: 39.9 ± 12.92; NS: 57.9±13.3) | Chinese arm (S: 49; NS: 72; Bangladeshi arm (S:49; NS: 59.5) | **NR** | **NR** | **NR** | **✔** |  |  |
|  | Ponce et al. (2021) | 57 cities in 12 countries from Latin America | 870 (DC: 697; VC:173) | S: 59.2±16.1; NS: 65.1±13.8 | S: 65; NS: 71 | **✔** |  |  | **✔** |  |  |
|  | Haji Aghajani et al. (2021) | Iran | 893 (DC:714; VC:179) | S:59±17.8; NS:72.3±16.4 | S:52.9; NS: 60.9 | **✔** |  |  | **✔** |  |  |
|  | Garrafa et al. (2021) | Italy | 2782 (DC:1474; VC:632) | S: 64.7±14.5; NS: 76.3±9.4 | S:62.5; NS:68.9 |  | **✔** |  | **✔** |  |  |
|  | He et al. (2021) | China | 3623 (DC:2119; VC:1504) | S: 59.3±13.3; NS: 69.8±12.1 | S: 50.7; NS: 63.6 |  | **✔** |  | **✔** |  |  |
|  | Moghaddam-Tabrizi et al. (2021) | Iran | 401 | 57.5±18.3 | S: 50.9; NS: 61.2 |  | **✔** |  | **✔** |  |  |
|  | Nishikimi et al. (2021) | United States | 1945 (DC: 1389, validation: 556) | S: 62.9±13.7; NS: 68.8±12.4 | S:65.7; NS: 71.7 | **✔** |  |  |  | **✔** |  |
|  | Sosa et al. (2021) | Argentina | 59 | 66.5 ±15 | 73 |  | **✔** |  |  | **✔** |  |
|  | Banoei et al. (2021) | United States | 250 | S: 60.6±16.8; NS: 78.1±10.6 | S: 53.8; NS: 70.9 | **✔** |  |  | **✔** |  |  |
|  | Riva et al. (2021) | Italy | 87 | 66 ±16 | 52.9 | **✔** |  |  | **✔** |  |  |
|  | Rahman et al. (2021) - | United States & China | DC:384; VC:375 | CD | NR | **✔** |  |  | **✔** |  |  |
|  | Timpau et al. (2021) | Romania | 150 | S: 61.2 ±13.5; NS: 69 ±12.5 | S:70; NS: 57 | **✔** |  |  | **✔** |  |  |
|  | Martín-Rodríguez et al. (2021) | Spain | 2320 | S: 75.7±16.3 AND NS: 84.3±8.9 | S:50.8; NS: 46.4 |  | **✔** |  | **✔** |  |  |
|  | Kar et al. (2021) | India | 2370 (DC:1393; VC:977) | DC (S:47.2±16.5; NS - 61.1±17.5)  VC (S:53.4±4.6; NS - 67.52±13.3) | DC (NS: 78)  VC (NS: 85) |  | **✔** |  | **✔** |  |  |
|  | Yu et al. (2021) | China | 1229 | S: 60 ±14.1; NS: 73 ±10.6 | S: 46.5; NS: 71.2 | **✔** |  |  | **✔** |  |  |
|  | Zelikovna-Golukhova et al. (2022) | Russia | 110 | S: 57; NS 58.8 | 57.3 | **✔** |  |  | **✔** |  |  |
|  | Alhamar et al. (2022) | Kuwait | 417 (S:357; NS:60) | S:43.8 ± 17.5; NS:53.6 ± 12.2 | S:58.3; NS:90 | **✔** |  |  |  | **✔** |  |
|  | Jamshidi et al. (2022) | Iran | 23,749 | 52 ± 43 | 53.4 |  | **✔** |  | **✔** |  |  |
|  | Araiza et al. (2021) | United States | 235 (S:197; NS:38) | 56 ±15.6 | 54.9 | **✔** |  |  |  | **✔** |  |
|  | Alkhasawneh et al. (2021) | Jordan | 193 (S: 99; NS:94) | S:58.5±9.9; NS: 58.1±10 | S:68.7; NS:63.8 | **NR** | **NR** | **NR** |  | **✔** |  |
|  | Bae et al. (2021) | United States | COVID-19 positive: 515; Chest X-rays: 530 | Center 1: 57 ± 17; Center 2: 59 ±14.3 | 52.6 | **✔** |  |  | **✔** |  |  |
|  | Gordon et al. (2022) | United States | 6802 | 64 ± 3.1 | 56.2 | **✔** |  |  | **✔** |  |  |
|  | Gutierrez-Camacho et al. (2022) | Mexico | 377 | Range: 18-93 | 48.9 |  | **✔** |  | **✔** |  |  |
|  | Hassan et al. (2022) | Italy & Netherlands | Italian cohort:1028; Dutch cohort: 432 | Italian cohort: 66; Dutch cohort: 65 | Italian cohort: 63; Dutch cohort 61 | **✔** |  |  | **✔** |  |  |
|  | Hippisley-Cox et al. (2021) | England | 6952440 | 52.5 ± 17.7 | 47.8 | **NR** | **NR** | **NR** |  |  | **✔** |
|  | Hohl et al. (2022) | Canada | 8761 | 54.7±19.8 | 52.2 |  | **✔** |  | **✔** |  |  |
|  | Huang Chun‑Yen et al. (2022) | Taiwan | 228 | 61.8 ± 3.6 | 54.4 | **✔** |  |  | **✔** |  |  |
|  | Jalalvand et al. (2022) | Iran | 226 | 57.8±16.6 | 52.7 |  | **✔** |  | **✔** |  |  |
|  | Khari et al. (2022) | Iran | 225 | 63.27±14.9 | 56.9 | **✔** |  |  |  | **✔** |  |
|  | Kibar Akilli et al. (2022) | Turkey | 1511 | 60.1 ± 14.7 | 58.2 | **✔** |  |  | **✔** |  |  |
|  | Klen et al. (2022) | Spain, United States, Latin America | 15902 | 67.5 ± 2.9 | 57.4 | **✔** |  |  | **✔** |  |  |
|  | Kucuk, Berkay et al. (2022) | Turkey | 322 | 69.1 ± 14.4 | 62.1 | **✔** (+CT) |  |  |  | **✔** |  |
|  | Laino et al. (2022) | Italy | 1135 | 69.5 ± 3.3 | 64 | **✔** |  |  | **✔** |  |  |
|  | Li G et al. (2021) | China | 540 | 54.6 ± 16 | 48.5 | **✔** |  |  | **✔** |  |  |
|  | Li Y et al. (2022) | United States | Center 1: 1673; C2: 558; C3: 1815; C4: 1570 | C1: 59±45.7; C2: 61±50.7; C3: 59± 44.7; C4: 59± 44.7 | C 1:51; C 2:49; C 3:49; C 4: 55 | **✔** |  |  | **✔** |  |  |
|  | Lyons et al. (2022) | United Kingdom | 1956760 | 50.8 ± 18.7 | 49.5 | **NR** | **NR** | **NR** |  |  | **✔** |
|  | Martın-Rodrıguez et al. (2022) | Spain | 2961 | 78 ± 3.1 | 50.8 | **✔** |  |  | **✔** |  |  |
|  | Morello et al. (2022) | Italy | DC: 838; VC:521 | 51± 17 | 52.6 | **NR** |  |  |  |  | **✔** |
|  | Moulaei et al. (2022) | Iran | 1500 | 57.25 ± 17.8 | 55.7 | **✔** |  |  | **✔** |  |  |
|  | Mousavi et al. (2021) | Iran | 4542 | 57.94 ± 17.3 | 58.9 | **✔** |  |  | **✔** |  |  |
|  | Munoz et al. (2022) | Columbia | 272 | 61.1 ± 3.6 | 58.8 | **✔(**Ag) |  |  | **✔** |  |  |
|  | Najafi et al. (2021) | Iran | 659 | 60.7 ± 16.4 | 56 | **✔ (CT)** |  |  | **✔** |  |  |
|  | Naser et al. (2021) | Bahrain | 353 | 55.8 ±15.7 | 100 | **✔** |  |  | **✔** |  |  |
|  | Ocho et al. (2022) | Japan | 206 (S:185; NS: 21) | 65 ±5.3 | 59.2 | **NR** | **NR** | **NR** | **✔** |  |  |
|  | Özdemir et al. (2021) | Turkey | 281 (S:146; NS: 135) | S: 68.1±10.8; NS:73±11 | S: 47.3, NS: 55.6 | **✔** |  |  |  | **✔** |  |
|  | Özdemir et al. (2022) | Turkey | 122 (S:109; NS: 13) | 72±2.3 | 50.8 |  | **✔** |  | **✔** |  |  |
|  | Ozger et al. (2021) | Turkey | 37 (S:29; NS: 8) | 61.0±5.2 | 64.9 | **✔** |  |  | **✔** |  |  |
|  | Pasculli et al. (2022) | Italy | 283 (S: 244; NS:39) | 64.5±4.2 | 56.9 |  | **✔** |  | **✔** |  |  |
|  | Plecko et al. (2022) | Netherlands | 2417 (DC: 1480; VC: 937) | DC: 64.8±2.2; VC:64.8±2.3 | 74 | **NR** | **NR** | **NR** |  | **✔** |  |
|  | Asaduzzaman et al. (2022) | Bangladesh | 442 | 60±14 | 65.8 | **NR** |  |  | **✔** |  |  |
|  | Aygun et al. (2022) | Turkey | 339 | 55 ±37.2 | 54.9 | **✔** |  |  | **✔** |  |  |
|  | Ayvat et al. (2022) | Turkey | 229 | 66.9 ± 11.5 | 69.9 | **✔** |  |  |  | **✔** |  |
|  | Bakpour et al. (2022) | United States | 682 (DC: 439; VC:189) | 60 ±16 | 58.1 | **✔** |  |  | **✔** |  |  |
|  | Bartoszko et al. (2022) | Canada | 127 | 58 ± 14 | 71 | **✔** |  |  |  | **✔** |  |
|  | Beigmohammadi et al. (2022) | Iran | 204 | S: 61.4 ± 14.7; NS:63.3 ± 12.3 | 63.7 | **✔** |  | **✔** |  | **✔** |  |
|  | Bengelloun et al. (2022) | Spain | 2844 | 67.3 ± 16.5 | 55.1 | **✔**(Ag) |  |  | **✔** |  |  |
|  | Besutti et al. (2022) | Italy | 308 | 64.6 ± 17.1 | 61.4 |  | **✔** |  | **✔** |  |  |
|  | Bezerra et al. (2022) | Brazil | 58 | 56.6 ± 15.8 | 58.6 | **✔** |  |  |  | **✔** |  |
|  | Bodolea et al. (2022) | Romania | 90 | 64 ± 39.3 | 58.9 | **✔** |  |  |  | **✔** |  |
|  | Bradley et al. (2022) | United States | 632 | NS: 72.3±12.7; S: 58.7±19.3 | NS:57; S:44 | **✔** |  |  | **✔** |  |  |
|  | Chikhalkar et al. (2022) | India | 814 | 44.5 | 69.3 | **✔** |  |  | **✔** |  |  |
|  | Chou et al. (2022) | United States | 1678 | 55.1 ± 19.2 | 50.1 | **✔** |  |  | **✔** |  |  |
|  | Cidade et al. (2022) | Portugal | 118 | 63 ± 13.1 | 76.3 | **✔** |  |  |  | **✔** |  |
|  | Citu Cosmin et al. (2022) | Romania | 133 | 65 ± 21 | 51.1 | **✔** |  |  | **✔** |  |  |
|  | Citu Cosmin et al. (2022) | Romania | 82 | 66.5 ± 17 | 48.8 | **✔** |  |  | **✔** |  |  |
|  | Comoglu et al. (2022) | Turkey | 1559 | 47.1±17.5 | 53.6 | **✔** |  |  | **✔** |  |  |
|  | Lee DS et al. (2021) | Canada | 64733 | 85± (9.6 | 31.8 | **✔** |  |  | **✔** |  | **✔** |
|  | **Outcome: Severity or critical illness** | | | | | | | | | | |
|  | Zhou Y et al. (2020) | China | 366 | 42 ± 3.2 | 56.6 | **✔** |  |  | **✔** |  |  |
|  | Ageno et al. (2021) | Italy | 610 (DC: 335; VC: 275) | DC:72; VC:65 | DC (NS: 56.8; S: 60.7)  VC:(NS: 60.9; S: 67.6) | **✔** |  |  | **✔** |  |  |
|  | Bello-Chavolla et al. (2020) | Mexico | 3007 | NS: 44 ± 4; S: 56 ± 2.7 | NS: 50.5; S:70.1 | **✔** |  |  |  |  |  |
|  | Bennouar et al. (2020) | Algeria | 330 | 66.6 ± 8.9 | 62.4 | **✔** |  |  | **✔** |  |  |
|  | Bennouar et al. (2021) | Algeria | 576 (DC: 329; VC: 247) | DC: 66.6 ± 8.9; VC: 65.1 ± 10.6 | DC: 62.3; VC:67.6 | **NR** |  |  | **✔** |  |  |
|  | Boero et al. (2021) | Italy | 274 | 67.7 ± 14.4 | 69.0 | **✔** |  |  | **✔** |  |  |
|  | Chen et al. (2021) | China | 582 | 50 ± 4.3 | 49.1 | **✔** |  | **✔** |  |  |  |
|  | De Socio et al. (2021) | Italy | 121 | 64.5 ± 13.4 | 65.3 | **✔** |  |  | **✔** |  |  |
|  | Fernandes et al. (2021) | Brazil & Spain | 1040 | 51.7 ± 18.9 | 53.0 | **✔** |  |  | **✔** |  |  |
|  | Gao et al. (2021) | China | 450 (DC: 111; internal VC: 111; external VC: 228 | DC: 62.6 ± 2.9; internal VC:62.6 ± 3.1; external VC: 61.5 | DC: 41.4; internal VC: 52.2; external VC: 49.1 | **✔** |  |  | **✔** |  |  |
|  | Li L et al. (2021) | China | 124 (DC: 84; VC:40) | DC: 63.4 ± 1.5 | 57 | **✔** |  |  | **✔** |  |  |
|  | Li S et al. (2021) | China | 53 | 62.9 ± 5.2 | 50.9 | **✔** |  |  | **✔** |  |  |
|  | Liang et al. (2020) | China | DC: 1590; VC: 710 | DC: 48.9 ± 15.7; VC: 48.2 ± 15.2 | DDC: 57.3; VC: 53.8 | **✔** |  |  | **✔** |  |  |
|  | Liu Q et al. (2021) | China | 158 | NS: 58.2 ± 14.4; S: 61.5 ± 11.7 | NS: 52 S: 71.4 | **✔** |  |  | **✔** |  |  |
|  | Liu, J et al. (2020) | China | 2106 | 67.5 ± 1.4 | NR | **✔** |  |  | **✔** |  |  |
|  | Marcos et al. (2021) | Spain | 918 | 72.8 ± 14.5 | 57.8 | **✔** |  |  | **✔** |  |  |
|  | Myrstad et al. (2020) | Norway | 66 | 67.9 | 58 | **✔** |  |  | **✔** |  |  |
|  | Prower et al. (2021) | England | 708 | 62.2 ± 18.1 | 57.6 | **✔** |  |  | **✔** |  |  |
|  | Purkayastha et al. (2021) | China & United States | 981 (DC: 687; VC: 97; test: 197) | (DC: 47.7±14.5; VC: 42.2 ±17.1; test: 46.2±15.9) | DC: 51; VC: 51; test: 52 | **✔** |  |  | **✔** |  |  |
|  | Schalekamp et al. (2021) | Netherlands | 356 | 69 ± 12 | 67 | **✔** |  |  |  |  |  |
|  | Schöning et al. (2021) | Switzerland | DC: 198; DC: 459 | DC (S: 65 ± 6; NS:53 ± 79)  VC (S: 69 ± 3; NS: 61 ± 5) | DC (S: 82.5; NS:48.9)  VC (S: 68.6; NS: 57.5) | **✔** |  |  | **✔** |  | **✔** |
|  | Shi Y et al. (2021) | United States | 257 | NR | NR | **✔** |  |  | **✔** |  |  |
|  | Su Y et al. (2020) | China | 116 | 62 ± 4.1 | 47.4 | **✔** |  |  | **✔** |  |  |
|  | Tu et al. (2021) | China | 202 | 44.7 ± 4.9 | 44.6 | **✔** |  |  | **✔** |  |  |
|  | Ucan et al. (2021) | Turkey | 298 | 61.8 ± 20. | 49.6 |  | **✔** |  | **✔** |  |  |
|  | Woo et al. (2021) | United States | 415 | 66 ± 5.4 | 55.2 | **✔** |  |  | **✔** |  |  |
|  | Wu et al. (2020) | China | 299 | 61.5 ± 3.8 | 45.8 | **✔** |  |  | **✔** |  |  |
|  | Xiao et al. (2020) | China | 408 | 47 ± 3.7 | 50.2 | **✔** |  |  | **✔** |  |  |
|  | Xu F et al. (2021) | China | 269 | 62.7 ± 0.6 | 51.0 | **✔** |  |  | **✔** |  |  |
|  | Xu J et al. (2021) | China | 98 | 47 ± 5.5 | 45.9 | **✔** |  | **✔** | **✔** |  |  |
|  | Yao et al. (2021) | China | 590 (DC 285; internal VC: 127; prospective VC: 178) | NR | DC:53.3; internal VC:50.4; prospective VC:51.7 |  |  | **✔** | **✔** |  |  |
|  | Yu Y et al. (2020) | China | 3265 | 58 ± 2.9 | 47.3 | **✔** |  |  | **✔** |  |  |
|  | Zhang B et al. (2020) | China | 233 | 55.4 | 55.4 | **✔** |  |  | **✔** |  |  |
|  | Assal et al. (2022) | Egypt | 175 | 59 | 77.0 | **✔** |  |  | **✔** |  |  |
|  | Shalmon et al. (2022) | United States | 80 | Median: 63.5 | 63 | **✔** |  |  | **✔** |  |  |
|  | Shankar et al. (2022) | India | 608 | NR | NR | **NR** |  |  | **✔** |  |  |
|  | Shi et al. (2022) | China | 260 (DC: 156; VC: 104) | DC (NS: 52.3; S:69.2)  VC (NS: 46.5; S:77.8) | DC (NS: 52.3; S:69.2)  VC (NS: 46.5; S:77.8) | **✔** |  |  | **✔** |  |  |
|  | Tang et al. (2022) | China | 314 | 61.5 ± 2.93 | S:54; NS: 44 | **✔** |  |  | **✔** |  |  |
|  | Vela et al. (2022) | Spain | DC: 7704171; VC: 218329 | DC: 42.2 ± 3.3; VC: 40.2 ± 3.8 | NR | **✔** |  |  |  | **✔** |  |
|  | Wong et al. (2021) | United Kingdom | 7846 | Range: 50-87 | NR | **NR** |  |  | **✔** |  |  |
|  | Xiong et al. (2022) | China | 287 | 59.2 ± 3.33 | 56.4 | **✔** |  |  | **✔** |  |  |
|  | Zhang et al. (2022) | China | 104 | 55.5 ± 13.9 | 51.9 | **✔** |  |  | **✔** |  |  |
|  | Zhao et al. (2022) | China | 116 (DC:65; VC: 51) | VC:48.2 ± 15.2 | VC: 74.5 | **✔** |  |  | **✔** |  |  |
|  | Gómez, (2021) | Spain | 540 | 64.2 ± 12.6 | 58.15 | **✔** |  |  |  | **✔** |  |
|  | Monterde et al. (2021) | Spain | 4607 | 60.5 ± 19.4 | 56.1 | **NR** |  |  | **✔** |  |  |
|  | Muto et al. (2021) | Japan | 300 | 58.75 ± 5.75 | 62.3 | **✔** |  |  | **✔** |  |  |
|  | Rinderknecht et al (2021) | United States | 15753 | 48.6 ± 19.4 | 43.1 | **✔** |  |  | **✔** |  |  |
|  | Bennett et al. (2021) | United States | 174568 | 44.4± 18.6 | 46.4 |  | **✔** |  |  | **✔** |  |
|  | Sengel et al. (2021) | Turkey | 256 | 54.8 ± 12.6 | 47.7 | **✔** |  |  | **✔** |  |  |
|  | Li et al. (2021) | China | 495 | 51± 19 | 49.7 | **✔** |  |  | **✔** |  |  |
|  | Huang Jiana et al. (2021) | China | 98 | 46.4± 2 | 47 | **✔** |  |  | **✔** |  |  |
|  | Ma et al. (2021) | China | 296 | 59.5± 14.4 | 54.4 | **✔** |  |  | **✔** |  |  |
|  | An et al. (2022) | China | 231 | 44.3 ± 12.7 | NR | **✔** |  |  | **✔** |  |  |
|  | Gurusamy et al. (2021) | India | 1100 | 38.2 ± 17.3 | 62 | **✔** |  |  | **✔** |  |  |
|  | Haimovich et al. (2020) | United States | 1172 | 66.2 ± 16.8 | 52.6 | **✔** |  |  | **✔** |  |  |
|  | Han et al. (2022) | China | 117 | 61.5 ± 4.1 | 57.3 | **✔** |  |  | **✔** |  |  |
|  | Jiang et al. (2022) | China | 479 | 46.4±14.2 | 50 | **✔** |  |  | **✔** |  |  |
|  | Lee et al. (2022) | South Korea | 561 | 56±3.9 | NR | **✔** |  |  | **✔** |  |  |
|  | Leyderman et al. (2021) | Russia | 55 | 57.2 ± 12.7 | 56.3 |  |  | **✔** |  | **✔** |  |
|  | Liu et al. (2021) | China | 122 | 60.2 ± 4.5 | 59 | **✔** |  |  | **✔** |  |  |
|  | Nadasdi et al. (2022) | Hungary | 102 | 64.8 | 45.1 | **NR** |  |  | **✔** |  |  |
|  | Nuevo‐Ortega et al. (2022) | Spain | 404 | 61 | 59 | **✔**(Ag) |  |  | **✔** |  |  |
|  | Patel et al. (2021) | United States | 129 | 60.8 ± 13.6 | 55 | **✔** |  |  | **✔** |  |  |
|  | Peng et al. (2022) | China | 239 | 45.0 ± 15.9 | 52.7 | **✔** |  |  | **✔** |  |  |
|  | Chang et al. (2022) | China | 1059 | NR | 43.4 | **NR** | **NR** | **NR** | **✔** |  |  |
|  | Chen et al. (2022) | China | 390 | 55.3 ± 1 | 54.1 | **✔**(Ag) |  |  | **✔** |  |  |
|  | **Outcome: Mortality and ICU admission** | | | | | | | | | | |
|  | Ak et al. (2021) | Turkey | 341 | 58.2 ± 17.2 | 48.3 |  | **✔** |  | **✔** |  |  |
|  | Arnold et al. (2021) | United Kingdom | 187 | 58.75 ± 4.9 | 54.0 | **✔** |  |  | **✔** |  |  |
|  | Covino et al. (2020) | Italy | 334 | 66 ± 4.1 | 64.4 | **✔** |  |  | **✔** |  |  |
|  | García Clemente et al. (2020) | Spain | 249 | 65.6 ± 16.1 | 57.4 | **✔** |  |  | **✔** |  |  |
|  | Kurt et al. (2021) | Turkey | 464 | 62.4 ± 16.7 | 52.8 | **✔** |  |  | **✔** |  |  |
|  | Lazar Neto et al. (2021) | Brazil and Spain | 1363 | 61.05 ± 16 | 59.2 | **✔** |  | **✔** | **✔** |  |  |
|  | Levine et al. (2021) | United States | 1326 (DC: 1014; VC: 312) | DC: 58 | 56.0 | **✔** |  |  | **✔** |  |  |
|  | Li X et al. (2020) | United States | 1108 (ICU: 271; non-ICU:837) | ICU: 59.5 ± 3.8; Non-ICU: 62.5 ± 4.0 | ICU: 67.5; non-ICU:54 | **✔** |  |  |  | **✔** |  |
|  | Pokeerbux et al. (2021) | France | 202 | 65 ± 4.7 | 61.4 | **✔** |  |  | **✔** |  |  |
|  | Ponsford et al. (2021) | United Kingdom | 391 | 67± 3.4 | 52.4 | **✔** |  |  | **✔** |  |  |
|  | Rasyid et al. (2021) | Indonesia | 295 | 47.4 ± 15.3 | 70.3 | **✔** |  |  | **✔** |  |  |
|  | Rodriquez-Nava et al. (2021) | United States | 313 | 68.3 ± 3.3 | 58.1 | **✔** |  |  | **✔** |  |  |
|  | Shi, S et al. (2021) | China | 87 | 57.5 ± 13.4 | 56.3 | **✔** |  |  | **✔** |  |  |
|  | Van Dam et al. (2021) | Netherlands | 642 | 71.5 ± 2.9 | 63.4 | **✔** |  |  | **✔** |  |  |
|  | Zhao Z et al. (2020) | United States | 641 | 60 ± 3.5 | 59.9 | **✔** |  |  | **✔** |  |  |
|  | Adderley et al. (2022) | United Kingdom | 1040 | 68.2 ± 17.7 | 57.0 |  | **✔** |  | **✔** |  |  |
|  | Aguadero et al. (2021) | Switzerland | 386 | 66 ± 4.7 | 61.0 | **✔** |  |  | **✔** |  |  |
|  | Regolo et al. (2022) | Italy | 411 | 72.2 ± 0.8 | 57.7 | **✔** |  |  | **✔** |  |  |
|  | Rizzi et al. (2022) | Italy | 139 | 63.9 ± 3.00 | 61.9 |  | **✔** |  | **✔** |  |  |
|  | Usul et al. (2021) | Turkey | 211 | 50.2 ± 18.8 | 51.2 | **✔** |  |  |  | **✔** |  |
|  | Wilfong et al. (2021) | United States | 128 | 56.3 ± 4.33 | 58.6 |  | **✔** |  | **✔** |  |  |
|  | Aznar-Gimeno et al. (2021) | Spain | 3623 | DC: 73.5 ± 17.8; VC (severity):82.8 ± 2.7 | NR | **✔** |  |  | **✔** |  |  |
|  | Subudhi et al. (2021) | United States | 5308 | Median: 55 | 51.3 | **✔** |  |  | **✔** |  |  |
|  | Ak et al. (2021) | Turkey | 364 | 68 ± 18.1 | 53.8 |  | **✔** |  | **✔** |  |  |
|  | Klaveren et al. (2021) | Netherlands | DC: 5831; VC: 3235 | 69.7± 16.3 | 58 |  |  | **✔** | **✔** |  |  |
|  | Ganesan et al. (2021) | India | 147 | S: 52.7 ± 13.6; NS: 58 ± 13.7 | 64.6 | **✔** |  |  |  | **✔** |  |
|  | Ahmed et al. (2022) | United Kingdom | 2387 | 71 ± 17.8 | 54.0 | **✔** |  |  | **✔** |  |  |
|  | Hormanstorfer et al. (2021) | Argentina | 335 | 44.8 ± 17.2 | 52 | **✔** |  |  | **✔** |  |  |
|  | Zahedin Kheyri et al. (2022) | Iran | 963 | 56.7 ±17.2 | 56.2 | **✔** (CT) |  |  |  |  |  |
|  | Jibril et al. (2022) | Pakistan | 581 | 56.3±14.8 | 69.2 | **✔** |  |  | **✔** |  |  |
|  | Munera et al. (2022) | Latin American Countries | 2552 | NR | NR | **NR** |  |  | **✔** |  |  |
|  | Prasetya et al. (2021) | Indonesia | 391 | 43.0±3.7 | 62.0 | **✔** |  |  | **✔** |  |  |
|  | Asmarawati et al. (2022) | Indonesia | 53 | 53.5±12.5 | 50.9 | **NR** |  |  | **✔** | **✔** |  |
|  | Brook et al. (2022) | Australia | 182 | 66±26.3 | 48.9 | **✔** |  |  | **✔** |  |  |
|  | Ceci et al. (2022) | Italy | 156 | Emergency group: 57.1 ±2; ICU: 66.8 ±2.2; deceased: 68.2 ±1.6 | 46.8 | **✔** |  |  | **✔** |  |  |
|  | **Outcome: ICU admission (only)** | | | | | | | | | | |
|  | Bellos et al. (2021) | Greece | 67 | 59 ± 17.4 | 65.7 | **✔** |  |  | **✔** |  |  |
|  | Bastug et al. (2020) | Turkey | 191 | 52 ± 13.6 | 56 |  | **✔** |  | **✔** |  |  |
|  | Paranjape et al. (2021) | United States | 2685 | 59.7 ± 17.3 | 51 | **✔** |  |  | **✔** |  |  |
|  | Hachim et al. (2020) | UAE | 417(DC: 289) | ICU: 57 ± 13; non-ICU: 44 ± 15 | VC (non-ICU: 57.5; ICU:42.5) | **✔** |  |  |  | **✔** |  |
|  | Cheng Fu-Yuan et al. (2020) | United States | 1987 | CD | 45.5 | **✔** |  | **✔** |  |  |  |
|  | Statsenko et al. (2021) | UAE | 560 | 40 ± 2.6 | 66.2 | **✔** |  |  | **✔** |  |  |
|  | Zhou Y et al. (2020) | China | 1087 (DC:763; VC:324) | DC: 51± 4.4; VC:51 ± 4.4 | DC: 48.6; VC: 47.5 | **✔** |  |  | **✔** |  |  |
|  | Shanbehzadeh et al. (2022) | Iran | 512 | 57.2 ± 17.6 | 75.8 | **✔** |  |  |  | **✔** |  |
|  | Suastika et al. (2021) | Indonesia | 382 | 48.5 ± 11.2 | 63 | **✔** |  |  |  |  | **✔** |
|  | Hashem et al. (2021) | Egypt | 351 (non-ICU: 206, ICU: 145) | non-ICU: 45.1 ± 17.1; ICU: 57.4 ± 14 | non-ICU: 47.6; ICU: 58.6 | **✔** |  |  | **✔** |  |  |
|  | Heo et al. (2021) | South Korea | 4663 | 53.5 ± 4.1 | 39.5 | **✔** |  |  | **✔** |  | **✔** |
|  | Durmus Kocak et al. (2021) | Turkey | 306 (non-ICU: 266; ICU: 40) | non-ICU 53.0 ± 15.8; ICU: 57.3 ± 15.4 | 58.8 | **✔** |  |  | **✔** |  |  |
|  | Guner et al. (2021) | Turkey | 686 | 45.6 ± 17.7 | 53.9 | **✔** |  |  | **✔** |  |  |
|  | Huespe et al. (2021) | Argentina | 1318 | 59 ± 50.5 | 51 | **✔** |  |  | **✔** |  |  |
|  | Geraili et al. (2022) | Iran | 724 | Survivors: 58.9±15.85; non-survivor: 71.4 ± 13.9 | 51.1 |  |  | **✔** | **✔** |  |  |
|  | Huang et al. (2021) | China | 681 | 62 ± 3.2 | 49.6 |  | **✔** |  | **✔** |  |  |
|  | Ketenci et al. (2022) | Turkey | 279 | 67.2 ± 15.3 | 63.8 | **✔** |  |  | **✔** |  |  |
|  | **Outcome: Mechanical ventilation** | | | | | | | | | | |
|  | Kulkarni et al. (2021) | United States | 528 (MV:79; non-MV: 449) | MV: 57.2 ± 13.9; non-MV: 54 ± 13.8 | 64.6 (MV); 68.4 (non-MV) | **✔** |  |  | **✔** |  |  |
|  | Amezcua‑Guerra et al. (2021) | Mexico | 100 | 55 ± 13 | 71 | **✔** |  |  | **✔** |  |  |
|  | Garcia-Gordillo et al. (2021) | Mexico | 401 (IMV = 142; No IMV = 259) | IMV: 58 ± 3.4; No IMV: 52 ± 4.3 | 65.8 | **✔** |  |  | **✔** |  |  |
|  | Alberdi-Iglesias et al. (2021) | Spain | 2040 | 66 ± 2.9 | 56.7 | **✔** |  |  | **✔** |  |  |
|  | Wen Li et al. (2022) | China | 516 | 60.7 ± 2.8 | 50.8 | **✔** |  |  | **✔** |  |  |
|  | Ashkenazi et al. (2022) | Israel | 385 | MV: 63.2 ± 14.3; non-MV: 54.4 ± 20.2 | 59 | **NR** | **NR** | **NR** | **All** |  |  |
|  | **Outcome: Combined outcomes (ECMO, HFNT, intubation, mortality, ICU-admission, MV)** | | | | | | | | | | |
|  | Arvind et al. (2020) | United States | 4087 | 58.6 ± 21.9 | 34.6 | **✔** |  |  | **✔** |  |  |
|  | Youssef et al. (2021) | United Kingdom | 472 | 68 ± 16 | 53.0 |  |  |  | **✔** |  |  |
|  | Jimenez‐Solem et al. (2021) | Denmark | 3944 | 53.0 ± 4 | 41.9 | **✔** |  |  |  | **✔** | **✔** |
|  | De Alencar et al. (2021) | Brazil | 180 | 60 ± 3.8 | 58.3 | **✔** |  |  | **✔** |  |  |
|  | Yu L et al. (2021) | United States | 1980 | 63.2 ± 17.1 | 51.2 | **✔** |  |  | **✔** |  |  |
|  | Zhou J et al. (2021) | China | 4644 | 45 ± 4.3 | 50.1 | **✔** |  |  | **✔** |  |  |
|  | Gresser et al. (2021) | Germany | 95 | 65 ± 3.8 | 77.9 | **✔** |  |  |  | **✔** |  |
|  | Gude et al. (2020) | Spain | 229 | 67 ± 3.4 | 60.7 | **✔** |  |  | **✔** |  |  |
|  | Aguirre-García et al. (2022) | Mexico | 529 | 55.8 ± 15 | 75.0 | **✔** |  |  | **✔** |  |  |
|  | Alessandri et al. (2022) | Italy | 263 | 71.7 ± 3.4 | 66.9 | **✔** |  |  | **✔** |  |  |
|  | Shanbhag et al. (2021) | India | 122 | 59 ± 14.3 | 77.8 | **✔** |  |  | **✔** |  |  |
|  | Venturini et al. (2022) | Italy | 458 | 67 ± 17 | 59 | **✔** |  |  | **✔** |  |  |
|  | Downing et al. (2021) | United States | 98 | 54 ± 14 | 61 | **✔** |  |  | **✔** |  |  |
|  | Gorgojo-Galindo et al. (2021) | Spain | 108 | Intubated/deceased 72.5 ± 15.2; non-intubated alive: 37 ± 54.4 | Intubated/deceased: 55; non-intubated alive: 54.4 | **✔** |  |  | **✔** |  |  |
|  | Torres-Macho et al. (2021) | Spain | 469 | 60.5 ± 16.2 | 45.8 | **✔** |  |  | **✔** |  |  |
|  | Varghese et al. (2021) | United States | 167 | 55 ± 17.6 | 64.1 | **✔** |  |  | **✔** |  |  |
|  | Tevald et al. (2021) | United States | 1486 | 64 ± 19.3 | 50.3 | **✔** |  |  | **✔** |  |  |
|  | Huang Dong et al. (2021) | China | 1370 | 53.7± 19.4 | 45.8 | **✔** |  |  | **✔** |  |  |
|  | Aly et al. (2021) | Egypt | 496 | 49.9± 16.3 | 57.1 | **✔** |  |  | **✔** |  |  |
|  | Pournazari et al- (2021) | United States | 724 | 60.9± 16.5 | 51.5 | **✔** |  |  | **✔** |  |  |
|  | Valencia et al. (2021) | Colombia | 245 | 62 ± 13 | 65 | **✔** |  |  | **✔** |  |  |
|  | Lombardi et al. (2021) | France | 14,43 | 68.4 ± 17.5 | 56.9 | **✔** |  |  | **✔** |  |  |
|  | Faria et al. (2021) | Portugal | 88 | 66.6 ± 22.9 | 56.8 | **✔** |  |  | **✔** |  |  |
|  | Hiremath et al. (2021) | China & United States | 869 | 55.8 ± 15.1 | 47.1 | **✔** |  |  | **✔** |  |  |
|  | Magunia et al. (2021) | Germany | 1039 (596 retrospectively  and 443 prospectively collected) | 63.3 ± 14.1 | 71.9 | **✔** |  |  | **✔** |  |  |
|  | Giamarellos-Bourboulis et al. (2022) | Greece & the Netherlands | 724 (DC:302; VC 1: 250; VC2:172) | DC: suPAR score (<6ng/ml): 53.2 ± 12.2; suPAR (<6ng/ml): 60.6 ± 12.3;  VC 1: suPAR (<6ng/ml): 53.4 ± 10.2; suPAR (<6ng/ml): 61.9 ± 13.6;  VC 2: suPAR (<6ng/ml):64.2 ± 13.2; suPAR (<6ng/ml): 66.8 ± 12.2 | DC: suPAR (<6ng/ml): 68.4; suPAR(<6ng/ml): 62.1;  VC 1: suPAR(<6ng/ml): 58.8; suPAR(<6ng/ml): 62.2;  VC 2: suPAR(<6ng/ml): 76.1; suPAR (<6ng/ml): 65.3 | **✔** |  |  | **✔** |  |  |
|  | González-Flores et al. (2022) | Mexico | 157 | 55 ± 12 | 66.3 | **✔** |  |  | **✔** |  |  |
|  | Alvarez-Uria et al. (2022) | India | DC: 4035; VC:2046 | DC: 47.34 ± 19.28; VC: 46 ± 18.55 | DC: 60.5; VC: 63.2 | **✔** |  |  | **✔** |  |  |
|  | He et al. (2022) | United States | 10752 | 60.8 ± 17.1 | 52.3 |  |  | **✔** | **✔** |  |  |
|  | Kamran et al. (2022) | United States | 9291 | 65.7 ± 3.3 | 53.3 |  | **✔** |  | **✔** |  |  |
|  | Kim et al. (2022) | South Korea | 44 | 59.7 ± 2.5 | 34.1 | **✔** |  |  | **✔** |  |  |
|  | Knight et al. (2022) | United Kingdom | 76588 | 71 ± 3 | 72.6 | **✔** |  | **✔** | **✔** |  |  |
|  | Kucuk et al. (2022) | Turkey | 85 | 67.7 ± 3.5 | 69.4 | **✔** |  |  |  | **✔** |  |
|  | Mu et al. (2022) | United States | 675 | 64.0 ± 3.5 | 57.5 | **NR** | **NR** | **NR** | **✔** |  |  |
|  | Nadasdi et al. (2022) | Hungary | 102 | 64.8 | 45.1 | **NR** |  |  | **✔** |  |  |
|  | Cervantes-Alvarez et al. (2022) | Mexico | 156 | 53.2 ± 13.2 | 60.6 | **✔** |  |  | **✔** |  |  |
|  | Cruciata et al. (2022) | Italy | 494 | 62.3 ± 20.1 | 54.0 | **✔** |  |  | **✔** |  |  |
|  | Hao et al. (2022) | United States | 7102 | 47.9 | 43.9 | **✔** |  |  | **✔** |  |  |
|  | Values are percentages or mean ± SD as applicable and available, unless otherwise specified. NS: under the mortality section it denotes non-survivors, but under the severity section it refers to non-severe; S: under the mortality section it denotes survivors, but under the severity section it refers to severe cases; SD: Standard Deviation; NR: value not reported or could not be found; PCR: polymerase chain reaction; COVID-19: Corona Virus Disease 2019; Ag: Antigen test; HFNT: High-Flow Nasal Therapy; ECMO: extracorporeal membrane oxygenation; ICU: Intensive Care Unit; IMV: invasive Mechanical Ventilation; MV: Mechanical Ventilation; DC: derivation/development/training set or cohort; VC: validation/test set or cohort. | | | | | | | | | | |
