# Supplementary material for: Prognostic models in COVID-19 infection that predict severity: a systematic review
Source: Eur J Epidemiol. 2023 Feb 25;38(4):355–72. doi: 10.1007/s10654-023-00973-x (PMC9958330; doi:10.1007/s10654-023-00973-x)
Supplement: Supplementary file 4 — Supplementary file4 (DOCX 228 KB) [file 10654_2023_973_MOESM4_ESM.docx]

**Table 4: Characteristics of eligible studies based on the type of the model, predictors, outcome, model performance and type of validation**

| **No.** | **Author (year)** | **Prediction time** | **Number of candidate predictors** | **Model** | **Predictors in the final model** | **Predictive performance of the model*** | | | | | | | | **Dev.** | **Internal validation** | **External validation** | **Dataset availability** |
| --- | --- | --- | --- | --- | --- | --- | --- | --- | --- | --- | --- | --- | --- | --- | --- | --- | --- |
|  |  |  |  |  |  | **Accuracy (%)** | **AUC/ROC** | **Sensitivity (%)** | **Specificity (%)** | **PPV (%)** | **NPV (%)** | **LR** | **C-index** |  |  |  |  |
| **Mortality (only)** | | | | | | | | | | | | | | | | | |
|  | Acar et al. (2021) | 9 ± 7 days | NR | Nomogram | Age, comorbidities, dyspnea, SpO_2_ (%), HCT, CRP, AST, Ferritin | NR | 0.92 | NR | NR | NR | NR | NR | NR | ✔ | - | - | Upon request |
|  | Aciksari et al. (2021) | NR | 8 | M-ATRIA RS | M-ATRIA-RS, cTnI, LDH and history of malignancy | NR | 0.74 (0.70-0.79) | NR | NR | NR | NR | NR | NR | ✔ | - | ✔ | NR |
|  | Al Abbasi et al. (2020) | NR | NR | Elevated troponin-I level in the first 24 h of admission | Age, atrial fibrillation, comorbidities, serum creatinine, lactic acid, total bilirubin, GLU, BUN, Na, and LYM count | 79.3 (74-84) | NR | 66 (52-78) | 83 (77-88) | 51.9 (43- 61) | 89.7 (86-93) | PLR= 3.89 (2.71-5.57); NLR = 0.41 (0.28 -0.59) | NR | ✔ | - | - | Statement provided |
|  | Alfaro-Martınez et al. (2021) | NR | NR | Alba-Score | T2DM, onco-hematologic disease, confusion, age, Heart rate, SaFiO_2_ and LDH | NR | Dev. cohort: (numerical scores: 0.86; categorical scores: 0.85); Validation cohort: (numerical scores: 0.85; categorical scores: 0.83) | NR | NR | NR | NR | NR | NR | ✔ | ✔ | - | NR |
|  | Allahverdiyev et al. (2020) | NR | NR | NLR | Age, NLR, LDH, GFR, ALT and AST on admission | NR | 0.84 (0.79–0.89) | 92 | 53 | NR | NR | NR | NR | ✔ | - | - | NR |
|  | Altschul et al. (2020) | NR | NR | Novel COVID-19 severity score | Age, sex, SaO_2_, MAP, international normalized ratio, creatinine, BUN, IL-6, CRP and PCT | NR | Dev. cohort: 0.82 (0.81–0.85); Validation cohort: 0.79 (0.79–0.82) | NR | NR | NR | NR | NR | NR | ✔ | ✔ | - | NR |
|  | Andreano et al. (2021) | 30 days | NR | Multivariate logistic regression model | Age, sex, comorbidities | NR | Dev.: 0.79; Internal validation: 0.78 | NR | NR | NR | NR | NR | 0.79 | ✔ | ✔ | - | NR |
|  | Asghar et al. (2020) | NR | 9 | Biochemical markers | LDH, PCT, DD, CRP, and ferritin | NR | PCT: 0.77, DD: 0.83, LDH: 0.72, CRP: 0.70, Ferritin: 0.64 | PCT:85; DD: 79.5; LDH: 59.8 | NR | PCT: 83.3; DD: 82.5; LDH: 80 | NR | NR | NR | ✔ | - | - | NR |
|  | Bertsimas et al. (2020) | 24 hrs. following admission | NR | COVID-19 Mortality Risk (CMR) tool | Age, SpO_2_, elevated CRP, BUN, and blood creatinine | 86.8 (82.3-91.3) | 0.91 (0.88-0.95) | NR | 87.4 (83.0-91.8) | NR | 97.1 (94.9-99.3) | NR | NR | ✔ | ✔ | ✔ | Upon request |
|  | Besutti et al. (2021) | 43 days | NR | Prognostic model based on clinical variables | Age, sex, HDL cholesterol, dementia, heart failure, vascular disease, time from symptom onset, NEU, LDH, chest CT and SaO_2_ | NR | Model without CT extension: Dev.: 0.94; validation: 0.93 (0.89–0.95); Model with CT extension: 0.95 (0.93–0.96), validation: 0.94 (0.91–0.9) | NR | NR | NR | NR | NR | NR | ✔ | ✔ | - | NR |
|  | Cai et al. (2021) | NR | NR | Nomogram | Age, CRP, DD | NR | Primary: 0.81 (0.7-0.9); Validation: 0.90 (0.8-1) | Dev.: 72.9; Validation: 86.4 | Dev.: 74.8; Validation: 89.5 | NR | NR | NR | Dev.: 0.81 (0.7-0.9); Validation: 0.90 (0.8-1.0) | ✔ | ✔ | ✔ | NR |
|  | **Cheng P et al. (2021)** | 25 day (symptoms onset to death) | NR | (a) APACHE II score; (b) MuLBSTA score | (a) Body temperature, Heart rate, breathing rate, BP, oxygen partial pressure, pH, K, Na, creatinine, HCT, WBC, consciousness, age, and chronic health evaluation (b= multilobular infiltration, LYM, bacterial infection, smoking status, hypertension, age | NR | (a) 0.90; (b) **0.99** | NR | NR | NR | NR | NR | NR | To investigate predictive performance of scoring system | - | - | ✔, Upon request |
|  | Ebell et al. (2021) | NR | NR | (a) COVID--Lab; (a) COVID-SimpleLab | (a) Age, respiratory rate, and SaO_2_; (b) Age, respiratory rate, SaO_2_, WBC, CRP, creatinine, and comorbid asthma | NR | (a) 0.77 (dev. group); 0.80 in the validation group; (b) 0.82 (dev. group) 0.83 in the validation group. | NR | NR | NR | NR | NR | NR | ✔ | ✔ | - | NR |
|  | Fan et al. (2021) | NR | 60 | 1. NLR Model; 2. NL model | NEU % and LDH with and without SaO_2_ using the Dev. data‐ set | NR | Dev. (NLR:0.93; NL model: 0.90; validation (NLR:0.91; NL model: 0.87) | NR | NR | NR | NR | NR | NR | ✔ | ✔ | ✔ | ✔, Upon request |
|  | Gue et al. (2020) | 30 days | NR | COVID mortality score | Age, sex and modified sepsis-induced coagulopathy score | NR | Dev.: 0.79 (0.75–0.84); validation: 0.77 | 67.6 | 78.4 | 72.6 | 74 | NR | NR | ✔ | ✔ | - | NR |
|  | Hajifathalian et al. (2020) | 7 & 14 days | 18 | COVID-AID risk tool | Age, MAP, presence of severe hypoxia, presence of kidney dysfunction | NR | Internal validation; 7-day: 0.86 (74.0–97.7); 14-day: 0.83 (0.69–0.97); External validation; 7-day: 0.85 (0.78–0.92); 14-day: 0.83 (0.76–0.89) | NR | NR | NR | NR | NR | NR | ✔ | ✔ (cross-validation) | ✔ | Statement provided in the supplemental material |
|  | Hu Hai et al. (2020) | NR | NR | REMS | MAP, pulse rate, respiratory rate, SpO_2_ GCS and age | NR | 0.84 (0.76- 0.91) | 89.5 | 69.8 | 39.5 | 96.8 | NR | NR | - | - | ✔ | NR |
|  | Hu C et al. (2021) | NR | NR | Logistic regression model | Age, hsCRP, LYM count and DD | NR | Dev.: 0.89; Ext. validation: 0.88 | Dev.:89.2; Ext. val.: 83.9 | Dev.: 98.7; Ext. val.:79.4 | NR | NR | NR | NR | ✔ | ✔ | ✔ | NR |
|  | Hu H et al. (2020) | NR | NR | COVID-19 risk model | Age, comorbidities, LYM count, NEU count, NLR, ALB and CRP | NR | 0.92 (0.83-1.00) | 90.5 | 84.2 | 86.4 | 88.9 | NR | NR | ✔ | - | - | NR |
|  | Jiang et al. (2021) | NR | NR | ABCS-mortality score | Age, biomarkers, COPD, sex, AST, hsCRP, hscTnI, WBC count, LYM count, DD, and PCT | NR | Dev.: 0.89 (0.87–0.91); Validation: 0.84 (0.78–0.90) | NR | NR | NR | NR | NR | NR | ✔ | - | ✔ | NR |
|  | King et al. (2020) | 30 days | >20 | VACO index | Age, sex, ethnicity, pre-comorbidities and CDCI derived from ICD-10 | NR | Dev.: 0.79 (0.77–0.81); validation 1: 0.81 (0.78–0.83); validation 2: 0.84 (0.78–0.86) | NR | NR | NR | NR | NR | NR | ✔ | ✔ | - | Statement provided |
|  | Knight et al. (2020) | NR | NR | 4C- Mortality Score | Age, sex comorbidities, respiratory rate, SpO_2_, level of consciousness, urea level and CRP | NR | Dev.: 0.77 (0.78-0.79); Validation: 0.77 (0.76-0.77) | NR | NR | NR | NR | NR | NR | ✔ | ✔ | - | NR |
|  | Laguna-Goya et al. (2021) | NR | NR | IL-6-based mortality risk model | IL-6, LDH, NEU to LymR, SpO_2_/FiO_2_ and age | NR | 0.94 (0.89-1) | 88 | 89 | 38 | 99 | NR | NR | ✔ | ✔ | - | NR |
|  | Li J et al. (2020) | 12 days | 11 | PLANS model | Platelet count, LYM count, age, NEU count and sex | NR | NR | NR | NR | NR | NR | NR | Dev.: 0.85 (0.83-0.87); Ext. validation: 0.87 (0.85-0.89) | ✔ | ✔ | ✔ | Upon request |
|  | Li L et al. (2021) | NR | NR | Nomogram | Age, severity on admission, dyspnea, CVD, LDH, total Bilirubin, GLU, urea | NR | NR | NR | NR | NR | NR | NR | Internal resampling: 0.97 (0.95-0.98); Internal validation: 0.96 (0.94-0.98); Ext. validation: 0.92 (0.86-0.98) | ✔ | ✔ | ✔ | NR |
|  | **Liu Q et al. (2020)** | NR | 9 | Prediction model | DD, LymR, BUN, PRE | NR | **0.99** (0.98 – 0.99) | 100 | 97.2 | 81.0 | 100 | NR | NR | ✔ | - | - | Data available on hospital website |
|  | Liu H et al. (2021) | 8 days following admission | NR | PAWNN score | Platelet count, age, WBC count, NEU count and NLR | 91.13 | Dev.: 0.92 (0.91–0.93)-0.93 (0.92–0.94); Internal validation: 0.97; Ext. validation: 0.80 | 93.8 (90.5–98.1) | 90.9 (85.1–92.8) | 46.3 (35.3–52.0) | 99.4 (99.1–99.8) | NR | NR | ✔ | ✔ | ✔ | Upon request |
|  | Liu S et al. (2020) | NR | NR | SOFA; qSOFA | SOFA score: PaO_2_/FiO_2_, creatinine, bilirubin, MAP, platelets, GCS; qSOFA: respiratory frequency, BP, vigilance | SOFA:84.2; qSOFA: 75.8 | SOFA: 0.92 (0.85–0.96); qSOFA: 0.74 (0.66–0.82) | SOFA: 90; qSOFA 70 | SOFA 83.2; qSOFA 80.4 | SOFA: 50; qSOFA: 40 | SOFA 97.8; qSOFA 93.5 | NR | NR | Evaluation of effectiveness | - | ✔ | NR |
|  | López-Escobar et al. (2021) | NR | NR | RIM Score | Age, sex, SpO_2_, CRP, NPR and NLR, VNPR and VNLR | NR | Internal validation; NPR model: 0.86 (0.82–0.90); NLR model: 0.85 (0.81–0.89); VPNR model: 0.90 (0.87–0.93); VNLR model: 0.86 (0.83–0.90) | NR | NR | NR | NR | NR | NR | ✔ | ✔ | - | Upon request |
|  | Ma X et al. (2020) | NR | NR | Death prediction model | Age, demographics, symptoms and laboratory tests | NR | Age: 0.91(0.83–0.98); Combined model: 0.98(0.96–1.00) | NR | NR | NR | NR | NR | NR | ✔ | ✔ | ✔ | Repository provided |
|  | Ma et al. (2020) | NR | NR | Nomogram based on 7 variables | Age, CHD, LYM%, platelets, CRP, LDH, DD | NR | 0.95 (0.92–0.97) | NR | NR | NR | NR | NR | NR | ✔ | - | - | Upon request |
|  | Magro et al. (2021) | 7-21 days | 10 | Simple clinical prediction app | Age, sex, duration of symptoms before admission shorter than 10 days, T2DM, CAD, chronic liver disease, LDH | NR | Internal validation: 0.82 (0.72–0.92); Ext. validation: 0.82 (0.72–0.92) | NR | NR | NR | NR | NR | NR | ✔ | ✔ | ✔ | Available online |
|  | Pan et al. (2020) | NR | 18 | Nomogram | CRP, PaO_2_/FiO_2_, cTnI | NR | Dev.: 0.98 (0.97 –1); Validation: 0.96 (0.87 –1) | NR | NR | NR | NR | NR | NR | ✔ | ✔ |  | Upon request |
|  | Pigoga et al. (2021) | NR | 34 | AFEM COVID-19 Mortality Scale (AFEM-CMS) | Sex, age, comorbidities, GCS, BP, respiratory and heart rate | NR | NR | NR | NR | NR | NR | NR | Dev.t: 0.78 (0.74-0.82); Valid. set: 0.72 (0.68-0.76) | ✔ | ✔ | - | Upon request |
|  | Quanjel et al. (2020) | 1 -33 days following admission | NR | NR | Labs (LDH, high sensitivity CRP and percent LYM) | 27 | NR | NR | NR | NR | NR | NR | NR | NR | - | ✔ | Partially available in the supplementary material |
|  | Satici et al. (2020) | Admission to death: 9.5 days | NR | (a) CURB-65; (b) PSI | (a) Confusion, urea, respiratory rate, BP, age; (b) Age, long-term care facility resident, comorbidities, symptoms at diagnosis and lab parameters | NR | CURB-65: 0.79 (72–86); PSI: 0.85 (78–90) | CURB-65: 73 (59–83); PSI: 80 (67-90) | CURB-65: 85 (82-88); PSI: 89 (86-91) | CURB-65: 31 (26-36); PSI: 39 (33-45) | CURB-65: 97 (96–98); PSI: 98 (97-99) | NR | NR | Assessment | - | ✔ | NR |
|  | Selcuk et al. (2021) | NR | 11 | DD levels | Age, DD 3^rd^ day following admission, WBC and creatinine |  | 0.90 (0.84-0.97) | 83.2 | 84.7 | NR | NR | NR | NR | Comparison | - | - | NR |
|  | Shang et al. (2020) | NR | NR | Scoring system of COVID-19 (CSS) | Age, CHD, LYM%, PCT and DD | NR | Dev. cohort: 0.92 (0.87-0.97); Valid. cohort: 0.94 (0.90-0.97) | NR | NR | NR | NR | NR | NR | ✔ | - | ✔ | NR |
|  | Soto-Mota et al. (2020) | NR | NR | LOW-HARM Score | Lymphopenia, SaO_2_ saturation, WBCs, hypertension, age, renal injury and myocardial injury | NR | 0.96 (0.94–0.98) | NR | 97.5 | 96 | NR | NR | NR | - | - | ✔ | NR |
|  | Stachel et al. (2021) | NR | NR | Machine learning algorithms; (Gradient boosting decision trees (GB)-highest performance) | Oximetry, respirations, BUN, LYM%, calcium troponin and NEU percentage | 0.82 | Gradient boosting decision trees (GB); 0.83 (0.80-0.86) | 53 | 91 | 64 | 8 | NR | NR | ✔ | ✔ | ✔ | Data are not available, but the model is available (link in the manuscript) |
|  | Tanboga et al. (2021) | 30 days | NR | Multivariable logistic regression model | Age, LDH, CRP, NLR, creatinine, DD, ALB, hemoglobin, platelet counts, presence of heart failure, T2DM, and pneumonia on CT were found to be the strongest predictors of 30‐day mortality. Age, LDH, ALB, CRP, and creatinine accounted for 80% of the variation in 30‐day mortality | NR | Dev.: 0.94 (0.94–0.95); Intern. valid.: 0.94 | NR | NR | NR | NR | NR | NR | ✔ | ✔ | ✔ | Upon request |
|  | Tezza et al. (2021) | NR | NR | Random forest | Age, vital signs (e.g., SaO_2_ and the quick SOFA) and lab parameters (creatinine, AST, LYM, platelets, and hemoglobin | NR | 84.0 (0.78 -0.9) | 79.0 (0.68-0.86) | 77.9 (0.72-0.82) | NR | NR | NR | NR | ✔ | - | - | Upon request |
|  | Wang X et al. (2020) | NR | 11 | NLR | CK, ALB, AST, serum creatinine and NLR | NR | 0.96 (0.91-1.00) NLR | 100 | 84 | NR | NR | NR | NR | ✔ | - | - | Upon request |
|  | Wang L et al. (2020) | NR | NR | MEWS | Systolic BP, pulse rate, respiratory rate, temperature and level of consciousness | NR | 0.91 (0.86–0.94) (cut off-4.5) | 67.6 | 94.5 | 78.1 | 94.1 | NR | NR | Evaluation | - | - | In supplemental files |
|  | Weng et al. (2020) | NR | NR | Nomogram (ANDC) | ANDC: Age, NLR, DD and CRP | NR | Dev.:0.92 (0.84–0.97); ext. validation: 0.98 | NR | NR | NR | NR | NR | NR | ✔ | ✔ | ✔ | Some data are available in the manuscript |
|  | Wongvibulsin et al. (2021) | 14 days | 105 | SCARP | Age, clinical severity, sex, demographic, admission, time-varying vital signs and comorbidities | NR | 1st Week: 0.89 (0.88–0.90); 2nd Week: 0.89 (0.87–0.91) | NR | NR | NR | NR | NR | NR | ✔ | ✔ (cross-validation) | ✔ | NR |
|  | Yang Y et al. (2021) | NR | NR | Nomogram | Age, breathing rate, LYM count, IL-6 | NR | Validation 1: 0.81 (0.76–0.96); Validation 2: 0.86 (0.70–0.92) | V1: 77.3; V2: 92.9 | V1: 73.5; V2: 64.5 | NR | NR | NR | NR | ✔ | ✔ | ✔ | Some data are available (link in the manuscript) |
|  | Yuan Y et al. (2020) | 30 days | NR | Risk score is based on 3 biomarkers | LDH, hsCRP and LYM % | NR | 0.96 | NR | NR | NR | NR | NR | NR | ✔ | ✔ | ✔ | Code implementation is available |
|  | Zayed et al. (2021) | NR | NR | CT-SS Score and CO-RADS score | CT-scans | NR | CT-SS:0.89 (0.84-0.95); CO-RAD: 0.97 (0.95-0.99) | NR | NR | NR | NR | NR | NR | Comparison | - | ✔ | Upon request |
|  | Zeng et al. (2021) | 8 weeks | 14 | Nomogram | WBC, CRP, LYM, LDH | NR | Dev.: 0.95 (0.91–0.98); Validation: 0.98; Overall: 0.95 | NR | NR | NR | NR | NR | NR | ✔ | ✔ | - | Upon request |
|  | Zhang S et al. (2020) | 14 & 28 days | NR | Nomogram | Age, LDH, AST, PT, Scr, Sodium, Fibrinogen, DD, NLR and direct bilirubin | NR | NR | NR | NR | NR | NR | NR | Dev.: 0.87 (0.87–0.90); ext. validation: 0.88 and 0.84 | ✔ | - | ✔ | Upon request |
|  | Zou X et al. (2020) | 14 days | NR | APACHE II score | NR | NR | 0.97 (0.94 – 0.99) | 96.15 | 86.27 | NR | NR | NR | NR | Assessment | - | - | NR |
|  | Ahirwar et al. (2022) | NR | NR | Biomarkers | Serum hsCRP, ferritin, IL-6 and plasma DD | NR | Plasma DD = 0.98 (0.97 -1.00) | NR | NR | NR | NR | NR | NR | Biomarkers analysis | - | - | NR |
|  | Aletreby et al. (2022) | NR | NR | 4C ISARIC Mortality score | NR | NR | 0.81 (0.79–0.83) | 70.5 | 74 | 62.4 | 80.2 | NR | NR | NR | - | ✔ | Upon request |
|  | Churpek et al. (2021) | 28 days following ICU admission | 20 | eXtreme Gradient Boosting (XG-Boost); STOP-COVID Mortality Index (SCMI) | XG-Boost model: age, number of ICU beds, serum creatinine, LDH, arterial pH, and P/F ratio | NR | XG-Boost ext. validation: 0.81; SCIM ext. validation:0.78 | NR | NR | NR | NR | NR | NR | ✔ | - | ✔ | NR |
|  | Raschke et al. (2022) | NR | 25 | C-TIME (COVID-19 Time of; Intubation Mortality Evaluation) | Age, sex, COPD, MAP, GCS, PaO_2_/FiO_2_, creatinine, bilirubin, length on IMV, corticosteroids and oral Xa inhibitors | NR | 0.75 (0.72–0.79) | NR | NR | NR | NR | NR | NR | ✔ | ✔ | - | Upon request |
|  | Reina Reina et al. (2022) | NR | NR | Machine learning model | Chronic airway obstruction, acute respiratory failure, demographic variables, and data from the medical records | 84.2 | 0.87 | 83.0 | 84.3 | NR | NR | NR | NR | ✔ | ✔ | - | Upon request |
|  | Riley et al. (2022) | 30 days | NR | 4C Mortality Score | Age, sex, comorbidities, respiratory rate, SaO_2_, GCS, BUN, and CRP | NR | 0.85 (0.79-0.89) | NR | NR | NR | NR | NR | NR | - | - | ✔ | NR |
|  | Shanbehzadeh et al. (2022) | NR | 58 | ANN-based CDSS | Nausea, oxygen therapy, loss of taste and/or smell, rhinorrhea, WBC count, platelet count, absolute NEU count, ESR, pleural fluid, ICU admission, length of stay and age | NR | 0.888 | NR | NR | NR | NR | NR | NR | ✔ | - | - | NR |
|  | Singh et al. (2022) | NR | NR | Biomarkers | BUN, ALB, BAR, CRP, and DD levels | NR | Best predictive power was ALB:.73 (0.63–0.82) | 73 | 64 | NR | NR | NR | NR | Investigation of biomarkers | - | - | NR |
|  | Surme et al. (2022) | NR | NR | SAD-60 score | SpO_2_, ALB, DD and age | NR | 0.78 | NR | NR | NR | NR | NR | NR | ✔ | - | - | NR |
|  | Van de Leur et al. (2022) | NR | NR | (a) Logistic regression model; (b) LASSO model; (c) Deep Neural Network (DNN) model | (a) age and sex; (b) LASSO: age, sex and human annotated ECG features; (c) DNN: age, sex and the raw ECG waveforms. | NR | (a) 0.73 (0.65–0.79); (b) 0.76 (0.68–0.82); (c) 0.77 (0.70–0.83) | NR | NR | NR | NR | NR | NR | ✔ | - | ✔ | NR |
|  | Vieira et al. (2022) | NR | NR | Risk score | Age, need for IMV, T2DM, CVDs and a laboratory or cardiological characteristic | NR | Age: 0.73; CRP: 0.84; DD: 0.73 | Age: 61.9; CRP: 73.7; DD: 67.5 | Age:63.3; CRP: 67.5; DD: 68.4 | NR | NR | NR | NR | ✔ | - | - | Upon request |
|  | Webb et al. (2022) | 28 days | NR | Simple scoring model | Age, T2DM, immunocompromised status and obesity, ethnicity, sex | NR | Dev. cohort = 0.91 (0.83–0.94); Validation cohort = 0.8 (0.69–0.90) | NR | NR | NR | NR | NR | NR | ✔ | - | ✔ | Upon request |
|  | Wirth et al. (2022) | NR | NR | 4C Deterioration Model and 4C Mortality Score | 4C Deterioration Model: age, sex, nosocomial infection, GCS, SpO_2_ on admission, breathing room air or oxygen therapy, respiratory rate, CRP, LYM count, and presence of radiographic chest infiltrates; 4C Mortality Score: age, sex, respiratory rate, SpO_2_, GCS, CRP and comorbidities | NR | 4C Deterioration Model: 0.78 (0.73–0.82); 4C Mortality Score: 0.85 (0.79–0.89) | NR | NR | NR | NR | NR | NR | - | - | ✔ | Upon request |
|  | Yilmaz et al. (2021) | NR | NR | CURB-65 Score; GESTALT | CURB -65 (confusion, BUN, respiratory rate, BP and age); GESTALT (Clinical gestalt is the theory of actively organizing the clinical perceptions of healthcare workers into coherent holistic structures) | NR | CURB-65 Score = 0.67; GESTALT = 0.63 | CURB-65 Score: 50; GESTALT: 78.9 | CURB-65 Score: 84.1; GESTALT: 44.4 | NR | NR | NR | NR | Comparison | - | - | NR |
|  | Leoni et al. (2021) | 28 days following ICU admission | NR | Multivariable prediction model | Age, obesity, procalcitonin, SOFA score and PaO_2_/FiO_2_ | NR | NR | NR | NR | NR | NR | NR | Dev.: 0.82 (0.77–0.88); Validation: 0.82 (0.77–0.87) | ✔ | ✔ | - | Authors state that all relevant data are in the manuscript |
|  | Marincu et al. (2021) | NR | 15 | Logistic regression model | Comorbidities, sex, and age | NR | 0.77 (0.71–0.82) | NR | NR | NR | NR | NR | NR | ✔ | ✔ | - | Upon request |
|  | Ottenhoff et al. (2021) | 21 days following admission | 80 | Extreme gradient boosting (XGB-10) and Logistic Regression (LR) | Age, medications, urea nitrogen, LDH, ALB, SpO_2_, SaO_2_, blood gas pH and history of CVDs. | NR | xGB-10: 0.82 (0.79-0.85); LR: 0.81 (0.77 -0.85) | NR | NR | NR | NR | NR | NR | ✔ | ✔ (cross-validation) | ✔ | Not available |
|  | Rozenbaum et al. (2021) | 7, 14 & 30 days | 109 | Light Gradient Boosting Machine (LightGBM) | 10 most important variables only |  | Validation cohort; 0.86 for 7-day; 0.88 for 14-day; 0.85 for 30-day mortality | NR | NR | NR | NR | NR | NR | ✔ | - | ✔ | NR |
|  | Ruscica et al. (2021) | NR | NR | Age and biochemical parameters | Age, NT-proBNP, IL-6 and LDH | NR | 0.88 (0.71-0.95) | 89 | 71 | 44 | 96 | NR | NR | ✔ | ✔ | - | NR |
|  | Valente Silva et al. (2021) | NR | NR | M-CHA2DS2-VASc Score | Modified CHA2DS2-VASc (congestive heart failure, hypertension, age, T2DM, prior stroke or transient ischemic attack, vascular disease | NR | 0.71 (0.64-0.77) | NR | NR | NR | NR | NR | NR | Score Evaluation | - | ✔ | NR |
|  | Cui et al. (2022) | NR | NR | Random forest (RF) algorithms models | Younger group: LDH, NEU, FIB, PT, CA, WBC, PCT, IL-6, LY, DD and IL-10; Older group: HCO3, LDH, AST; hsCRP, IL-10, NEU, Hs-CTnT, TCHO, IL-2R, IL-6 and TNF-α | NR | Younger group: 0.87 (0.83–0.92); older group: 0.84 (0.77–0.92) | NR | NR | NR | NR | NR | NR | ✔ | ✔ | - | NR |
|  | Ergenç et al. (2022) | NR | NR | Biomarker | PCT/ALB ratio (PAR) | NR | 0.95 | 87.3 | 91.3 | NR | NR | NR | NR | ✔ | - | - | NR |
|  | Falandry et al. (2022) | NR | NR | 8 variables (IADL8) score | Age increase of 10 years and IADL8 | NR | Dev.: 0.78 (0.72-0.85); Cross-validation = 0.8 (0.66 – 0.88) | NR | NR | NR | NR | NR | NR | ✔ | - | ✔ | NR |
|  | Marcolino et al. (2021) | NR | 36 | Risk score | Age, BUN, comorbidities, CRP, SpO_2_/FiO_2_ ratio, platelet count, and heart rate | NR | ABC 2-SPH: Dev. cohort: 0.84 (0.84-0.84); Validation cohort-0.86 (0.85-0.86). External validation: 0.89 (0.87-0.92) | NR | NR | ABC2-SPH:73.7 for patients at high mortality risk. | ABC2-SPH: Low-risk, intermediate-risk and high-risk groups, respectively 99.7, 88.1 and 71 | NR | NR | ✔ | ✔ | ✔ | NR |
|  | Alkaabi et al. (2021) | NR | 36 | Risk score | Age, LDH, creatinine, GCS, NEU %, SpO_2_ and respiratory rate |  | 0.88 (0.86-0.92) | 81 | 79 | NR | NR | NR | NR | ✔ | - | - | Upon request |
|  | Mahdavi et al. (2021) | 60 days | 37 | Machine learning model | Age, SpO_2_, CVD, PTT, BUN and LDH | 0.8 (0.797-0.803) | Joint model: 0.85 (0.85-0.85) | 73 | 88 | NR | NR | NR | NR | ✔ | ✔ | - | NR |
|  | Murri et al (2021) | CD | NR | Machine learning model | Age, platelet count, SpO_2_, BUN, hemoglobin, CRP, NEU count and sodium | NR | Dev. set: 0.87; Valid. set: 0.82 | Dev. set: 84; Valid. set: 81.3 | Dev. set: 76.6; Valid. set: 65.0 | Dev. set: 34.1; Valid. set: 40.5 | Dev. set: 97.1; Valid. set: 92.2 | NR | NR | ✔ | ✔ . | ✔ | NR |
|  | Heber et al (2021) | 4 days | 12 | Predictive model | Age, fever on admission, LDH, platelet count, CRP, LYM and creatinine | NR | Dev.: 0.88 (0.83-0.93), Valid.: 0.92 | NR | NR | NR | NR | NR | NR | ✔ | ✔ . | ✔ | Upon request |
|  | Kilercik et al. (2021) | NR | 29 | Hematocytometric index | MNR, NLR, PLT, RDW | 0.848 | 0.91(0.89-0.93) | 61.6 | 93.8 | 79.6 | 86.1 | LR+: 99; LR-:41 | NR | ✔ | - | - | Provided in supplement |
|  | Al Mutair et al. (2021) | NR | 15 | Predictive model | Age, sex, cough, septic shock, ARDS, AKI, NEUT, DD, BUN, Blood culture | NR | NR | NR | NR | NR | NR | NR | NR | ✔ | - | - | NR |
|  | Yang et al. (2021) | NR | 19 | Machine learning model | LDH, NLR and CRP | 0.98 | Decision tree model: 0.96, LDH: 0.94, NLR: 0.95, CRP: 0.90 | NR | NR | NR | NR | NR | NR | ✔ | ✔ | - | NR |
|  | Vicka et al. (2021) | NR | NA | Four prognostic scores compared (4cmortality score, SAPPS II, APACHE II and SOFA score) | NR | NR | SOFA: 0.68 (0.618-0.75), SAPS II: 0.76 (0.70-0.82), APACHE II: 0.77 (0.71-0.83) and 4C mortality score: 0.75 (0.69-0.81) | NR | NR | NR | NR | NR | NR | - | - | ✔ | NR |
|  | Jain et al. (2021) | NR | 11 | Predictive model | Age, CDCI, CRP, CT severity and DD on admission | NR | Age: **0.49 (0.31-0.68),** CMI:0.58 (0.42-0.75), CRP: 0.75 (0.61-0.89), DD: 0.86 (0.74-0.99), CT severity: 0.73 (0.58-0.88) | Age: 80; CMI:80; CRP: 80; DD: 80; CT severity: 80 | Age: 73; CMI:66.4; CRP:44.2; DD: 10.9; CT severity: 42.7 | NR | NR | NR | NR | ✔ | - | - | NR |
|  | Rahman et al. (2021) | NR | 23 | Prognostic model, Nomogram and machine learning | Age, RB-DW, WBC, LYM count, monocytes, platelet count, NEU count | Dev.: 88; Validation: 7 features-91 | Dev. set: 0.95, inter. validation: 0.88, ext. validation: 0.96 | Dev.: 87; Validation: 7 features: 91 | Dev.: 90; Validation: 7 features: 91 | NR | NR | NR | NR | ✔ | ✔ | ✔ | Chinese data available; Bangladeshi data NR |
|  | Ponce et al. (2021) | 28 days | 18 | Machine learning model | Age, MV, Vasopressors, WBC, AST, renal replacement, infection severity on admission, AKI etiology sepsis, hypertension, creatinine, AKI etiology COVID, sex, time from COVID to AKI, diuresis, nephrotoxic medication and dehydration | NR | Dev.: 0.88 (0.83-0.97); Validation: 0.82 (0.75-0.88) | NR | NR | NR | NR | NR | NR | ✔ | ✔ | - | Upon request |
|  | Haji Aghajani et al. (2021) | NR | 14 | Nomogram | Age, sex, need for ICU/MV, pulse rate, lymphocytes, RBC, Troponin 1, LDH | Dev.: 81.93; Validation: 73.18 | Dev.t: 0.82 (0.80-0.86), Validation: 0.80 (0.73-0.86) | Dev.: 72.3; Validation: 64.4 | Dev.: 86.1; Validation: 85 | Dev.: 62.1; Validation: 47.8 | Dev.: 92.1; Validation: 89.1 | NR | Dev.: 0.89 (0.86-0.91); Validation: 0.85 (0.78-0.91) | ✔ | ✔ | - | NR |
|  | Garrafa et al. (2021) | NR | 19 | Brescia early-warning model | Age, LDH, DD, NLR, CRP, LYM %, ferritin and monocytes % and Brescia chest X-ray score | NR | Dev.: 0.97 (0.97–0.98); Validation: 0.83 (0.80–0.87) | Dev.: 93 (91-97); Validation: 82 (72-92) | Dev.: 92 (88-94); Validation: 75 (63-83) | NR | NR | NR | NR | ✔ | ✔ | - | Reported that data cannot be shared |
|  | He et al. (2021) | NR | 40 | Nomograms | Nomogram1 (NG): age, dyspnea, anorexia, WBC, NLR, PLT, AST, ALB, CRP; NG2: age, dyspnea, NLR, CRP | Dev.: Nomogram1: 85.28; (83.81-89.15); Nomogram2: 85.94; (84.46-87.42); Validation: Nomogram1: 88.70; (87.10–90.30); Nomogram2: 89.56; (88.02–91.11) | Dev. (NG1: 0.92 (0.88-0.96); NG2: 0.90 (0.86-0.94); Validation (NG1: 0.92; (0.86–0.98); NG2: 0.89; (0.83–0.96)) | Dev. (NG1: 86.4 (77.3-93.9); NG2: 80.3 (70.7-89.9); Validation: NG1: 86.7 (74.50–98.); NG2: 76.7 (61.5–91.8) | Dev.: N1:85.2 (83.6-88.9). N2: 86.1 (84.6-87.6).; Validation: NG1: 88.74; (87.12–90.35); NG2: 89.8; (88.3–91.4) | NR | NR | Dev. (NG1: LR+:5.85, LR-: 0.16; NG2: LR+: 5.78, LR-: 0.22);Validation: NR | NR | ✔ | ✔ | - | Upon request |
|  | Moghaddam-Tabrizi et al. (2021) | NR | NR | Prognostic model | Age, pulmonary symptoms, need for MV, brain symptoms, nasal airway, job, GI symptoms, brain disease history, CVD history, heart symptoms, CKD history, dyspnea, history of drug use and hypertension | Dev.: 89.8 (85.6-93.1); Validation: 94.7 (89.4-97.9) | Dev.: 0.83; Validation: 0.79 | Dev.: 91.9 (87.6-95.1); Validation: 94.5 (88.5-97.9) | Dev.: 89.5 (77.3-96.5); Validation: 95.7 (73.9-99.8) | NR | NR | NR | NR | ✔ | ✔ | - | NR |
|  | Nishikimi et al. (2021) | 14 & 28 days | 36 | Intubated COVID‑19 predictive (ICOP) score | Age, history of CKD, BUN, ferritin, oxygen index, pH, MAP, dose of needed vasopressor | NR | Dev.: 0.75 (0.73-0.78); validation: 0.71(0.67-0.75) | NR | NR | NR | NR | NR | NR | ✔ | ✔ | - | Upon request |
|  | Sosa et al. (2021) | On admission & 5^th^ day following admission | NR | Lung Ultrasound Score; (LUS) | LUS | NR | LUS on admission: 0.64; LUS at 5^th^ day: 0.80 | LUS on admission: 63; LUS at 5^th^ day: 75 | LUS on admission: 59; LUS at 5^th^ day: 78 | LUS on admission: 65; LUS at 5^th^ day: 80 | LUS on admission: 64; LUS at 5^th^ day 84 | NR | NR | ✔ | - | - | NR |
|  | Banoei et al. (2021) | NR | 108 | Machine learning model | 18 clinical and comorbidities predictors and 3 blood biochemical markers. Top most differentiating mortality predictors (CAD, T2DM, altered mental status, age) | Dev. set: 90 and validation: 87 | Dev. set: 0.95; Validation: 0.91 | Dev.: 80 and validation: 75 | Dev. set: 92 and validation: 90 | NR | NR | NR | NR | ✔ | ✔ | - | Upon request |
|  | Riva et al. (2021) | NR | NR | Prognostic model | Monocyte Distribution Width | NR | 0.76 (0.66–0.87) | 75 | 70 | NR | 93 | NR | NR | ✔ | - | - | Upon request |
|  | **Rahman et al. (2021)** | NR | NR | Nomogram | Age, Lymphocyte count, DD, CRP and Creatinine (ALDCC), information acquired at hospital admission | Dev.: 91 | Dev.: 0.987, Internal validation: **0.999; external validation**: 0.992 | Dev.: 91 | Dev.: 78 | NR | NR | NR | NR | ✔ | ✔ | ✔ | Publicly available |
|  | Timpau et al. (2021) | NR | NR | Prognostic model | DD and CRP | NR | CRP: 0.71 (0.62-0.79) and DD: 0.74 (0.66-0.82) | CRP (cut-off 0.74): 65.7 and DD (cut-off 48.5): 61.8 | CRP (cut-off 0.74): 70.8 and DD (cut-off 48.5): 62.5 | NR | NR | NR | NR | ✔ | - | - | All data provided in the manuscript |
|  | Martín-Rodríguez et al. (2021) | 48 hrs. | 30 | Risk prediction model | Age, location (rural or urban), institutionalization, desaturation, rhonchus, tachypnea, and altered level of consciousness | NR | Dev. cohort: 0.72 (0.68-0.75); validation: 0.74 (0.69-0.79); re-validation: 0.76(0.73-0.80) | Metrics were presented for three risk levels (i.e., low (L), intermediate (I) and high (H)) in three models, i.e., Global model, SARS-COV-2 negative and SARS-COV-2 positive. Global-L: 98 (94-100), I: 67 (57-76) and H: 12 (7-17); SARS-CoV-2 negative – L: 97 (94-99), I: 58 (47-68), H: 8 (3-13) and SARS-CoV-2 positive-L: 96 (93-98), I: 70 (59-80) and H: 13 (7-18). | Global-L: 27.9 (22-32), I: 64 (54-73) and H: 97 (95-98); SARS-CoV-2 negative – L: 25 (20-30), I: 63 (53-73), H: 98 (96-99) and SARS-CoV-2 positive-L: 32 (27-36), I: 61 (51-71) and H: 96 (94-98). | Global-L: 25 (23-25), I: 33 (29-36) and H: 67 (58-74); SARS-CoV-2 negative – L: 17 (15-17), I: 21 (18-23), H: 47 (35-57) and SARS-CoV-2 positive-L: 35 (33-36), I: 42 (39-45) and H: 63 (58-68). | Global-L: 98 (96-99), I: 90 (88-91) and H: 82 (81-83); SARS-CoV-2 negative – L: 99 (97-99), I: 91 (90-92), H: 88 (87-88) and SARS-CoV-2 positive-L: 96 (93-97), I: 85 (82-87) and H: 74 (73-75). | Global-L: (LR+)1,35 (1,28-1,41), (LR-)0,06 (0-0,13), I:(LR+)2,08 (1,76-2,4), (LR-)0,47 (0,39-0,56) and H: (LR+)6,35 (5,23-7,47), (LR-)0,90 (0,86-0,94); SARS-CoV-2 negative – L: (LR+)1,30 (1,24-1,36), (LR-)0,08 (0,01-0,16), I:(LR+)1,74 (1,43-2,05), (LR-)0,63 (0,54-0,72) and H: (LR+)4,98 (3,99-5,97), (LR-)0,93 (0,89-0,97) and SARS-CoV-2 positive-L: (LR+)1,41 (1,35-1,47), (LR-)0,11 (0,06-0,17), I: (LR+)1,97 (1,71-2,21), (LR-)0,45 (0,36-0,54) and H: (LR+)5,25 (3,93-6,57), (LR-)0,90 (0,86-0,93) | NR | ✔ | ✔ | - | NR |
|  | Kar et al. (2021) | 7- & 28-days following admission | 63 | Machine learning model | Age, sex, respiratory distress, T2DM, CKD, CAD, respiratory rate, SaO_2,_ LYM % in DLC, INR, LDH and Ferritin | Dev.: 97.1; validation: 93.0 | Dev.: 0.88; Validation: 0.78 | Dev.: 78.1; validation: 60.5 | Dev.: 98.7; validation: 95.9 | Dev.: NR; validation: 57.7 (48.6-66.3) | Dev.: NR; validation: 96.4 (95.3-97.2) | Dev.: NR and validation-LR+: 15.1 (10.4-21.7) and LR-: 41(0.3-54) | NR | ✔ | ✔ | (Ongoing) | Data can-t be shared |
|  | Yu et al. (2021) | 14 & 28 days | NR | Prognostic model with cardiac-specific biomarkers | hs-TnI, CK-MB andMYO | NR | Early stage: HS-Tnl (7.9 pg/ml)-0.84 (0.78-0.89); CK-MB (1.2 ng/ml)-0.78 (0.72-0.85) and MYO (80.8 ng/ml)-0.89 (0.84-0.94); Late stage: HS-Tnl (15.7 pg/ml)-0.94 (0.90-0.98); CK-MB (1.5 ng/ml)-0.92 (0.87-0.96) and MYO (98.0 ng/ml) -0.96 (0.92-1.00). | Early stage: HS-Tnl (7.9 pg/ml)-77 (65-87); CK-MB (1.2 ng/ml)-71 (59-82) and MYO (80.8 ng/ml)-85 (74-93); Late stage: HS-Tnl (15.7 pg/ml)-89 (79-96); CK-MB (1.5 ng/ml): 85 (74-93) and MYO (98.0 ng/ml)-91 (81-97) | Early stage: HS-Tnl (7.9 pg/ml)-77 (75-80); CK-MB (1.2 ng/ml)-76 (74-79) and MYO (80.8 ng/ml) -86 (84-88); Late stage: HS-Tnl (15.7 pg/ml)-94 (93-96); CK-MB (1.5 ng/ml)-91 (89-93) and MYO (98.0 ng/ml) -0.96 (0.95-0.97) | Early stage: HS-Tnl (7.9 pg/ml)-16 (14-27) CK-MB (1.2 ng/ml)-15 (13-23) and MYO (80.8 ng/ml)-26 (23-43); Late stage: HS-Tnl (15.7 pg/ml)-48 (41-70); CK-MB (1.5 ng/ml)-35 (31-54) and MYO (98.0 ng/ml)-56 (49-78) | Early stage: HS-Tnl (7.9 pg/ml)-98 (97-99); CK-MB (1.2 ng/ml)-98 (96-98) and MYO (80.8 ng/ml)-99 (98-99); Late stage: HS-Tnl (15.7 pg/ml)-99 (99-100); CK-MB (1.5 ng/ml)-99 (98-99) and MYO (98.0 ng/ml) -100 (99-100) | Early stage: HS-Tnl (7.9 pg/ml)-LR+: 3.42 (2.89-4.05) and LR-: 0.29 (0.19-0.46); CK-MB (1.2 ng/ml)-LR+:2.99 (2.49-3.60) and LR-: 0.38 (0.26-0.55) and MYO (80.8 ng/ml)-LR+: 6.13 (5.14-7.31) and LR-:0.18 (0.10-0.31); Late stage: HS-Tnl (15.7 pg/ml)-LR+:16.00 (12.45-20.55) and LR-:0.11 (0.06-0.23); CK-MB (1.5 ng/ml)-LR+: 9.58 (7.76-11.83) and LR-:0.17 (0.09-0.29) and MYO (98.0 ng/ml) -LR+:22.50 (16.83-30.07) and LR-:0.10 (0.04-0.20) | NR | ✔ | - | - | The original contributions presented in the study are included; in the article/supplementary materials, further data upon request |
|  | Zelikovna-Golukhova et al. (2022) | NR | 28 | Transthoracic Echocardiography-Based Prediction Model | Right ventricle/left ventricle area, sPAP and right ventricle free wall longitudinal strain | NR | 0.93 (0.86–0.99) | 93.8 | 81.9 | NR | NR | NR | NR | ✔ | - | - | NR |
|  | Alhamar et al. (2022) | NR | NR | Biomarkers | Sex, Non-Kuwaiti nationality, asthma, GLU categories | NR | Dev.: 0.90; internal validation: 0.83; ext. validation: 0.69 | Dev.: 75; 66.9; 66.7 | Dev.: 86.3; internal validation:76.7; ext. validation: 70.7 | Dev.: 47.8; internal validation:30.2; ext. validation: 23.3 | Dev.: 95.4; internal validation: 93.9; ext. validation: 94.1 | NR | NR | ✔ | ✔ | ✔ | Upon request |
|  | Jamshidi et al. (2022) | NR | NR | Machine learning based mortality prediction model | Age, sex, medical history, symptoms (around 20 parameters in total) | NR | Dev.: 0.80; validation: 0.79 | Dev.: 76.1; validation: 74.9 | NR | Dev.: 21.4; validation: 21.2 | Dev.: 96.5; validation: 96.6 | NR | NR | ✔ | ✔ | - | Upon request |
|  | Araiza et al. (2021) | NR | NR | Ichikado CT score | The Ichikado CT score is based on the involvement of the lung | NR | 0.87 for a cut-off of 172 points on the Ichikado CT score | 84.2 | 79.7 | NR | NR | NR | NR | ✔ (Evaluation) | - | - | Upon reasonable request |
|  | Alkhasawneh et al. (2021) | 28 days | 3 | Single predictor | Monocytes to LYM ratio had the highest accuracy index of the 3 tested parameters. The following values are for MLR with a cut-off of 0.67. | 71.55 | NR | 66.8 | NR | 73.82 | 69.68 | NR | NR | ✔ (evaluation of parameters) | - | - | NR |
|  | Bae et al. (2021) | NR | 143 radiomics features | Radiomics based model | 32 radiomics features | NR | 0.83 | 79 | 74 | NR | NR | NR | NR | ✔ | ✔ | - | Statement in the manuscript promising only portion of data will become available soon |
|  | Gordon et al. (2022) | 30 days | 26 | 4C Mortality Score | Age, sex, respiratory rate, SaO_2_, number of comorbidities, level of consciousness, BUN and CRP | NR | 4C validation: 0.76 (0.76-0.77); RECOVER 0.79 (0.77-0.80) | NR | NR | NR | NR | NR | NR | Score Evaluation | ✔ | ✔ | No data are available |
|  | Gutierrez-Camacho et al. (2022) | NR | NR | CDCI | CDCI cutoff > 31.69 | NR | 0.75 (0.69-0.8) | 63.6 (54.8–71.8) | 87.7 (82.6–91.7) | NR | NR | NR | NR | Score Evaluation | - | - | Upon reasonable request. |
|  | Hassan et al. (2022) | 30 days |  | 4C mortality score; CURB-65 score | 4C: age, sex, respiratory rate, oxygen SaO_2_, GCS, urea, CRP, comorbidities; CURB-65: mental confusion, urea, respiratory rate BP, age | NR | NR | NR | NR | NR | NR | NR | Lombardy: 4C:0.85 (0.82− 0.89); CURB: 0.80 (0.75− 0.85). Leiden: 4C: 0.87 (0.80− 0.94), 0.82 (0.76− 0.88) |  | ✔ | ✔ | Upon reasonable request. |
|  | Hippisley-Cox et al. (2021) | 14 days | 43 | Cause specific Cox proportional hazard models | Age, sex, ethnicity, deprivation, BMI, comorbidities and SARS- CoV-2 infection rate | NR | NR | NR | NR | NR | NR | NR | 74.1(71.1- 77) | ✔ | ✔ | - | NR |
|  | Hohl et al. (2022) | NR | 19 | Canadian COVID-19 Emergency Department Rapid Response Network (CCEDRRN) COVID-19 Mortality Score | Age, sex, type of residence, arrival mode, chest pain, liver disease, respiratory rate and level of respiratory support | NR | 0.92(0.9-0.93) | NR | NR | NR | NR | NR | NR | ✔ | ✔ | - | Upon request |
|  | Huang CY et al. (2022) | NR | NR | Hematologic and biochemical parameters | Age, sex and comorbidities. | NR | CRP 0.68 and 0.74; LDH: 0.68; Ferritin: 0.68; DD: 0.76; NLR: 0.57 | CRP: 89.5, 47.4; LDH: 52.6; Ferritin: 47.4; DD: 73.7; NLR: 52.6 | CRP: 46.2, 90.4; LDH: 81.3; Ferritin: 82.8; DD:75.1; NLR: 66 | NR | NR | NR | NR | ✔ | - | - | NR |
|  | Jalalvand et al. (2022) | NR | 33 | Logistic regression model | GGO, age, LYM count, creatinine, CRP, LDH, and BP | GGO: 94 (83-99); age: 66 (54-77); Creatinine:18 (9-30); LYM. count: 82 (70-90) | GGO:0.96 (0.91-1); Age: 0.79 (0.65- 0.93); Creatinine: 0.76 (0.62-0.89); LYM. count: 0.79 (0.62-0.96) | GGO: 100 (74-100); age: 83 (52-98); Creatinine 100 (72-100); LYM count: 78 (40 -97) | GGO:92 (78 -98); age: 63 (49 -75); Creatinine: 44 (30-59); LYM count: 82 (69-92) | GGO:100 (89-100); age: 32 (21-82); Creatinine 1 (83-100); LYM count: 95 (80-98) | GGO: 100 (89-100); age: 95 (79-97); Creatinine 100 (83-100); LYM count: 95 (80-98) | Comorbidities: 1.9 (1.2, 3.2); Hb: 0.7 (0.5, 0.9) | NR | ✔ | - | - | NR |
|  | Khari et al. (2022) | NR | NR | qSOFA, CURB-65, SIRS |  | NR | SIRS 0.62 (0.55-0.69); CURB-65: 0.66 (0.59-0.73); qSOFA: 0.61 (0.54-0.67) | SIRS: 85.2 (75.7-91.6); CURB-65: 96.6 (89.7-99.1); qSOFA: 78.4 (68.1-86.2) | SIRS: 34.3 (26.6-42.9); CURB-65: 6.57 (3.2-12.5); qSOFA: 38 (30.6-47.4) | SIRS: 45.5 (37.8-53.4); CURB-65: 39.9 (33.3-46.8); qSOFA: 45.1 (37.1-53.3) | SIRS: 78.3 (65.5-87.5); CURB-65: 75 (42.8-93.3); qSOFA: 73.6 (61.7-82.9) | NR | NR | ✔ | ✔ | - | NR |
|  | Kibar Akilli et al. (2022) | NR | NR | PSI/PORT, A-DROP, NEWS-2, MEWS, CURB-65, Expanded CURB-65, qSOFA, qCSI, 4C mortality | NR | NR | PSI/POST: 0.97 (0.96-0.98); A-DROP: 0.93 (0.91-0.95) | PSI/POST: 91.7; A-DROP: 84.2 | PSI/POST: 91.9; A-DROP: 86.1 | PSI/POST: 52.1; A-DROP: 37 | PSI/POST: 99.1; A-DROP: 98.3 | PSI/POST: 25.2 (11.2-56.4); A-DROP: NR | NR | ✔ | ✔ | ✔ | Upon request |
|  | Klen et al. (2022) | NR | 20 | Machine learning-based online model CODOP | Age, NEU, CRP, creatinine, LDH, serum Na, serum K, GLU and DD, and reduced values of platelets, eosinophils and monocytes | NR | Range: 0.90–0.96 | Range: 78–100 | Range: 89–97 | NR | NR | NR | NR | ✔ | ✔ | ✔ | US cohort data is available, others are available upon request to ethics committee |
|  | Kucuk, Berkay et al. (2022) | NR | NR | NUTRIC (nutrition risk in the critically ill) score, modified NUTRIC | NUTRIC: age, APACHE II score, SOFA score, comorbidities, length of stay before admittance to ICU and IL-6. mNUTRIC: NUTRIC without IL-6 | NR | NUTRIC: 0.79 (0.74-0.85); mNUTRIC: 0.79 (0.74-0.84) | NR | NR | NR | NR | NR | NR | ✔ | - | - | Upon request |
|  | Laino et al. (2022) | NR | 37 | Machine learning model | Age, P/F ratio, hs-TI, BNP, IL-6, PCT, RDW, urea, creatinine, ALB | NR | 0.85 (0.82–0.87) | NR | NR | NR | NR | NR | NR | ✔ | ✔ | - | NR |
|  | Li G et al. (2021) | NR | 26 | Biomarker | Age, sex; hypertension, T2DM, CHD; WBC, cTnI, hs-CRP and creatinine | NR | NR | 76.9 (68.5–85.1) | 74.9 (70.3–78.9) | NR | NR | 98 (0.98–0.99) | NR | ✔ | - | - | Upon request |
|  | Li Y et al. (2022) | NR | 22 | Two-step risk score | Age, comorbidities, respiratory rate, SpO_2,_ heart rate, BUN, NLR, Platelets, CRP, NEU | NR | NR | NR | NR | NR | NR | NR | 0.83 (0.79–0.87) | ✔ | ✔ | ✔ | Upon request |
|  | Lyons et al. (2022) | NR | NR | QCOVID | NR | NR | NR | NR | NR | NR | NR | NR | Females: 0.95 (0.94-0.96); males: 0.93 (0.92-0.95) |  | - | ✔ | NR |
|  | Martın-Rodrıguez et al. (2022) | 90 days | NR | Quick COVID-19 Severity Index (qCSI) and the National Early Warning Score (NEWS) | NR | NR | qCSI (1^st^ day mortality: 0.77; 90-day mortality: 0.75;) NEWS (1^st^ day mortality: 0.83; 90-day mortality: 0.78 | NR | NR | NR | NR | NR | NR | - | - | ✔ | Upon request |
|  | Morello et al. (2022) | 30 days | NR | 4C Mortality dichotomic rule | NR | NR | 0.93 (0.84-1) | NR | NR | NR | NR | NR | NR | ✔ | ✔ | ✔ | Upon request |
|  | **Moulaei et al. (2022)** | NR | 54 | Machine learning algorithms | Dyspnea, ICU admission, oxygen therapy, age, fever, cough, loss of taste; loss of smell, hypertension, muscular pain, chills, runny nose, BUN, T2DM, sore throat, absolute LYM count nausea, comorbidities, chest pain, absolute NEU count, headache, sex, GI symptoms, WBC, CCRP, hsTI, Pneumonia, GLU, ESR, Creatinine; Alkaline phosphatase, length of stay AST, smoking, ALT | 95 | **0.99** | 90.7 | 95.1 | NR | NR | NR | NR | ✔ | ✔  (Cross-validation) | - | Upon request |
|  | Mousavi et al. (2021) | NR | 35 | Machine learning | RBC, platelets, hemoglobin, hematocrit, and platelet distribution width, all laboratory tests, age, sex, and the need for special care, such as oxygen therapy and ventilation | NR | NR | NR | NR | NR | NR |  | NR | ✔ | - | - | NR |
|  | Munoz et al. (2022) | 30 days | NR | PESI | Age, sex, comorbidities, heart rate, BP, respiratory rate, body temperature, mental status and SaO_2_ | NR | 0.71 (0.63- 0.79) | NR | NR | NR | NR | NR | NR | - | - | ✔ | NR |
|  | Najafi et al. (2021) | 30 days | 24 | NR | Age, CKD, AKI, GFR | NR | NR | GFR: 72.8 | GFR:65.8 | NR | NR | NR | NR | ✔ | ✔ | - | NR |
|  | Naser et al. (2021) | NR | 1 | NR | AKI | NR | NR | NR | NR | NR | NR | NR | NR | ✔ | - | - | All relevant data are available within the manuscript and its supporting information files |
|  | Ocho et al. (2022) | NR | NR | 4C mortality score | Age and sex | 0.84 (0.76–0.92) | NR | 82 | 74 | NR | NR | NR | NR | - | - | ✔ | Upon request |
|  | Özdemir et al. (2021) | NR | NR | Biomarkers | ALB, CRP, DD, CRP/ALB ratio | NR | CRP/ALB ratio: 0.81 | 71.1 | 71.4 | NR | NR | NR | NR | Investigation of prognostic markers | - | - | NR |
|  | Özdemir et al. (2022) | 30 days | 3 | 3 scoring systems | modified early warning score (MEWS), rapid acute physiology score (RAPS) and rapid emergency medicine score (REMS) | MEWS:20.5; RAPS:9.9; REMS:35.1 | MEWS:0.51 (0.42-0.60); RAPS:0.50 (0.41-0.59); REMS: 0.68 (0.59-0.76) | MEWS: 53.8; RAPS: 15.4; REMS: 84.6 | MEWS 25.7; RAPS 96.5; REMS 50.5 | MEWS 8; RAPS 25; REMS 16.9 | MEWS 82.4; RAPS 90.4; REMS 96.5 | MEWS LR+:72, LR-:18; RAPS LR+:27.9, LR-: 90; REMS LR+: 17.1, LR-= 30 | NR | Comparison of scoring systems | - | - | NR |
|  | Ozger et al. (2021) | 28 days | 8 | Biomarkers, measured serially at different sampling times | GCS, IL-6, IL-7, IL-10, IL-15, IL-27 IP-10, MCP-1, DD, Ferritin, CRP | NR | IL-6: 0.69 (0.51–0.83); IL-10: 0.64 (0.46–0.79) | NR | NR | NR | NR | NR | NR | Evaluation of predictive value of cytokines | - | - | All relevant data are within the paper and its supporting information files. |
|  | Pasculli et al. (2022) | NR | 3 | Chest computed tomography (CT) score | Chest CT findings; cycle threshold (Ct) values in RT-PCR of SARS-CoV-2; secondary infection occurrence | NR | CT: 0.68; Ct values: 0.75 | CT: 61.9 (55.3-68.1); Ct values: 75.8 (69.3-81.2) | CT: 68.8 (51.4-82.1); Ct values: 69.4 (53.1-82) | NR | NR | NR | NR | Investigation of CT findings and Ct values | - | - | NR |
|  | Plecko et al. (2022) | NR | NR | RECOILS score; Rapid Evaluation of Coronavirus Illness Severity (RECOILS) | Age, platelets, PaO_2_/FiO_2_ ratio, pH, BUN, temperature, PaCO_2_, GCS measured within 24 hrs. following ICU admission | NR | 0.78 (0.76–0.81) | NR | NR | NR | NR | NR | NR | ✔ | - | ✔ | Not publicly available |
|  | Asaduzzaman et al. (2022) | On admission | NR | Hemogram-derived ratios | NLR, derived NLR and NPR | NR | NLR, d‐NLR, NPR, PLR, and; SII were 0.66 (0.58–0.73), 0.65 (0.58–0.73), 0.68 (0.61–0.75), 0.55; (0.46–0.63), and 0.60 (0.52–0.67) respectively | NR | NR | NR | NR | NR | NR | Evaluation of hemogram-derived ratios | - | - | - |
|  | Aygun et al. (2022) | 28 days | 1 | Emergency department triage early warning score (TREWS) and modified early warning score (MEWS) | TREWS: age, respiratory rate, SaO_2_, need for oxygen support, body temperature, systolic BP, heart rate, and consciousness; MEWS: systolic BP, heart rate, respiratory rate, temperature | NR | MEWS: 83 (0.78–0.89) | MEWS (cut-off of 0.5): 96.5 | 46.8 | NR | NR | NR | NR | Evaluation of effectiveness of scoring systems | - | - | NR |
|  | Ayvat et al. (2022) | CD | 7 | CT scan | APACHE, CT score, CRP and age | 89.9 | NR | NR | NR | NR | NR | NR | NR | ✔ | - | - | NR |
|  | Baikpour et al. (2022) | On admission | NR | Chest X-ray (CXR) scoring system | Age, sepsis, S/F ratio, need for MV and the chest x-ray severity score | NR | 0.93 (0.89- 0.96) | NR | NR | NR | NR | NR | NR | ✔ | ✔ | - | Upon request |
|  | Bartoszko et al. (2022) | Repeated-measures day 0 to 15 | 61 | Biomarkers and physiological variables | Age, temperature, lactate level, ventilation tidal volume, and vasopressor use | NR | 0.90 (0.8-0.9) | NR | NR | NR | NR | NR | NR | ✔ | ✔ | - | NR |
|  | Beigmohammadi et al. (2022) | CD | 2 Scores | APACHE II and SOFA scores | APACHE II and SOFA scores | NR | 0.89 for SOFA Score | NR | NR | NR | NR | NR | NR | Evaluation of mortality values of two models | - | - | NR |
|  | Bengelloun et al. (2022) | 4 days following admission | 1 | CONtrolling NUTritional status (CONUT) index | CD | NR | 0.71 (0.68–075) | NR | NR | NR | NR | NR | NR | To determine the usefulness of CONUT | - | - | NR |
|  | Besutti et al.b (2022) | On admission | CD | CT scan, CXR, CRP or SpO_2_ | age and sex, severity/time indices (based on the ratio; between measures of disease severity and time from symptom onset) | NR | CT :0.92 (0.89–0.95); CXR: 0.90 (0.86–0.94); CRP: 0.88 (0.83–0.93); SpO_2_: 0.88 (0.84–0.92) | NR | NR | NR | NR | NR | NR | ✔ | - | ✔ | NR |
|  | Bezerra et al. (2022) | On ICU admission | NR | Urinary biomarkers, NGAL, KIM-1, MCP-1 and nephrin | proteinuria*KIM-1*NGAL | NR | 0.81 (0.69–0.93) | Combined proteinuria, KIM-1, NGAL: 71 | 86 | NR | NR | NR | NR | Evaluation of urinary biomarkers | - | - | NR |
|  | Bodolea et al. (2022( | First 24 hrs. following ICU admission | NR | Biological nutritional risk assessment instruments | Prognostic Nutritional Index (PNI), the Controlling Nutritional Status Score—(CONUT), the Nutrition Risk in Critically Ill (NUTRIC), and the modified NUTRIC (mNUTRIC), along with CT-derived fat tissue and muscle mass measurements | NR | PNI (best discriminative capability for mortality): 0.77 | NR | NR | NR | NR | NR | NR | Evaluation of the role of four biological nutritional risk assessment instruments | - | - | NR |
|  | Bradley et al. (2022) | On admission | 2 scores | CURB-65; Pneumonia Severity Index (PSI) | CURB-65: confusion, urea > 7 mM, respiratory rate, BP, age; PSI | NR | PSI: 0.82 (0.78-0.86); CURB-65: 0.79 (0.75-0.84) | 83 | 66 | 37 | 94 | NR | NR | Evaluation of the ability of PSI and CURB-65 score | - | - | NR |
|  | **Chikhalkar et al. (2022)** | On admission | NR | NEWS2 Score | NEWS2 Score | 0.96 | **0.99** | 97 | 96.6 | NR | NR | NR | NR | Assessment of the NEWS2 | - | - | NR |
|  | Chou et al. (2022) | On admission | 9 | NR | Age, BMI, CKD, CHF, Hepatitis, Transplant, NLR, LDH, NEWS2 | NR | Dev. cohort: 0.94 (0.92–0.96) | NR | NR | NR | NR | NR | NR | ✔ | ✔ | - | Upon request |
|  | Cidade et al. (2022) | On ICU admission | 1 | Biomarker | DD levels | NR | 0.57 (0.42-0.71) | NR | NR | NR | NR | NR | NR | ✔ | - | - | Upon request |
|  | Citu Cosmin et al. (2022a) | On admission |  | Multiple scores: 4C Mortality Score, NEWS and CURB-65 Scores | NEWS Score |  | 0.86 (0.68-0.92) | 100 | 51 | NR | NR | NR | NR | Evaluation and comparison of multiple scores | - | - | Upon request |
|  | Citu Cosmin et al. (2022b) | On admission | 1 | Coagulation parameters: DD, INR, PT, aPTT | DD levels | NR | 0.75 | DD: 76 | 61 | NR | NR | NR | NR | Evaluate predictive value of PTT | - | - | Upon request |
|  | Comoglu et al. (2022) | On admission | 1 | CDCI | CDCI categorized into groups: CDCI score 0, CDCI score 1–2, CDCI score 3–4, CDCI score 5–6, and CDCI score ≥7. |  | 0.81 (0.75–0.86) | 87.2 | 61.4 | 18. | 97.9 | NR | NR | Examining the predictive role of CDCI | - | - | NR |
|  | Lee DS et al. (2021) | 30 days | 47 | Machine learning (Random Forest) | 304 variables including all demographic, interRAI functional, LTC-related, and community characteristics, along with comorbidities and laboratory tests | NR | 0.70 (0.67-0.74) | NR | NR | NR | NR | NR | NR | ✔ | - | - | NR |
| **Disease severity and/or critical illness (Definitions are found in Table 6 of the supplemental)** | | | | | | | | | | | | | | | | | |
|  | Zhou Y et al. (2020) | NR | NR | LASSO regression models/logistic regression models | Body temperature on admission, cough, dyspnea and comorbidities | NR | NR | NR | NR | NR | NR | NR | Dev.: 0.86 (0.80–0.93); internal validation: 0.84 | ✔ | ✔ | - | Within the manuscript files |
|  | Ageno et al. (2021) | NR | NR | SIMI SCORE | Age, CAD, CRP, AST, DD, and NLR | NR | Dev.:0.79; validation: 0.80 | 93 | 34 | 59 | 82 | NR | NR | ✔ | - | ✔ | NR |
|  | Bello-Chavolla et al. (2020) | CD | >20 | MSL-COVID-19 and Nutri-CoV score | Pneumonia, T2DM, age, chronic kidney disease, any form of immunosuppression, COPD, obesity and age; Nutri-CoV score includes MSL-COVID-19 plus respiratory rate and pulse oximetry | NR | NR | NR | NR | NR | NR | NR | Mortality: 0.72 (0.69–0.75); severity: 0.78 (0.75–0.80); Nutri-Cov score: Dev.: 0.79 (0.77–0.83); validation: (0.77 (0.75–0.80) | ✔ | ✔ | ✔ | Upon request |
|  | Bennouar et al. (2020) | 3-30 days | 12 | Biological markers | Elevated urea nitrogen, LDH, NLR and CRP, ALB and Na | NR | 0.87 (ALB); 0.7-0.81 (other biomarkers) | 68 | 93 | NR | NR | NR | NR | Biomarkers comparison | - | - | NR |
|  | Bennouar et al. (2021) | CD | NR | RISK SCORE based on biological routine; parameters | Age, CRP, NLR, urea, Na, ALB, and LDH | NR | Severity: 0.74 (0.66–0.82); Mortality: 0.90 (0.87–0.94) | 82 | 91 | NR | NR | NR | NR | ✔ | ✔ | - | NR |
|  | Boero et al. (2021) | CD | 10 | COVID-19 Worsening Score (COWS) | LUS score, comorbidities, days from symptoms onset, dyspnea on admission and P/F ratio | 80 | NR | 85 (75-96) | 75 (64-84) | 58 (46-71) | 93 (87-98) | NR | NR | ✔ | ✔ | - | Upon request |
|  | Chen et al. (2021) | NR | NR | CANPT (for severity of COVID-19); CAN score (for predicting mortality) | Body temperature, NLR, PLT count, total bilirubin, creatinine, CK and ALB | CANPT: 85.9; CAN. 97.4 | CANPT: 0.84 (0.82–0.89); CAN: 0.90 (0.83-0.97) | NR | NR | NR | NR | NR | NR | ✔ | ✔ (leave-one-out cross validation) | - | Upon request |
|  | De Socio et al. (2021) | CD | NR | NEWS2 ≥ 4 | NEWS2 parameters: respiratory rate, hypercapnic respiratory failure, supplemental oxygen, body temperature, systolic BP, pulse rate and level of consciousness. | 76.9 (68.3–84.0) | 0.87 (0.80–0.93) | 86 (73.3–94.2) | 70.4 (58.4–80.7) | 67.2 (58.4–74.9) | 87.7 (78–93.5) | 2.9 (2–4.2) | NR | Comparison of scores | - | ✔ | Upon request |
|  | Fernandes et al. (2021) | CD | NR | 5 Machine learning algorithms (artificial neural networks, gradient boosted decision tree, random forests, catBoost, and extreme gradient boosting | 57 variables; 3 most important variables; ratio of lymphocyte per CRP and Braden Scale | NR | All Algorithms-average AUROC: 0.92 | 92 | 82 | NR | NR | NR | NR | ✔ | ✔ (cross validation) | - | Upon request |
|  | **Gao et al. (2021)** | 20 days | 28 | 5 machine learning models, including logistic regression, support; vector machine (SVM), gradient boosted decision tree (GBDT), k-nearest neighbor, and neural network; (NN) | Immune-inflammatory parameters: PCT, [T + B + NK cell] count, IL-6, CRP, IL-2 receptor, T-helper LYM/T-suppressor LYM | NR | Best performance (SVM, GBDT, and NN); Internal validation: **0.991** (0.98–1); Ext. validation: **0.999 (0.99–1)** | NR | NR | NR | NR | NR | NR | ✔ | ✔ | ✔ | Upon request |
|  | Liu L et al. (2021) | NR | 8 | Prognostic model based on LC, LDH and hsCRP | LYM count, LDH and hsCRP | NR | Dev.: 0.88 (0.80–0.95); internal validation: 0.85 (0.76–0.93); ext. validation: 0.84 (0.69–0.95) | 75 | 90.7 | 88.2 | 79.6 | NR | NR | ✔ | ✔ (leave-one-out cross-validation) | ✔ | NR |
|  | Li S et al. (2021) | NR | NR | CT score | Number of CT scan, Lung involvement, time consumed for CT reading, ICC between two radiologists, ICC between two clinicians, ICC between radiologists and clinicians | NR | 0.75 (0.57-0.93) | 81 | 69.20 | NR | NR | NR | NR | ✔ | - | - | NR |
|  | Liang et al. (2020) | NR | >20 | COVID-GRAM | Chest X-ray abnormalities, hemoptysis, dyspnea, unconsciousness, number of comorbidities, cancer history, NLR, LDH, direct bilirubin | NR | Dev.: 0.88 (0.85-0.91); internal validation: 0.88 (0.84-0.93) | NR | NR | NR | NR | NR | NR | ✔ | ✔ | - | NR |
|  | Liu Q et al. (2021) | NR | NR | Machine learning classifiers; (XGBoost best performance) | LYM%, LDH, NEU count, DD and four quantitative CT features | 90.6 | 0.96 (0.91–1)-XGBoost clinical model | 100 | 87.8 | 70.6 | 100 | NR | NR | ✔ | ✔ (cross-validation) | - | NR |
|  | Liu, J et al. (2020) | NR | NR | Nomogram | Age, COPD, temperature, fatigue, shortness of breath, lymphocyte percentage | NR | Dev. cohort: 0.77 (0.73-0.82); Internal validation: 0.73 (0.67-0.79); ext. validation: 0.77 (0.71-0.83) | NR | NR | NR | NR | NR | NR | ✔ | ✔ | ✔ | NR |
|  | Marcos et al. (2021) | NR | NR | Machine learning model composed of random forest, XG boost, regularized logistic regression | Peripheral blood oxygen saturation (SpO2)/fraction of inspired oxygen (FiO2) ratio, age, estimated glomerular filtration rate, PCT, CRP, updated CDCI and LYM | NR | Internal validation: 0.86 (0.83–0.88; Ext. validation: 0.83 (0.81– 0.85) | NR | NR | NR | NR | NR | NR | ✔ | ✔ (cross validation) | ✔ | Upon request |
|  | Myrstad et al. (2020) | NR | NR | NEWS2 (threshold≥ 6) | NEWS2: physiological parameters; respiratory rate, SaO_2_, systolic BP, pulse rate, level of consciousness or new confusion, and body temperature | NR | 0.82 (0.69–0.95) | 80 (52.9-95.7) | 84.3 (71.4-92.9) | 60 (43.1-74.8) | 93.5 (83.8 -97.5) | NR | NR | Assess and compare the performance of NEWS2 | - | - | Upon request |
|  | Prower et al. (2021) | NR | NR | NEWS2; ROX Index | Respiration rate, oxygen saturation, supplemental oxygen, systolic BP, pulse rate, level of consciousness and temperature (NEWS2) | NR | NEWS2: 0.82 (0.80-0.83); ROX index: 0.85 (0.84-0.86) | NEWS: 72; ROX: 85 | NEWS: 79; ROX: 75 | NR | NR | NR | NR | Test the predictive ability of NEWS2 and compare with ROX | - | - | Upon request |
|  | Purkayastha et al. (2021) | CD | 15 | Four models based on CT radiomics and clinical features | Age, sex, fever, WBC, LYM count and comorbidities | NR | Model 1: 0.89, 0.93, and 0.93 for the prediction of progression risk at 3, 5 and 7 days, respectively | NR | NR | NR | NR | NR | Model 1: 0.87 (0.83–0.91); Model 2: 0.77 (0.71–0.83); Model 3: 0.85 (0.80–0.89); Model 4: 0.86 (0.82–0.90) | ✔ | ✔ | - | Some supplementary information available |
|  | Schalekamp et al. (2021) | CD | NR | Model (based on clinical, laboratory and radiographic findings obtained on admission) | Age, sex, COPD, comorbidities, leukocytes, LYM, NLR, CRP, LDH, preexistent abnormalities in chest Rx, lung involvement, chest Rx score. | NR | 0.77 (0.72-0.81) | NR | NR | NR | NR | NR | NR | ✔ | ✔ | - | NR |
|  | Schöning et al. (2021) | NR | 7 | COVID-19 severity assessment (COSA) | Sex, CRP, sodium, hemoglobin, eGFR, GLU and leucocytes | NR | Dev. AUROC: 0.94; Internal validation: 0.85 | NR | NR | Dev.:97; intern. valid.:91 | Dev.:80; intern. valid.:81 | NR | NR | ✔ | ✔ (cross validation) | ✔ | Score available, but data only upon request |
|  | Shi Y et al. (2021) | NR | NR | COVID-GRAM score CALL score | COVID-GRAM: Age, chest X-ray, Hemoptysis, Dyspnea, unconsciousness, Comorbidities, ANC/ALC ratio, LDH, Direct bilirubin; CALL: comorbidities, age, ALC/ALC, LDH | NR | COVID-GRAM (mortality: 0.78 (0.53-0.92); critical illness: 0.70 (0.44-0.87) CALL (mortality: 0.64; (0.36-0.85); critical illness: 0.57 (0.32-0.79)) | NR | NR | NR | NR | NR | NR | - | - | ✔ | Upon request |
|  | Su Y et al. (2020) | NR | NR | NEWS-C (most accurate) IRS | Age, clinical severity, sex, demographic, admission source, time-varying vital signs, and comorbid conditions were used as the predictors. | NR | EDRF: 0.79 (0.69–0.89); IRS: 0.89 (0.82–0.96) | 59.3 | 85.4 | NR | NR | NR | NR | Investigating the predictive value of NEWS | - | - | Some data available in the article and supplementary materials |
|  | Tu et al. (2021) | NR | NR | Clinical Nomogram model | Sex, age, comorbidities, BMI, temperature, LYM, LDH to LYM ratio, platelet, CRP, DD, total protein, ALB, α-HBDH, lung involvement | NR | Dev.: 0.91 (0.87-0.96); Validation: 0.87 (0.76-0.99) | Dev.:84; Validation:66 | Dev.:86; Validation: 80 | NR | NR | NR | NR | ✔ | ✔ | - | The datasets used in this study are available from the corresponding author upon reasonable request. |
|  | Ucan et al. (2021) | NR | NR | PSI; CURB-65; A-DROP; CALL score; COVID-GRAM score | Comparison | NR | PSI :0.87 (0.82–0.93); CURB-65 :0.86 (0.80–0.91); A-DROP: 0.88 (0.82–0.94); CALL score: 0.83 (0.77–0.88); COVID-GRAM score :0.86 (0.80–0.91) | NR | NR | NR | NR | NR | NR | Investigation of prognostic values | - | - | NR |
|  | Woo et al. (2021) | 14 days | >20 | Web- Based Severe COVID-19 Risk Prediction Model | Age, sex, T2DM, dyspnea, CRP, AST, DD and troponin |  | Severe COVID-19 (Dev.: 0.82; validation: 0.82); Mortality (Dev.: 0.85; validation: 0.81 | 83 | 60 | NR | NR | NR | NR | ✔ | ✔ | - | NR |
|  | Wu et al. (2020) | NR | NR | Machine learning models | Age, LYM%, CRP, LDH, CK, urea and Ca | external -; 74.4 to 87.5 | Model 4 (combination model); Dev. dataset: 0.86 (0.82–0.90); Validation set: 0.90 (0.82–0.98); Ext. validation = ranging from 0.84 to 0.93 | Range: 75- 96.9 | Range: 55- 88 | NR | NR | NR | NR | ✔ | ✔ (cross validation) | - | NR |
|  | Xiao et al. (2020) | NR | NR | Deep learning model | Clinical indicators and imaging findings | Dev. set: 97.4; Test set: 81.9 | Dev. set: **0.987** (0.97–1); Valid set: 0.96 (0.88–1.00) | NR | NR | NR | NR | NR | NR | ✔ | ✔ | - | Upon request |
|  | Xu F et al. (2021) | NR | 21 | K–Nearest Neighbor (KNN) model (highest AUC in the validation set) | DD, CRP, age, WBC count, LDH, and ALB | 89.6 (85.9 – 93.3) | 0.95 (0.92 – 0.97) | 83.2 (75.7 – 90.6) | 93.5 (89.7 – 97.2) | 88.4 (81.9 – 95.0) | 90.2 (85.8 – 94.7) | NR | NR | ✔ | ✔ |  | Upon request |
|  | Xu J et al. (2021) | NR | NR | Predictive model | CRP, prothrombin time, age and SpO_2_ | NR | 0.94 | 96 | 78 | NR | NR | NR | NR | ✔ | - | - | Upon request |
|  | Yao et al. (2021) | 10 days | 31 | Machine learning‐based Nomogram | ESR, CDCI, Age, LDH and CRP | NR | Dev.: 0.82 (0.77–0.88); internal validation: 0.76 (0.77–0.84); ext. validation: 0.71 (0.63–0.78): | NR | NR | NR | NR | NR | NR | ✔ | ✔ | ✔ | NR |
|  | Yu Y et al. (2020) | NR | 12 | Multivariate logistic regression models | Serum amyloid A protein (SAA), ESR, age | NR | SAA: 0.92 | 83.9 | 97.67 | NR | NR | NR | NR | Identification of biomarkers | - | - | Upon request |
|  | Zhang B et al. (2020) | 30 days | NR | Nomogram | Age, LDH, AST, PT, Scr, Sodium, Fibrinogen, DD | Internal valid.: 83.3; extern. Valid.: 79.1 | Internal validation: 0.93; ext. validation 1 (0.95) and 2 (0.88) | Intern. valid.: 93.6; ext. val.1: 100; ext. val.1: 79 | Intern. valid.: 80.7; ext. val.1: 76.5; ext. val.2: 83.8, | Intern. valid.: 55.; ext. val.1: 34.3; ext. val.2: 82.2 | Intern. valid.:98; ext. val.1: 100; ext. val.2: 95.3 | NR | NR | ✔ | ✔ | ✔ | Some data available in the article and supplementary materials, further data upon request |
|  | Assal et al. (2022) | NR | NR | Multivariate logistic regression model | Elevated DD, leukocytosis, and elevated CRP | NR | TLC:0.75; DD: 0.76; CRP:0.70 | TLC: 61; DD: 70; CRP: 67 | TL: 78; DD: 70; CRP: 65 | TLC: 37; DD: 34; CRP: 30 | TLC: 90; DD: 91; CRP: 90 | NR | NR | Investigation | - | - | Upon request |
|  | Shalmon et al. (2022) | CD | NR | Chest CT images on segmented lungs | CT images and NLR | NR | 0.92 (0.83-0.98) | NR | NR | NR | NR | NR | NR | ✔ | - | - | Upon request |
|  | Shankar et al. (2022) | NR | NR | Nomogram scoring system | 9 variables: age group, sex, education, chronic kidney disease, tobacco, cough, dyspnea, olfactory-gustatory dysfunction [OGD], and gastrointestinal symptoms = basic model, ; 9 variables above (except OGD) and CRP, LDH, ferritin, DD, and CT severity score = advanced model | NR | NR | NR | NR | NR | NR | NR | Basic model: 0.78 (0.74–0.82); Advanced model: 0.83 (0.79–0.87) | ✔ | ✔ | - | NR |
|  | Shi et al. (2022) | NR | NR | Radiomics nomogram | Radiomics signature, comorbidities, WBC | Dev.: 97; Valid.: 97 | Dev.: 0.97; Valid.: 0.98 | Dev.: 88; valid.:89.4 | Dev.: 90; valid.:92.9 | NR | NR | NR | NR | ✔ | ✔ | - | Statement provided, but no full dataset |
|  | Tang et al. (2022) | NR | NR | Nomogram models | IgG, NLR, LDH, platelets, ALB, and BUN | NR | NR | NR | NR | NR | NR | NR | 0.86 and 0.87 for two nomograms | ✔ | - | - | Upon request |
|  | Vela et al. (2022) | NR | NR | COVID-19 risk stratification system | Age, sex, a summary measure of the comorbidity burden, the socioeconomic status, and the presence of diagnosed comorbidities potentially associated with severe COVID-19 | NR | Hospital admissions:0.85 (0.85–0.85); ICU transfers: 0.86 (0.86–0.97); death: 0.96 (0.96–0.96) | NR | NR | NR | NR | NR | NR | ✔ | - | ✔ | Upon request |
|  | Wong et al. (2021) | NR | 97 | Simplified prediction model (lite) | Anthropometric measures (BMI, weight, WC), demographic variables (e.g., age, sex, ethnic group), and general indicators of health (number of medications taken, comorbidities ). | NR | Severe infection: (0.72 (0.71-0.74); fatal infections: 0.8 (0.79-0.84) | NR | NR | NR | NR | NR | NR | ✔ | - | ✔ | NR |
|  | Xiong et al. (2022) | NR | NR | Machine learning techniques (random; forest (RF), support vector machine (SVM), and logistic regression (LR) | Chest CT, fever, malignancies, heart rate, systolic BP, hemoglobin, NLR, RDW, immature granulocyte, IL-6, ESR, indirect bilirubin, alkaline phosphatase, cystatin C, LDH, Ca2+, α-fructosidase, amylase, high sensitivity troponin, myoglobin, prothrombin time, DD. | 84.5 | RF: 0.97 | 96.7 | 69.5 | NR | NR | NR | NR | Comparing different ML techniques | - | - | Upon request |
|  | Zhang et al. (2022) | NR | NR | Nomogram | Age, NEU count, creatinine, number of involved lung segments and PCT | NR | 0.94 (0.89–0.98) | NR | NR | NR | NR | NR | NR | ✔ | - | - | NR |
|  | Zhao et al. (2022) | NR | 50 | Prediction model | Age, comorbidities, chest CT, NEU count, LDH, CRP, hemoglobin and DD levels | NR | Dev.:0.94 (0.89–0.99); validation: 0.82 (0.80–0.97) | NR | NR | NR | NR | NR | NR | ✔ | - | ✔ | NR |
|  | Gómez et al. (2021) | NR | NR | Multivariate logistic regression model based on blood parameters | LYM, CRP, creatinine, total protein levels, GLU and AST | NR | 0.89 (0.85-0.93) | 81.5 | 81 | NR | NR | NR | NR | ✔ | - | - | NR |
|  | Monterde et al. (2021) | NR | NR | Queralt DxS tool-comprehensive index of the comorbidity burden | Age, sex, and comorbidities present on admission measured using three indices: the CDCI, the Elixhauser index, and the Queralt DxS index for comorbidities on admission | NR | Queralt DxS index: 0.79 (0.77–0.80) | NR | NR | NR | NR | NR | NR | ✔ | - | - | Tool is available online |
|  | Muto et al. (2021) | NR | NR | Predictive model | Age, sex, T2DM, hemodialysis, need for O_2_ supplementation upon diagnosis and CRP | NR | Dev. :0.86 (0.81-0.90); validation: 0.83 (0.58-1) | Dev. :43.0; validation: 40 | Dev.: 93.9; validation. 100 | Dev.: 74; validation:100 | Dev.: 80.4; validation: 88 | NR | NR | ✔ | ✔ | - | NR |
|  | Rinderknecht et al. (2021) | 28 days following COVID-19 diagnosis | NR | Prognostic, prediction model | Age, BMI, sex and comorbidities | NR | 0.86 (0.84-0.88) | NR | NR | NR | NR | NR | NR | ✔ | ✔ | - | Available but restrictions apply |
|  | Bennett et al. (2021) | 1 day | 64 | Predictive model | age, oxygen saturation, respiratory rate, BUN, systolic BP, and AST | Range: 62.4-70.9 | 0.87 (0.86-0.88) | NR | NR | NR | NR | NR | NR | ✔ | ✔ | - | NR |
|  | Sengel et al. (2021) | NR | NR | CALL score | Age, comorbidities, LDH and LYM count | NR | 0.59 (0.50-0.66) | 93.1 (83.3-98.1) | 19.5 (14-25.9) | 26.6 (20.7-33.2) | 90.0 (76.3-97.2) | 35 (13.2-95.4) | NR | - | - | ✔ | Available |
|  | Li Xue-lian et al. (2021) | NR | 15 | Nomogram | Age, sex, T2DM, dyspnea, tachycardia, LYM count, anemia, CRP, multilobar involvement | CD | Dev.: 0.89 (0.86–0.93); internal validation: 0.85 (0.79–0.91) | Dev.: NR; Validation: 65.6 | Dev.: NR; Validation: 85.1 | Dev.: NR; Validation: 62.6 | Dev.: NR; Validation: 85.4 | NR | Dev.: 0.88; Validation: 0.82 | ✔ | ✔ | - | NR |
|  | Huang Jiana et al. (2021) | NR | 30 | Prognostic model | Age, lobular involvement score and lymphocyte cell count | NR | 0.90 | 90.9 | 78.1 | NR | NR | NR | NR | ✔ | - | - | Upon request |
|  | Ma et al. (2021) | NR | 34 | Prognostic severity score | Carcinoma, dyspnea, CRP, T2DM, respiratory rate, WBCs, Platelets and LYM | NR | Dev.: 0.83 (0.78-0.88); validation: 0.79 (0.73-0.87) | CD | CD | NR | NR | NR | NR | ✔ | ✔ | - | NR |
|  | An et al. (2022) | 12 days | 11 | Inflammation/coagulopathy/immunology responsive index (ICIRI) | CRP, fibrinogen, DD, CD8 T cell count | NR | Overall progression: 0.70 (0.59−0.81); severe progression: 0.65 (0.51-0.78); critical progression: 0.80 (0.65-0.95) | Overall progression: 52.9; severe progression: 48.6; critical progression 73.3 | total progression: 96.7; severe progression 96.7; critical progression: 90.6 | NR | NR | NR | NR | ✔ | - | - | Upon request |
|  | Gurusamy et al. (2021) | NR | NR | Logistic regression | NLR, age, sex, comorbidities, NLR | NR | 0.89 [0.85-0.93] | 80.6 | 87.1 | NR | NR | 19.8 (10.2-38.6) | NR | ✔ | - | - | NR |
|  | Haimovich et al. (2020) | 24 hours | 16 | Quick COVID- 19 Severity Index, a machine-learning model, the COVID-19 Severity Index, Elixhauser mortality index, quick Sequential [Sepsis-related] Organ Failure Assessment | Respiratory rate, Oxygen flow rate, Pulse oximetry | CSI: 0.79(0.72-0.86); CURB-65: 0.50 (0.40–0.60); Elixhauser: 0.49 (0.26–0.74); qCSI: 0.82 (0.77–0.88); qSOFA: 0.83 (0.79–0.88) | CSI: 0.76(0.65-0.86); CURB-65: 0.64(0.42-0.89); Elixhauser: 0.61 (0.51–0.70); qCSI: 0.81 (0.73–0.89); qSOFA: 0.59 (0.50–0.68) | CSI: 73 (56-88); CURB-65: 57 (3.0 -97); Elixhauser: 82 (45-100); qCSI: 79 (63-93); qSOFA: 47 (6-66) | CSI: 81(72-89) CURB-65: 52 (18-100) Elixhauser: 42 (15-78); qCSI: 79 (71-87); qSOFA: 72 (64-100) | 36(25-47) | 96 (93-99) | LR+ 3.55(3.51-3.59); LR- 0.2(0.26,0.289) | NR | ✔ | Compared with the Elixhauser Comorbidity Index, quick Sequential SOFA and the CURB-65 pneumonia severity score. | - | Tool is available online |
|  | Han et al. (2022) | NR | NR | Prognostic, prediction model | CT images | NR | 0.93 | 77.5 | 94.8 | NR | NR | NR | NR | ✔ | ✔ | - | NR |
|  | Jiang et al. (2022) | 5-day, 15-day, and 30-day | 19 | Prognostic nomogram | age, CRP, fibrinogen, lactic dehydrogenase, NLR, urea, ALB-to-globulin ratio, and eosinophil count | NR | 0.86 | NR | NR | NR | NR | NR | NR | ✔ | ✔ | - | NR |
|  | Lee et al. (2022) | NR | 14 | KDDH (Keimyung University Daegu Dongsan Hospital) scoring system | Age, hemoglobin, CRP and LDH | NR | NR | NR | NR | NR | NR | NR | Dev.: 0.88 (0.83–0.93); validation: 0.83 (0.73–0.92) | ✔ | ✔ | - | Upon request |
|  | Leyderman et al. (2021) | 14 days | 2 | Biomarker | Serum ALB and daily urinary nitrogen excretion | NR | ALB: 0.516(0.397-0.634); nitrogen excretion: 0.624 (0.49-0.76) | ALB: 75 (57.8–87.9); nitrogen excretion: 75.9 (56.5–89.7) | ALB: 39.47 (24.0–56.6); nitrogen excretion: 58.1 (39.1–75.5) | NR | NR | NR | NR | Evaluation of the prognostic value | - | - | NR |
|  | Liu et al. (2021) | NR | 65 | NLR-LDH grading system | NLR-LDH | NR | NLR:0.89 (0.83-0.94); LDH: 0.87 (0.83-0.94); CRP: 0.72 (0.61-0.84); BNP: 0.68 (0.53-0.84) | NR | NR | NR | NR |  | NR | ✔ | ✔ | - | Upon request |
|  | Nadasdi et al. (2022) | NR | 1 | Biomarker | Serum circulating DPP4 activity | NR | 0.86 (0.80-0.91) | NR | NR | NR | NR | NR | NR | Investigation | - | - | NR |
|  | Nuevo‐Ortega et al. (2022) |  | 3 | A model composed of Age‐adjusted CDCI, CRB score and basal oxygen saturation | Age, CDCI, CRB scale, and baseline desaturation by pulse oximetry | NR | (a) 0.85 (0.80–0.89); (b) 0.90 (0.86-0.94) | NR | NR | NR | NR | NR | NR | ✔ | - | - | Available |
|  | Patel et al. (2021) | NR | NR | ROX Index (Ratio of Oxygen saturation) |  | NR | 0.77 | NR | NR | NR | NR | NR | NR | Assess ROX index | - | - | NR |
|  | Peng et al. (2022) | NR | 7 | Model considering incubation period along with clinical and lung CT features | Dyspnea, incubation period, number of comorbidities, DD, CRP and CT score | NR | DRM-COVID-19: 0.97 (0.95–0.99); incubation period: 0.68 (0.59–0.76); Clinical: 0.95 (0.92–0.98); CT scores: 0.89 (0.84–0.95) | DRM-COVID-19: 93; incubation period: 54.8; clinical: 88.7; CT scores: 80.6 | DRM-COVID-19: 93.8; incubation period: 75.1; clinical: 87.6; CT scores: 87.6 |  | NR | NR | NR | NR | ✔ | - | Upon request |
|  | Chang et al. (2022) | NR | NR | Nomogram model | Age, dyspnea, CRP, IL-6 and LYM count | NR | 0.86 | 72.1 | 86.4 | NR | NR | NR | 0.86 (0.84–0.88) | ✔ | ✔ | - | NR |
|  | Chen et al. (2022) | On admission | 21 | LASSO regression model | Age, comorbidities, computed tomography severity score, LYM count, AST and ALB. |  | 0.89 | 75.5 | 89.7 | NR | NR | NR | NR | ✔ | ✔ | ✔ | Upon request |
| **Outcome: Mortality and ICU admission** | | | | | | | | | | | | | | | | | |
|  | Ak et al. (2021) | NR | NR | BCRSS; qCSI | BCRSS: difficulty breathing, respiratory rate; SpO_2_, chestX-ray  ; qCSI: HFNT, respiratory rate and pulse oximetry | NR | In-hospital mortality (BCRSS: 0.80; qCSI: 0.85; ICU admission; BCRSS: 0.84; qCSI: 0.85) | NR | NR | NR | NR | NR | NR | Comparison | - | - | NR |
|  | Arnold et al. (2021) | 28 days | NR | Biomarkers and NEWS2 | CRP, Complete blood count, renal profile, LDH, PCT, IL-6, KL-6, ferritin, troponin, NT-pro-BN), suPAR, NEWS2, Radiographic severity score, CURB-65 | NR | NEWS2: 0.70 (0.60-0.79); Age: 0.70 (0.62- 0.77) | NEWS2: 95; Age: 95 | NEWS2: 88; Age: 88 | NR | NR | NR | NR | Comparing biomarkers | - | - | Code available |
|  | Covino et al. (2020) | 48 hours and 7 days | NR | NEWS (most accurate predictor of ICU admission); REMS (most accurate predictor of death) | Different scores | NR | NEWS: 0.78 (0.74-0.83); REMS: 0.82 (0.78-0.86) | NEWS: 71.4 (57.8-82.7); REMS: 96.1 (80.4-99.9) | NR | NR | NR | NR | NR | Comparison | - | - | NR |
|  | García- Clemente et al. (2020) | NR | NR | Multivariate logistic regression models; PSI; CURB-65; MuLBSTA; SMART-COP | Age, LYM, confusion and NTproBNP | NR | Mortality (PSI score: 0.87 (0.81-0.94); CURB-65: 0.85 (0.79-0.91)); ICU admission (best performance: SMART-COP: 0.75 (0.70-0.82); MuLBSTA score = 0.78 (0.71-0.84)) | NR | NR | NR | NR | NR | NR | ✔Analyzing prognostic scores | - | - | NR |
|  | Kurt et al. (2021) | NR | NR | Shock Index (SI) and Modified Shock Index (MSI) | Chest x-rays | NR | In-hospital mortality (SI: 0.72; MSI: 0.74); ICU (SI: 0.70; MSI: 0.73) | In-hospital mortality (SI: 71.2; MSI: 74); ICU (SI: 61.2; MSI: 65.5) | In-hospital mortality (SI: 69.6; MSI: 72.4); ICU (SI: 78.3; MSI: 78.9) | In-hospital mortality (SI: 30.4; MSI: 33.3); ICU requirement (SI: 60.8; MSI: 63.2) | In-hospital mortality (SI: 92.8; MSI: 93.7); ICU (SI: 78.5; MSI: 80.6) | NR | NR | ✔Investigation of accuracy | - | - | NR |
|  | Lazar Neto et al. (2021) | 30 days | NR | Severity Index (PSI), CURB, CURB-65, qSOFA, Infectious Disease Society of America and American Thoracic Society Mi-r Criteria, REA-ICU, SCAP, SMART-COP, CALL, COVID GRAM and 4C | Not applicable due to simple comparison of different scores | NR | PSI: 0.79 (0.77-0.82); 4C: 0.78 (0.75-0.81); COVID GRAM: 0.77 (0.75-0.80); CURB-65: 0.74 (0.72-0.77) | NR | NR | NR | NR | NR | NR | - | - | ✔ | Upon request |
|  | Levine et al. (2021) | 14 days | NR | Simple score | Age, SO_2_, ALB | NR | NR | 83.2 | 82.2 | NR | NR | NR | Dev.: 0.89 (0.87-0.92); Validation: 0.87 (0.81-0.93) | ✔ | ✔ | - | NR |
|  | Li X et al. (2020) | NR | NR | Deep learning model | ICU: PCT, LDH, CRP, ferritin and oxygen saturation; Mortality: age, LDH, PCT, cardiac troponin, CRP and oxygen saturation. | NR | ICU admission: 0.78 (0.76–0.79); Mortality: 0.84 (0.84–0.85) | NR | NR | NR | NR | NR | NR | ✔ | ✔ (fold cross-validation) | - | Some data available in the supplemental files. |
|  | Pokeerbux et al. (2021) | NR | NR | NEWS score | Respiration rate, oxygen saturation, supplemental oxygen, systolic BP, pulse rate, level of consciousness and temperature | NR | 0.68 (0.60-0.77) | 52.3 (40.2-64.5) | 78.8 (72.0-85.7) | 54 (41.7-66.3) | 77.7 (70.8-84.6) | NR | NR | Evaluation | - | - | NR |
|  | Ponsford et al. (2021) | 28 days | NR | Laboratory markers | CRP, ALB, urea, NLR, creatinine, age, sex and WIMD | NR | Mortality: 0.79 (0.67-0.91); 28-day mortality or ICU): 0.70 (0.56-0.84) | NR | NR | NR | NR | NR | NR | ✔ | ✔(cross-validation) | - | Upon request |
|  | Rasyid et al. (2021) | NR | NR | Biomarkers (Ferritin and NLR) | Age, Ferritin, NLR levels | NR | ICU (Ferritin: 0.72 (0.64–0.80); NLR: 0.78 (0.69–0.86)); Mortality (Ferritin: 0.70 (0.59–0.81); NLR: 0.76 (0.67–0.86)) | ICU (Ferritin: 70.5; NLR:79.5); Mortality (Ferritin: 69; NLR: 67.7 | ICU (Ferritin: 67; NLR: 63.5); Mortality (Ferritin: 63.; NLR: 68.9) | ICU (Ferritin: 78.8; NLR: 81.6; Mortality (Ferritin: 78.9; NLR: 70.5 | ICU (Ferritin: 92.7; NLR: 91.3); Mortality (Ferritin:95.1; NLR: 94.9) | NR | NR | Evaluation | - | - | Upon request |
|  | Rodriquez-Nava et al. (2021) | NR | NR | (a). CURB-65; (b) BCRSS; (c) qCSI | BCRSS; Brescia-COVID Respiratory Severity Scale; CURB-65; confusion, BUN; qCSI; Quick COVID-19 Severity Index | NR | In- hospital mortality (a) 0.78 (0.73-0.83); (b) 0.66 (0.61-0.72); (c) 0.71 (0.66-0.76); ICU admission; (a) 0.63 (0.57 -0.68); (b) 0.74 (0.68-0.78); (c) 0.76 (0.68-0.78) | NR | NR | NR | NR | NR | NR | - | - | ✔ (validation study) | Upon request |
|  | Shi S et al. (2021) | NR | 11 | Pneumonia severity index (PSI); Confusion-Urea-Respiratory Rate-BP-65 (CURB-65) | Multivariate analysis: NLR, IL-6 | NR | NLR: 0.88; IL-6: 0.85; PSI score: 0.82; CURB-65 score: 0.78 | NR | NR | NR | NR | NR | NR | Investigation of biomarkers | - | - | NR |
|  | Van Dam et al. (2021) | 30 days | NR | RISE UP score (Risk Stratification in the Emergency Department in Acutely ill Older Patients) | Age, sex and information regarding comorbidities according to the CDCI | NR | 30-day mortality: 0.77 (0.73-0.81); Composite outcome (30-day mortality and ICU admission):.72 (0.68 to 0.76) | NR | NR | NR | NR | NR | NR | ✔ | - | ✔ | Upon request |
|  | Zhao Z et al. (2020) | NR | NR | Risk scores | Heart failure, PCT, LDH, COPD, SaO_2_, heart rate and age | NR | ICU admission: 0.74 (0.63–0.85); Mortality: 0.82 (0.73–0.92) | ICU :10.5; Mortality: 7.1 | ICU :99.2; Mortality: 100 | NR | NR | NR | NR | ✔ | ✔ | - | Upon request |
|  | Adderley et al. (2022) | Within 28 days from admission | 63 | ISARIC 4C score | Age, breathlessness, sputum, systolic BP, temperature, respiratory rate, oxygen saturation, FiO_2_, alkaline phosphatase, CRP, corrected calcium, eosinophils, GLU, pH, urea, WBC count, platelets and frailty score. | NR | Dev. (mortality: 0.78 (0.74-0.81); ICU: 0.89 (0.86-0.92); Ext. validation (mortality: 0.77 (0.75-0.78); ICU: 0.81 (0.79-0.83) | NR | NR | NR | NR | NR | NR | ✔ | - | ✔ | No data available |
|  | Aguadero et al. (2021) | Day 9 following hospitalization | 2 | Biochemical markers | LDH | NR | 0.92 (0.84-1.00) | 80 | 92 | 80 | 92 | NR | NR | Analysis of biochemical markers | - | - | NR |
|  | Regolo et al. (2022) | NR | NR | Inflammatory biomarkers | NLR, PLR and CRP | NR | NLR: 0.77 | NLR:72.9 | NLR:71.9 | NR | NR | NR | NR | Assessing biomarkers | - | - | Upon request |
|  | Rizzi et al. (2022) | At baseline and at day 7 | NR | Prognostic biomarkers | RDW-CV, IP-10, DD, platelet count, PiO_2_/FiO_2_, age, sex, NEWS2 | NR | IP-10 (at baseline) and of CRP (day 7): 0.77 | 87 and 83 | 86 and 73 | NR | NR | NR | NR | Identifying biomarkers | - | - | Upon request |
|  | Usul et al. (2021) | 28 days | NR | qSOFA score | This scale uses the following 3 criteria, assigning a score each for low BP (SBP ≤100 mmHg), high respiratory; rate (22 breaths per minute), or altered mentation (GCS <15). The score ranged from 0 to 3. | NR | ICU :0.94; 28-day mortality: 0.96 | ICU: 97.4; 28-day mortality: 84.6 | ICU admission: 85; 28-day mortality: 90.4 | NR | NR | NR | NR | Assessment of qSOFA score | - | - | NR |
|  | Wilfong et al. (2021) | NR | NR | APACHE-II score | NR | NR | ICU: 0.85 (0.79-0.92) | NR | NR | NR | NR | NR | NR | Assessment of scores | - | - | Data available with agreement |
|  | Aznar-Gime- et al. (2021) | 30 days | 165 | ITAIN-VA / COVID_IIS | 20 | Dev.: NR; Cut-off 0.4 (Ext. valid.):76 | Dev. Final model (xBoost): 0.83 (0.78-0.87); Ext. validation: 0.82(0.79-0.85) | Dev.: NR; Cut-off 0.4 (ext. validation): 71 | Dev.: NR; Cut-off 0.4 (ext. validation):7 | Dev.: NR; Cut-off 0.4 (ext. validation):60 | Dev.: NR; Cut-off 0.4 (ext. validation):85 | NR | NR | ✔ | ✔ | ✔ | NR |
|  | Subudhi et al. (2021) | NR | NR | 18 algorithms | ICU admission: CRP, NEU percentages, LDH and first respiratory rate; Mortality: DD and initial SaO_2_. | CD | ICU: (RFC): 0.88 and (Logistic): 0.86; Mortality: (RFC): 0.93 and (logistic): 0.84 | NR | NR | NR | NR | NR | NR | ✔ | ✔ | ✔ | Restricted |
|  | Ak et al. (2021) | 30 days | 1 | Shock index (SI) | 1 | NR | 30-day mortality: 0.67 (0.62-0.72); ICU: 0.75; (0.7-0.79) | 30-day mortality: 83.6; ICU: 98.7 | 30-day mortality: 50.8; ICU: 50.5 | 30-day mortality: 42.4; ICU:34.1 | 30-day mortality: 87.8; ICU:99.3 | NR | NR | - | - | ✔ | NR |
|  | Klaveren et al. (2021) | 28 days following admission | 22 | COPE | Age, RR, LDH, CRP, ALB and urea | NR | In-hospital mortality Dev.: Hospital 1: 0.82 (0.78-0.86); H2:0.82 (0.74-0.90); H3: 0.79 (0.70-0.88); H4: 0.83 (0.79-0.86); Validation: H1-0.82, H2-0.85, H3-0.88, H4-0.85. ICU admission Dev.: H1: 0.84 (0.78-0.90), H3:0.81 (0.66-0.95); validation: H1-0.66, H3-0.74. | Mortality: Threshold (T) 10%-76; T 5%-93; T 20%-49; ICU: NR | Mortality: Threshold (T) 10%-24; T 5%-49; T 20%-89; ICU: NR | NR | NR | NR | NR | ✔ | ✔ | - | NR |
|  | Ganesan et al. (2021) | NR | NR | Prognostic model | CDCI, LDH, NLR, DD and SOFA score | 78 | 0.88 (0.81-0.93) | NR | NR | NR | NR | NR | NR | ✔ | - | - | NR |
|  | Ahmed et al. (2022) | within 7 days of admission | 5 risk scores were evaluated | NEWS2 score | respiratory rate, SaO_2_, systolic BP, pulse rate, level of consciousness or new-onset confusion and temperature | NR | 0.68 | 80.1 | 50.7 | 38.8 | 86.7 | NR | NR | - | - | ✔ | NR |
|  | Hormanstorfer et al. (2021) | NR | NR | NR | Age, need for supplemental oxygen | NR | NR | NR | NR | NR | NR |  | NR | ✔ | - | - | NR |
|  | Zahedin Kheyri et al. (2022) | 30 days | 11 | Biomarkers | Age, sex, comorbidities, treatments, NLR | 62.72 | NR | 70.2 | 60.2 |  |  | Mortality: NLR: 2.6(1.5-4.4); ICU admission: 2.9(1.8-4.6) | NR | Investigation | - | - | NR |
|  | Jibril et al. (2022) | 24 hrs | NR | COVID GRAM score, SOFA |  | NR | Critical illness: 0.80(0.75–0.85) | NR | NR | NR | NR | NR | NR | ✔ | ✔ | - | NR |
|  | Munera et al. (2022) | NR | 16 | Deep- learning model | ICU admission: age, fraction of inspired oxygen (FiO_2_) on admission, dyspnea on admission and obesity. Mortality: age, FiO_2_ on admission and dyspnea. | NR | ICU: 0.92 (0.91–0.92); Mortality: 0.81(0.81–0.82) | ICU:91; mortality:74 | ICU: 78; mortality:75 | ICU: 89; mortality:58 | ICU: 83; mortality:85 | NR | NR | ✔ | ✔ | - | NR |
|  | Prasetya et al. (2021) | 30 days | NR | Inflammatory markers | CRP, NLR, d-NLR, and PLR | NR | CRP:0.85 (0.78–0.91); NLR:0.85 (0.78–0.92), d-NLR: 0.85 (0.79–0.92); PLR:0.70 (0.62–0.78) | CRP: 64.8; (50.6–77.3); NLR: 75.9; (62.4–86.5); d-NLR: 77.8; (64.4–88.0); PLR: 53.7; (39.6–67.4) | CRP: 89.0; (85.2–92.2); NLR: 88.7; (84.9–91.9); d-NLR: 87.2; (83.2–90.6); PLR: 83.4; (79.0–87.2) | NR | NR | NR | NR | Investigation of inflammatory markers | - | - | NR |
|  | Asmarawati et al. (2022) | Upon admission & day 5 | qSOFA, SOFA, APACHE II, and NEWS-2 on admission and day 5 | Disease severity scores | qSOFA, SOFA, APACHE II, and NEWS-2 on admission and day 5 | NR | AUROC of mortality =The initial NEWS-2 revealed a higher AUC value than the qSOFA, APACHE II, and SOFA score in estimating mortality (0.867; 0.83; 0.822; 0.794).; | 95.7 | 86.7 | NR | NR | NR | NR | Assessment of scores | - | - | NR |
|  | Brook et al. (2022) | admission | NR | 4C Deterioration and DL Poor Scores | 4C Score | NR | 0.79 (0.68–0.90) | NR | NR | NR | NR | NR | NR |  | - | Ext. validation of 4C Deterioration score | NR |
|  | Ceci et al. (2022) | ED admission | CD | Routine laboratory tests | AST, ALT, LDH, MGB, CK, CRP, Ferritin, and DD | NR | CRP deceased vs emergency: **0.98 (0.94–1.0)** | NR | NR | 0.96 | NR | NR | NR | Assessment of laboratory biomarkers | - | - | Upon request |
| **Outcome: ICU admission (only)** | | | | | | | | | | | | | | | | | |
|  | Bellos et al. (2021) | 5 days (of hospital stay) | NR | A10-variable model | Patients’ sex, presence of hypertension and DM, fever, short-ness of breath, serum GLU, AST, LDH, CRP and fibrinogen | NR | **0.97** | 92.3 | 93.3 | NR | NR | NR | 0.86 | ✔ | ✔ (cross-validation) | - | NR |
|  | Bastug et al. (2020) | NR | 21 | Blood parameters | %LUC, NLR, DD and CRP | NR | DD: 0.89; CRP: 0.87; NLR: 0.86 | NR | NR | NR | NR | NR | NR | ✔ | - | - | NR |
|  | Paranjape et al. (2021) | NR | NR | Prediction model (probability score) | DM, CAD, CKD, CRP, and serum LDH | NR | Dev. set: 0.75; Validation set: 0.77 | NR | NR | 34 | 94 | NR | NR | ✔ | ✔ | - | Part of the data are available in the manuscript |
|  | Hachim et al. (2020) | NR | NR | 3 markers: DD, troponin, urea level) | Validation of the 3 predictors (DD, troponin and Urea level) | NR | NR | 30.3 (22.2–39.4) | 93.1 (88.3–96.4) | 4.4 (2.4–8.1) | 75 (0.7–0.9) | NR | NR | ✔ | ✔ | - | Upon request |
|  | Cheng Fu-Yuan et al. (2020) | NR | 31 | Machine learning (Random Forest classifier) | Respiratory rate, WBC count, LYM count, diastolic BP, CRP, oxygen saturation by pulse oximetry, age, temperature, pulse, QRS duration, BUN, serum Na, T wave axis, anion gap, systolic BP, PR interval, R wave axis, RBC count, serum Ca, serum ALB | 76.2 (74.6–77.7) | Dev. Set: 0.79 (0.75–0.85): Test set; 0.94: Dev. set | 72.8 (63.2–81.1) | 76.3 (74.7–77.9) | NR | NR | NR | NR | ✔ | ✔ (cross-validation) | - | NR |
|  | Statsenko et al. (2021) | NR | NR | Predictive biomarkers (predictors) | WBC, LYM count, total bilirubin, ALT, AST, DD, aPTT, CK, CRP, LDH, troponin, ferritin and fibrinogen on admission | NR | Top valuable tests (aPTT, CRP and fibrinogen): 0.86 (0.49-0.88); Model trained with all the tests: 0.9 (0.81-0.9) | NR | NR | NR | NR | NR | NR | ✔ | ✔ | - | Upon request |
|  | Zhou Y et al. (2020) | NR | NR | Nomogram | Age, respiratory rate, systolic BP, smoking status, fever, and CKD | NR | NR | NR | NR | NR | NR | NR | Dev.: 0.83; Validation: 0.78 (0.68–0.87) | ✔ | - | ✔ | Upon request |
|  | Shanbehzadeh et al. (2022) | NR | 20 | DT (Decision Tree) algorithms | Clinical predictors | 81.9 | Dev. J-48 algorithm :0.85; Validation J-48: 0.82 | 92.4 | 65.9 | 80.5 | 85 | NR | NR | ✔ | ✔ | ✔ | NR |
|  | Suastika et al. (2021) | NR | NR | Biomarker | Eosinophil count | NR | Absolute and percentage eosinophil count :0.66 (0.50–0.82) and 0.74 (0.61–0.87), respectively | 77.7 and 78.3 | 50 and 57.1 | NR | NR | NR | NR | Determination of diagnostic value of eosinophil | - | - | NR |
|  | Hashem et al. (2021) | NR | NR | Laboratory biomarkers | Anemia, NLR, platelet-to-LYM ratio, and DD on admission | NR | DD level above 0.9 mg/l had the best value to predict ICU admission for COVID-19 patients: 0.74 | 72.4 | 68.9 | NR | NR | NR | NR | Assessment of laboratory biomarkers | - | - | ✔ |
|  | Heo et al. (2021) | NR | NR | Integer-based scoring system | Age, sex, body temperature, dyspnea, hemoptysis, history of CKD, and activities of daily living scale | NR | Validation set: 0.88 (0.85–0.91) | NR | NR | NR | NR | NR | NR | ✔ | - | ✔ | Available in Korean |
|  | Durmus Kocak et al. (2021) | NR | NR | Selected biomarkers | DD and mean platelet volume (MPV) combination | Combination: 63 | Serum DD levels: 0.71 (0.62–0.79); MPV: 0.69 (0.59–0.80), | Combination: 57.7 | Combination: 70.8 | Combination: 32 | Combination: 84 | NR | NR | Investigation of efficacy of prognostic markers | - | - | ✔ |
|  | Guner et al. (2021) | NR | 35 | Nomogram | Saturation, LDH, CRP, PCT, Troponin | NR | 0.93 (0.90–0.95) | NR | NR | NR | NR | NR | 0.91; (0.90–0.95) | ✔ | ✔ | - | The anonymized datasets generated and/or analyzed during the current study are not publicly available due to Turkey’s Personal Data Protection Law, but can be upon reasonable request. |
|  | Huespe et al. (2021) | 24, 48 h and on admission | NA | COVID-19 Severity index, NEWS2 & NEWS-C | NA | NR | NEWS2: 24 h: 0.73 (0.68-0.78), 48 h: 0.71 (0.65-0.77) and on admission 0.52 (0.47-0.57); NEWS-C: 24h- 0.74 (0.70-0.79), 48h-0.72 (0.68-0.79) and on admission 0.56 (0.52-0.61); COVID-19 Severity Index: 24 h-0.80 (0.77-0.84), 48h-0.79 (0.75-0.84) and on admission 0.61 (0.58-0.66) | NEWS2 (≥7): 80; NEWS-C (≥7): 30.2 and COVID-19 severity index (≥12): 39.7 | NEWS2 (≥7): 99.8; NEWS-C (≥7): 99.5 and COVID-19 severity index (≥12): 99.8 | NR | NR | NEWS2 (≥7)-LR+: 4.77 and LR-: 0.99 NEWS-C (≥7)-LR+: 6.36 and LR-: 0.97 and COVID-19 severity index (≥12)-LR+: 15.77 and LR-: 0.96 | NR | - | - | ✔ | NR |
|  | Geraili et al. (2022) | 3, 6, 9 & 12 days prior to ICU admission | 24 | Time-dependent ROC curves for ICU admission | CRP and NLR | NR | CRP: Day 3-0.74 (0.66–0.82); Day 6-0.72 (0.64–0.80); Day 9-0.67 (0.59–0.75); Day 12-0.67 (0.59–0.75); NLR: Day 3-0.69 (0.61–0.77), Day 6-0.67 (0.58–0.75); Day 9-0.66 (0.57–0.74); Day 12-0.58 (0.50–0.67) | CRP: D3 -81.7, D6-76.3. D9-80.4 and D12- 82.9; NLR: D3-71.5, D6-66.5, D9-61.5; 0.730 | CRP: D3-60.7, D6-62.8, D9-45.5, D12-56.5; D3-62.5, D6-61.8, D9-57.4, D12-40.6 | NR | NR | NR | NR | ✔ | - | - | NR |
|  | Huang et al. (2021) | NR | 47 | Random forest (RF) algorithm | NLR, age, LDH, creatinine, GLU and ALB | 91 | 0.94 | 88 | 93 | NR | NR | NR | NR | ✔ | ✔ | ✔ | Additional data are available |
|  | Ketenci et al. (2022) | NR | 10 | Binary logistic regression model | NEU, lymphocyte, PLT, CRP, PCT, NLR, and SII variables | NR | Age: 0.70; NEU: 0.44; Systemic immune-inflammation index (SII):0.58; NLR:0.65; CRP:0.61 | Age: 68.7; NEU: 45.5; SII:59.5; NLR:60.6; CRP:59.7 | Age: 63.9; NEU: 45.5; SII:59.5; NLR:61.0; CRP:59.3 | NR | NR | Age: 1(1-1.1); NEU: 1(1-1.1); CRP: 1(1-1); NLP: 1(1-1.1), SII: 0.99(0.99-0.99) | NR | ✔ | ✔ | - | NR |
| **Outcome: MV** | | | | | | | | | | | | | | | | | |
|  | Kulkarni et al. (2021) | NR | NR | Deep learning algorithm | NR | 84.7 | 0.90 | 86.3 | 84.4 | 55.9 | 86.4 | NR | NR | Evaluation | - | ✔ | Upon request |
|  | Amezcua-Guerra et al. (2021) | NR | NR | Inflammation-based risk scoring system | Serum levels of DD, ferritin, cTnI, and IL-6 | NR | 0.73 | NR | NR | NR | NR | NR | NR | Investigation | ✔ | - | NR |
|  | Garcia-Gordillo et al. (2021) | NR | NR | COVID-IRS-NLR’s (IRS: Intubation Risk Score) | Predictive scores, one based on IL-6 and the other one on NLR, using the following variables: respiratory rate, SpO_2_/FiO_2_ ratio and LDH, respiratory rate, IL-6 or NLR | NR | Internal validation: 0.88 (0.82–0.93)-using NLR based score; 0.89 (0.84–0.94)-using IL-6 based score; Ext. validation; 0.82 (0.76–0.89)-using NLR based score; 0.83 (0.76–0.89)-using IL-6 based score | NR | NR | NR | NR | NR | NR | ✔ | ✔ | ✔ | Databases available (link in the manuscript) |
|  | Alberdi-Iglesias et al. (2021) | Follow- up period up to 120 days | NR | SpO_2_/FiO_2_ and the ROX indices | Pulse oximetry saturation, fraction of inspired oxygen, and respiratory rate | NR | SpO_2_/FiO_2_ ratio: 0.80 (0.75–0.86) | NR | NR | NR | NR | NR | NR | Analyze the predictive ability of SpO_2_/FiO_2_ and the ROX indices | - | ✔ | Upon request |
|  | Wen Li et al. (2022) | NR | NR | Biomarkers | Glucocorticoid, NEU count, LDH and PCT | NR | 0.89 (0.84–0.93); 0.89 (0.83–0.95) | 90.9; 96.4 | 77.7; 70.3 | NR | NR | NR | NR | Identification of predictors | - | - | NR |
|  | Ashkenazi et al. (2022) | NR | NR | NEWS Score, physiobiological and clinical parameters | NEWS Score, arterial hypertension, T2DM, Protein, Calcium, LDH, DD, CRP | NR | 0.92 | 81 | 89 | 37.3 | 98.4 | NR | NR | ✔ | - | - | NR |
| **Outcome: Combined outcomes (mortality, intubation, MV, ECMO, ICU admission, HFNT)** | | | | | | | | | | | | | | | | | |
|  | Arvind et al. (2020) | 3.1 days from admission | NR | Machine learning model | PH, CRP, heart rate, respiratory rate, temperature, O_2_ saturation, PaO_2_, PaCo_2_, HCO_3_, DD, Creatinine, WBC, Platelets, systolic pressure, and diastolic pressure. | NR | 0.84 | NR | NR | NR | NR | NR | NR | ✔ | ✔ | - | Upon request |
|  | Youssef et al. (2021) | 24 hours | NR | Early Warning Score (EWS) based on a Gradient Boosting Trees (GBT) algorithm | EWS Scores, vital signs, consciousness level, oxygen support and laboratory markers | 70 | 0.94 | 90 | 70 | NR | NR | NR | NR | ✔ | ✔ (cross validation) | - | NR |
|  | Jimenez‐Solem et al. (2021) | NR | NR | Machine learning | Age, sex, BMI, Comorbidities and laboratory tests | NR | ICU admission: 0.80; MV: 0.82; Death: 0.90; Ext. validation; ICU admission: 0.53; Mortality: 0.74 | NR | NR | NR | NR | NR | NR | ✔ | ✔ (cross-validation) | ✔ | NR |
|  | De Alencar et al. (2021) | NR | NR | LUS (Lung Ultra Sound) | Age and lung involvement on chest CT | NR | Death: 0.72; Endotracheal intubation: 0.76; ICU admission: 0.71 | NR | NR | NR | NR | NR | NR | ✔ | - | - | Upon request |
|  | Yu L et al. (2021) | NR | NR | Machine learning models (XGBoost and catBoost) | Age, temperature, SpO2, BMI, diastolic BP, Respiratory Rate, Pulse, Systolic BP, Sex, DM, Smoking, Race, Alcohol use, Cardiovascular disease, and pulmonary diseases | MV: 86.2 Mortality: 80.3 | MV: 0.68 | NR | NR | Mortality: 79. | NR | NR | NR | ✔ | ✔ (cross-validation) | ✔ | Available (link in the manuscript) |
|  | Zhou J et al. (2021) | NR | NR | Clinical risk score | Age, sex, comorbidities, medications and laboratory examination results | internal: 83 (80-86); external – 87 (85-91) | Hong Kong (internal): 0.86 (0.82–0.91); Wuhan (external): 0.89 (0.85–0.93) | NR | Internal-85 (83-89); external-88 (85-92) | NR | NR | NR | NR | ✔ | ✔ (cross-validation) | ✔ | Upon request |
|  | Gresser et al. (2021) | Median time from hospital admission to ECMO placement: 1.4 days (IQR 0.2–4.0) | NR | SOFA score on admission and lung involvement according to CT (%) | Age, sex, BMI, SOFA score on admission, LDH on admission and percentage of lung involvement on admission | NR | 0.91 (0.84–0.97) | 93 | 57 | NR | NR | NR | NR | Evaluation of clinical parameters | - | - | Upon request |
|  | Gude et al. (2020) | NR | NR | DALSH | T2DM, Age, Lymphocyte count, SaO2, and pH | NR | 0.87 (0.81-0.92) | NR | NR | NR | NR | NR | NR | ✔ | ✔ | - | NR |
|  | Aguirre-García et al. (2022) | Median follow-up; time was 8 (3-11) days | NR | The R4 score | Age, eGFR, room air SpO2, comorbidities | NR | 0.72 | NR | NR | NR | NR | NR | NR | ✔ | - | - | NR |
|  | Alessandri et al. (2022) | NR | 20 covariates | CPAP Failure Score (CPAP-FS) | SpO_2_, P/F ratio, Call Score, a pre-existing chronic lung disease, age, male sex | NR | Dev.: 0.87; Internal validation: 0.76–0.80 | NR | NR | NR | NR | NR | NR | ✔ | ✔(cross-validation) | - | Upon request |
|  | Shanbhag et al. (2021) | NR | NR | Age-adjusted CDCI | Age, sex, age-adjusted CDCI, CDCI score, IMV, length of stay, outcome of hospital stay | NR | Mortality: 0.71; IMV requirement: 0.69; LOS: 0.45 | Mortality: 68; IMV: 67; LOS: 38.7 | Mortality: 62; IMV: 63; LOS: 53.3 | NR | NR | NR | NR | ✔ | - | - | NR |
|  | Venturini et al. (2022) | Outcome within 30 days | NR | acute Pneumonia early assessment (aPNea) score model | Evaluating respiratory rate, arterial oxygen saturation, need for oxygen support, systolic BP, heart rate, consciousness by the Acute, Confusion, Voice, Pain, Unresponsive scale, and body temperature | NR | (Retrospective) Dev. cohort: 0.86 (0.78–0.93); (Prospective) Validation cohort: 0.79 (0.73–0.84) | NR | NR | NR | NR | NR | NR | ✔ | - | ✔ | Upon request |
|  | Downing et al. (2021) | first 48 h of admission and over the course of hospitalization | NR | ROX Index | CD | NR | ROC index at 24 hrs.: 0.81 (0.71-0.88); ROX Index at triage: 0.84 (0.75-0.9) | NR | NR | NR | NR | NR | NR | Identification of predictors | - | - | NR |
|  | Gorgojo-Galindo et al. (2021) | NR | NR | Multivariate predictive model | GLU, creatine, leukocytes, LYM, NEU, PCT, CRP, ferritin, DD, LDH, Growth Factor (HGF), IL-15, Monocyte Chemoattractant Protein-1, Human Platelet-Derived Growth Factor-BB | CD | **0.94** | 91.7 | 95 | NR | NR | NR | NR | ✔ | ✔ (Leave One Out Cross-Validation) | - | Upon request |
|  | Torres-Macho et al. (2021) | 48 h after admission and 72-96 h after first examination | NR | Serial lung ultrasound (LUS): SCORE 1 & SCORE 2 | Two Ultrasound examination: first one within 48 h following hospital admission (SCORE-1) and the second on day 3 or 4 (72–96 h) after the first examination (SCORE-2) | NR | SCORE 2: 0.72 (0.58–0.85); SCORE 1: 0.61 (0.52–0.7) | NR | NR | NR | NR | NR | NR | Analyze the prognostic accuracy of serial lung ultrasound | - | - | NR |
|  | Varghese et al. (2021) | NR | NR | Machine learning algorithms | Evaluation of chest X-ray -based imaging metrics | NR | Adaboost-based radiomics for: Intubation: 0.72 (0.63-0.81); Death: 0.71 (0.58-0.84); ICU: 0.61 (0.49-0.67) | NR | NR | NR | NR | NR | NR | ✔ | ✔ | - | NR |
|  | Tevald et al. (2021) | CD | NR | Score and predictive model | Age, sex, ethnicity, hospital, length of stay, cohort, COVID-19, MV, ICU, insurance, BMI, CDCI | NR | Discharge: 0.85 (0.81-0.88); Length of stay:0.66 (0.61-0.71); Mortality: 0.68 (0.59-0.76) | Discharge:79; Length of stay:70; Mortality: 64.7 | Discharge:77.9; Length of stay:59.3; Mortality: 64.7 | NR | NR | NR | NR | ✔ | - | - | NR |
|  | Huang Dong et al. (2021) | NR | 14 | BAR-centered and nomogram | Age, unconsciousness, respiratory rate, LYM, bilirubin, BAR | NR | 0.82 (0.78-0.86) | 69 | 78.6 | 22.5 | 96.6 | 0.39 | 0.9 | ✔ | - | - | Upon request |
|  | Aly et al. (2021) | NR | 15 | Hematological predictive model | Age, NLR, CRP, DD, and oxygen aid upon entry | NR | NLR: 0.84 (0.80-0.87); d-NLR:0.82(0.78-0.85); PLR: 0.64(0.60-0.69); LMR:0.29 (0.24-0.33) | NLR: 71.38; d-NLR:67.2; PLR: 50; LMR:67.6 | NLR: 87; d-NLR:89.2; PLR: 77.6; LMR:65.9 | NLR: 90.2; d-NLR:91.3; PLR: 78.7; LMR:76.8 | NLR: 64.4; d-NLR:61.8; PLR: 48; LMR:55 | NLR:33; d-NLR:37; PLR: 65; LMR:49 | NR | ✔ | - | - | NR |
|  | Pournazari et al- (2021) | NR | 51 | Optimized prediction model | Age, BMI, presence of respiratory disease, presence of renal disease, BNP, IL-6, CRP, ferritin, stroke volume index, free-wall right ventricular strain, total right ventricular strain, left atrial pump strain, LV GLS, right atrial pressure, pulmonary artery systolic pressure, and average E/eʹ ratio | NR | Mortality: 0.91(0.85-0.96); Mortality + ECMO: 0.90 (0.85-0.96) | NR | NR | NR | NR | NR | NR | ✔ | - | - | Upon request |
|  | Valencia et al. (2021) | After 2 h of HFNT | NR | HACOR and ROX scales | NA | NR | ROX: 0.72 (0.66-0.79); HACOR: 0.71 (0.65-0.78) | ROX: 62; HACOR: 66 | ROX: 65; HACOR: 65 | NR | NR | NR | NR | - | - | ✔ | NR |
|  | Lombardi et al. (2021) | 30-day in-hospital mortality and the composite of 30-day in-hospital mortality or ICU transfer | Ext. validation of 32 different scores | 32 different prognostic scores | 7 different scores (ANDC, COVID-19 SEIMC, Coronation-TR, 4C-mortality score, ABCS, COVID-GRAM, RISE UP) with various number of predictors | NR | In-hospital mortality [4C Mortality Score: 0.79 (0.78–0.80); ABCS:0.79 (0.78–0.80); COVID-GRAM: 0.77 (0.76–0.78); RISE UP: 0.77 (0.76–0.78); CORONATION-TR: 0.77 (0.76–0.78); ANDC: 0.76 (0.75–0.77): COVID-19 SEIMC:0.75 (0.74–0.76) and In-hospital mortality or ICU admission [4C Mortality Score: 0.66 (0.65–0.67); ABCS: 0.68 (0.67–0.69); COVID-GRAM: 0.69 (0.68–0.69); RISE UP: 0.66 (0.65–0.67); CORONATION-TR: 0.72 (0.71–0.73); ANDC: 0.64 (0.63–0.65); COVID-19 SEIMC: 0.59 (0.58–0.60) | 4C Mortality Score: 21.5 (19.6-23.4); ABCS: 88.2 (86.7-89.7); RISE UP: 50.8 (48.2-53.4); ANDC: 63.4 (61.1-65.7); COVID-19 SEIMC: 78.0 (76.3-79.7) | 4C Mortality Score: 96.8 (96.4-97.2); ABCS: 51.2 (49.6-52.7); RISE UP: 84.0 (83.1-84.9); ANDC: 73.4 (71.9-74.9); COVID-19 SEIMC: 61.0 (60.1-61.9) | NR | NR | NR | NR | - | - | ✔ | Authorized access to staff |
|  | Faria et al. (2021) | NR | 42 | Intelligent optical fingerprinting of blood samples with a stacking ensemble machine learning model | Backscattered signal features (optical fingerprint), patient comorbidities and age | 79.6 (75.8–83.0) | 0.80 (0.76–0.84) | 85.7 (81-89.6) | 72.6 (66.4–78.2) | 78.3 (73.2–82.8) | NR | NR | NR | ✔ | ✔ | - | Upon request |
|  | Hiremath et al. (2021) | NR | CD | CIAIN Nomogram | AIP, LDH, PT, ALB, AST, LYM | Dev.: 77.9, valid.: 77.9; | Dev.: 0.86 valid.: 0.84 (0.79-0.89) | Dev.: 80.71, valid.: 90.6 | Dev.: NR valid.: 61.4 | Dev.:69.3, valid.: NR | Dev.:NR; valid: NR | Dev.: NR; valid: NR | Dev.: NR; valid: NR | ✔ | ✔ | Reported but CD | NR |
|  | Magunia et al. (2021) | NR | 50 | Machine learning model | Survival outcome: 15 predictors; ECMO: 10 and renal replacement therapy: 10 | ICU: 64; ECMO: 73 and RRT: 70 | ICU: 0.81; ECMO: 0.69; RRT: 0.69 | NR | NR | NR | NR | NR | NR | ✔ | - | - | NR |
|  | Giamarellos-Bourboulis et al. (2022) | 14 day | 4 | SCOPE score | CRP, DD, ferritin, and IL-6 | NR | Discovery: 0.81 (0.75-0.86); validation 1: 0.79 (0.73-0.85) and validation II: 0.69 (0.55-0.72) | Discovery: 89.4; validation 1: 86.3 and; validation II: 98.6 | Discovery: 57.3; validation 1: 49.7 and validation II: 13.7 | Discovery: 32.3; validation 1: 41.4 and validation II: 43.9 | Discovery: 96.7; validation 1: 89.8 and; validation II: 93.3 | NR | NR | ✔ | ✔ | CD | Upon request |
|  | González-Flores et al. (2022) | NR | NA | Eight different scores | Obesity and T2DM score | NR | Death (Obesity and T2DM score (ODS): 0.86 (0.79-0.92), MV (ODS: 0.96 (0.93-0.99) Composite outcome; ODS: 0.89 (0.84-0.94) | Death: ODS (≥7 points): 78.8 (64.9 to 88.4); MV; ODS (≥5 points): 92.5 (83.8 to 96.9); Composite outcome; ODS (≥5 points): 77.0 (67.1 to 84.7) | Death: ODS (≥7 points): 82.8 (73.9 to 89.2); MV: ODS (≥5 points): 96.1 (88.2 to 98.9); Composite outcome; ODS (≥5 points): 95.0 (85.4 to 98.7) | Death: ODS (≥7 points): 0.86 (0.79-0.92); MV; ODS (≥5 points): 96.1 (88.2 to 98.9); Composite outcome; ODS (≥5 points): 96.1 (88.2 to 98.9) | Death: ODS (≥7 points): 69.4 (55.9 to 80.4); MV; ODS (≥5 points): 92.5 (83.8 to 96.9); Composite outcome; ODS (≥5 points): 72.5 (61.2 to 81.6) | Death: ODS (≥7 points)-LR+: 4.6 (2.9 to 7.1) and LR-: 0.2 (0.1 to 0.4); MV; ODS (≥5 points)-LR+: 23.7 (7.8 to 72.1) and LR-: 0.08 (0.04 to 0.17); Composite outcome; ODS (≥5 points)-LR+: 15.6 (5.1 to 47.5) and LR-: 0.2 (0.1 to 0.3) | NR | NA | NA | ✔ | Upon request |
|  | Alvarez-Uria et al. (2022) | NR | 20 | RCOS prognostic index | Age, systolic BP, heart rate, respiratory rate, AST, LDH, urea, CRP, sodium, LYM count, NEU count, and NLR | NR | Dev. cohort: 0.91 (0.89-0.92), Validation cohort: 0.91 (0.89-0.92) | CD | NR | NR | NR | NR | NR | ✔ | ✔ | - | Specific requests for data sharing will be; considered subject to ethical approval and data transfer; agreements. |
|  | He et al. (2022) | 28 days | 386 | Machine learning algorithms | Age, BUN, SpO_2_, systolic and diastolic BP, respiration rate, pulse, temperature, ALB, and major cognitive disorder | NR | All-cause mortality: 87.6 (87.2-87.9); ICU admission: 73.6 (73.2-74); MV: 78.1 (77.7-78.5) | NR | NR | NR | NR | NR | NR | ✔ | ✔ | ✔ | NR |
|  | Kamran et al. (2022) | first 5 days of hospital admission | 2686 | Machine learning model | Age, respiratory rate, oxygen saturation, oxygen flow rate, pulse oximetry, position of patient during BP measurement, venous blood gas pH, and partial pressure of CO_2_ in arterial blood | NR | 0.81 | NR | NR | NR | NR | NR | NR | ✔ | ✔ | ✔ | NR |
|  | Kim et al. (2022) | 30 days | 38 | Machine learning model | Activities of daily living, age, dyspnea, body temperature, sex, and symptoms of dyspnea | NR | ICU: 0.77 (0.61-0.93); oxygen supplementation: 0.73 (0.56-0.89) | NR | NR | NR | NR | NR | NR | ✔ | ✔ | ✔ | NR |
|  | Knight et al. (2022) | 4 weeks | NR | 4C mortality | Age, sex, number of comorbidities, respiratory rate, peripheral oxygen on room air, GCS, Urea, CRP, | ROX: 69.41 | NR | NR | NR | NR | NR | NR | 4C mortality: 0.78 (0.77-0.78); 4C deterioration 0.76 (0.76-0.77) | NR | ✔ | ✔ | Upon request |
|  | Kucuk et al. (2022) | NR | NR | Respiratory rate, oxygenation index, CT score | ROX, chest CT severity score, APACHE II score, SOFA score, CDCI and sex | ROX: 69.4; CT: 56.5 | Respiratory rate oxygenation index: 0.69 (0.58-0.81) CT score: 0.63 (0.51-0.75) | ROX: 78.3, CT: 65.3 | ROX: 59. CT:44.4 | ROX: 69.2; CT: 56.5 | ROX: 69.7; CT:48.5 | ROX index<3.8: OD: 4.8 (1.7–13); CT>15 OD: 2.8 (1–7.9) | NR | ✔ | ✔ | - | Upon request |
|  | Mu et al. (2022) | 5 days following hospitalization | NR | Machine learning model: augmenting Epic Deterioration Index (EDI) and MCURES with chest radiographs | NR | NR | Image-augmented EDI:0.66 (0.60-0.71); image-augmented MCURES: 0.81 (0.76-0.85) | NR | NR | NR | NR | NR | NR | ✔ | ✔ | - | NR |
|  | Nadasdi et al. (2022) | NR | 1 | Single biomarker | Serum circulating DPP4 activity | NR | 0.85 (0.79-0.90); 0.73 (0.62-0.84) | NR | NR | NR | NR | NR | NR | Investigation of the serum circulating DPP4 activity | - | - | Upon request |
|  | Cervantes-Alvarez et al. (2022) | On admission | NR | Laboratory values and galectin-3 levels | Galectin‑3 in combination with CRP, ALB and pulmonary CT | NR | GALECTIN-3: 0.75 (0.67–0.84) | 74.1 | 73.5 | NR | NR | NR | NR | ✔ | - | - | Upon request |
|  | Cruciata et al. (2022) | On admission | NR | Age, SaO_2_, NLR, CRP and LDH | Age, SaO_2_, NLR, CRP, LDH | NR | 0.88 (0.85–0.91) | A cutoff value; of 3: 93.5 (for death); 81 for worsening | 68.5 for death; 84.5 for worsening | 47.4 for death; 79.6 for worsening | 97.2 for death; 87.9 for worsening | NR | NR | ✔ | ✔ | - | Upon request |
|  | Hao et al. (2022) | 36 hrs. | NR | Parsimonious models | score intercept, immature granulocytes, sex, venous pH, absolute LYM, RDW, GLU, total elective surgery, CRP, age | NR | 0.94 | NR | NR | NR | NR | NR | NR | ✔ | ✔ | ✔ | Not available |
| *Values are percentages or mean age ± SD as appropriate, accompanied by their respective 95% confidence intervals if applicable or available.  **Abbreviations**: **SD**: Standard Deviation; **NR**: value not reported or found; **CD**: value could not be determined; **PCR**: polymerase chain reaction; **COVID-19**: Coronavirus Disease 2019; **HFNT**: High-Flow Nasal Therapy; **ECMO**: Extra Corporeal Membrane Oxygenation; **ICU**: Intensive Care Unit; **IMV**: invasive mechanical ventilation; **MV**: mechanical ventilation; **Dev.**: Dev.; **RT-PCR**: Reverse Transcriptase-Polymerase chain reaction; **AUC**: Area Under the Curve; **ROC**: Receiver Operating Characteristic; **PPV**: Positive Predictive Value; **NPV**: Negative Predictive Value; **LR**: Likelihood Ratio (denoted with a (+) if positive LR and with a (-) if negative LR; **C-index**: Concordance Statistics; **EDRF**: Early deterioration of respiratory function; **IRS**: intensive respiratory support; **CI**: Confidence Interval; **MEWS**; Modified Early Warning Score; **ANDC**: age; NLR; DD; and CRP; PSI; pneumonia severity index; **SpO_2_**: saturation of peripheral oxygen; **HCT**: Hematocrit; **CRP**: C-reactive protein; **AST**: aspartate aminotransferase; **MATRIA-RS**: Modified Anticoagulation and Risk Factors in Atrial Fibrillation Risk Score; **LDH**; lactate dehydrogenase; **PCT**: Procalcitonin; **4C Mortality Score**: 4C stands for Coronavirus Clinical Characterization Consortium); **PLANS**: Platelet count, lymphocyte count, age, NEU count, and sex; **VNPR**: Velocity of NPR (VNPR) and Velocity of NLR (VNLR); **NPR**: neutrophil-to-platelet-ratio; **NLR**: neutrophil-to-lymphocyte ratio; **DD**: DD; **ALB**: Albumin; **LymR**: Lymphocyte ratio; **NeuR**: NEU ratio; **BUN**: blood urea nitrogen; **PRE**: a combined predictive factor with lymphocyte ratio, BUN, DD; **SOFA**: Sequential Organ Failure Assessment; **qSOFA**; Quick Sequential Organ Failure Assessment; **SCARP**: Severe COVID-19 Adaptive Risk Predictor; **COPD**: chronic obstructive pulmonary disease; **APACHE II**: Acute Physiology and Chronic Health Evaluation II; **LUS**: Lung Ultra Sound; **ICC**: intraclass correlation coefficient; **NEWS2**: National Early Warning Score 2; **BMI**: Body Mass Index; **ESR**: erythrocyte sedimentation rate; **CDCI**: Charlson/Deyo comorbidity index; **ALT**: alanine aminotransferase; **aPTT**; activated partial thromboplastin time; **CK**: creatine kinase ; **WBC**: white blood cell; **cTnI**: troponin I; **IL-6**: interleukin-6; **T2DM**: type 2 diabetes mellitus; **BP**: Blood Pressure; **GLU**: blood glucose; **LYM**: lymphocytes; RDW: Red cell distribution width; **DD**: d-dimer; **DPP4**: Dipeptidyl peptidase-4; **GCS**: Glasgow Coma Scale; MAP: mean arterial pressure; | | | | | | | | | | | | | | | | | |
