# Supplementary material for: Prognostic models in COVID-19 infection that predict severity: a systematic review
Source: Eur J Epidemiol. 2023 Feb 25;38(4):355–72. doi: 10.1007/s10654-023-00973-x (PMC9958330; doi:10.1007/s10654-023-00973-x)
Supplement: Supplementary file 5 — Supplementary file5 (DOCX 246 KB) [file 10654_2023_973_MOESM5_ESM.docx]

| **Table 5: Summary of Risk of bias (ROB) assessment of the included studies according to Prediction model Risk Of Bias Assessment Tool (PROBAST)** | | | | | | | | | | | | | | | | | | | | | | | | | | | | | | |
| --- | --- | --- | --- | --- | --- | --- | --- | --- | --- | --- | --- | --- | --- | --- | --- | --- | --- | --- | --- | --- | --- | --- | --- | --- | --- | --- | --- | --- | --- | --- |
|  | **Author (year)** | **Participants** | | **ROB** | **Applicability** | **Predictors** | | | **ROB** | **Applicability** | **Outcome** | | | | | | **ROB** | **Applicability** | **Analysis** | | | | | | | | | **ROB** | **Overall Applicability** | **Overall, ROB** |
|  | (2) Zhou Y et al. (2020) | x | ✓ | x | ✓ | ✓ | x | ✓ | x | ✓ | ✓ | ✓ | ✓ | ✓ | ✓ | ✓ | ✓ | ✓ | ✓ | ✓ | ✓ | ✓ | x | ? | ✓ | ✓ | ✓ | x | ✓ | x |
|  | Acar et al. (2021) | x | ✓ | x | ✓ | ✓ | x | ✓ | x | ✓ | ✓ | ✓ | ✓ | ✓ | x | ✓ | x | ✓ | ✓ | ✓ | x | ? | ? | ? | ✓ | ? | ✓ | x | ✓ | x |
|  | Aciksari et al. (2021) | x | ? | x | ✓ | ✓ | x | ✓ | x | ✓ | ✓ | ✓ | ✓ | ✓ | x | ✓ | x | ✓ | ✓ | ✓ | ✓ | x | ✓ | ? | x | ✓ | ✓ | x | ✓ | x |
|  | Ageno (et al. 2021) | x | ? | x | ✓ | ✓ | x | ✓ | x | ✓ | ✓ | ✓ | ✓ | ✓ | x | ✓ | x | ✓ | ✓ | x | ✓ | ✓ | ? | ? | x | ✓ | ✓ | x | ✓ | x |
|  | Ak et al. (2021) | x | ✓ | x | ✓ | ✓ | x | ✓ | x | ✓ | ✓ | ✓ | x | ✓ | x | ? | x | ✓ | ✓ | ? | ? | x | ? | ? | x | ? | ✓ | x | ✓ | x |
|  | Al Abbasi et al. (2020) | x | ✓ | x | ✓ | ✓ | x | ✓ | x | ✓ | ✓ | ✓ | ✓ | ✓ | x | ✓ | x | ✓ | ✓ | ✓ | ✓ | ? | ? | ? | x | x | ? | x | ✓ | x |
|  | Alfaro-Martínez et al. (2021) | x | ✓ | x | ✓ | ✓ | x | ✓ | x | ✓ | ✓ | ✓ | ✓ | ✓ | x | ? | x | ✓ | ✓ | ✓ | ✓ | ? | x | ? | ? | ? | ✓ | x | ✓ | x |
|  | Allahverdiyev et al. (2020) | x | ✓ | x | ✓ | ✓ | x | ✓ | x | ✓ | ✓ | ✓ | ✓ | ✓ | ✓ | ? | ? | ✓ | ✓ | ✓ | ✓ | ✓ | ✓ | x | x | x | x | x | ✓ | x |
|  | Altschul et al. (2020) | x | ✓ | x | ✓ | ✓ | x | ✓ | x | ✓ | ✓ | ✓ | ✓ | ✓ | x | ✓ | x | ✓ | ✓ | x | x | x | x | ? | ? | ? | ✓ | x | ✓ | x |
|  | Amezcua‑Guerra et al. (2021) | ✓ | ✓ | ✓ | ✓ | ✓ | ✓ | ? | ? | ✓ | ✓ | ✓ | ✓ | ✓ | ✓ | ? | ? | ✓ | ✓ | ✓ | ✓ | ✓ | ✓ | ✓ | x | x | x | x | ✓ | x |
|  | Andreano et al. (2021) | x | ✓ | x | ✓ | ✓ | x | ✓ | x | ✓ | ✓ | ✓ | ✓ | ✓ | ✓ | ✓ | ✓ | ✓ | ✓ | ✓ | ✓ | ✓ | ✓ | x | ✓ | ✓ | ✓ | x | ✓ | x |
|  | Arnold et al. (2021) | ✓ | ✓ | ✓ | ✓ | ✓ | ✓ | ✓ | ✓ | ✓ | ✓ | ✓ | ? | ✓ | x | ✓ | x | x | x | x | ✓ | ? | ? | ? | x | ✓ | ✓ | x | x | x |
|  | Arvind et al. (2020) | x | ✓ | x | ✓ | ✓ | x | ✓ | x | ✓ | ✓ | ✓ | ✓ | ✓ | x | ✓ | x | ✓ | ✓ | ✓ | ✓ | ✓ | ? | ✓ | ✓ | x | ? | x | ✓ | x |
|  | Asghar et al. (2020) | x | ? | x | ✓ | ✓ | x | ✓ | x | ✓ | ✓ | ✓ | ✓ | ✓ | x | ✓ | x | ✓ | ✓ | ✓ | ✓ | ? | ? | ? | ? | ? | ? | x | ✓ | x |
|  | Bastug et al. (2020) | x | ✓ | x | ✓ | ✓ | x | ✓ | x | ✓ | ✓ | ✓ | ✓ | ✓ | x | ✓ | x | ✓ | ✓ | ✓ | ✓ | ? | x | ? | x | x | ✓ | x | ✓ | x |
|  | Bello-Chavolla et al. (2021) | ✓ | ✓ | ✓ | ✓ | ✓ | ✓ | ✓ | ✓ | ✓ | ✓ | ✓ | ✓ | ✓ | ✓ | ✓ | ✓ | ✓ | ✓ | ✓ | ✓ | ✓ | ✓ | ✓ | ✓ | ✓ | x | x | ✓ | x |
|  | Bellos et al. (2021) | ✓ | ✓ | ✓ | ✓ | ✓ | x | ✓ | x | ✓ | ✓ | ✓ | ? | ✓ | x | ✓ | x | ✓ | x | x | ✓ | ✓ | ✓ | ? | ? | ✓ | ✓ | x | ✓ | x |
|  | Bennouar S et al. (2020) | ✓ | ✓ | ✓ | ✓ | ✓ | ✓ | ✓ | x | ✓ | ✓ | ✓ | ✓ | ✓ | x | ✓ | x | ✓ | ✓ | ✓ | ? | ? | x | ? | ? | ? | ✓ | x | ✓ | x |
|  | Bennouar S et al. (2021) | x | ✓ | x | ✓ | ✓ | x | ✓ | x | ✓ | ✓ | ✓ | ✓ | ✓ | x | ✓ | x | ✓ | ✓ | ? | ? | ? | ? | ? | ? | ? | ✓ | ? | ✓ | x |
|  | Bertsimas et al. (2020) | x | ✓ | x | ✓ | ✓ | x | ✓ | x | ✓ | ✓ | ✓ | ? | ✓ | ✓ | ? | ? | ✓ | ✓ | ✓ | x | ✓ | ? | ? | ? | x | ✓ | x | ✓ | x |
|  | Besutti et al. (2021) | x | ✓ | x | ✓ | ✓ | x | ✓ | x | ✓ | ✓ | ✓ | ✓ | ✓ | ? | ✓ | ? | ✓ | ✓ | ✓ | ? | ✓ | x | ? | ? | ? | ✓ | x | ✓ | x |
|  | Boero et al. (2021) | x | ✓ | x | ✓ | ✓ | x | ✓ | x | ✓ | ✓ | ✓ | ✓ | ✓ | x | ✓ | x | x | ✓ | ✓ | x | x | x | ? | ✓ | ✓ | ✓ | x | x | x |
|  | Cai et al. (2021) | x | ✓ | x | ✓ | ✓ | x | ✓ | x | ✓ | ✓ | ✓ | ✓ | ✓ | ? | ✓ | ? | ✓ | ✓ | ✓ | x | ? | ✓ | ? | ✓ | ✓ | ✓ | x | ✓ | x |
|  | Chen Yuanyuan et al. (2021) | x | ✓ | x | ✓ | ✓ | x | ✓ | x | ✓ | ✓ | ✓ | ✓ | ✓ | ✓ | ? | ? | ✓ | ✓ | ✓ | ✓ | ✓ | ✓ | ? | x | ✓ | ✓ | x | ✓ | x |
|  | Cheng Fu-Yuan et al. (2020) | x | ? | x | ✓ | ? | ✓ | ✓ | ? | ✓ | ✓ | ✓ | ✓ | ? | x | ✓ | x | ✓ | ✓ | ? | x | ✓ | x | ✓ | ? | ✓ | x | x | ✓ | x |
|  | Cheng P et al. (2021) | x | ✓ | x | ✓ | ✓ | ? | ✓ | ? | ✓ | ✓ | ✓ | ✓ | ✓ | x | ✓ | x | ✓ | x | ✓ | x | ? | ? | ? | x | x | ✓ | x | ✓ | x |
|  | Covino et al. (2020) | x | ✓ | x | ✓ | ✓ | ✓ | ✓ | ✓ | ✓ | ✓ | ✓ | ✓ | ✓ | x | ✓ | x | ✓ | ? | ✓ | ✓ | ✓ | ? | ? | ? | x | ✓ | x | ✓ | x |
|  | De Alencar et al. (2021) | ✓ | ✓ | ✓ | ✓ | ✓ | ✓ | ✓ | ✓ | ✓ | ✓ | ✓ | ✓ | ✓ | ✓ | ✓ | ✓ | ✓ | ✓ | ✓ | x | ✓ | x | ? | x | ? | ✓ | x | ✓ | x |
|  | De Socio et al. (2021) | x | ✓ | x | ✓ | ✓ | x | ✓ | x | ✓ | ✓ | ✓ | x | ✓ | x | ? | x | ✓ | x | ? | x | ? | ? | ? | ? | ? | ✓ | x | ✓ | x |
|  | Ebell et al. (2021) | x | ✓ | x | ✓ | ✓ | x | ✓ | x | ✓ | ✓ | ✓ | ✓ | ✓ | ? | ? | ? | ✓ | ✓ | ✓ | x | ✓ | x | ? | ✓ | ✓ | ✓ | x | ✓ | x |
|  | Fan et al. (2021) | x | ✓ | x | ✓ | ? | x | ✓ | x | ✓ | ✓ | ✓ | ✓ | ✓ | ? | ? | ? | ✓ | x | ✓ | ? | ? | ✓ | ? | x | ✓ | ✓ | x | ✓ | x |
|  | Fernandes et al. (2021) | ✓ | ✓ | ✓ | ✓ | ✓ | x | ✓ | x | ✓ | ✓ | ✓ | ✓ | ? | ? | ? | ? | ✓ | ✓ | ✓ | x | ✓ | x | ? | ? | ✓ | ✓ | x | ✓ | x |
|  | Gao et al. (2021) | x | ✓ | x | ✓ | ✓ | x | ✓ | x | ✓ | ✓ | ✓ | ✓ | ✓ | ? | ✓ | ? | ✓ | ✓ | ✓ | x | ✓ | ✓ | ? | x | ✓ | ✓ | x | ✓ | x |
|  | García Clemente et al. (2020) | ✓ | ✓ | ✓ | ✓ | ✓ | x | ✓ | x | ✓ | ✓ | ✓ | ? | ✓ | ✓ | ✓ | ? | ✓ | ? | ✓ | ✓ | ? | x | ? | ? | x | ✓ | x | ✓ | x |
|  | Garcia-Gordillo et al. (2021) | x | ✓ | x | ✓ | ✓ | x | x | x | ✓ | ✓ | ✓ | ✓ | ✓ | ✓ | ✓ | ✓ | ✓ | ✓ | ✓ | x | ? | x | ? | x | ✓ | ✓ | x | ✓ | x |
|  | Gresser et al. (2021) | x | ? | x | ✓ | ✓ | x | ✓ | x | ✓ | ✓ | ✓ | x | ✓ | x | ✓ | x | ✓ | x | ✓ | ✓ | x | ? | ? | ✓ | x | ✓ | x | ✓ | x |
|  | Gude et al. (2020) | x | ✓ | x | ✓ | ✓ | x | ✓ | x | ✓ | ✓ | ✓ | ✓ | ✓ | x | ✓ | x | ✓ | ✓ | ✓ | ✓ | ? | x | ✓ | x | x | ✓ | x | ✓ | x |
|  | Gue, (2020) | x | ✓ | x | ✓ | ✓ | x | ✓ | x | ✓ | ✓ | ✓ | ✓ | ✓ | x | ✓ | x | ✓ | ✓ | ? | ✓ | ? | x | ? | ? | ? | ✓ | x | ✓ | x |
|  | Hachim et al. (2020) | x | ✓ | x | ✓ | ✓ | x | ? | x | ✓ | ✓ | ? | ? | ✓ | ? | ? | ? | ✓ | ✓ | ✓ | x | ? | ? | ? | ✓ | ✓ | ? | x | ✓ | x |
|  | Hajifathalian et al. (2020) | x | ✓ | x | ✓ | ✓ | x | ✓ | x | ✓ | ✓ | ✓ | ✓ | ✓ | x | ✓ | x | ✓ | ✓ | ✓ | ✓ | ✓ | x | ✓ | ✓ | ✓ | ✓ | x | ✓ | x |
|  | Hu, C. et al. (2021) | x | ✓ | x | ✓ | ✓ | ✓ | ✓ | ✓ | ✓ | ? | ? | ? | ? | x | ✓ | x | ? | ✓ | ✓ | ✓ | ✓ | ? | ? | x | ✓ | ✓ | x | ? | x |
|  | Hu, H. et al. (2020) | x | ✓ | x | ✓ | ✓ | x | ✓ | x | ✓ | ✓ | ? | ? | ? | ✓ | ✓ | ? | ✓ | x | ✓ | ✓ | ? | x | ? | ? | ✓ | ✓ | x | ✓ | x |
|  | Hu, Hai. et al. (2020) | x | ✓ | x | ✓ | ✓ | x | ✓ | x | ✓ | ✓ | ? | ? | ? | ? | ✓ | ? | ✓ | ✓ | ✓ | x | x | ? | ? | ✓ | ? | ✓ | x | ✓ | x |
|  | Jiang et al. (2021) | x | ✓ | x | ✓ | x | x | ✓ | x | ✓ | ✓ | ? | ? | ? | ? | ✓ | ? | ✓ | ✓ | ✓ | ? | x | ✓ | ? | ✓ | ? | ✓ | x | ✓ | x |
|  | Jimenez‐Solem et al. (2021) | ✓ | ✓ | ✓ | ✓ | ✓ | x | ? | x | ✓ | ✓ | ✓ | ✓ | ✓ | ? | ? | ? | ✓ | ✓ | ✓ | x | ✓ | ? | ? | ✓ | x | ✓ | x | ✓ | x |
|  | King et al. (2020) | x | ✓ | x | ✓ | ? | x | ✓ | x | ✓ | ✓ | ✓ | ✓ | ✓ | x | ✓ | x | ✓ | ✓ | ? | ? | ? | x | ? | ✓ | ✓ | ✓ | x | ✓ | x |
|  | Knight et al. (2020) | ✓ | ✓ | ✓ | ✓ | ✓ | ✓ | ✓ | ✓ | ✓ | ✓ | ✓ | ✓ | ✓ | ✓ | ✓ | ✓ | ✓ | ✓ | ✓ | ✓ | ✓ | ✓ | ✓ | ✓ | ✓ | ✓ | ✓ | ✓ | ✓ |
|  | Kulkarni et al. (2021) | x | x | x | ✓ | ✓ | x | ✓ | x | ✓ | ✓ | ✓ | ✓ | ✓ | x | ✓ | x | ✓ | ✓ | ✓ | ✓ | ? | ✓ | ✓ | x | ✓ | ✓ | x | ✓ | x |
|  | Kurt et al. (2021) | x | ✓ | x | ✓ | ✓ | x | ✓ | x | ✓ | ✓ | ✓ | ✓ | ✓ | x | ✓ | x | ✓ | ✓ | ✓ | ✓ | ? | ? | ? | ✓ | x | ? | x | ✓ | x |
|  | Laguna-Goya et al. (2021) | ✓ | ✓ | x | ✓ | ✓ | ✓ | ✓ | ✓ | ✓ | ✓ | ✓ | ✓ | ✓ | ✓ | ✓ | ✓ | ✓ | ✓ | ✓ | ✓ | ? | x | x | ✓ | ✓ | ✓ | x | ✓ | x |
|  | Li J et al. (2020) | x | ✓ | x | ✓ | ✓ | x | ✓ | x | ✓ | ✓ | ✓ | ✓ | ✓ | x | ✓ | x | ✓ | ✓ | ✓ | ✓ | x | x | ✓ | ✓ | ✓ | ✓ | x | ✓ | x |
|  | Lazar Neto et al. (2021) | x | ✓ | x | ✓ | ✓ | x | ✓ | x | ✓ | ✓ | ✓ | ✓ | ✓ | x | ✓ | x | ✓ | ✓ | ✓ | ✓ | ✓ | ? | ✓ | ✓ | ✓ | ? | x | ✓ | x |
|  | Levine et al (2021) | x | ✓ | x | ✓ | ✓ | x | ✓ | x | ✓ | ✓ | ✓ | ✓ | ✓ | x | ✓ | x | ✓ | ✓ | ✓ | ✓ | x | ? | ✓ | ✓ | ✓ | ✓ | x | ✓ | x |
|  | Li L et al. (2021) | x | ✓ | x | ✓ | ? | ? | ? | ? | ✓ | ? | ? | ? | ? | x | ? | x | ✓ | ✓ | x | ✓ | ✓ | x | x | ✓ | ✓ | ✓ | x | ✓ | x |
|  | Liu Li et al. (2021) | x | x | x | ✓ | ✓ | x | ✓ | x | ✓ | ✓ | ✓ | ✓ | ✓ | x | ✓ | x | ✓ | ✓ | x | ✓ | x | x | ? | ✓ | ✓ | ✓ | x | ✓ | x |
|  | Liu Q et al. (2020) | x | ✓ | x | ✓ | ✓ | x | ✓ | x | ✓ | ✓ | ✓ | ✓ | ✓ | x | ✓ | x | ✓ | ✓ | ✓ | ✓ | x | x | ✓ | ✓ | ✓ | ✓ | x | ✓ | x |
|  | Li S et al. (2021) | x | ✓ | x | ✓ | ✓ | x | ✓ | x | ✓ | ✓ | ✓ | ✓ | ✓ | x | ✓ | x | ✓ | x | ✓ | ✓ | x | ? | x | x | x | ✓ | x | ✓ | x |
|  | Li X et al. (2020) | x | ✓ | x | ✓ | ✓ | x | ✓ | x | ✓ | ✓ | ✓ | ✓ | ✓ | x | ✓ | x | ✓ | ✓ | ✓ | x | x | ? | x | x | ✓ | ✓ | x | ✓ | x |
|  | Liang et al. (2020) | x | x | x | ✓ | ✓ | x | ✓ | x | ✓ | ✓ | ✓ | ✓ | ✓ | x | ✓ | x | ✓ | ✓ | ✓ | ✓ | ✓ | ? | x | x | ✓ | ✓ | x | ✓ | x |
|  | Liu H et al. (2021) | x | ✓ | x | ✓ | ✓ | x | ✓ | x | ✓ | ✓ | ✓ | ✓ | ✓ | ✓ | ✓ | ✓ | ✓ | ✓ | ✓ | x | ✓ | x | ✓ | ✓ | ✓ | ✓ | x | ✓ | x |
|  | Liu Q et al. (2021) | x | ✓ | x | ✓ | ✓ | x | ✓ | x | ✓ | ✓ | ✓ | ✓ | ✓ | x | ✓ | x | ✓ | ✓ | ✓ | ✓ | ✓ | ? | ? | x | ✓ | ? | x | ✓ | x |
|  | Liu S et al. (2020) | x | ✓ | x | ✓ | ✓ | x | ✓ | x | ✓ | ✓ | ✓ | ✓ | ✓ | x | ✓ | x | ✓ | x | ✓ | ✓ | x | ✓ | ? | ✓ | x | ? | x | ✓ | x |
|  | Liu, J. et al. (2020) | x | ✓ | x | ✓ | ✓ | x | ✓ | x | ✓ | ✓ | ✓ | ✓ | ✓ | x | ? | x | ✓ | ✓ | ✓ | ? | ? | x | ? | ✓ | ✓ | ✓ | x | ✓ | x |
|  | López-Escobar et al. (2021) | x | ✓ | x | ✓ | ✓ | x | ✓ | x | ✓ | ✓ | ✓ | ✓ | ✓ | x | ✓ | x | ✓ | ✓ | x | ✓ | ✓ | ✓ | ? | ✓ | ✓ | ✓ | x | ✓ | x |
|  | Ma X et al. (2020) | x | ✓ | x | ✓ | ✓ | x | ✓ | x | ✓ | ✓ | ✓ | ✓ | ✓ | x | ✓ | x | ✓ | x | x | ✓ | x | ? | x | ✓ | ✓ | ? | x | ✓ | x |
|  | Ma et al. (2020) | x | ✓ | x | ✓ | ✓ | x | ✓ | x | ✓ | ✓ | ✓ | ✓ | ✓ | x | ✓ | x | ✓ | ✓ | ✓ | ✓ | x | x | x | ✓ | x | ✓ | x | ✓ | x |
|  | Magro et al (2021) | x | ✓ | x | ✓ | ✓ | x | ✓ | x | ✓ | ✓ | ✓ | ✓ | ✓ | x | ✓ | x | ✓ | ✓ | ✓ | ✓ | x | x | x | ✓ | ✓ | ✓ | x | ✓ | x |
|  | Marcos et al. (2021) | x | ? | x | ✓ | ✓ | x | ✓ | x | ✓ | ✓ | ✓ | ✓ | ✓ | x | ✓ | x | ✓ | ✓ | ✓ | ✓ | ✓ | ? | x | x | ✓ | ? | x | ✓ | x |
|  | Myrstad et al. (2020) | ✓ | ✓ | ✓ | ✓ | ✓ | x | ✓ | x | ✓ | ✓ | ✓ | x | ✓ | x | ✓ | x | ✓ | x | ✓ | ✓ | ? | ? | ? | ✓ | ? | ✓ | x | ✓ | x |
|  | Pan et al. (2020) | x | ✓ | x | ✓ | ✓ | x | ✓ | x | ✓ | ✓ | ? | ? | ✓ | ✓ | ✓ | ? | ✓ | ✓ | ✓ | x | ? | x | ? | ✓ | ✓ | ✓ | x | ✓ | x |
|  | Paranjape et al. (2021) | x | ✓ | x | ✓ | ✓ | x | ✓ | x | ✓ | ✓ | x | ✓ | ✓ | x | ✓ | x | ✓ | ✓ | ✓ | ✓ | ✓ | ✓ | ? | ? | ✓ | ✓ | ? | ✓ | x |
|  | Pigoga et al. (2021) | x | ✓ | x | ✓ | ✓ | x | ✓ | x | ✓ | ✓ | ✓ | ✓ | ✓ | x | x | x | ✓ | ✓ | ✓ | ✓ | ✓ | ? | ? | ? | ✓ | ✓ | ? | ✓ | x |
|  | Pokeerbux et al. (2021) | x | ✓ | x | ✓ | ✓ | x | ✓ | x | ✓ | ✓ | ✓ | ✓ | ✓ | x | ✓ | x | ✓ | ✓ | ✓ | ✓ | ✓ | ✓ | ? | x | x | ✓ | x | ✓ | x |
|  | Ponsford et al. (2021) | x | ✓ | x | ✓ | ✓ | x | ✓ | x | ✓ | ✓ | ✓ | ✓ | ✓ | x | ✓ | x | ✓ | ✓ | ✓ | x | ✓ | x | ? | ✓ | ✓ | ✓ | x | ✓ | x |
|  | Prower et al. (2021) | x | ✓ | x | ✓ | ✓ | x | ✓ | x | ✓ | ✓ | ✓ | x | ✓ | x | ✓ | x | ✓ | ✓ | ✓ | ? | ✓ | x | ? | x | ? | ✓ | x | ✓ | x |
|  | Purkayastha et al. (2021) | x | ✓ | x | ✓ | ✓ | x | ✓ | x | ✓ | ✓ | ✓ | ✓ | ✓ | x | ✓ | x | ✓ | ✓ | ? | x | ✓ | ? | ? | ? | x | ✓ | x | ✓ | x |
|  | Quanjel et al. (2020) | x | ✓ | x | ✓ | ✓ | x | ✓ | x | ✓ | ✓ | ✓ | ✓ | ✓ | x | ✓ | x | ✓ | ✓ | ? | ? | ? | ? | ? | ? | ? | ✓ | ? | ✓ | x |
|  | Rasyid et al. (2021) | x | ✓ | x | ✓ | ✓ | x | ✓ | x | ✓ | ✓ | ✓ | ✓ | ✓ | x | ✓ | x | ✓ | ✓ | ✓ | ? | ? | ? | ? | ? | ? | ✓ | ? | ✓ | x |
|  | Rodriguez-Nava et al. (2021) | x | ✓ | x | ✓ | ✓ | x | ✓ | x | ✓ | ✓ | ✓ | x | ✓ | x | ✓ | x | ✓ | ✓ | ✓ | ✓ | ? | ? | ? | ✓ | ? | ✓ | ? | ✓ | x |
|  | Satici et al. (2020) | x | ✓ | x | ✓ | ✓ | x | ✓ | x | ✓ | ✓ | ✓ | x | ✓ | x | ✓ | x | ✓ | ✓ | ✓ | ✓ | ✓ | ✓ | ? | ✓ | ? | ✓ | ? | ✓ | x |
|  | Schalekamp et al. (2021) | x | ✓ | x | ✓ | ✓ | x | x | x | ✓ | ✓ | ✓ | x | ✓ | x | ✓ | x | ✓ | ✓ | ✓ | x | ✓ | x | ? | ✓ | ✓ | ✓ | x | ✓ | x |
|  | Schöning et al. (2021) | x | ✓ | x | ✓ | ✓ | x | ✓ | x | ✓ | ✓ | ✓ | ✓ | ✓ | ✓ | ? | ? | ✓ | x | x | ✓ | ✓ | x | x | x | ✓ | x | x | ✓ | x |
|  | Selcuk et al. (2021) | x | ✓ | x | ✓ | ✓ | x | ✓ | x | ✓ | ✓ | ✓ | ✓ | ✓ | x | ✓ | x | ✓ | ✓ | ✓ | ✓ | x | x | ? | ? | ? | ✓ | x | ✓ | x |
|  | Shang et al. (2020) | x | ✓ | x | ✓ | ✓ | x | ✓ | x | ✓ | ✓ | ✓ | ✓ | ✓ | x | ✓ | x | ✓ | ✓ | ? | x | ? | ? | ? | ✓ | ? | ✓ | x | ✓ | x |
|  | Shi Y et al. (2021) | x | ✓ | x | ✓ | ✓ | x | ✓ | x | ✓ | ✓ | ✓ | ✓ | ✓ | ? | ✓ | ? | ✓ | x | ✓ | x | ✓ | ? | ? | ✓ | ? | ✓ | x | ✓ | x |
|  | Shi, S. et al. (2021) | x | ✓ | x | ✓ | ✓ | x | ✓ | x | ✓ | ✓ | ✓ | ✓ | ✓ | ? | ? | ? | ✓ | x | ? | ✓ | ? | x | ? | ? | ? | ✓ | x | ✓ | x |
|  | Soto-Mota et al. (2020) | x | ✓ | x | ✓ | ✓ | x | ✓ | x | ✓ | ✓ | ✓ | ✓ | ✓ | ? | ? | ? | ✓ | ✓ | x | x | ? | ? | ? | x | ? | ✓ | x | ✓ | x |
|  | Stachel et al. (2021) | x | ✓ | x | ✓ | ✓ | x | ✓ | x | ✓ | ✓ | ? | ? | ✓ | ✓ | ✓ | ? | ✓ | ✓ | x | ? | ✓ | ? | ? | ✓ | ✓ | ✓ | x | ✓ | x |
|  | Statsenko et al. (2021) | x | ✓ | x | ✓ | ✓ | x | ✓ | x | ✓ | ✓ | ? | ? | ✓ | x | ✓ | x | ✓ | ✓ | ✓ | ✓ | ✓ | ✓ | ? | ? | ✓ | ✓ | ? | ✓ | x |
|  | Su Y et al. (2020) | x | ✓ | x | ✓ | ? | ? | ? | ? | ? | ✓ | ✓ | ? | ✓ | ✓ | ? | ? | x | ? | ✓ | ? | ? | ? | ? | ? | ? | ? | ? | x | ? |
|  | Tanboga et al. (2021) | x | ✓ | x | ✓ | ✓ | x | x | x | ✓ | ✓ | ✓ | ✓ | ✓ | ✓ | ✓ | ✓ | ✓ | ✓ | ✓ | ✓ | ✓ | ✓ | ? | ✓ | ✓ | ✓ | ? | ✓ | x |
|  | Tezza et al. (2021) | x | ✓ | x | ✓ | ✓ | x | ✓ | x | ✓ | ✓ | ✓ | ✓ | ✓ | ✓ | ? | ? | ✓ | ✓ | ✓ | ✓ | ? | ? | ? | ? | ? | ? | ? | ✓ | x |
|  | Tu et al. (2021) | x | ✓ | x | ✓ | ✓ | x | ✓ | x | ✓ | ✓ | ✓ | ✓ | ✓ | ✓ | ✓ | ✓ | ✓ | ✓ | ✓ | ✓ | ? | x | ? | x | ✓ | ✓ | x | ✓ | x |
|  | Ucan et al. (2021) | x | ✓ | x | ✓ | ? | x | ? | x | ✓ | ✓ | ? | ? | ? | ✓ | ✓ | ? | ✓ | ✓ | ✓ | x | ? | ? | ? | ? | ? | ? | x | ✓ | x |
|  | Van Dam et al. (2021) | x | ✓ | x | ✓ | ✓ | x | ✓ | x | ✓ | ✓ | ✓ | x | ✓ | x | ✓ | x | ✓ | ✓ | x | ✓ | ✓ | ? | ? | ✓ | ✓ | ✓ | x | ✓ | x |
|  | Wang X et al. (2020) | x | ✓ | x | ✓ | ✓ | x | ✓ | x | ✓ | ✓ | ✓ | ✓ | ✓ | x | ✓ | x | ✓ | ✓ | ? | ✓ | ? | ? | ? | x | x | ✓ | x | ✓ | x |
|  | Wang L et al. (2020) | x | ✓ | x | ✓ | ✓ | x | ✓ | x | ✓ | ✓ | ✓ | ✓ | ✓ | x | x | x | ✓ | ✓ | ✓ | x | ? | x | ? | ? | ? | ✓ | x | ✓ | x |
|  | Weng et al. (2020) | x | ✓ | x | ✓ | ✓ | x | ✓ | x | ✓ | ✓ | ✓ | ✓ | ✓ | x | ✓ | x | ✓ | ✓ | ✓ | ✓ | ✓ | ? | ? | ✓ | ✓ | ✓ | ? | ✓ | x |
|  | Wongvibulsin et al. (2021) | x | ✓ | x | ✓ | ✓ | x | ✓ | x | ✓ | ✓ | ✓ | x | ✓ | x | ✓ | x | ✓ | ✓ | ✓ | ? | ✓ | x | ? | ✓ | ✓ | ✓ | x | ✓ | x |
|  | Woo et al. (2021) | x | ✓ | x | ✓ | ✓ | ✓ | ✓ | ✓ | ✓ | ✓ | ✓ | ✓ | ✓ | ? | ✓ | ? | ✓ | ✓ | ✓ | ✓ | ✓ | ✓ | ? | ✓ | ✓ | ✓ | ? | ✓ | ? |
|  | Wu et al. (2020) | x | ✓ | x | ✓ | ✓ | ✓ | ✓ | ✓ | ✓ | ✓ | ✓ | ✓ | ✓ | x | x | x | ✓ | ✓ | ✓ | x | ? | ? | ? | ? | ✓ | ? | x | ✓ | x |
|  | Xiao et al. (2020) | x | ✓ | x | ✓ | ✓ | x | ✓ | x | ✓ | ✓ | ✓ | x | ✓ | x | ? | x | ✓ | ✓ | ? | ✓ | ? | ? | ? | ✓ | ✓ | ? | ? | ✓ | x |
|  | Xu F et al. (2021) | x | x | x | ✓ | ✓ | x | ✓ | x | ✓ | ✓ | ✓ | x | ✓ | x | ? | x | ✓ | ✓ | ✓ | ? | ? | x | ? | ? | ✓ | ✓ | x | ✓ | x |
|  | Xu J et al. (2021) | x | ✓ | x | ✓ | ✓ | x | ✓ | x | ✓ | ✓ | ✓ | ✓ | ✓ | ✓ | ✓ | ✓ | ✓ | ✓ | ✓ | ✓ | ? | ? | ? | ? | x | ✓ | x | ✓ | x |
|  | Yang Y et al. (2021) | x | x | x | ✓ | ✓ | x | ✓ | x | ✓ | ? | ? | ? | ✓ | x | ✓ | x | ✓ | ✓ | ✓ | ? | ? | ? | ? | ✓ | ✓ | ✓ | ? | ✓ | x |
|  | Yao et al. (2021) | x | ✓ | x | ✓ | ✓ | x | ✓ | x | ✓ | ✓ | ✓ | ✓ | x | x | ✓ | x | ✓ | ✓ | x | x | ? | x | ? | ✓ | ✓ | ✓ | x | ✓ | x |
|  | Youssef et al. (2021) | x | x | x | ✓ | ✓ | x | ✓ | x | ✓ | ✓ | ✓ | x | ✓ | x | ✓ | x | ✓ | x | ? | ? | ✓ | ? | ? | ✓ | ✓ | ? | x | ✓ | x |
|  | Yu L et al. (2021) | x | ✓ | x | ✓ | ✓ | x | ✓ | x | ✓ | ✓ | ✓ | ? | ✓ | x | ? | x | ✓ | ✓ | ✓ | ✓ | ✓ | ? | ? | ? | ✓ | ? | ? | ✓ | x |
|  | Yu Y et al. (2020) | x | ✓ | x | ✓ | ✓ | x | ✓ | x | ✓ | ✓ | ✓ | ✓ | ✓ | x | ✓ | x | ✓ | ✓ | ? | ✓ | ? | x | ? | x | x | ✓ | x | ✓ | x |
|  | Yuan Y et al. (2020) | x | ✓ | x | ✓ | ✓ | x | ✓ | x | ✓ | ✓ | ✓ | ✓ | ✓ | x | ✓ | x | ✓ | ✓ | ? | ? | ? | ✓ | ? | ? | x | ✓ | x | ✓ | x |
|  | Zayed et al. (2021) | x | ✓ | x | ✓ | ✓ | ? | ✓ | ? | ? | ✓ | ? | ? | ✓ | ✓ | ? | ? | ? | ✓ | ✓ | ✓ | ? | ? | ? | ? | x | ? | x | ? | x |
|  | Zeng et al. (2021) | x | ✓ | x | ✓ | ✓ | x | ✓ | x | ✓ | ✓ | ✓ | ✓ | ✓ | x | ✓ | x | ✓ | ✓ | x | ? | ? | x | ? | ✓ | ✓ | ✓ | x | ✓ | x |
|  | Zhang B et al. (2020) | x | ✓ | x | ✓ | ✓ | ✓ | ✓ | ✓ | ✓ | ✓ | ✓ | ✓ | ✓ | ✓ | ✓ | ✓ | ✓ | ✓ | ✓ | ✓ | ✓ | ✓ | ✓ | ✓ | ✓ | ✓ | ✓ | ✓ | x |
|  | Zhang S et al. (2020) | x | ✓ | x | ✓ | ✓ | x | ✓ | x | ✓ | ✓ | ✓ | ✓ | ✓ | ✓ | ✓ | ✓ | ✓ | ✓ | x | x | ✓ | ✓ | ✓ | ✓ | ✓ | ✓ | x | ✓ | x |
|  | Zhao Z et al. (2020) | x | ✓ | x | ✓ | ✓ | x | ✓ | x | ✓ | ✓ | ✓ | ✓ | ✓ | x | ? | x | ✓ | ✓ | x | ✓ | ? | ✓ | ? | ? | ✓ | ✓ | x | ✓ | x |
|  | Zhou J et al. (2021) | x | ✓ | x | ✓ | ✓ | x | ✓ | x | ✓ | ✓ | ✓ | ✓ | ✓ | x | ? | x | ✓ | ✓ | ✓ | ? | x | ✓ | ? | ? | ✓ | ? | x | ✓ | x |
|  | Zhou Y et al. (2020) | x | ✓ | x | ✓ | ✓ | x | ✓ | x | ✓ | ✓ | ✓ | ✓ | ✓ | ✓ | ? | x | ✓ | ✓ | ✓ | ? | ✓ | x | ? | ✓ | ✓ | ✓ | x | ✓ | x |
|  | Zou X et al. (2020) | x | ✓ | x | ✓ | ? | x | ✓ | x | ✓ | ✓ | ✓ | ? | ✓ | x | ✓ | x | ✓ | ? | ✓ | ✓ | ? | x | ? | ? | ? | ✓ | x | ✓ | x |
|  | Aguirre-García et al. (2021) | x | ✓ | x | ✓ | ✓ | ? | ✓ | ? | ✓ | ✓ | ✓ | ? | ✓ | ? | ? | ? | ✓ | ✓ | ✓ | ✓ | ✓ | x | ✓ | x | ✓ | x | x | ✓ | x |
|  | Alessandri et al. (2022) | x | ✓ | x | ✓ | ✓ | ? | ? | ? | ✓ | ✓ | ✓ | x | ✓ | x | ? | x | ✓ | ✓ | ✓ | ✓ | x | ✓ | x | x | ✓ | ✓ | x | ✓ | x |
|  | Downing et al. (2021) | x | ✓ | x | ✓ | ✓ | ? | ✓ | ? | ✓ | ✓ | ✓ | ? | ✓ | ? | ✓ | ? | ✓ | x | ✓ | ✓ | x | x | ? | x | ✓ | x | x | ✓ | x |
|  | Shanbhag et al. (2021) | x | ✓ | x | ✓ | ? | ? | ✓ | ? | x | ✓ | ✓ | ? | ✓ | ? | ? | ? | ✓ | x | ? | ✓ | x | ? | ? | x | x | x | x | x | x |
|  | Venturini et al. (2022) | x | ✓ | x | ✓ | ✓ | ? | ✓ | ? | ✓ | ✓ | ✓ | ? | ✓ | ? | ✓ | ? | ✓ | ✓ | ✓ | ✓ | x | x | ? | ✓ | ✓ | ? | x | ✓ | x |
|  | Shanbehzadeh et al. (2022) | x | ? | x | ✓ | ✓ | ? | ✓ | ? | ✓ | ✓ | ✓ | ? | ✓ | ? | ? | ? | ✓ | ✓ | ✓ | ✓ | x | x | ? | x | ✓ | x | x | ✓ | x |
|  | Suastika et al. (2021) | ✓ | ✓ | ✓ | ✓ | ✓ | ✓ | ✓ | ✓ | ✓ | ✓ | ✓ | ✓ | ✓ | ✓ | ? | ? | ✓ | ✓ | x | ✓ | x | ✓ | x | x | x | ? | x | ✓ | x |
|  | Alberdi-Iglesias et al. (2021) | x | ✓ | x | ✓ | ✓ | ? | ✓ | ? | ✓ | ✓ | ✓ | ? | ✓ | ? | ? | ? | ✓ | ✓ | ✓ | ✓ | x | ? | ? | x | ✓ | x | x | ✓ | x |
|  | Assal et al. (2022) | x | ✓ | x | ✓ | ✓ | ? | ✓ | ? | ✓ | ✓ | ✓ | ? | ✓ | ? | ? | ? | ✓ | x | ✓ | ✓ | x | ✓ | x | x | x | ? | x | ✓ | x |
|  | Gómez et al (2021) | x | ✓ | x | ✓ | ✓ | ? | x | x | ✓ | ✓ | ✓ | ? | ✓ | ? | ? | ? | ✓ | ✓ | ✓ | ✓ | x | ✓ | x | x | ✓ | ? | x | ✓ | x |
|  | Shalmon et al. (2022) | x | ✓ | x | ✓ | ✓ | ? | ✓ | ? | ✓ | ✓ | ✓ | ? | ✓ | ? | ? | ? | ✓ | x | x | ✓ | x | ✓ | ? | ✓ | ✓ | ? | x | ✓ | x |
|  | Shankar et al. (2021) | x | ✓ | x | ✓ | ✓ | ? | ✓ | ? | ✓ | ✓ | ✓ | ? | ✓ | ? | ? | ? | ✓ | x | ✓ | x | x | ✓ | x | ✓ | ✓ | ✓ | x | ✓ | x |
|  | Shi et al. (2022) | x | ✓ | x | ✓ | ✓ | ? | ✓ | ? | ✓ | ✓ | ✓ | ✓ | ✓ | ✓ | ? | ? | ✓ | x | ✓ | ✓ | x | ✓ | ? | ✓ | ✓ | ✓ | x | ✓ | x |
|  | Tang et al. (2021) | x | ✓ | x | ✓ | ✓ | ? | ✓ | ? | ✓ | ✓ | ✓ | ? | ✓ | ? | ? | ? | ✓ | ✓ | x | ✓ | x | ✓ | ✓ | ✓ | ✓ | ? | x | ✓ | x |
|  | Vela et al. (2022) | x | ✓ | x | ✓ | ✓ | ? | ✓ | ? | ✓ | ✓ | ✓ | ? | ✓ | ? | ? | ? | ✓ | ✓ | ✓ | ✓ | ✓ | ✓ | ? | ✓ | ✓ | ? | x | ✓ | x |
|  | Wong et al. (2021) | x | ✓ | x | ✓ | ✓ | ? | ✓ | ? | ✓ | ✓ | ✓ | ? | ✓ | ? | ? | ? | ✓ | ✓ | ✓ | ✓ | ✓ | ? | ? | ✓ | ✓ | ? | x | ✓ | x |
|  | Xiong et al. (2022) | x | ✓ | x | ✓ | ✓ | ? | ✓ | ? | ✓ | ✓ | ✓ | ? | ✓ | ? | ? | ? | ✓ | x | ✓ | ✓ | x | ? | ? | x | ✓ | ✓ | x | ✓ | x |
|  | Zhang et al. (2022) | x | ✓ | x | ✓ | ✓ | ? | ✓ | ? | ✓ | ✓ | ✓ | ? | ✓ | ? | ? | ? | ✓ | x | x | ✓ | x | ✓ | ? | ✓ | ✓ | ? | x | ✓ | x |
|  | Zhao et al. (2022) | x | ✓ | x | ✓ | ✓ | ? | ✓ | ? | ✓ | ✓ | ✓ | ? | ✓ | ? | ? | ? | ✓ | x | ✓ | ✓ | ✓ | ✓ | ? | ✓ | ✓ | ✓ | x | ✓ | x |
|  | Ahmed et al. (2022) | x | ✓ | x | ✓ | ✓ | ? | ✓ | ? | ✓ | ✓ | ✓ | ? | ✓ | ? | ? | ? | ✓ | ✓ | x | ✓ | ✓ | ? | x | x | x | x | x | ✓ | x |
|  | Alhamar et al. (2022) | x | ✓ | x | ✓ | ✓ | ? | ✓ | ? | ✓ | ✓ | ✓ | ✓ | ✓ | ✓ | ? | ? | ✓ | ✓ | x | ✓ | x | ✓ | ? | x | ✓ | ✓ | x | ✓ | x |
|  | Alkhasawneh et al. (2021) | x | ✓ | x | ✓ | ? | ? | ✓ | ? | x | ✓ | ✓ | ✓ | ✓ | ✓ | ? | ? | ✓ | ✓ | ✓ | x | x | ? | x | x | x | x | x | x | x |
|  | Alvarez-Uria et al. (2022) | x | ✓ | x | ✓ | ✓ | ? | ✓ | ? | ✓ | ✓ | ✓ | ✓ | ✓ | ✓ | ? | ? | ✓ | ✓ | x | ✓ | ✓ | ✓ | x | ✓ | ✓ | ✓ | x | ✓ | x |
|  | Jamshidi et al. (2021) | x | ✓ | x | ✓ | ✓ | ? | ✓ | ? | ✓ | ✓ | ✓ | ✓ | ✓ | ✓ | ? | ? | ✓ | ✓ | ✓ | ✓ | ✓ | ? | x | ✓ | ✓ | x | x | ✓ | x |
|  | An et al. (2022) | x | ✓ | x | ✓ | ✓ | ? | ✓ | ? | ✓ | ✓ | ✓ | ✓ | ✓ | ✓ | ? | ? | ✓ | x | ✓ | ✓ | x | ✓ | x | x | x | ? | x | ✓ | x |
|  | Araiza et al. (2021) | x | ✓ | x | ✓ | ✓ | ? | ✓ | ? | ✓ | ✓ | ✓ | ? | ✓ | ? | ? | ? | ✓ | x | ✓ | ? | x | ✓ | ✓ | x | x | ? | x | ✓ | x |
|  | Asaduzzaman et al. (2022) | x | ✓ | x | ✓ | ✓ | ? | ✓ | ? | ✓ | ✓ | ✓ | ✓ | ✓ | ✓ | ? | ? | ✓ | x | ✓ | ✓ | x | ✓ | x | x | x | ? | x | ✓ | x |
|  | Ashkenazi et al. (2022) | x | ✓ | x | ✓ | ✓ | ? | ✓ | ? | ✓ | ✓ | ✓ | ? | ✓ | ? | ? | ? | ✓ | x | x | ✓ | x | ✓ | x | x | x | ? | x | ✓ | x |
|  | Asmarawati et al. (2022) | ✓ | ? | ? | ✓ | ? | ✓ | ? | ? | x | ✓ | ✓ | ? | ✓ | ? | ? | ? | ✓ | x | ✓ | ? | x | ✓ | x | x | x | ? | x | x | x |
|  | Aygun et al. (2021) | x | ✓ | x | ✓ | ✓ | ? | ✓ | ? | ✓ | ✓ | ✓ | ✓ | ✓ | ✓ | ✓ | ✓ | ✓ | x | ✓ | ✓ | x | ? | x | x | x | x | x | ✓ | x |
|  | Ayvat et al. (2022) | x | ✓ | x | ✓ | ✓ | ? | ✓ | ? | ✓ | ✓ | ✓ | ✓ | ✓ | ✓ | ? | ? | ✓ | ? | ? | ✓ | x | ✓ | x | x | x | ? | x | ✓ | x |
|  | Bae et al. (2021) | x | ✓ | x | ✓ | ✓ | ? | x | x | ✓ | ✓ | ✓ | ? | ✓ | ? | ? | ? | ✓ | ? | ? | ? | x | ? | x | x | ✓ | x | x | ✓ | x |
|  | Baikpour et al. (2022) | x | ✓ | x | ✓ | ✓ | ? | ✓ | ? | ✓ | ✓ | ✓ | ? | ✓ | ? | ? | ? | ✓ | ✓ | ✓ | ✓ | x | ✓ | x | x | ✓ | x | x | ✓ | x |
|  | Bartoszko et al. (2022) | x | ✓ | x | ✓ | ✓ | ? | ✓ | ? | ✓ | ✓ | ✓ | ? | ✓ | ? | ✓ | ? | ✓ | x | ✓ | ✓ | ✓ | ✓ | x | ✓ | ✓ | ✓ | x | ✓ | x |
|  | Beigmohammadi et al. (2022) | x | ✓ | x | ✓ | ✓ | ? | x | x | ✓ | ✓ | ✓ | ✓ | ✓ | ✓ | ? | ? | ✓ | x | ✓ | ✓ | x | x | x | x | x | x | x | ✓ | x |
|  | Ben Jemaa et al. (2022) | x | ✓ | x | ✓ | ✓ | ? | ✓ | ? | ✓ | ✓ | ✓ | ✓ | ✓ | ✓ | ? | ? | ✓ | x | ✓ | ✓ | x | ✓ | x | x | x | ? | x | ✓ | x |
|  | Bengelloun et al. (2022) | x | ✓ | x | ✓ | ✓ | ? | x | x | ✓ | ✓ | ✓ | ✓ | ✓ | ✓ | ? | ? | ✓ | x | ✓ | ? | x | ✓ | ✓ | x | x | ? | x | ✓ | x |
|  | Besutti et al. (2021) | x | ✓ | x | ✓ | ✓ | ? | x | x | ✓ | ✓ | ✓ | ✓ | ✓ | ✓ | ? | ? | ✓ | x | ✓ | ? | x | ✓ | x | x | ✓ | ? | x | ✓ | x |
|  | Bezerra et al. (2021) | ✓ | ✓ | ✓ | ✓ | ✓ | ✓ | x | x | ✓ | ✓ | ✓ | ✓ | ✓ | ✓ | ? | ? | ✓ | x | ✓ | ? | x | ✓ | ✓ | x | x | ? | x | ✓ | x |
|  | Bodolea et al. (2022) | x | ✓ | x | ✓ | ✓ | ? | x | x | ✓ | ✓ | ✓ | ✓ | ✓ | ✓ | ? | ? | ✓ | x | ✓ | ✓ | x | ✓ | ✓ | x | x | ? | x | ✓ | x |
|  | He et al. (2021) | x | ✓ | x | ✓ | ✓ | ? | ✓ | ? | ✓ | ✓ | ✓ | ✓ | ✓ | ✓ | ? | ? | ✓ | ✓ | ✓ | x | x | ✓ | x | x | x | ? | x | ✓ | x |
|  | Bradley et al. (2022) | x | ✓ | x | ✓ | ✓ | ? | ✓ | ? | ✓ | ✓ | ✓ | ✓ | ✓ | ✓ | ? | ? | ✓ | ? | x | x | x | x | x | x | ✓ | x | x | ✓ | x |
|  | Brook et al. (2021) | x | ✓ | x | ✓ | ✓ | ? | ✓ | ? | ✓ | ✓ | ✓ | ? | ✓ | ? | ? | ? | ✓ | x | ✓ | x | x | x | x | x | x | x | x | ✓ | x |
|  | Ceci et al. (2021) | x | ✓ | x | ✓ | ✓ | ? | ✓ | ? | ✓ | ✓ | ✓ | ✓ | ✓ | ✓ | ? | ? | ✓ | x | ? | x | x | x | x | x | x | x | x | ✓ | x |
|  | Cervantes‑Alvarez et al. (2022) | ✓ | ✓ | ✓ | ✓ | ✓ | ✓ | x | x | ✓ | ✓ | ✓ | ? | ✓ | ✓ | ? | ? | ✓ | x | ✓ | ? | x | ✓ | x | x | x | x | x | ✓ | x |
|  | Chang et al. (2022) | x | ✓ | x | ✓ | ✓ | ? | ? | ? | ✓ | ✓ | ✓ | ? | ✓ | ? | ? | ? | ✓ | ✓ | x | ✓ | x | ✓ | x | ✓ | ✓ | ? | x | ✓ | x |
|  | Chen et al. (2021) | x | ✓ | x | ✓ | ✓ | ? | ✓ | ? | ✓ | ✓ | ✓ | ? | ✓ | ? | ? | ? | ✓ | ✓ | ✓ | ✓ | ✓ | ✓ | x | ✓ | ✓ | ? | x | ✓ | x |
|  | Chikhalkar et al. (2022) | ✓ | ✓ | ✓ | ✓ | ✓ | ✓ | ✓ | ✓ | ✓ | ✓ | ✓ | ✓ | ✓ | ✓ | ? | ? | ✓ | x | x | ✓ | x | x | x | x | x | x | x | ✓ | x |
|  | Chou et al. (2022) | x | ✓ | x | ✓ | ✓ | ? | ✓ | ? | ✓ | ✓ | ✓ | ✓ | ✓ | ✓ | ? | ? | ✓ | ? | ✓ | ✓ | ✓ | ✓ | x | ✓ | ✓ | ✓ | x | ✓ | x |
|  | Cidade P. et al. (2022) | x | ✓ | x | ✓ | ✓ | ? | ? | ? | ✓ | ✓ | ✓ | ? | ✓ | ? | ? | ? | ✓ | ✓ | ✓ | ✓ | x | x | ✓ | x | x | x | x | ✓ | x |
|  | Citu et al. (2022) | x | ✓ | x | ✓ | ✓ | ? | ✓ | ? | ✓ | ✓ | ✓ | ? | ✓ | ? | ? | ? | ✓ | x | ✓ | ✓ | x | ✓ | x | x | x | x | x | ✓ | x |
|  | Citu et al. (2022)-1686 | x | ✓ | x | ✓ | ✓ | ? | ✓ | ? | ✓ | ✓ | ✓ | ? | ✓ | ? | ? | ? | ✓ | x | ✓ | x | x | ✓ | x | ✓ | x | ? | x | ✓ | x |
|  | Comoglu et al. (2022) | x | ✓ | x | ✓ | ✓ | ? | ✓ | ? | ✓ | ✓ | ✓ | ✓ | ✓ | ✓ | ? | ? | ✓ | x | ✓ | ✓ | x | ? | x | x | x | x | x | ✓ | x |
|  | Cruciata et al. (2022) | x | ✓ | x | ✓ | ✓ | ? | ✓ | ? | ✓ | ✓ | ✓ | ? | ✓ | ? | ? | ? | ✓ | ✓ | ✓ | ✓ | x | ✓ | x | ✓ | x | ? | x | ✓ | x |
|  | Durmus Kocak et al. (2021) | x | ✓ | x | ✓ | ✓ | ? | x | x | ✓ | ✓ | ✓ | ? | ✓ | ? | ? | ? | ✓ | x | ✓ | x | x | ? | x | x | x | x | x | ✓ | x |
|  | Gorgojo-Galindo et al. (2021) | ✓ | ✓ | ✓ | ✓ | ✓ | ✓ | ? | ? | ✓ | ✓ | ✓ | ✓ | ✓ | ✓ | ? | ? | ✓ | x | x | ✓ | x | x | x | x | ✓ | x | x | ✓ | x |
|  | Hashem et al. (2021) | x | ✓ | x | ✓ | ✓ | ? | ✓ | ? | ✓ | ✓ | ✓ | ✓ | ✓ | ? | ? | ? | ✓ | x | x | ✓ | x | x | x | x | x | x | x | ✓ | x |
|  | Heo et al. (2021) | x | ✓ | x | ✓ | ✓ | ? | ✓ | ? | ✓ | ✓ | ✓ | ? | ✓ | ? | ? | ? | ✓ | ✓ | ✓ | x | x | ✓ | x | x | ✓ | ? | x | ✓ | x |
|  | Monterde et al. (2021) | x | ✓ | x | ✓ | ✓ | ? | ✓ | ? | ✓ | ✓ | ✓ | ? | ✓ | ? | ? | ? | ✓ | ✓ | ✓ | ✓ | x | ✓ | x | x | ✓ | ? | x | ✓ | x |
|  | Muto et al. (2021) | x | x | x | x | ✓ | ? | x | x | ✓ | ✓ | ✓ | x | ✓ | ? | ? | ? | ✓ | x | x | x | x | x | x | x | ✓ | x | x | x | x |
|  | Ocho et al. (2022) | x | ✓ | x | ✓ | ✓ | ? | ✓ | ? | ✓ | ✓ | ✓ | ? | ✓ | ? | ? | ? | ✓ | x | ✓ | ✓ | x | ✓ | ✓ | x | x | ? | x | ✓ | x |
|  | Özdemir et al. (2021) | x | ✓ | x | ✓ | ✓ | ? | ? | ? | ✓ | ✓ | ✓ | ✓ | ✓ | ? | ? | ? | ✓ | ✓ | ✓ | x | x | ✓ | x | ✓ | x | ? | x | ✓ | x |
|  | Özdemir et al. (2022) | ✓ | ✓ | ✓ | ✓ | ✓ | ✓ | ✓ | ✓ | ✓ | ✓ | ✓ | ✓ | ✓ | ✓ | ? | ? | ✓ | x | ✓ | ✓ | x | ? | x | x | x | x | x | ✓ | x |
|  | Ozger et al. (2021) | ✓ | ✓ | ✓ | ✓ | ✓ | ✓ | ✓ | ✓ | ✓ | ✓ | ✓ | ✓ | ✓ | ✓ | ? | ? | ✓ | x | ✓ | ? | x | ? | x | x | x | x | x | ✓ | x |
|  | Pasculli et al. (2021) | x | ✓ | x | ✓ | ✓ | ? | x | x | ✓ | ✓ | ✓ | ✓ | ✓ | ? | ? | ? | ✓ | x | ✓ | ? | x | ✓ | ✓ | x | x | ? | x | ✓ | x |
|  | Patel et al. (2021) | x | ✓ | x | ✓ | ✓ | ? | ? | ? | ✓ | ✓ | ✓ | ✓ | ✓ | ? | ? | ? | ✓ | x | ✓ | ✓ | x | x | x | ✓ | x | x | x | ✓ | x |
|  | Peng et al. (2022) | x | ✓ | x | ✓ | ✓ | ? | ? | ? | ✓ | ✓ | ✓ | ? | ✓ | ? | ? | ? | ✓ | x | ✓ | ✓ | x | ✓ | ✓ | ✓ | ✓ | ✓ | x | ✓ | x |
|  | Plečko et al. (2021) | x | ✓ | x | ✓ | ✓ | ? | x | x | ✓ | ✓ | ✓ | ? | ✓ | ? | ? | ? | ✓ | x | ✓ | x | ✓ | ✓ | x | ✓ | ✓ | ✓ | x | ✓ | x |
|  | Prasetya et al. (2021) | x | ✓ | x | ✓ | ✓ | ? | ✓ | ? | ✓ | ✓ | ✓ | ? | ✓ | ? | ? | ? | ✓ | x | ✓ | ✓ | x | x | x | x | x | x | x | ✓ | x |
|  | Rinderknecht et al. (2021) | x | ✓ | x | ✓ | ✓ | ? | ✓ | ? | ✓ | ✓ | ? | ? | ✓ | ? | ? | ? | ✓ | ✓ | ? | x | x | ✓ | x | ✓ | ✓ | ? | x | ✓ | x |
|  | Torres-Macho et al. (2021) | ✓ | ✓ | ✓ | ✓ | ✓ | ✓ | x | x | ✓ | ✓ | ✓ | ? | ✓ | ✓ | ? | ? | ✓ | x | ✓ | ? | x | ? | x | ✓ | x | x | x | ✓ | x |
|  | Varghese et al. (2021) | x | ✓ | x | ✓ | ✓ | ? | x | x | ✓ | ✓ | ✓ | ? | ✓ | ? | ? | ? | ✓ | ? | ✓ | ? | x | ? | x | x | ✓ | x | x | ✓ | x |
|  | Haji Aghajani et al. (2021) | x | ✓ | x | ✓ | ✓ | ? | ✓ | ? | ✓ | ✓ | ✓ | ? | ✓ | ? | ? | ? | ✓ | ✓ | x | ✓ | x | ✓ | x | ✓ | ✓ | ? | x | ✓ | x |
|  | Ak et al. (2021) | x | ✓ | x | ✓ | ✓ | ? | ✓ | ? | ✓ | ✓ | ✓ | ✓ | ✓ | ✓ | ✓ | ✓ | ✓ | x | ✓ | ✓ | x | ? | x | x | x | x | x | ✓ | x |
|  | Al Mutair et al. (2021) | x | ✓ | x | ✓ | ✓ | ? | ✓ | ? | ✓ | ✓ | ✓ | ? | ✓ | ? | ? | ? | ✓ | x | ✓ | ✓ | x | ✓ | ✓ | x | x | ? | x | ✓ | x |
|  | Alkaabi et al. (2021) | x | ✓ | x | ✓ | ✓ | ? | ✓ | ? | ✓ | ✓ | ✓ | ? | ✓ | ? | ? | ? | ✓ | x | ✓ | x | x | x | x | ✓ | ✓ | ✓ | x | ✓ | x |
|  | Aly et al. (2021) | x | ✓ | x | ✓ | ✓ | ? | ? | ? | ✓ | ✓ | ✓ | ? | ✓ | ? | ? | ? | ✓ | x | ? | ✓ | x | x | ✓ | x | x | x | x | ✓ | x |
|  | Aznar-Gimeno et al. (2021) | x | ✓ | x | ✓ | ? | ? | x | x | x | ✓ | ✓ | ? | ✓ | ? | ? | ? | ✓ | x | x | x | x | ? | x | ✓ | ✓ | x | x | x | x |
|  | Banoei M et al. (2021) | x | ✓ | x | ✓ | ? | ? | ? | ? | x | ✓ | ✓ | ✓ | ✓ | ✓ | ? | ? | ✓ | x | x | x | ? | ✓ | x | x | ✓ | ? | x | x | x |
|  | Bennett M et al. (2021) | x | ✓ | x | ✓ | ✓ | ? | ? | ? | ✓ | ✓ | ✓ | ? | ✓ | ? | ? | ? | ✓ | ✓ | ✓ | x | x | ✓ | x | x | ✓ | ? | x | ✓ | x |
|  | Faria et al. (2021) | ✓ | ✓ | ✓ | ✓ | ? | ✓ | x | x | ✓ | ? | ? | ? | ? | ? | ? | ? | ✓ | x | x | ✓ | x | ? | x | x | ✓ | x | x | ✓ | x |
|  | Ganesan et al. (2021) | x | ✓ | x | ✓ | ✓ | ? | ✓ | ? | ✓ | ✓ | ✓ | ✓ | ✓ | ✓ | ? | ? | ✓ | x | x | ✓ | x | ? | x | x | x | x | x | ✓ | x |
|  | Garrafa et al. (2021) | x | x | x | x | ✓ | ? | x | x | ✓ | ✓ | ✓ | ✓ | ✓ | ✓ | ? | ? | ✓ | ✓ | ? | ✓ | ✓ | ? | x | x | ✓ | x | x | x | x |
|  | Geraili et al. (2022) | x | ✓ | x | ✓ | ✓ | ? | ✓ | ? | ✓ | ✓ | ✓ | ? | ✓ | ? | ? | ? | ✓ | ✓ | ✓ | ✓ | x | x | ✓ | x | x | x | x | ✓ | x |
|  | Giamarellos-Bourboulis et al. (2022) | ✓ | ✓ | ✓ | ✓ | ✓ | ✓ | x | x | ✓ | ✓ | ✓ | ? | ✓ | ? | ? | ? | ✓ | ? | x | x | x | ✓ | ✓ | x | ✓ | ? | x | ✓ | x |
|  | Golukhova et al. (2022) | ✓ | ✓ | ✓ | ✓ | ✓ | ✓ | x | x | ✓ | ✓ | ✓ | ? | ✓ | ? | ? | ? | ✓ | x | ✓ | ? | x | ✓ | x | x | ✓ | ? | x | ✓ | x |
|  | González-Flores et al. (2021) | x | ✓ | x | ✓ | ✓ | ? | ✓ | ? | ✓ | ✓ | ✓ | ? | ✓ | ? | ? | ? | ✓ | x | x | ? | x | ? | x | x | x | x | x | ✓ | x |
|  | Guner et al. (2021) | x | ✓ | x | ✓ | ✓ | ? | x | x | ✓ | ✓ | ✓ | ? | ✓ | ? | ? | ? | ✓ | x | ✓ | x | ✓ | ✓ | x | ✓ | ✓ | ? | x | ✓ | x |
|  | He et al. (2021) | x | ✓ | x | ✓ | ✓ | ? | ✓ | ? | ✓ | ✓ | ✓ | ✓ | ✓ | ✓ | ? | ? | ✓ | x | ✓ | ✓ | ? | x | x | ✓ | ✓ | x | x | ✓ | x |
|  | Heber et al. (2021) | x | ✓ | x | ✓ | ✓ | ? | x | x | ✓ | ✓ | ✓ | ✓ | ✓ | ✓ | ? | ? | ✓ | ✓ | ✓ | ? | ? | ? | x | ✓ | ✓ | x | x | ✓ | x |
|  | Hiremath et al. (2021) | x | ✓ | x | ✓ | ✓ | ? | x | x | ✓ | ✓ | ✓ | ? | ✓ | ? | ? | ? | ✓ | ? | ? | x | ? | x | x | x | ✓ | x | x | ✓ | x |
|  | Dong Huang et al. (2021) | x | ✓ | x | ✓ | ✓ | ? | x | x | ✓ | ✓ | ✓ | ? | ✓ | ? | ? | ? | ✓ | ✓ | ✓ | ? | x | ✓ | x | ✓ | x | ? | x | ✓ | x |
|  | Huang Jiana et al. (2021) | x | ✓ | x | ✓ | ✓ | ? | ✓ | ? | ✓ | ✓ | ✓ | ? | ✓ | ? | ? | ? | ✓ | x | ✓ | ? | x | x | x | ✓ | x | x | x | ✓ | x |
|  | Huespe et al. (2021) | x | ✓ | x | ✓ | ✓ | ? | ✓ | ? | ✓ | ✓ | ✓ | ? | ✓ | ? | ✓ | ? | ✓ | x | ? | ? | x | ? | x | ✓ | x | x | x | ✓ | x |
|  | Jain et al. (2021) | x | ? | x | x | ? | ? | ? | ? | x | ✓ | ✓ | ✓ | ✓ | ✓ | ? | ? | ✓ | x | ? | ? | x | x | x | x | x | x | x | x | x |
|  | Kar et al. (2021) | x | ✓ | x | ✓ | ✓ | ? | ? | ? | ✓ | ✓ | ✓ | ✓ | ✓ | ? | ✓ | ? | ✓ | x | ✓ | x | x | ✓ | ✓ | x | ✓ | ? | x | ✓ | x |
|  | Kilercik et al. (2021) | x | ✓ | x | ✓ | ✓ | ? | ✓ | ? | ✓ | ✓ | ✓ | ? | ✓ | ? | ? | ? | ✓ | x | ✓ | ? | x | ✓ | x | x | x | ? | x | ✓ | x |
|  | Klaveren et al. (2021) | x | ✓ | x | ✓ | ✓ | ? | ✓ | ? | ✓ | ✓ | ✓ | ? | ✓ | ? | ✓ | ? | ✓ | ✓ | x | ✓ | ✓ | ✓ | x | ✓ | ✓ | ✓ | x | ✓ | x |
|  | Li et al. (2021) | x | ✓ | x | ✓ | ✓ | ? | ✓ | ? | ✓ | ✓ | ✓ | ✓ | ✓ | ? | ? | ? | ✓ | ? | ? | x | ✓ | x | x | ✓ | ✓ | ? | x | ✓ | x |
|  | Cui et al. (2022) | x | x | x | ✓ | ? | x | ✓ | x | ✓ | ✓ | ✓ | ? | ✓ | ? | ? | x | ✓ | x | ✓ | x | ? | ✓ | ? | x | ✓ | ? | x | ✓ | x |
|  | Ergenç et al. (2022) | x | ✓ | x | ✓ | ✓ | x | ✓ | x | ✓ | ✓ | ✓ | ✓ | ✓ | ✓ | x | x | ✓ | ? | ? | ? | ? | x | x | x | x | ? | x | ✓ | x |
|  | Falandry et al. (2022) | ? | ✓ | ? | ✓ | ✓ | ✓ | ? | ? | ✓ | ✓ | ✓ | ✓ | x | x | ✓ | x | ✓ | ✓ | ✓ | ✓ | x | x | x | ? | ✓ | ? | x | ✓ | x |
|  | Gordon et al. (2022) | x | x | x | ✓ | ✓ | x | ✓ | x | ✓ | ✓ | ✓ | ✓ | ✓ | ✓ | ✓ | ✓ | ✓ | ✓ | ✓ | ? | ✓ | ✓ | ? | ✓ | ✓ | ✓ | ? | ✓ | x |
|  | Gurusamy et al. (2022) | ? | x | x | ✓ | ✓ | ? | ✓ | ? | ✓ | ✓ | ? | ✓ | ✓ | x | ✓ | x | ✓ | ✓ | ✓ | ✓ | ? | ✓ | ? | x | x | ✓ | x | ✓ | x |
|  | Gutierrez-Camacho et al. (2022) | x | ✓ | x | ✓ | ✓ | x | ✓ | x | ✓ | ✓ | ✓ | ✓ | ✓ | x | ✓ | x | ✓ | x | ✓ | ✓ | ? | ? | ? | x | x | ✓ | x | ✓ | x |
|  | Haimovich et al. (2020) | x | ✓ | x | ✓ | ✓ | x | ✓ | x | ✓ | ✓ | ✓ | x | ✓ | x | ✓ | x | ✓ | ✓ | x | ? | x | ✓ | ? | x | ✓ | ? | x | ✓ | x |
|  | Han et al. (2022) | x | ✓ | x | ✓ | ✓ | x | ✓ | x | ✓ | ✓ | ✓ | ✓ | ✓ | x | ✓ | x | ✓ | x | ✓ | x | x | ✓ | ? | x | x | ✓ | x | ✓ | x |
|  | Hao et al. (2022) | ? | ✓ | ? | ✓ | ✓ | ? | ? | ? | ✓ | ? | ? | ? | ✓ | ? | ✓ | ? | ? | ✓ | ✓ | x | ✓ | ✓ | ? | ✓ | ✓ | ✓ | x | ✓ | x |
|  | Hassan et al. (2022) | ✓ | ✓ | ✓ | ✓ | ✓ | ✓ | ✓ | ✓ | ✓ | ✓ | ✓ | ✓ | ✓ | ✓ | ✓ | ✓ | ✓ | x | ✓ | ✓ | ✓ | ✓ | ✓ | ✓ | ✓ | ✓ | x | ✓ | x |
|  | Hippisley-Cox et al. (2021) | ✓ | ✓ | ✓ | ✓ | ? | ✓ | ✓ | ? | ✓ | ✓ | ✓ | ✓ | ✓ | ✓ | ✓ | ✓ | ✓ | ✓ | ✓ | ✓ | ✓ | ✓ | ✓ | ✓ | x | ✓ | x | ✓ | x |
|  | Hohl et al. (2022) | ? | x | x | ✓ | ✓ | ✓ | ✓ | ✓ | ✓ | ✓ | ✓ | ✓ | ✓ | ✓ | ✓ | ✓ | ✓ | ✓ | ✓ | x | ✓ | ✓ | ? | ✓ | ? | x | x | ✓ | x |
|  | Hormanstofer et al. (2021) | ✓ | ? | ? | ? | ✓ | ✓ | ✓ | ✓ | ✓ | ✓ | ✓ | ✓ | ✓ | x | ? | x | ✓ | x | x | ? | ? | x | x | x | ✓ | ✓ | x | ? | x |
|  | Huang et al. (2021) -3846 | x | ✓ | x | ? | ✓ | x | ✓ | x | ✓ | ✓ | ✓ | ✓ | ✓ | x | ? | x | ✓ | x | ? | x | x | ✓ | x | x | x | ✓ | x | ? | x |
|  | Huang et al. (2021) | x | x | x | ✓ | ✓ | ? | ✓ | ? | ✓ | ? | ? | ✓ | ✓ | x | ? | x | ✓ | x | ✓ | x | x | x | ? | ✓ | ✓ | ✓ | x | ✓ | x |
|  | Jalalvand et al. (2022) | x | ? | x | ✓ | ✓ | x | ? | x | ✓ | ✓ | ✓ | ✓ | ✓ | ✓ | ✓ | ✓ | ✓ | x | ✓ | ✓ | ? | x | ? | ✓ | x | x | x | ✓ | x |
|  | Jiang et al. (2022) | x | x | x | ✓ | ? | x | ✓ | x | ✓ | ✓ | ? | ✓ | ✓ | ? | ✓ | ? | ✓ | x | x | x | ? | x | ? | ✓ | x | ✓ | x | ✓ | x |
|  | Kamran et al. (2022) | x | x | x | ✓ | ? | x | ✓ | x | ✓ | ✓ | ? | x | ✓ | x | ✓ | x | ✓ | ✓ | ✓ | x | x | ✓ | ? | ✓ | ✓ | ? | x | ✓ | x |
|  | Ketenci et al. (2022) | x | ✓ | x | ✓ | ✓ | x | ✓ | x | ✓ | ? | ? | ? | ? | x | ? | x | ✓ | x | ✓ | ✓ | ? | ? | ? | x | x | ? | x | ✓ | x |
|  | Kim et al. (2022) | ✓ | ? | ? | ✓ | ? | ✓ | ✓ | ? | ✓ | ✓ | ✓ | ✓ | ✓ | x | ✓ | x | ✓ | x | ✓ | ✓ | ? | ✓ | ✓ | x | x | x | x | ✓ | x |
|  | Klen et al. (2022) | x | ✓ | x | ✓ | ? | x | ✓ | x | ✓ | ✓ | ✓ | ✓ | ✓ | x | ✓ | x | ✓ | ? | ? | ✓ | ✓ | ✓ | ? | ✓ | ✓ | ? | ? | ✓ | x |
|  | Knight et al. (2022) | ✓ | ✓ | ✓ | ✓ | ✓ | ✓ | ✓ | ✓ | ✓ | ✓ | ✓ | ✓ | ✓ | ✓ | ✓ | ✓ | ✓ | ✓ | ✓ | ✓ | ✓ | ✓ | ✓ | ✓ | ✓ | ✓ | ✓ | ✓ | ✓ |
|  | Kucuk et al. (2022), 1068 | x | ✓ | x | ? | ✓ | x | ? | x | ✓ | ✓ | ✓ | x | ✓ | ✓ | ✓ | x | ✓ | x | ✓ | ✓ | ? | ? | ? | ✓ | x | ✓ | x | ? | x |
|  | Laino et al. (2022) | x | ? | x | ✓ | ✓ | x | ✓ | x | ✓ | ✓ | ✓ | x | ✓ | ✓ | ✓ | x | ✓ | x | x | ✓ | ✓ | ? | ? | ✓ | ✓ | x | x | ✓ | x |
|  | Lee et al. (2022) | x | x | x | ✓ | ✓ | x | ✓ | x | ✓ | ✓ | ✓ | ✓ | ✓ | ? | ✓ | ? | ✓ | x | x | ✓ | ? | ✓ | ? | ✓ | x | ✓ | x | ✓ | x |
|  | Leoni et al. (2021) | x | ✓ | x | ✓ | ✓ | x | ✓ | x | ✓ | ✓ | ✓ | x | ✓ | ? | ✓ | x | ✓ | x | ✓ | ✓ | ? | ✓ | ? | ✓ | ✓ | ✓ | ✓ | ✓ | x |
|  | Leyderman et al. (2021), | ✓ | ✓ | ✓ | ✓ | ✓ | ✓ | ✓ | ✓ | ✓ | ✓ | ✓ | ✓ | ✓ | ? | ✓ | ? | ✓ | ? | ✓ | ✓ | ? | ✓ | ? | ✓ | x | ? | x | ✓ | x |
|  | Wen Li et al. (2022) | x | x | x | ✓ | ✓ | x | ✓ | x | ✓ | ✓ | ? | ✓ | ✓ | ? | ✓ | ? | ✓ | x | x | ✓ | ✓ | ? | ✓ | x | x | x | x | ✓ | x |
|  | Li et al. (2021) | x | ? | x | ? | ✓ | x | ✓ | x | ✓ | ✓ | ✓ | ✓ | ✓ | ✓ | ✓ | ✓ | ✓ | ✓ | ✓ | ✓ | ? | ✓ | ? | ✓ | x | x | x | ? | x |
|  | Li et al. (2022 | x | ? | x | ? | ✓ | x | ✓ | x | ✓ | x | x | x | x | x | x | x | ? | x | x | ✓ | ✓ | ✓ | ? | ✓ | ✓ | ✓ | x | ? | x |
|  | Naser et al. (2021) | x | ✓ | x | ? | ✓ | x | ✓ | x | ✓ | x | x | x | x | x | x | x | ? | x | ? | ? | ? | x | x | ? | x | x | x | ? | x |
|  | Liu et al. (2021) | x | ? | x | ✓ | ✓ | x | ? | x | ✓ | ✓ | ✓ | ✓ | ✓ | ? | ? | ? | ✓ | ? | x | ? | ? | x | x | ✓ | x | x | x | ✓ | x |
|  | Martin Rodriquez et al. (2021) | ✓ | ✓ | ✓ | ? | ✓ | ✓ | ? | ? | ✓ | ? | ✓ | ? | ✓ | ✓ | ? | ? | ✓ | x | ✓ | ✓ | ? | x | x | x | x | ✓ | x | ? | x |
|  | Lyons et al. (2022) | x | ? | x | ✓ | ? | x | ✓ | x | ? | ✓ | ✓ | ✓ | ✓ | ✓ | ? | ? | ✓ | ✓ | ✓ | ? | ✓ | ✓ | ? | ✓ | ? | ? | x | ? | x |
|  | Pournazari et al. (2020) | ? | ? | ? | ? | ✓ | ✓ | ? | ? | ✓ | ✓ | ✓ | ✓ | ✓ | ✓ | ✓ | ✓ | ✓ | x | ? | ? | ? | ? | ? | ✓ | ✓ | ✓ | x | ? | x |
|  | Marincu et al.(2021) | ? | ? | ? | ? | ✓ | x | ✓ | x | ✓ | ✓ | ✓ | ✓ | ✓ | ✓ | ✓ | ✓ | ✓ | ✓ | ? | ✓ | ? | ✓ | ? | ? | x | ✓ | x | ? | x |
|  | Martin Rodriguez et al. (2022) | x | ? | x | ✓ | ✓ | ✓ | ✓ | ✓ | ✓ | ✓ | ✓ | ✓ | ✓ | ✓ | ✓ | ? | ✓ | ✓ | ✓ | ✓ | ? | ? | ? | ✓ | ? | ? | ? | ✓ | x |
|  | Morello et al.(2022) | ✓ | ? | ? | ✓ | ✓ | ✓ | ✓ | ✓ | ✓ | ✓ | ✓ | ✓ | ✓ | ✓ | ✓ | ✓ | ✓ | ✓ | ✓ | ✓ | ? | ? | ? | ✓ | ? | ? | ? | ✓ | x |
|  | Moulaei et al. (2022) | x | ? | x | ✓ | ✓ | x | ✓ | x | ✓ | ✓ | ✓ | ✓ | ✓ | ✓ | ✓ | ✓ | ✓ | x | ✓ | x | x | ? | x | ? | ? | ? | x | ✓ | x |
|  | Valente, Silva et al. (2021) | x | ✓ | x | ✓ | ✓ | x | ? | x | ✓ | ✓ | ? | ? | ✓ | ? | ? | ? | ? | x | x | ? | x | ? | x | ? | ? | ? | x | ? | x |
|  | Mousavi et al. (2021) | x | ? | x | ✓ | ✓ | x | ✓ | x | ✓ | ✓ | ✓ | ✓ | ✓ | ✓ | ✓ | ✓ | ✓ | ✓ | ✓ | ✓ | x | x | x | x | ✓ | ✓ | x | ✓ | x |
|  | Mu et al. (2022) | x | x | x | x | ✓ | x | ? | x | ✓ | x | ? | ✓ | ? | ? | ? | x | ? | x | ✓ | ? | ? | ✓ | x | ? | ✓ | x | x | x | x |
|  | Munera et al. (2022) | ✓ | ? | ? | ? | ✓ | ✓ | ? | ? | ✓ | x | x | ? | ? | ✓ | ? | x | x | ? | ✓ | ? | x | x | ? | ✓ | x |  | x | x | x |
|  | Munoz et al. (2022) | x | ? | x | ? | ✓ | x | ✓ | x | ✓ | ✓ | ✓ | ✓ | ✓ | ? | ✓ | ? | ✓ | x | ✓ | ✓ | ? | ? | ? | ✓ | ? | ? | x | ? | x |
|  | Nadasdi et al. (2022) | x | x | x | x | ? | x | ? | x | ? | ✓ | ✓ | ✓ | ✓ | ✓ | ? | ? | ✓ | ? | ✓ | ? | ✓ | ✓ | ? | x | x | x | x | x | x |
|  | Najafi et al. (2021) | x | ✓ | x | ✓ | ? | ✓ | ? | ? | ✓ | ✓ | ✓ | ✓ | ✓ | ✓ | ✓ | ✓ | ✓ | x | ✓ | ✓ | ? | x | ? | x | x | ? | x | ✓ | x |
|  | Nuevo-Ortega et al. (2022) | ✓ | ✓ | ✓ | ✓ | ? | ✓ | ✓ | ? | ✓ | ✓ | ✓ | ✓ | ✓ | ✓ | ✓ | ✓ | ✓ | ? | ✓ | ✓ | x | ✓ | ? | ? | x | x | x | ✓ | x |
|  | Ottenhoff et al. (2021) | x | ? | x | ✓ | ✓ | ✓ | ✓ | ✓ | ✓ | ✓ | ? | ✓ | ✓ | ? | ? | ? | ✓ | ? | ✓ | x | ✓ | ✓ | ? | ✓ | ✓ | ✓ | x | ✓ | x |
|  | Rozenbaum et al. (2021) | x | x | x | ? | ✓ | ✓ | ? | ? | ? | ✓ | ✓ | ✓ | ✓ | ✓ | ? | ? | ✓ | x | ✓ | ✓ | ✓ | ✓ | ? | ✓ | ✓ | ? | x | ? | x |
|  | Ponce et al. (2022) | x | ✓ | x | ✓ | ? | ? | ✓ | ? | ✓ | V | ✓ | ✓ | ✓ | ? | ✓ | ? | ✓ | x | ✓ | ? | x | x | x | x | x | ✓ | x | ✓ | x |
|  | Lombardi et al. (2021) | x | ✓ | x | ✓ | ✓ | ✓ | ✓ | ✓ | ✓ | ? | ? | ? | ? | ? | ✓ | ? | ✓ | ✓ | ✓ | ✓ | ✓ | ? | ? | ✓ | ? | ? | ? | ✓ | x |
|  | Ma et al. (2021) | x | ? | x | ✓ | ✓ | ✓ | ✓ | ✓ | ✓ | ✓ | ✓ | x | ✓ | ? | ? | x | ✓ | x | x | ✓ | ✓ | x | ? | ✓ | ✓ | ✓ | x | ✓ | x |
|  | MoghadamTabrizi et al. (2021) | x | x | x | x | ✓ | ✓ | ✓ | ✓ | ✓ | ✓ | ✓ | ✓ | ✓ | ✓ | ✓ | ✓ | ✓ | ? | ✓ | ✓ | x | ? | ? | ? | ? | ? | x | x | x |
|  | Magunia et al.(2021) | ? | x | x | x | ? | ? | ? | ? | ? | x | x | x | x | x | x | x | x | ? | ✓ | ✓ | x | ? | ? | ? | ✓ | x | x | x | x |
|  | Mahdavi et al. (2021) | x | ✓ | x | ✓ | ✓ | ✓ | ✓ | ✓ | ✓ | ✓ | ✓ | ✓ | ✓ | ✓ | ✓ | ✓ | ✓ | ? | ✓ | ✓ | ? | ? | x | ? | ✓ | ? | ? | ✓ | x |
|  | Marcolino et al. (2021) | ? | ✓ | ? | ✓ | ✓ | ✓ | ✓ | ? | ✓ | ✓ | ✓ | ✓ | ✓ | ✓ | ✓ | ✓ | ✓ | ✓ | ✓ | ✓ | ✓ | ✓ | ✓ | ? | ✓ | ✓ | ? | ✓ | ? |
|  | Murri et al. (2021) | ? | ✓ | ? | ✓ | ✓ | ✓ | ✓ | ✓ | ✓ | ✓ | ✓ | ✓ | ✓ | V | ✓ | ✓ | ✓ | x | ✓ | x | x | ? | x | ✓ | ✓ |  | x | ✓ | x |
|  | Rahman et al. (2021)-blood biomarkers ML | x | ? | x | ✓ | ? | ? | ? | ? | ? | ? | ? | ? | ? | ? | ? | ? | ? | x | x | ? | ✓ | x | x | ✓ | ✓ | ✓ | x | ? | x |
|  | Rahman et al. (2021) | x | ✓ | ? | ✓ | ? | ? | ? | ? | ✓ | ✓ | ✓ | ✓ | ✓ | ✓ | ✓ | ✓ | ✓ | x | ✓ | ✓ | ✓ | ✓ | ? | ✓ | ✓ | ✓ | x | ? | x |
|  | Riva et al. (2021) | x | ✓ | x | ✓ | ✓ | ✓ | ✓ | ✓ | ✓ | ✓ | ✓ | ✓ | ✓ | ✓ | ✓ | ✓ | ✓ | x | x | ✓ | ? | ? | ✓ | x | x | x | x | ✓ | x |
|  | Sengel et al. (2021) | x | ? | x | ? | ✓ | ✓ | ✓ | ✓ | ✓ | ✓ | ✓ | ✓ | ✓ | ✓ | ✓ | ✓ | ✓ | ? | x | ? | x | ? | ? | ✓ | ? | ? | x | ? | x |
|  | Sosa et al. (2021) | ✓ | ✓ | ✓ | ✓ | ✓ | ✓ | ? | ? | ? | ✓ | ✓ | ✓ | ✓ | ✓ | ✓ | ✓ | ✓ | ✓ | ✓ | V | V | V | ✓ | x | x | ✓ | x | ? | x |
|  | Subudhi et al. (2021) | ? | ✓ | ? | ✓ | ? | ? | ? | ? | ? | ✓ | ? | ? | ? | ? | ? | ? | ? | ? | ✓ | ✓ | ✓ | ✓ | ? | ? | ? | ? | ? | ? | ? |
|  | Surme et al. (2021) | x | ✓ | x | ✓ | ✓ | ✓ | ✓ | ✓ | ✓ | ✓ | ✓ | ✓ | ✓ | ✓ | ✓ | ✓ | ✓ | x | x | ? | ? | x | x | x | x | x | x | ✓ | x |
|  | Yu et al. (2021) | x | ✓ | x | ✓ | ✓ | ✓ | ✓ | ✓ | ✓ | ✓ | ✓ | ✓ | ✓ | ✓ | ? | ? | ✓ | ✓ | ? | ✓ | ? | ✓ | x | x | x | ✓ | x | ✓ | x |
|  | Yang et al. (2021) | x | ✓ | x | ✓ | ✓ | ? | ✓ | ? | ✓ | ✓ | ✓ | ✓ | ✓ | ? | ✓ | ? | ✓ | x | ✓ | x | ✓ | ✓ | ? | ? | ✓ | ? | x | ✓ | x |
|  | Vicka et al. (2021) | x | ✓ | x | ✓ | ? | ✓ | ✓ | ? | ✓ | ✓ | ✓ | ✓ | ✓ | ✓ | ✓ | ✓ | ✓ | ? | ? | ? | ? | NA | ? | ✓ | NA | NA | ? | ✓ | x |
|  | Valencia et al. (2021) | x | ✓ | x | ✓ | ? | ? | ✓ | ? | ✓ | ✓ | ? | ✓ | ? | ? | ? | ? | ✓ | ✓ | ✓ | ✓ | ? | NA | ? | ? | NA | NA | ? | ✓ | x |
|  | Timpau et al. (2021) | x | ? | x | ? | ✓ | ✓ | ? | ? | ✓ | ✓ | ✓ | ✓ | ✓ | ✓ | ✓ | v | ✓ | x | ✓ | ✓ | ? | ✓ | ? | ✓ | x | ? | x | ? | x |
|  | Tevald et al. (2021) | x | ? | x | ? | ✓ | ? | ? | ? | ? | ? | ? | ? | ? | ? | ? | ? | ? | ? | x | ✓ | ? | ✓ | ? | ✓ | x | ✓ | x | ? | x |
|  | Jibril et al.(2022) | x | ? | x | ? | ? | ? | ? | ? | ? | ? | ✓ | ✓ | ? | ? | ? | ? | ? | ? | ✓ | ? | x | ✓ | x | x | x | x | x | ? | x |
|  | Khari et al (2022) | ✓ | ? | ? | ✓ | ✓ | ✓ | ✓ | ✓ | ✓ | ✓ | ✓ | ✓ | ✓ | ✓ | ✓ | ✓ | V | ? | ✓ | ✓ | ? | NA | ? | ✓ | NA | NA | ? | ✓ | ? |
|  | Zahedin Kheyri et al. (2021) | x | ✓ | x | ✓ | ✓ | ? | ✓ | ? | ✓ | ✓ | ✓ | ✓ | ✓ | ? | ? | ? | ✓ | ✓ | ? | ? | ? | ? | ? | ✓ | x | ✓ | x | ✓ | x |
|  | Kibar et al. (2022) | x | ? | x | ? | ✓ | ? | ? | ? | ? | ✓ | ✓ | ✓ | ✓ | ✓ | ✓ | ✓ | ✓ | ? | ✓ | V | ? | NA | ? | ? | NA | NA | ? | x | ? |
|  | Ruscica et al. (2021) | x | ? | x | ? | ✓ | ? | ? | ✓ | ✓ | ✓ | ✓ | ✓ | ✓ | ✓ | ✓ | ✓ | ✓ | x | ✓ | x | x | x | ✓ | ? | ✓ | ✓ | x | ? | x |
|  | Nishikimi et al (2021) | x | ? | x | ✓ | ✓ | ✓ | ✓ | ✓ | ✓ | ✓ | ✓ | ✓ | ✓ | ✓ | ✓ | ✓ | ✓ | ✓ | ✓ | ✓ | ✓ | ✓ | ? | ✓ | ✓ | ✓ | ? | ✓ | x |
|  | Yilmaz et al. (2021) | ✓ | x | x | ✓ | ✓ | ✓ | ✓ | ✓ | ✓ | ✓ | ✓ | ✓ | ✓ | ✓ | ? | ? | ✓ | ? | ? | ? | ? | NA | x | x | x | ✓ | x | ✓ | x |
|  | Wirth et al. (2022) | x | ✓ | x | ✓ | ✓ | ✓ | ✓ | ✓ | ✓ | ✓ | ✓ | ✓ | ✓ | ✓ | ✓ | ✓ | ✓ | ✓ | ✓ | ✓ | ✓ | NA | ✓ | ✓ | NA | NA | ✓ | ✓ | x |
|  | Webb et al. (2022) | x | ? | x | ? | ✓ | ? | ✓ | ? | ✓ | ✓ | ✓ | ✓ | ✓ | ✓ | ✓ | ✓ | ✓ | ✓ | ✓ | x | ? | ✓ | ? | ✓ | ✓ | ✓ | x | ? | x |
|  | Vieira et al. (2022) | ✓ | ✓ | ✓ | ✓ | ✓ | ✓ | x | x | ? | ✓ | ✓ | ✓ | ✓ | ✓ | ? | ? | ✓ | x | ? | ✓ | ? | ? | ? | ✓ | x | ✓ | x | ? | x |
|  | Van de leur et al. (2022) | x | ? | x | ? | ✓ | ✓ | ✓ | ✓ | ✓ | ✓ | ✓ | ✓ | ✓ | ✓ | ✓ | ✓ | ✓ | x | ? | x | ? | ✓ | ? | ✓ | ✓ | ? | x | ? | x |
|  | Singh et al. (2022) | x | ✓ | x | ✓ | ✓ | ✓ | ✓ | ✓ | ✓ | ✓ | ✓ | ✓ | ✓ | ✓ | ✓ | ✓ | ✓ | x | ? | ? | ? | ? | ? | ? | ? | ? | x | ✓ | x |
|  | Shanbehzadeh et al. (2022) | x | ? | x | ? | ? | ✓ | ? | ? | ✓ | ✓ | ✓ | ✓ | ✓ | ✓ | ? | ? | ✓ | x | ✓ | ✓ | ✓ | x | ? | ✓ | ✓ | ? | x | x | ? |
|  | Riley et al. (2022) | x | ✓ | x | ✓ | ✓ | ✓ | ✓ | ✓ | ✓ | ✓ | ✓ | ✓ | ✓ | ✓ | ✓ | ✓ | ✓ | ? | ✓ | ✓ | ✓ | NA | ? | ✓ | NA | Na | ? | x | x |
|  | Reina Reina et al. (2022) | x | ✓ | x | ✓ | ✓ | ✓ | ✓ | ✓ | ✓ | ✓ | ✓ | ✓ | ✓ | ✓ | ✓ | ✓ | ✓ | x | ✓ | ✓ | ? | ? | ✓ | ✓ | ✓ | x | x | x | x |
|  | Raschke et al. (2022) | x | ? | x | ? | ✓ | ✓ | ✓ | ✓ | ✓ | ✓ | ✓ | ✓ | ✓ | ✓ | ✓ | ✓ | ✓ | ✓ | ✓ | x | ✓ | ✓ | ? | ✓ | ✓ | ✓ | x | x | ? |
|  | Wilfong et al. (2021) | x | ✓ | x | ✓ | ✓ | ✓ | ✓ | ✓ | ✓ | ✓ | ✓ | ✓ | ✓ | ✓ | ✓ | ✓ | ✓ | x | ✓ | ✓ | ? | NA | ✓ | ✓ | NA | NA | x | x | x |
|  | Usul et al. (2021) | x | ✓ | x | ✓ | ✓ | ? | ✓ | ? | ✓ | ? | ? | ? | ? | ? | ? | ? | ? | x | ✓ | ✓ | ? | NA | x | ✓ | NA | NA | x | x | ? |
|  | Rizzi et al. (2022) | ✓ | ✓ | ✓ | ✓ | ✓ | ✓ | ? | ? | ✓ | ? | ? | ? | ? | ? | ? | ? | ? | x | ✓ | ✓ | ? | x | x | ? | x | ✓ | x | x | ? |
|  | Regolo et al. 2022) | x | ✓ | x | ✓ | ✓ | ? | ✓ | ? | ✓ | ? | ? | ? | ? | ? | ? | ? | ? | ✓ | ? | ✓ | ? | x | x | x | x | ✓ | x | x | ? |
|  | Kucuk et al.(2022) | x | ✓ | x | ✓ | ✓ | ✓ | ? | ? | ? | ✓ | ✓ | ✓ | ✓ | ✓ | ✓ | ✓ | ✓ | x | ✓ | ✓ | ? | NA | x | x | NA | NA | x | x | ? |
|  | Adderley et al. (2022) | x | ✓ | x | ✓ | ✓ | ? | ✓ | ? | ✓ | ✓ | ✓ | ? | ✓ | ✓ | ✓ | ? | ✓ | x | ✓ | ✓ | ✓ | ? | ? | ✓ | ✓ | ✓ | x | ✓ | x |
|  | Aguadero et al.(2020) | x | ✓ | x | ✓ | ✓ | ? | ✓ | ? | ✓ | ✓ | ✓ | ? | ✓ | ? | ? | ? | ✓ | ✓ | ✓ | ✓ | x | x | x | x | x | x | x | ✓ | x |
|  | Ahirwar et al. (2022) | x | ✓ | x | ✓ | ✓ | ? | ✓ | ? | ✓ | ✓ | ✓ | ? | ✓ | ? | ? | ? | ✓ | x | ✓ | ? | x | x | x | x | x | ? | x | ✓ | x |
|  | Aletreby et al. (2021) | x | x | x | x | ✓ | ? | ✓ | ? | ✓ | ✓ | ✓ | ✓ | ✓ | ✓ | ? | ? | ✓ | ✓ | ✓ | ✓ | x | x | x | x | ✓ | x | x | ✓ | x |
|  | Churpek et al. (2021) | x | x | x | x | ✓ | ? | ✓ | ? | ✓ | ✓ | ✓ | ✓ | ✓ | ✓ | ✓ | ✓ | ✓ | ✓ | x | x | ✓ | ? | ? | ✓ | ✓ | ? | x | ✓ | x |
|  | Lee DS et al. (2021) | ✓ | ? | ? | ✓ | ? | x | ? | x | ? | ✓ | ✓ | ? | ✓ | ? | x | ? | ? | ✓ | ✓ | ✓ | ✓ | ✓ | ? | ? | x | x | ? | ? | ? |

| ✓ | ? | x |  |
| --- | --- | --- | --- |

**Abbreviations**: ROB, risk of bias

✓ indicates low ROB/low concern regarding applicability

x indicates high ROB/high concern regarding applicability

? indicates unclear ROB/unclear concern regarding applicability

NA; Not Applicable
