# Supplementary material for: Prognostic models in COVID-19 infection that predict severity: a systematic review
Source: Eur J Epidemiol. 2023 Feb 25;38(4):355–72. doi: 10.1007/s10654-023-00973-x (PMC9958330; doi:10.1007/s10654-023-00973-x)
Supplement: Supplementary file 6 — Supplementary file6 (DOCX 35 KB) [file 10654_2023_973_MOESM6_ESM.docx]

**Table 5: Supplemental material: Definitions of Covid-19 severity or critical illness**

| **Author (year)** | **Definition of severity and critical illness** |
| --- | --- |
| Zhou Y et al. (2020) | Severe Covid-19: Severe cases should meet one major criterion (septic shock with need for vasopressors or respiratory failure requiring MV) or at least three minor criteria RR ≥30 breaths/min, PaO_2_/FiO_2_ ≤250 mmHg, multilobar infiltrates, confusion/disorientation, uremia (BUN ≥20 mg/dL), leukopenia (white blood cell count <400 cells/μL), thrombocytopenia (platelet count <100,000/μL), hypothermia (core temperature <36˚C), and hypotension requiring aggressive fluid resuscitation. |
| Ageno et al. (2021) | Severe outcome was defined as composite of need for non-invasive MV, need for orotracheal intubation, or death, whichever came first. |
| Bello-Chavolla et al. (2020) | Severe COVID-19 was determined as a composite event of either death, ICU admission requirement or MV. |
| Bennouar et al. (2020) | Progression to the severe form defined by one of the following criteria: admission to ICU, use of MV or death. |
| Bennouar et al. (2021) | Severity was defined according to the following standard criteria: severe form by one of the following criteria: 1) shortness of breath: RR > 30 breaths/min in the resting state; 2) SpO_2_ < 93% or 3) PaO_2_/FIO_2_ < 300 mmHg. Very severe or ‘‘critical form” was defined by the presence of one of the following criteria: 1) respiratory failure requiring MV; 2) shock or 3) multi-organ failure, requiring ICU. |
| Boero et al. (2021) | Critical illness was defined by the occurrence of either; admission to ICU, need for invasive IMV, or death due to COVID-19 within a follow-up of 30 days post-admission. Supplementary oxygen support or non-invasive ventilation (NIV) was considered favorable outcomes. |
| Chen et al. (2021) | Severe cases; respiratory failure needing MV; (2) shock; or (3) other organ failure requiring monitoring and treatment in the ICU. |
| De Socio et al. (2021) | Critical COVID-19 illness defined as admission to the ICU, invasive ventilation, or death. |
| Fernandes et al. (2021) | Severe prognosis, i.e., ICU admission, use of MV or death. |
| Gao et al. (2021) | Critical illness was defined as admission to ICU, receiving invasive ventilation, or death. |
| Liu Li et al. (2021) | Patients meeting any of following items should be identified as critically ill cases: shock; respiratory failure requires MV; complication of other organ failure occur; and patients require treatment in ICU. |
| Li S et al. (2021) | The severity of disease was retrospectively classified as severe/critical or moderate base on the worst situation during hospitalization. Criteria for severe cases were as following:(1) respiratory distress, RR ≥ 30 beats/min; (2) SpO_2_ ≤ 93%; or (3) PaO_2_/ FiO_2_≤ 300 mmHg. Criteria for critical patients were: (1) respiratory failure needing MV; (2) shock; (3) other organ failure needing ICU monitoring treatment. |
| Liang et al. (2020) | Critical illness: composite measure of admission to the ICU, invasive ventilation, or death. |
| Liu Q et al. (2021) | Critical illness: composite of admission to ICU, respiratory failure requiring MV, shock during hospitalization, or death. |
| Liu, J et al. (2020) | Critically ill cases were defined as cases meeting any of the following criteria: 1) respiratory failure and requiring MV; 2) shock; or 3) another organ failure that requires ICU care. |
| Marcos et al. (2021) | Severity of disease progression; defined as the use of MV or death. |
| Myrstad et al. (2020) | Severe disease was defined as a composite measure of death during hospitalization or ICU treatment for any reason during the hospital stay. In-hospital mortality was defined as death during hospital stay for any reason, related or unrelated to the covid-19 infection. |
| Prower et al. (2021) | Primary outcome was a composite of cardiac arrest, unplanned critical care admission or death within 24 hours. |
| Purkayastha et al. (2021) | Critical or severe if they reached any of the following endpoints: ICU admission, MV, or death. |
| Schalekamp et al. (2021) | Critical illness was defined as admission to the ICU for invasive MV and/or death. |
| Schöning et al. (2021) | Severe COVID-19 - a composite endpoint of admission to intensive care, or death from any cause. |
| Shi Y et al. (2021) | Critical illness, defined as MV or in-hospital death. |
| Su Y et al. (2020) | EDRF was defined as changes in the respiratory component of the SOFA score at day 3 (1SOFAresp = SOFAresp at day 3–SOFAresp on admission), in which the positive value reflects clinical deterioration. IRS was defined as the use of high flow nasal cannula oxygen therapy, noninvasive or invasive MV. |
| Tu et al. (2021) | Severe, with one of the following; (a) RR ≥30 breaths / min, (b) mean SpO_2_ at rest ≤93%, (c) PaO_2_/FiO_2_ ≤300 mmHg (1 mmHg = 0.133 kPa), or (d) pulmonary imaging showing an increase in manifestations of >50% within 24~48 h, (4) critical, with one of the following, (a) respiratory failure requiring MV, (b) shock, or (c) ICU admission due to multiple-organ failure. |
| Ucan et al. (2021) | Severe COVID-19 was defined by at least one of the following: ARDS (RR ≥ 30 breaths/minute), SpO_2_ < 90%, requirement of noninvasive, or invasive m MV or admission to the ICU. |
| Woo et al. (2021) | Severe COVID-19: ICU admission, use of MV, and/or death within 14 days of hospitalization |
| Wu et al. (2020) | Patients were labelled as having a “severe disease” if at least one of the following criteria were met during hospitalization: (1) respiratory failure requiring MV; (2) shock; (3) ICU admission;(4) organ failure; or (5) death. |
| Xiao et al. (2020) | Severe disease: SpO_2_ ≤ 93% at rest; ARDS (RR≥30 breaths/min); obvious lesion progression in chest imaging within 24–48 h > 50%; PaO_2_/FiO_2_ ≤300 mmHg. Patients with organ failure and respiratory failure were considered to have a critical disease. |
| Xu F et al. (2021) | Severe COVID19 cases were defined as patients with fever plus one of RR >30 breaths/minute, severe respiratory distress, or SpO_2_ ≤93% in room air. |
| Xu J et al. (2021) | Severity was defined as discharge or death, and by whether the subject required continued hospitalization |
| Yao et al. (2021) | Critical COVID19: (1) have respiratory failure and need MV; (2) have shock; (3) combined with other organ failures which require ICU monitoring treatment. |
| Yu Y et al. (2020) | Severe COVID-19 cases were defined as patients having dyspnea, respiratory frequency ≥ 30/min, SpO_2_ ≤ 93%, PaO_2_/FiO_2_ < 300, and/or lung infiltrates > 50% within 24- 48 hours. |
| Zhang B et al. (2020) | Severity of COVID-19 according to the newest COVID-19 guidelines released by the National Health Commission of China and the guidelines of the American Thoracic Society for community-acquired pneumonia. Thirty-day poor outcome is defined as meeting at least one of the following criteria within 30 days after admission to hospital: respiratory failure requiring MV, shock, ICU admission, multiple organ dysfunction or death. |
| Assal et al. (2022) | Severe COVID-19 was defined as requiring oxygen therapy (flow rate of more than 8 L/min or use of high-flow oxygen cannula), non-invasive or invasive MV at any time point during hospitalization. |
| Shalmon et al. (2022) | Critical illness status was defined as meeting one or more of these patient conditions: admission to ICU, need for MV, ECMO or death within 1 month following admission |
| Shankar et al. (2022) | COVID-19 severity was graded based on any of the following criteria: patients with R) > 30 breaths/min, SpO_2_ < 93%, PaO_2_/FiO_2_ ≤ 300 mmHg, or requirement of artificial ventilation |
| Shi et al. (2022) | Severe patient was defined as (1) RR ≥ 30 breaths/min; (2) SpO_2_ in resting state ≤ 93%; (3) respiratory distress; (4) the presence of shock; (5) PaO_2_≤ 300 mmHg; (6) respiratory failure requires MV; (7) patients with organ failure need ICU monitoring and treatment. |
| Tang et al. (2022) | Severity = disease progression = patients with disease progression consisted of patients requiring ICU care and dead patients. |
| Vela et al. (2022) | Severe COVID-19: hospital admission, transfer to ICU and death |
| Wong et al. (2021) | Severe COVID-19 was considered if the individual is an inpatient or if the cause of mortality |
| Xiong et al. (2022) | COVID-19 severity was determined based on the Diagnosis and Treatment Protocol for COVID-19 (7^th^ edition) (Diagnosis and Treatment Protocol for Novel Coronavirus Pneumonia (Trial Version 7). |
| Zhang et al. (2022) | Severe/critical type: admission to the ICU that required MV or had a fraction of inspired oxygen concentration of at least 60% or more. |
| Zhao et al. (2022) | Patients were defined to have severe COVID-19 if they met one of the following criteria: (1) respiratory distress with RR ≥30/min; (2) SpO_2_ ≤93% at rest and (3) PaO_2_/FiO_2_ ≤300 mmHg. |
| Gómez et al. (2021) | Deceased and/or ICU inpatients were con­sidered as severe cases, whereas those patients after hospital discharge were considered as non-severe. |
| Monterde et al. (2021) | Critical illness: the need for invasive MV, transfer to ICU, or in-hospital death |
| Muto et al. (2021) | Critical illness and severe illness were defined using the National Institutes of Health classification criteria: critical illness, for individuals who have respiratory failure, septic shock, and/or multiple organ dysfunction, and severe illness, for individuals who have SpO_2_ <94% in room air at sea level, PaO_2_/FiO_2_ <300 mmHg, respiratory frequency >30 breaths/min, and/or lung infiltrates >50%. |
| Rinderknecht et al. (2021) | Critical state encompassed patients with a reported ICD code for sepsis, septic shock, or respiratory failure (e.g., ARDS) within the 28 days after COVID-19 diagnosis or patients being flagged as deceased in the database without having been in a critical state before COVID-19 diagnosis |
| Bennett et al. (2021) | Clinical severity: hospitalization with death, discharge to hospice, invasive MV support, or ECMO |
| Xu et al (2021) | Severe cases – those who required treatment in the ICU and ventilation in the ICU |
| Sengel et al. (2021) | Severe cases were defined as at least one of the following, RR≥30 breaths/min, resting SpO_2_ <94%, PaO_2_/FiO_2_ ≤300 mmHg or requirement of MV. Progression to severe COVID-19 was defined as appearing one or more of the parameters mentioned above 24 hours from admission |
| Li Xue-Lian et al. (2021) | For diagnosis of severe COVID-19 group, at least one of the following conditions should be met according to WHO guidelines, complemented by the COVID-19 Diagnosis and Treatment Guidance (2020) of China (version 6.0): (1) RR ≥ 30 breaths/min; (2) SpO_2_ (resting status) ≤ 93% in the resting state; or (3) PaO_2_/FiO_2_ ≤ 300 mmHg (1 mm Hg = 0.133 kPa) |
| Huang Jiana et al. (2021) | The degree of severity of Covid-19 was defined as following: (1) Mild: slight clinical symptoms without CT abnormality (2) Moderate: fever, respiratory symptoms, etc., CT presented with pneumonia. (3) Severe: complied any of the following: (1), RR ≥30/min; (2) at rest, SpO_2_ ≤93%; (3) PaO_2_ /FiO_2_ ≤ 300 mm Hg; (4) Pulmonary imaging showed that the lesion progressed more than 50% within 24-48 hours. (4) Critically severe: complied with any of the following: (a) Respiratory failure and required MV; (2) Shock; (3) Combined with another organ failure required ICU. |
| Ma et al. (2021) | The severity of the case was classified according to the worst status during hospital stay. Severe cases – those with severe pneumonia, ARDS, sepsis, or septic shock) |
| An et al. (2022) | Severe illness: respiratory distress syndrome, RR ≥30/min, SpO_2_saturation [measured after 5 minutes of rest] ≤93%, or oxygenation index [PaO_2_/FiO_2_] ≤300 mmHg). Critical illness: respiratory failure requiring intubation, shock, other organ failures, or admission to the ICU |
| Gurusamy et al. (2021) | Clinical classification is as follows: 1) Mild: Patients with uncomplicated upper respiratory tract infection (may have mild symptoms such as fever, cough, sore throat, nasal congestion, malaise, headache, RR <24 breath/min, SpO_2_ >94% in room air); 2) Moderate: Pneumonia with no signs of severe disease (RR ≥24-30 breath/min, SpO_2_ ≤94% (90-94) in room air); 3) Severe: ARDS with mean SpO_2_ <90%, RR >30 breaths/min, respiratory failure with shock, and multiorgan dysfunction |
| Haimovich et al. (2020) | respiratory failure within 24 hours of admission as defined by oxygen requirement of greater than 10 L/min by low-flow device, high-flow device, non-invasive or invasive ventilation, or death. |
| Han et al. (2022) | The severity of COVID-19 patients (severe vs. non-severe) was defined at admission, according to American Thoracic Society (ATS) guidelines for community-acquired pneumonia (CAP) |
| Jiang et al. (2022) | Criteria of severe or critical COVID-19 were as follows: 1) ARDS (RR, ≥30 breaths/ minute); 2) SpO_2_ ≤93% or PaO_2_/FiO_2_ ≤300 mmHg at rest; 3) respiratory failure requiring MV; 4) shock; and 5) other organ failure requiring monitoring and treatment in the ICU |
| Lee et al. (2022) | Severe Covid-19 pneumonia was defined as at least one of the followings: (1) resting SpO_2_ ≤ 93% in room air, or (2) PaO_2_/FiO_2_ ≤ 300 mmHg or requirement of MV |
| Leyderman et al. (2021) | The severe COVID-19 is a specific c type of the systemic inflammatory response, in which the manifestation of organ dysfunction, as a rule, begins with damage of the lungs |
| Liu et al. (2021) | Severe disease progression was defined as the clinical type worsened or death. |
| Nadasdi et al. (2022) | Disease severity was defined on the WHO ordinal scale: (0) never exposed to SARS-CoV-2, (1) no prior hospitalization due to COVID-19 (2) prior hospitalization due to COVID-19 (3–6) hospitalized and with acute COVID-19, i.e. (3): not requiring O_2_ therapy (WHO ordinal scale: 3), 4)required O_2_ therapy via nasal cannula only (WHO ordinal scale: 4), (5) ICU admission required (due to invasive MV or other cause) and survived ( WHO ordinal scale: 6 + 7), (6) died in COVID-19 (WHO ordinal scale: 8). Neither high-flow oxygen nor non-invasive ventilation therapy was extensively used in the participating study centers during the sample collection period, and therefore no patient was classified into WHO ordinal scale 5. |
| Nuevo‐Ortega et al. (2022) | The primary outcome was the development of severe disease, defined by the presence of one of the following criteria: a respiratory failure that needs an FiO_2_≥0.6, shock or severe dysfunction of another organ, or death. The secondary outcome was vital status at hospital discharge (alive/dead) |
| Patel et al. (2021) | The need for and timing of invasive MV; Secondary outcomes were mortality, rate of intubation, length of stay, and rate of nosocomial infections in treated initially with high-flow nasal therapy |
| Peng et al. (2022) | Severity was classified according to WHO guidelines. According to the WHO guidelines, the cases were classified as mild, moderate, severe, or critical, including SRDS, sepsis, septic shock]. As the primary outcome, deterioration refers to the progression from mild or moderate to severe, critical or fatal. |
| Chang et al. (2022) | Severity was classified according to the latest version of the guidelines on the Diagnosis and Treatment of COVID-19 by the National Health Commission of China, patients infected with COVID-19 are clinically defined as mild, moderate, severe and critical. Among them, mild patients present light symptoms without signs of pneumonia, and moderate patients start to show fever, respiratory symptoms and pneumonia by imaging. For severe patients, most of them develop dyspnea and/or hypoxia within one week, and even critical cases progress rapidly to ARDS, septic shock, irreversible metabolic acidosis, coagulation dysfunction and multi-organ dysfunction. |
| Chen et al. (2022) | Severe disease was defined as those patients that had one of the following conditions: (a) respiratory failure requiring MV; (b) shock; (c) organ failure and/or need for ICU |

ICU: intensive care unit; SOFA: sequential organ failure assessment; SpO_2_: oxygen saturation level; PaO_2_: oxygen pressure; ARDS: acute respiratory distress syndrome; FiO_2_: oxygen concentration; MV: mechanical ventilation; ECMO: extracorporeal membrane oxygenation; RR: respiratory rate.
